# Supplementary material for: Iodide-enhanced palladium catalysis via formation of iodide-bridged binuclear palladium complex
Source: Commun Chem. 2020 Mar 31;3:41. doi: 10.1038/s42004-020-0287-0 (PMC9814094; doi:10.1038/s42004-020-0287-0)
Supplement: Supplementary file 1 — Supplementary Information [file 42004_2020_287_MOESM1_ESM.pdf]

Supplementary Methods

General Information

All reactions were performed under nitrogen. The reagents used for experiments were purchased from Sigma-Aldrich, Acros Organics, TCI, and Alfa Aesar and used as received unless otherwise noted. DMF, DCE, DCM, DCB and DMSO were distilled from CaH_2 under nitrogen and stored in glove box. ^1H, ^{13}C and ^{19}F NMR spectra were recorded at 400, 100.6 and 376.5 MHz respectively using Bruker Avance 400 MHz spectrometer. HPLC yields were measured on Waters ACQUITY. High resolution mass spectra (HRMS) were determined on Waters Micromass GCT Premier Mass Spectrometer at Shanghai institute of organic chemistry. Note that $[NO][BF_4]$ should be stored in glove box and the freshly opened $[NO]BF_4$ chemical gives the best result.

Experimental Section

Supplementary Table 1. Optimization studies resulting in discovery of benefited effect of Iodide[a]

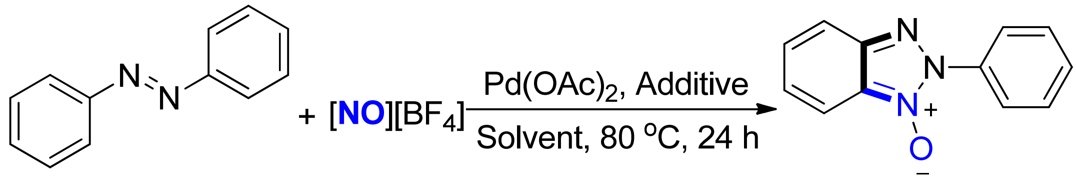

1a 2a 3a

Entry	Additive	Solvent	Yield (%)[b]
1	-	DCE	40
2	-	DCM	12
3	-	PhCl	43
4	-	DCB	46
5	-	DMF	0
6	-	DMSO	0
7	-	CH_3CN	0
8	TBAC	DCB	76
9[c]	TBAI	DCB	73
10[d,e]	TBAI	DCB	78
11[e]	TBAI	DCB	62
12[f]	TBAI	DCB	85
**13[g]**	**TBAI**	**DCB**	**90**
14[g, h]	TBAI	DCB	90
15[g, i]	TBAI	DCB	89
16[g, j]	TBAI	DCB	90
17[g, k]	TBAI	DCB	89
18[g, l]	TBAI	DCB	87
19[m]	TBAI	DCB	82
20[n]	TBAI	DCB	0

[a] Reaction conditions: azobenzene (0.2 mmol, 0.0364 g), [NO][BF_4] (0.4 mmol, 0.0468g), $Pd(OAc)_2$ (10 mol%, 0.0045 g) , additive (10 mol%), 1.5 mL of solvent, 80 °C, 24 h. [b] Isolated yields. [c] The reaction was conducted at 70 °C. [d] 5 mol% of TBAI was used. [e] 5 mol% of $Pd(OAc)_2$ was used. [f] 3 mol% $Pd(OAc)_2$ and 3 mol% TBAI were used. [g] 1 mol% $Pd(OAc)_2$ and 1 mol% TBAI were used. [h] 2 mol% TsOH was added. [i] 2 mol% HOAc was added. [j] 2 mol% PivOH was added. [k] 2 mol% benzoic acid was added. [l] 2 mol% 4-nitrobenzoic acid was added. [m] 0.4 mmol scale reaction was conducted with 0.5 mol% $Pd(OAc)_2$ and 0.5 mol% TBAI. [n] The reaction was conducted without $Pd(OAc)_2$.

Identification of iodide-bridged binuclear palladium complex from catalytic reaction system:

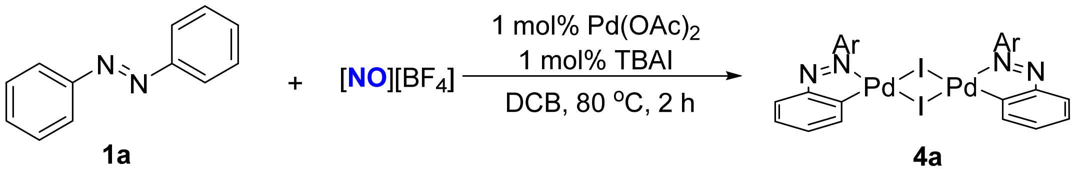

Procedure: In a glove box, 250 mL flask equipped with a stir bar was charged with **1a** (20 mmol, 3.6440 g), Pd(OAc)$_2$ (0.2 mmol, 0.0449 g), TBAI (0.2 mmol, 0.0738 g), [NO][BF$_4$] (40 mmol, 4.6724 g) and DCB (50 mL). The flask was sealed and removed out of the glove box. The reaction mixture was stirred at 80 °C for 2 h. After cooling to room temperature, the reaction mixture was added hexane (100 mL) and freezed in refrigerator for 24 h to produce some red solid. Poured out the liquid and the solid residue was washed with Et$_2$O (2× 15 mL), the red solid was then dissolved in CH$_2$Cl$_2$ and by diffusion of hexane to afford **4a** as red needle crystals. The structure of **4a** was established by single-crystal X-ray diffraction analysis (see below).

After the screening of methyl, ethyl and *n*-butyl substituents on azobenzenes, we found only (*E*)-bis(4-butylphenyl)diazene could be used to affod soluble iodide-bridged binuclear palladium complex, an analogue of **4a**, and therefore (*E*)-bis(4-butylphenyl)diazene was used to prepare iodide-bridged binuclear palladium complex with cyclopalladated (*E*)-bis(4-butylphenyl)diazene for investigation of the reaction mechanism.

Preparation of iodide-bridged binuclear palladium complex with cyclopalladated (*E*)-bis(4-butylphenyl)diazene 4b.

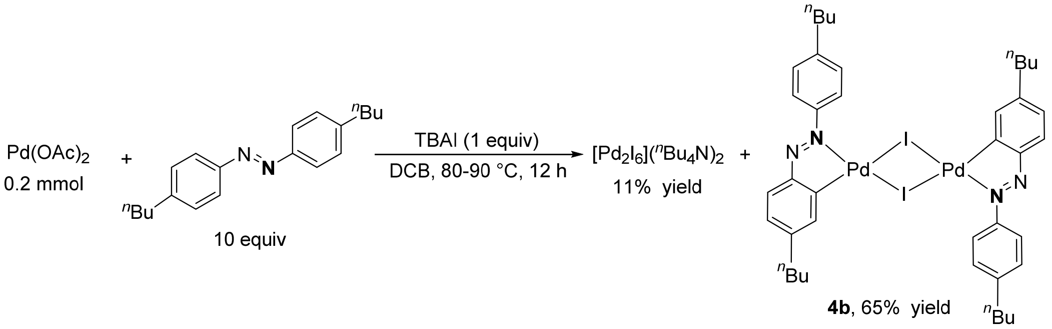

Procedure: In a glove box, a 25 mL of the Schlenk tube equipped with a stir bar was charged with Pd(OAc)$_2$ (0.2 mmol, 0.0449), TBAI (0.2 mmol, 0.0738 g), **1b** (2 mmol, 0.5888 g) and DCB (50 mL). The tube was sealed and removed out of the glove box. The reaction mixture was stirred at 90 °C for 12 h. The reaction mixture was cooled to room temperature, filtered and the filtrate was concentrated under reduced pressure. The residue was purified by chromatography on silica gel (eluent: 5-10% CH$_2$Cl$_2$/hexane) to provide the corresponding product **4b** (Binuclear Pd^{II}-I complex, red solid, 68.2 mg, 65%) and [Pd$_2$I$_6$](^{n}Bu$_4$N)$_2$ (black solid, 16.5 mg, 11%). **^{1}H NMR (400 MHz, CDCl$_3$) δ:** 7.83 (d, *J* = 7.7 Hz, 1H), 7.69 (s, 1H), 7.64 (d, *J* = 6.2 Hz, 2H), 7.28-7.25 (m, 2H), 7.05 (d, *J* = 7.7 Hz, 1H), 2.70-2.59 (m, 4H), 1.73-1.57 (m, 4H), 1.41-1.36 (m, 4H), 0.97-0.92 (m, 6H). **^{13}C NMR (100 MHz, CDCl$_3$) δ:** 162.7, 159.9, 149.4, 145.6, 137.6, 129.7, 128.2, 126.0, 123.6, 36.4, 35.4, 33.2, 33.1, 22.3, 22.2, 13.9. Anal. Calcd. for $C_{40}H_{50}I_2N_4Pd_2$: C, 45.60; H, 4.78; N, 5.32; Found: C, 45.54; H, 4.85; N, 5.31. The structure of **4b** was established by single-crystal X-ray diffraction analysis (see below).

The reactivity and catalytic activity of iodide-bridged binuclear palladium complexes:

Stoichiometric reaction of 4b with [NO][BF$_4$] to form 3b

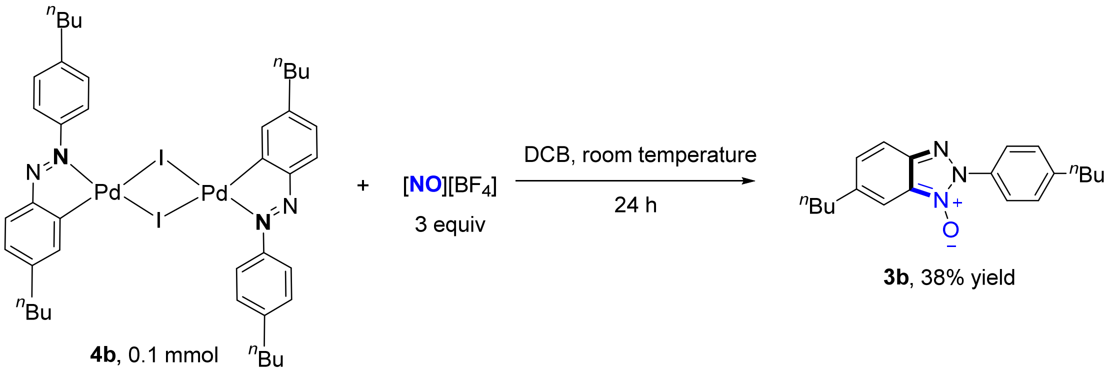

In a glove box, a 25 mL of the Schlenk tube equipped with a stir bar was charged with **4b** (0.1 mmol, 0.1053 g), [NO][BF$_4$] (0.6 mmol, 0.0701 g) and DCB (5 mL). The tube was sealed and removed out of the glove box. The reaction mixture was stirred at room temperature for 24 h. Upon completion, the reaction mixture was purified directly by chromatography on silica gel (eluent: 5-10% ether/hexane) to provide the corresponding product **3b** (yellow oil, 24.6 mg, 38%).

Studies of the catalytic activity of 4b:

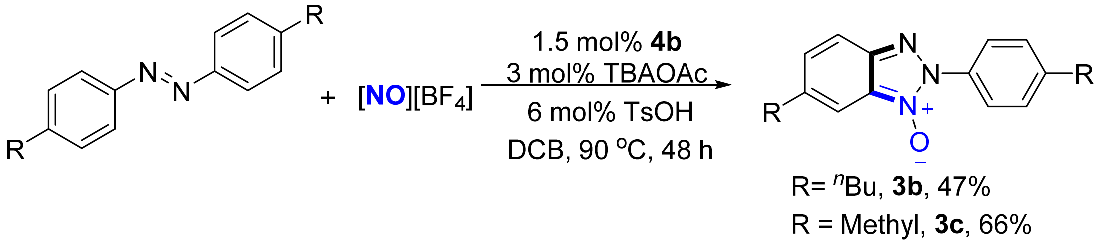

Procedure: In a glove box, a 25 mL of the Schlenk tube equipped with a stir bar was charged with **4b** (1.5 mol%, 0.0032 g), TBAOAc (3 mol%, 0.0019 g), anhydrous p-toluenesulfonic acid (6 mol%, 0.0021 g), **1b** (0.2 mmol, 0.0589 g) or **1c** (0.2 mmol, 0.0421 g), [NO][BF$_4$] (0.6 mmol, 0.0701 g) and DCB (1.5 mL). The tube was sealed and removed out of the glove box. The reaction mixture was stirred at 90 °C for 48 h. **3b** and **3c** were obtained with 47% and 66% yields respectively (yields given in parentheses were isolated yields under standard reaction conditions). **4b** provided the similar yields to the combination of 3 mol% Pd(OAc)$_2$ and 3 mol% TBAI (see below).

Conversion of the acetate-bridged binuclear palladium complex to the corresponding iodide-bridged binuclear palladium complex and the regeneration of iodide-bridged binuclear palladium complex under the conditions mimicking catalytic reaction:

Preparation of the acetate-bridged binuclear palladium complex with cyclopalladated (*E*)-bis(4-butylphenyl)diazene

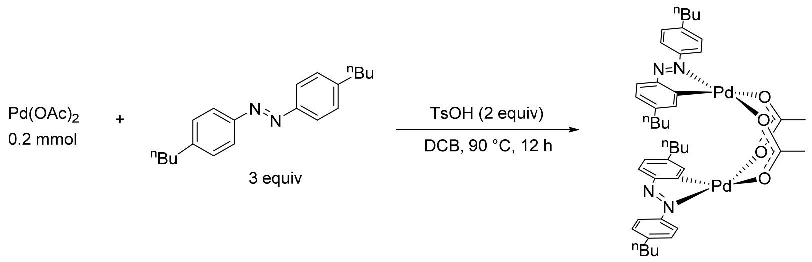

5b, 78%

Procedure: In a glove box, a 25 mL of the Schlenk tube equipped with a stir bar was charged with $Pd(OAc)_2$ (0.2 mmol, 0.0449), anhydrous p-toluenesulfonic acid (0.4 mmol, 0.0689 g), **1b** (0.6 mmol, 0.1766 g) and DCB (3 mL). The tube was sealed and removed out of the glove box. The reaction mixture was stirred at 90 °C for 12 h. Upon completion, the reaction mixture was purified directly by chromatography on silica gel (eluent: 1-5% MeOH/CH_2Cl_2) to provide the corresponding product **5b** (Binuclear Pd^{II}-OAc complex, black red solid, trans : cis = 5.71 : 1, 71.7 mg, 78%). **^{1}H NMR (400 MHz, CDCl$_3$) δ:** 7.56 (d, J = 7.9 Hz, 2H), 7.43 (d, J = 7.9 Hz, 0.35H), 7.39 (d, J = 8.5 Hz, 0.70H), 7.28 (d, J = 8.5 Hz, 4H), 7.04 (d, J = 8.5 Hz, 4H), 6.97 (d, J = 8.5, 0.69H), 6.93 (dd, J = 7.9 Hz, 1.5 Hz, 2H), 6.77 (dd, J = 7.9 Hz, 1.5 Hz, 0.33H), 6.50 (d, J = 1.4 Hz, 0.34H), 6.32 (d, J = 1.4 Hz, 2H), 2.65-2.61 (m, 4.83H), 2.45-2.36 (m, 3.30H), 2.30-2.20 (m, 2.15H), 2.06 (s, 6H), 1.65-1.57 (m, 5H), 1.52-1.43 (m, 4.88H), 1.42-1.27 (m, 10.15H), 0.98-0.89 (m, 14.53H). **^{13}C NMR (100 MHz, CDCl$_3$) δ:** 180.6, 161.3, 155.1, 148.9, 146.8, 145.5, 145.4, 132.9, 132.5, 128.1, 127.9, 127.6, 125.4, 125.2, 123.3, 123.2, 36.5, 36.4, 35.4, 33.3, 32.9, 32.6, 24.4, 22.5, 22.2, 13.8. Anal. Calcd. for $C_{44}H_{56}N_4O_4Pd_2$: C, 57.58; H, 6.15; N, 6.10; Found: C, 57.59; H, 6.18; N, 6.14. The structure of **5b** was established by single-crystal X-ray diffraction analysis (see below).

Conversion of 5b to 4b

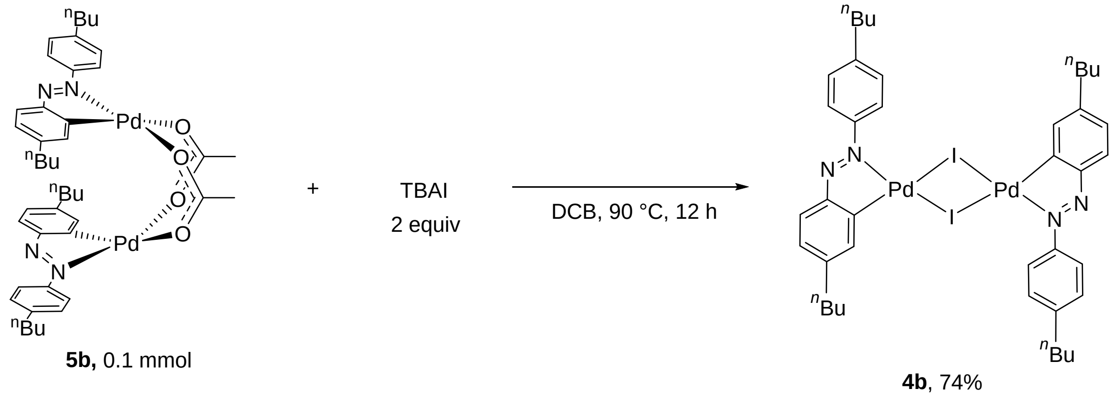

5b, 0.1 mmol

4b, 74%

Procedure: In a glove box, a 25 mL of the Schlenk tube equipped with a stir bar was charged with **5b** (0.1 mmol, 0.0917 g), TBAI (0.4 mmol, 0.0738 g) and DCB (3 mL). The tube was sealed and removed out of the glove box. The reaction mixture was stirred at 90 °C for 12 h. Upon

completion, the reaction mixture was purified directly by chromatography on silica gel (eluent: 5-10% CH_2Cl_2/hexane) to provide the corresponding product **4b** (77.9 mg, 74%).

Experiment for the regeneration of 4b under the conditions mimicking catalytic reaction:

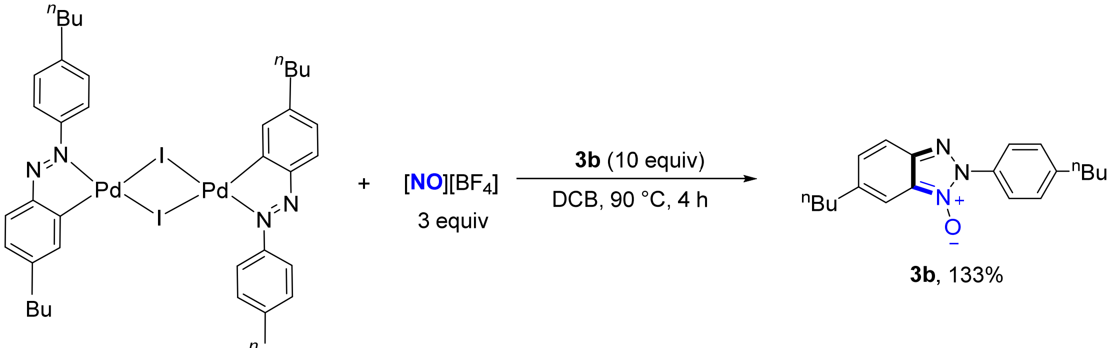

4b, 0.1 mmol
21% Recovered

Procedure: In a glove box, a 50 mL of the Schlenk tube equipped with a stir bar was charged with **4b** (0.1 mol, 0.1053 g), **1b** (1.0 mmol, 0.2944 g), [NO][BF_4] (0.3 mmol, 0.0351 g) and DCB (10 mL). The tube was sealed and removed out of the glove box. The reaction mixture was stirred at 90 °C for 4 h. Upon completion, the reaction mixture was cooled down to room temperature, then NEt_3 (1.5 mmol, 0.1521 g) in 1 mL MeOH was injected by a syringe under N_2 atmosphere to neutralize the acid formed in the reaction and extra [NO][BF_4]. The mixture was diluted with 10 mL of ethyl acetate, filtered through a pad of silica gel, followed by washing the pad of the silica gel with the ethyl acetate (10 mL). The filtrate was concentrated under reduced pressure. The residue was then purified by chromatography on silica gel to provide product **3b** (43.5 mg, 133%), at the same time, **4b** (22.1 mg, 21%) was also recovered.

The catalytic activity and reactivity of [Pd_2I_6](^{n}Bu_4N)$_2$:

Catalytic activity of [Pd_2I_6](^{n}Bu_4N)$_2$

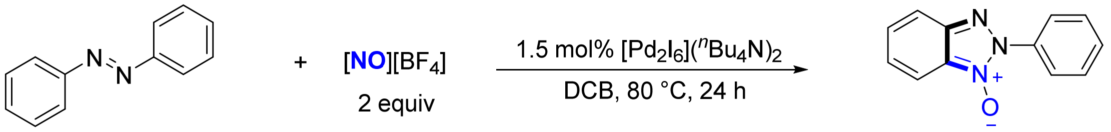

1a **3a**, 82%

Procedure: In a glove box, a 25 mL of the Schlenk tube equipped with a stir bar was charged with [Pd_2I_6](^{n}Bu_4N)$_2$ (1.5 mol%, 0.0043 g), **1a** (0.2 mmol, 0.0364 g), [NO][BF_4] (0.4 mmol, 0.0468g) and DCB (1.5 mL). The tube was sealed and removed out of the glove box. The reaction mixture was stirred at 80 °C for 24 h. Upon completion, the reaction mixture was diluted with 10 mL of ethyl acetate, filtered through a pad of silica gel, followed by washing the pad of the silica gel with the ethyl acetate (20 mL). The filtrate was concentrated under reduced pressure. The residue was then purified by chromatography on silica gel to provide product **3a** (34.9 mg, 82%).

Transformation of $[Pd_2I_6](^nBu_4N)_2$ to 4b

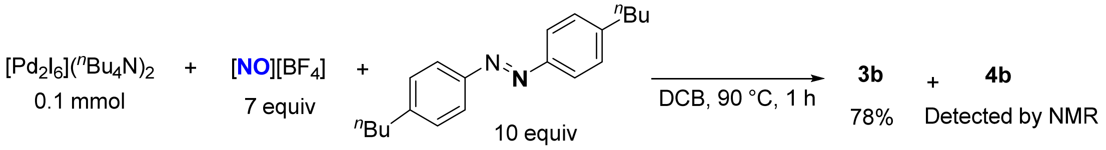

Procedure: In a glove box, a 100 mL of the Schlenk tube equipped with a stir bar was charged with $[Pd_2I_6](^nBu_4N)_2$ (0.1 mmol, 0.1433 g), [NO][BF_4] (0.7 mmol, 0.0818 g), **1b** (1.0 mmol, 0.2944 g) and DCB (10 mL). The tube was sealed and removed out of the glove box. The reaction mixture was stirred at 90 °C for 4 h. Upon completion, the reaction mixture was cooled down to room temperature, then NEt_3 (1.5 mmol, 0.1521 g) in 1 mL MeOH was injected by a syringe under N_2 atmosphere to neuthralize the acid formed in the reaction and extra [NO][BF_4]. The mixture was diluted with 10 mL of ethyl acetate, filtered through a pad of silica gel, followed by washing the pad of the silica gel with the ethyl acetate (10 mL). The filtrate was concentrated under reduced pressure. The residue was then purified by chromatography on silica gel to provide product **3b** (25.3 mg, 78%), at the same time, **4b** was just detected by ^1H NMR, but no pure compound was isolated because of its small amount.

Stoichiometric reaction of aceatate-bridged binuclear palladium 5a to generate 3a:

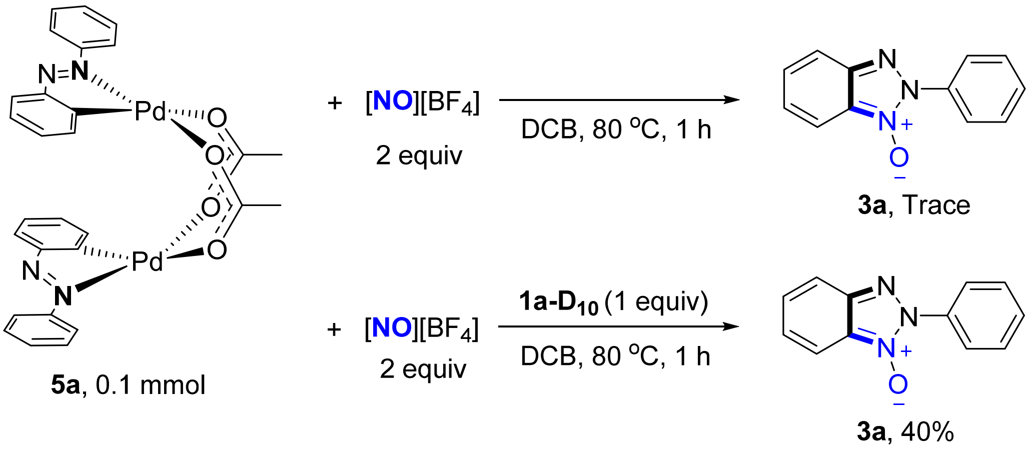

Procedure: In a glove box, a 50 mL of the Schlenk tube equipped with a stir bar was charged with **5a** (0.1 mol, 0.0693 g), **1a-D$_{10}$** (0.2 mol, 0.0385 g), [NO][BF_4] (0.4 mmol, 0.0468 g) and DCB (5 mL). The tube was sealed and removed out of the glove box. The reaction mixture was stirred at 80 °C for 1 h. Upon completion, the reaction mixture was cooled down to room temperature, diluted with 10 mL of ethyl acetate, filtered through a pad of silica gel, followed by washing the pad of the silica gel with the ethyl acetate (20 mL). The filtrate was concentrated under reduced pressure. The residue was then purified by chromatography on silica gel, the yield of **3a** was determined by ^1H NMR using dibromomethane as the internal standard.

Kinetic experiments to evaluate activity of catalyst systems and the order in 4b:

Kinetic experiments to evaluate activity of catalyst systems

Procedure: In a glove box, a 100 mL flask equipped with a stir bar was charged with $Pd(OAc)_2$ (3 mol%, 0.0134 g) or **4b** (1.5 mol%, 0.0316 g), anhydrous p-toluenesulfonic acid (6 mol%, 0.0206 g), **1c** (2 mmol, 0.4204 g), [NO][BF_4] (6 mmol, 0.7008 g), internal stardard 3,4-dichlorotoluene (2

mmol, 0.3221 g) and DCB (15 mL). The flask was sealed and removed out of the glove box. The reaction mixture was stirred at room temperature for 5 min and then 90 °C. The samples were obtained at the indicated time using syringes with long needles which were filled with N_2 before use. Filtered the samples with a pad of silica gel and washed with 1.5 mL MeOH. The concentrations of **3c** were determined by HPLC analysis. The reaction was monitored to approximately 10-15% conversion.

The Initial rate with $Pd(OAc)_2$ and TsOH catalyst system (Catalyst System A)

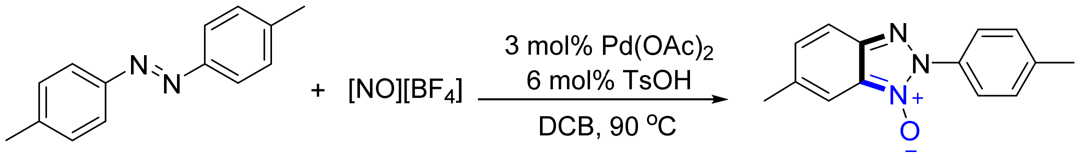

Following the above mentioned procedure: the reaction was carried out with $Pd(OAc)_2$ (3 mol%, 0.0134 g), anhydrous p-toluenesulfonic acid (6 mol%, 0.0206 g), **1c** (2 mmol, 0.4204 g), $[NO][BF_4]$ (6 mmol, 0.7008 g) and internal stardard 3,4-dichlorotoluene (2 mmol, 0.3221 g) in DCB (15 mL).

Supplementary Table 2. Initial rate with catalytic system A

Time (min)	0.5	1	1.5	2	2.5	3	3.5	4
**3b** (M)	0.001459	0.001847	0.002159	0.002806	0.003488	0.004124	0.004904	0.005503
Time (min)	5	6	7	8	9	10	12	14
**3b** (M)	0.006506	0.007366	0.008263	0.008884	0.00935	0.010229	0.010909	0.012376
Time (min)	16	18	20					
**3b** (M)	0.01294	0.013631	0.014167					

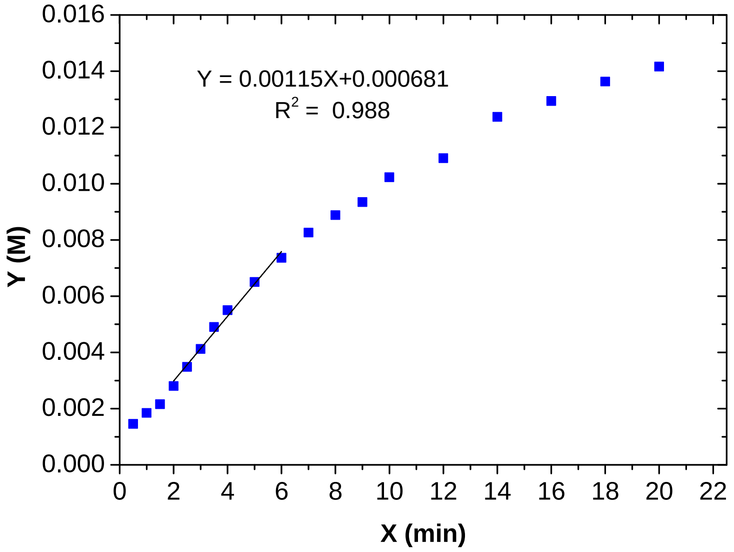

Supplementary Figure 1. Plot of the initial rate versus time with catalytic system A

The initial rate with Pd(OAc)$_2$, TBAI and TsOH catalyst system (Catalyst System B)

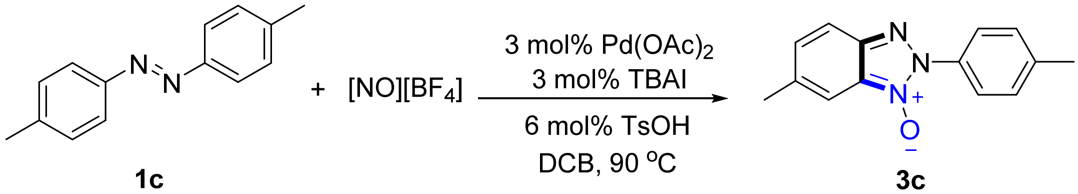

Following the above mentioned procedure: the reaction was carried out with Pd(OAc)$_2$ (3 mol%, 0.0134 g), anhydrous p-toluenesulfonic acid (6 mol%, 0.0206 g), TBAI (3 mol%, 0.0222 g), **1c** (2 mmol) and DCB (10 mL). The flask was sealed and removed out of the glove box. The reaction mixture was stirred at room temperature for 5 min and then 90 °C for 1 h. Put the flask into the glove box after evacuation. [NO][BF$_4$] (6 mmol, 0.7008 g), internal stardard 3,4-dichlorotoluene (2 mmol, 0.3221 g) and DCB (5 mL) were added. The flask was sealed and removed out of the glove box. The reaction mixture was stirred at room temperature for 5 min and then 90 °C.

Supplementary Table 3. Initial rate with catalytic system B

Time (min)	0.5	1	1.5	2	2.5	3	3.5	4
**3b** (M)	0.001308	0.001618	0.001902	0.002571	0.002827	0.003317	0.003931	0.004679
Time (min)	5	6	7	8	9	10	12	14
**3b** (M)	0.006619	0.00877	0.010437	0.01208	0.014246	0.015185	0.01813	0.019825
Time (min)	16							
**3b** (M)	0.022124							

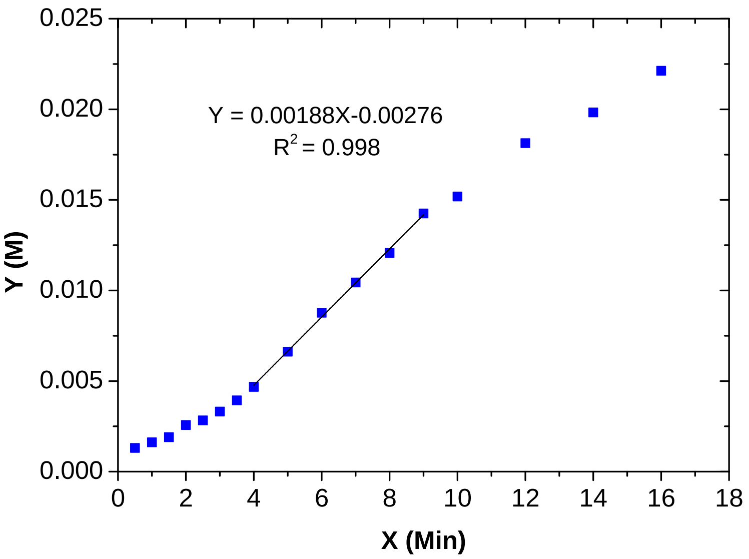

Supplementary Figure 2. Plot of the initial rate versus time with catalytic system B

The Initial rate with 4b catalyst system (Catalyst System C)

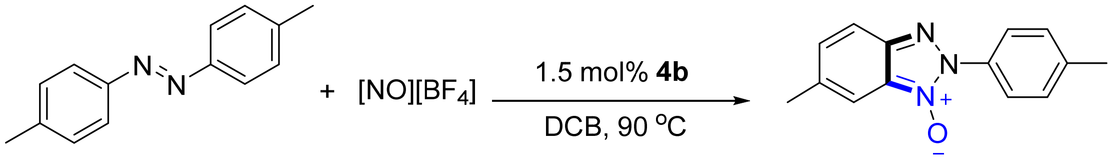

1c **3c**

Following the above mentioned procedure: the reaction was carried out with **4b** (1.5 mol%, 0.0316 g), **1c** (2 mmol, 0.4204 g), [NO][BF_4] (6 mmol, 0.7008 g) and internal stardard 3,4-dichlorotoluene (2 mmol, 0.3221 g) in DCB (15 mL). The flask was sealed and removed out of the glove box. The reaction mixture was stirred at room temperature for 5 min and then 90 °C.

Supplementary Table 4. Initial rate with catalytic system C

Time (min)	0.5	1	1.5	2	2.5	3	3.5	4
**3b** (M)	0.000121	0.00034	0.000708	0.001343	0.00175	0.002467	0.00345	0.004413
Time (min)	5	6	7	8	9	10	12	
**3b** (M)	0.007094	0.009253	0.010807	0.013421	0.014722	0.0162	0.019485	

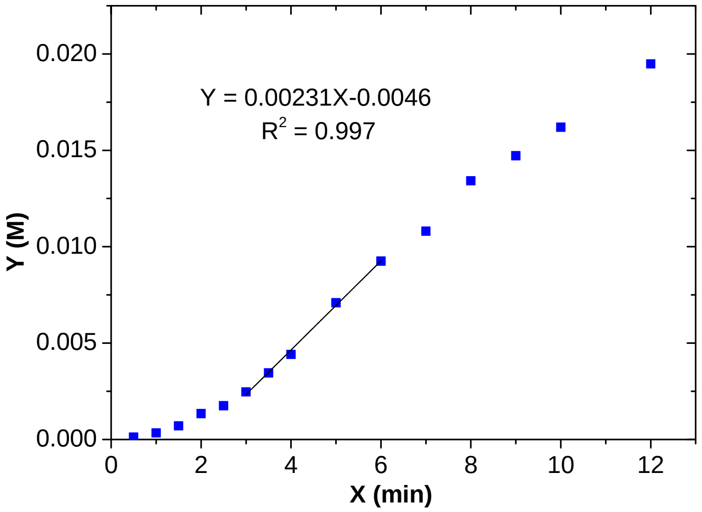

Supplementary Figure 3. Plot of the initial rate versus time with catalytic system C

The initial rate with 5b, TsOH and HOAc catalyst system (Catalyst System D)

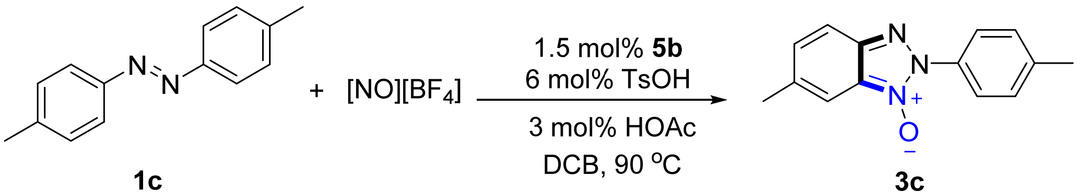

1c **3c**

Following the above mentioned procedure: the reaction was carried out with **5b** (1.5 mol%, 0.0275 g), anhydrous p-toluenesulfonic acid (6 mol%, 0.0206 g), HOAc (3 mol%, 0.0036 g), **1c** (2 mmol, 0.4204 g), [NO][BF_4] (6 mmol, 0.7008 g) and internal stardard 3,4-dichlorotoluene (2 mmol, 0.3221 g) in DCB (15 mL).

Supplementary Table 5. Initial rate with catalytic system D

Time (min)	0.5	1	1.5	2	2.5	3	3.5	4
**3b** (M)	0.000321	0.000526	0.000783	0.001483	0.002137	0.002485	0.003242	0.004335
Time (min)	5	6	7	8	9	10	12	14
**3b** (M)	0.005391	0.006487	0.007937	0.008848	0.010131	0.010971	0.012534	0.013543
Time (min)	16	18	20					
**3b** (M)	0.014175	0.014794	0.016159					

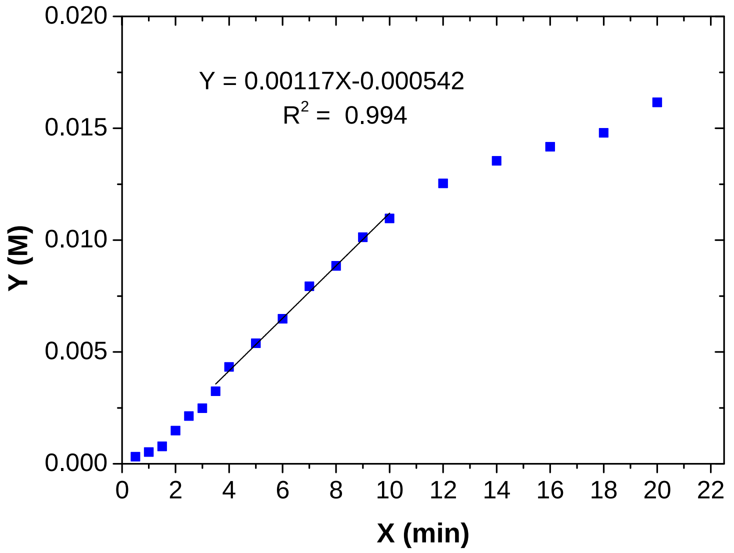

Supplementary Figure 4. Plot of the initial rate versus time with catalytic system D

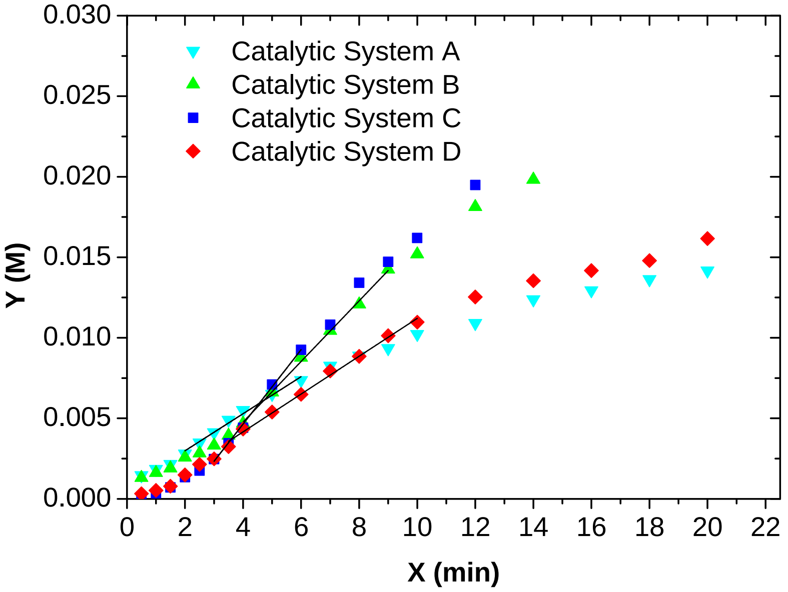

Supplementary Figure 5. Kinetic profiles for the formation of **3b** using different catalytic systems

The initial rates were 0.00117, 0.00188, 0.00231, and 0.00115 with regard to condition A, B, C, and D respectively**.** The initial rate of binuclear Pd-I species **4b** is two times as fast as $Pd(OAc)_2$ and TsOH catalytic system and is more fast than $Pd(OAc)_2$, TBAI and TsOH catalytic system, therefore indicating that **4b** is a kinetically competent catalyst for the reaction. The initial rate of binuclear Pd-OAc species **5b** is almost the same as $Pd(OAc)_2$ and TsOH catalytic system, thus **5b** is the kinetically competent catalyst when without the addition of TBAI.

Determination of the order in 4b

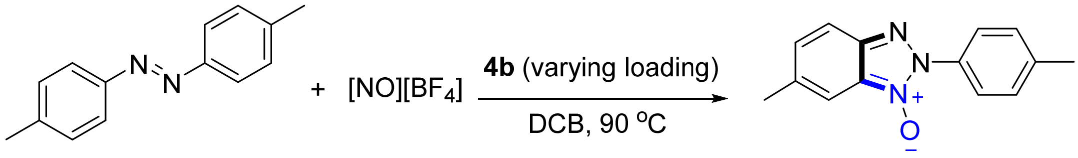

1c **3c**

The order in **4b** was obtained by determining the initial rate of reactions with different [**4b**]. **Procedure:** In a glove box, a 100 mL flask equipped with a stir bar was charged with 1.5 mol%, 1 mol%, 0.75 mol%, 0.5 mol% or 0.25 mol% of **4b**, **1b** (2 mmol, 0.4204 g), $[NO][BF_4]$ (6 mmol, 0.7008 g), internal stardard 3,4-dichlorotoluene (2 mmol, 0.3221 g) and DCB (15 mL). The flask was sealed and removed out of the glove box. The reaction mixture was stirred at room temperature for 5 min and then 90 °C. The reactions were monitored to approximately 2-15% conversion.

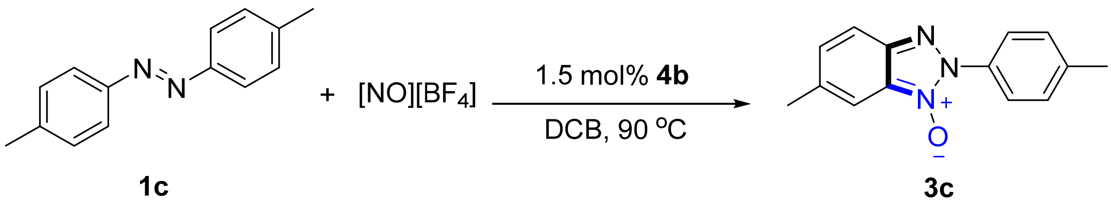

Following the above mentioned procedure: the reaction was carried out with **4b** (1.5 mol%, 0.0316 g), **1c** (2 mmol, 0.4204 g), [NO][BF_4] (6 mmol, 0.7008 g) and internal stardard 3,4-dichlorotoluene (2 mmol, 0.3221 g) in DCB (15 mL). The flask was sealed and removed out of the glove box. The reaction mixture was stirred at room temperature for 5 min and then 90 °C.

Supplementary Table 6. Initial rate with 1.5 mol% of **4b**

Time (min)	0.5	1	1.5	2	2.5	3	3.5	4
**3b** (M)	0.000121	0.00034	0.000708	0.001343	0.00175	0.002467	0.00345	0.004413
Time (min)	5	6	7	8	9	10	12	
**3b** (M)	0.007094	0.009253	0.010807	0.013421	0.014722	0.0162	0.019485	

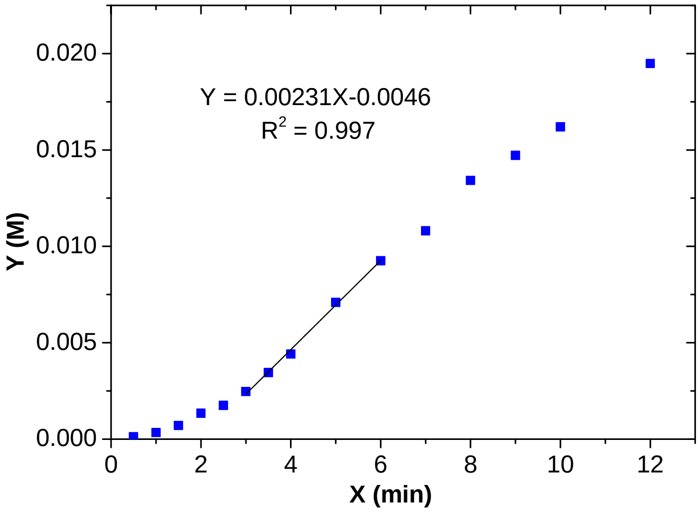

Supplementary Figure 6. Plot of the initial rate versus time with 1.5 mol% of **4b**

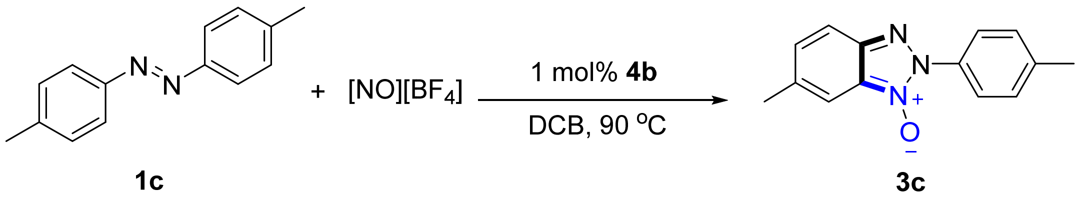

Following the above mentioned procedure: the reaction was carried out with **4b** (1 mol%, 0.0211

g), **1c** (2 mmol, 0.4204 g), [NO][BF_4] (6 mmol, 0.7008 g) and internal stardard 3,4-dichlorotoluene (2 mmol, 0.3221 g) in DCB (15 mL). The flask was sealed and removed out of the glove box. The reaction mixture was stirred at room temperature for 5 min and then 90 °C.

Supplementary Table 7. Initial rate with 1 mol% of **4b**

Time (min)	0.5	1	1.5	2	2.5	3	3.5	4
**3b** (M)	0.000338	0.000514	0.000735	0.001038	0.00136	0.001912	0.002726	0.003626
Time (min)	5	6	7	8	9	10	12	14
**3b** (M)	0.006059	0.007707	0.009044	0.010985	0.012699	0.014104	0.016357	0.018258

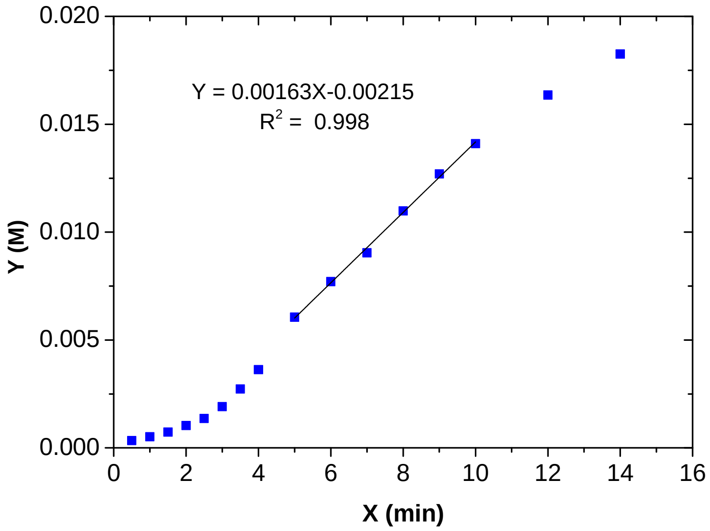

Supplementary Figure 7. Plot of the initial rate versus time with 1 mol% of **4b**

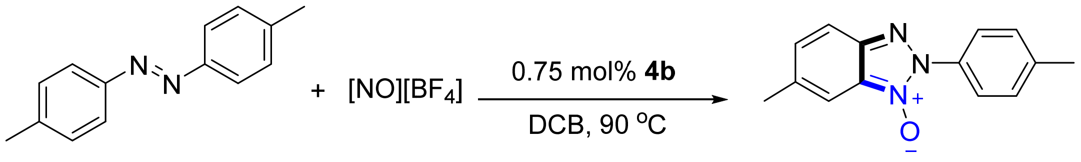

Following the above mentioned procedure: the reaction was carried out with **4b** (0.75 mol%, 0.0158 g), **1c** (2 mmol, 0.4204 g), [NO][BF_4] (6 mmol, 0.7008 g) and internal stardard 3,4-dichlorotoluene (2 mmol, 0.3221 g) in DCB (15 mL). The flask was sealed and removed out of the glove box. The reaction mixture was stirred at room temperature for 5 min and then 90 °C.

Supplementary Table 8. Initial rate with 0.75 mol% of **4b**

Time (min)	0.5	1	1.5	2	2.5	3	3.5	4
**3b** (M)	0.000986	0.001027	0.001164	0.001351	0.001623	0.001851	0.002082	0.002423
Time (min)	5	6	7	8	9	10	12	14
**3b** (M)	0.003107	0.003965	0.005444	0.006571	0.007553	0.008467	0.009992	0.011466
Time (min)	16							
**3b** (M)	0.012386							

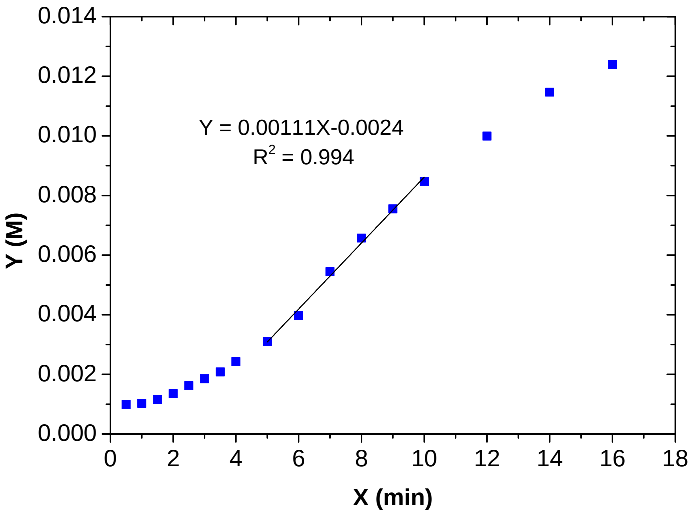

Supplementary Figure 8. Plot of the initial rate versus time with 0.75 mol% of **4b**

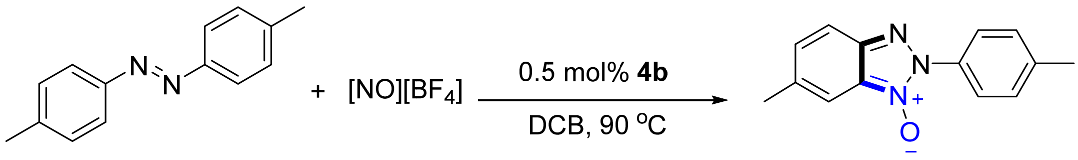

1c **3c**

Following the above mentioned procedure: the reaction was carried out with **4b** (0.5 mol%, 0.0105 g), **1c** (2 mmol, 0.4204 g), $[NO][BF_4]$ (6 mmol, 0.7008 g) and internal stardard 3,4-dichlorotoluene (2 mmol, 0.3221 g) in DCB (15 mL). The flask was sealed and removed out of the glove box. The reaction mixture was stirred at room temperature for 5 min and then 90 °C.

Supplementary Table 9. Initial rate with 0.5 mol% of **4b**

Time (min)	0.5	1	1.5	2	2.5	3	3.5	4
**3b** (M)	0.000531	0.000761	0.001012	0.001238	0.001459	0.001857	0.002015	0.002343
Time (min)	5	6	7	8	9	10	12	14
**3b** (M)	0.003025	0.003725	0.004457	0.005097	0.005949	0.006632	0.007256	0.007948
Time (min)	16	18	20					
**3b** (M)	0.008744	0.00927	0.010122					

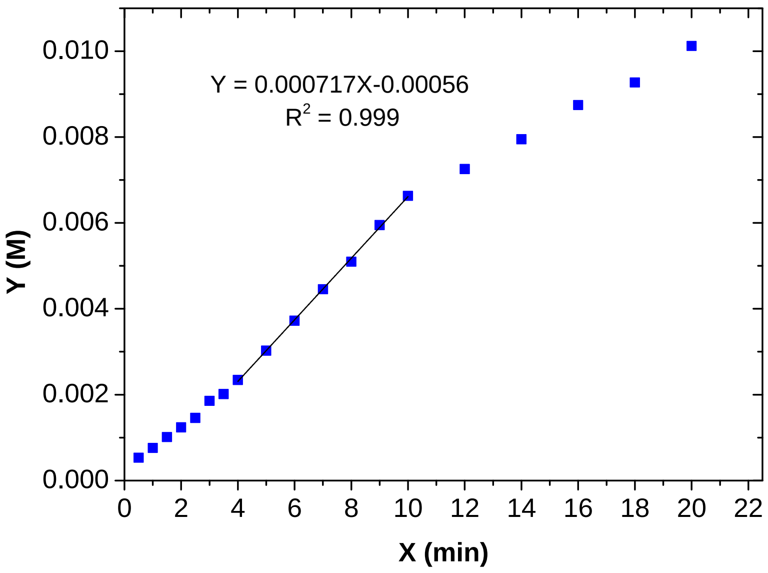

Supplementary Figure 9. Plot of the initial rate versus time with 0.5 mol% of **4b**

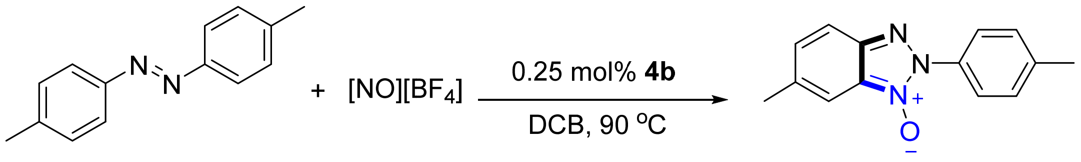

1c **3c**

Following the above mentioned procedure: the reaction was carried out with **4b** (0.25 mol%, 0.0053 g), **1c** (2 mmol, 0.4204 g), [NO][BF_4] (6 mmol, 0.7008 g) and internal stardard 3,4-dichlorotoluene (2 mmol, 0.3221 g) in DCB (15 mL). The flask was sealed and removed out of the glove box. The reaction mixture was stirred at room temperature for 5 min and then 90 °C.

Supplementary Table 10. Initial rate with 0.25 mol% of **4b**

Time (min)	0.5	1	1.5	2	2.5	3	3.5	4
**3b** (M)	0.000258	0.000352	0.000494	0.000555	0.000656	0.000767	0.000881	0.000974
Time (min)	5	6	7	8	9	10	12	14
**3b** (M)	0.00116	0.001337	0.001481	0.001641	0.001756	0.001879	0.002088	0.002222
Time (min)	16	18	20					
**3b** (M)	0.002353	0.002526	0.002618					

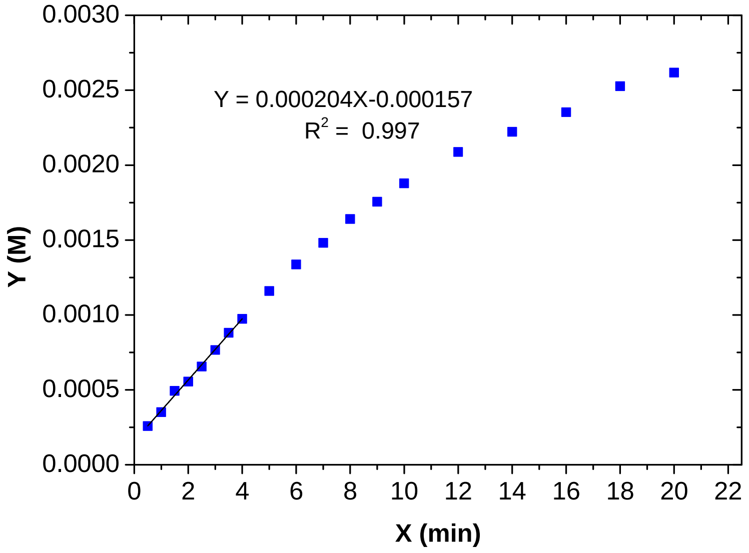

Supplementary Figure 10. Plot of the initial rate versus time with 0.25 mol% of **4b**

A plot of initial rate (Δ[**3b**]/Δt) versus [**4b**] proved to be linear indicating first order dependence on **4b** (Figure S11). This observation would support that **4b** could remain intact during the couse of the reaction.

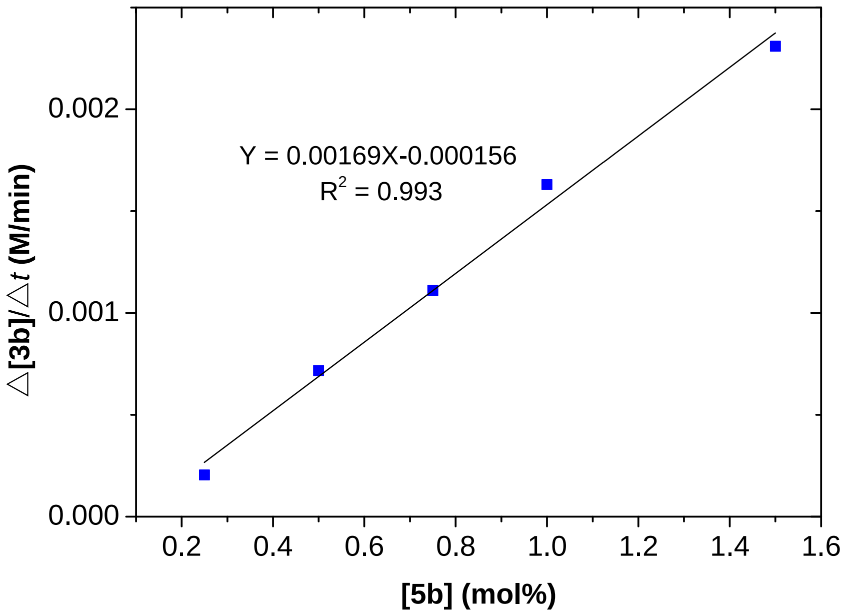

Supplementary Figure 11. Plot of Initial rate (Δ[**3b**]/Δt) versus [**4b**]

KIE Experiments

Intramolecular KIE experiments: In a glove box, a 25 mL of the Schlenk tube equipped with a stir bar was charged with $Pd(OAc)_2$ (1 mol%, 0.0005 g), **1a-*D*$_5$** (0.2 mmol, 0.00374 g), [NO][BF_4] (0.4 mmol, 0.0468 g), TBAI (1 mol%, 0.0007 g), and DCB (1.5 mL). The tube was sealed and removed out of the glove box. The reaction mixture was stirred at 80 °C for 2.5 h. Upon completion, the reaction mixture was diluted with 10 mL of ethyl acetate, filtered through a pad of silica gel, followed by washing the pad of the silica gel with the ethyl acetate (20 mL). The filtrate was concentrated under reduced pressure. The residue was then purified by chromatography on silica gel to give **1a-*D*$_5$** and the corresponding products **3a-*D*$_5$** and **3a-*D*$_4$** (10.1 mg, 23%, **3a-*D*$_5$**:**3a-*D*$_4$** = 5.4:1 (Figure S12))**.** The residue **1a-*D*$_5$** was subject to ^{1}H NMR and *H-D* exchange was not observed. This phenomenon indicates that C-H activation might be irreversible (Figure S13).

(a) Intramolecular KIE experiment

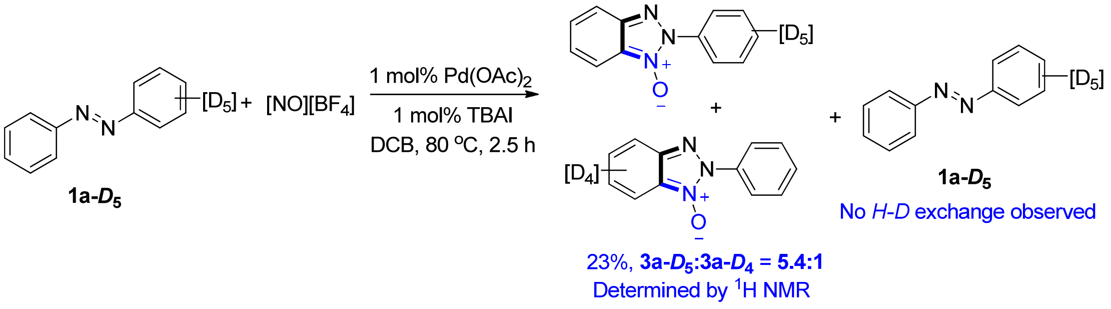

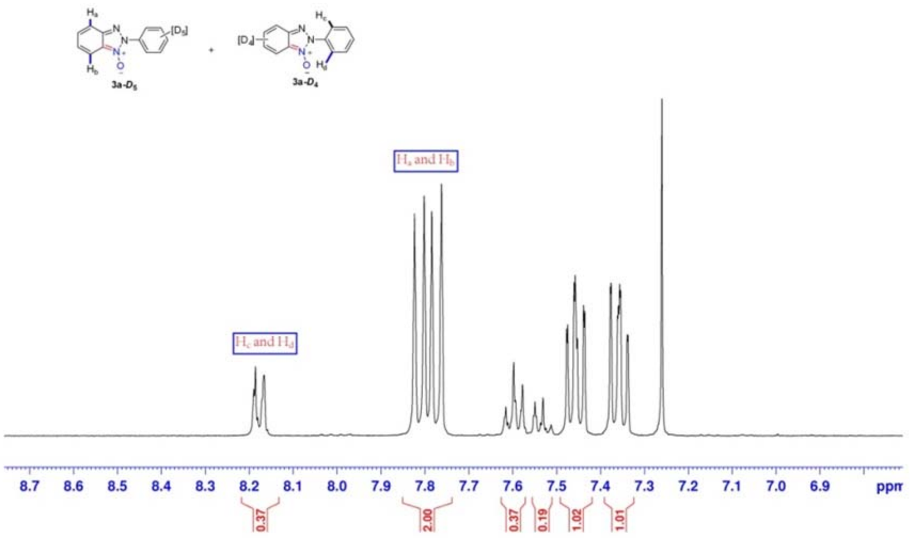

Supplementary Figure 12

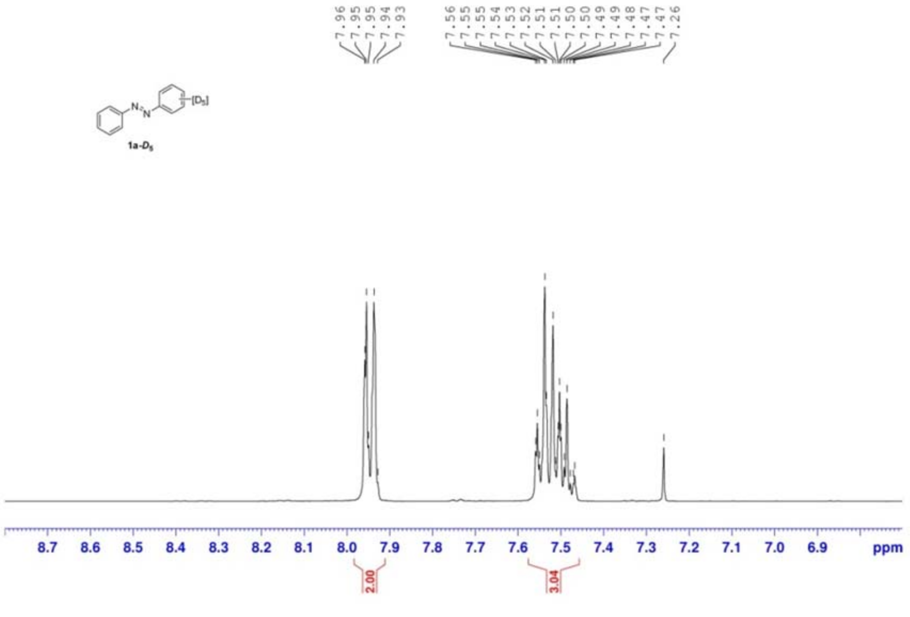

Supplementary Figure 13

Intermolecular KIE experiments: In a glove box, a 25 mL of the Schlenk tube equipped with a stir bar was charged with $Pd(OAc)_2$ (1 mol%, 0.0005 g), TBAI (1 mol%, 0.0007 g), **1a** (0.2 mmol, 0.0364 g) or **1a-D$_{10}$** (0.2 mmol, 0.0384 g), [NO][BF_4] (0.4 mmol, 0.0468 g) and DCB (1.5 mL). The tube was sealed and removed out of the glove box. The reaction mixture was stirred at 80 °C for indicated time. Upon completion, the reaction mixture was diluted with 10 mL of CH_3CN, filtered through a pad of silica gel, followed by washing the pad of the silica gel with the CH_3CN

(20 mL). The filtrate was diluted with CH_3CN to 100 mL. The concentrations of **3b** were determined by HPLC analysis.

(b) Intermolecular KIE experiment from two parallel reactions

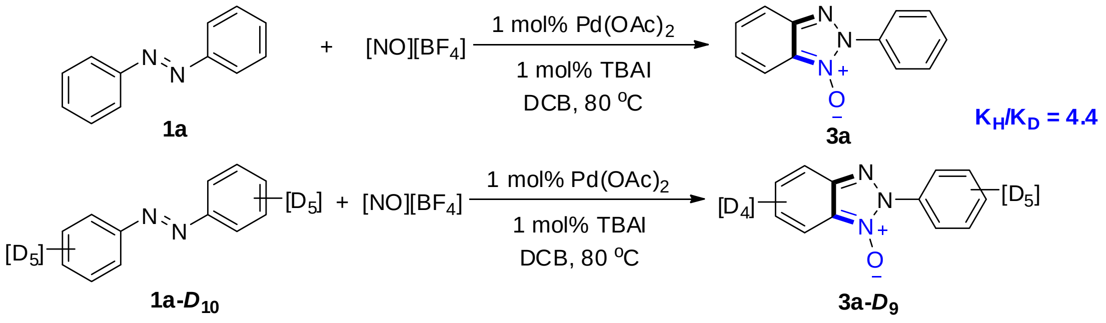

Supplementary Table 11

Time (min)	10	20	30	40	50	60	70	80
**3a** (M)	0.001984	0.007089	0.013317	0.020256	0.024633	0.032971	0.038553	0.044021
**3a-*D*$_9$** (M)	0.000403	0.001767	0.003437	0.004491	0.006301	0.007577	0.008508	0.010135

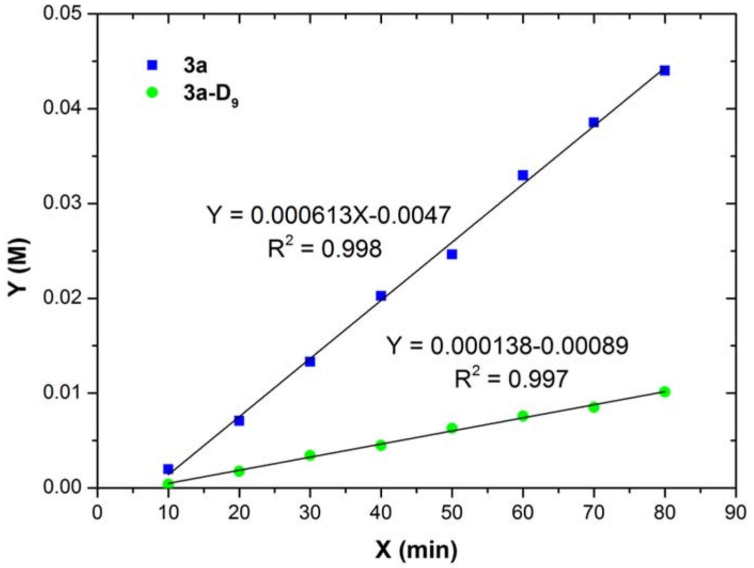

Supplementary Figure 14. Plot of Initial rates of **1a** and **1a-D$_{10}$** versus time

KIE = 0.000613/0.000138 = 4.4

Intramolecular KIE and intermolecular KIE experiments, of values 5.4 and 4.4 respectively, suggest that the cleavage of C-H bond is the rate-determination step.

Preparation of azobene starting materials

Synthesis of symmetric azobenzenes[1]

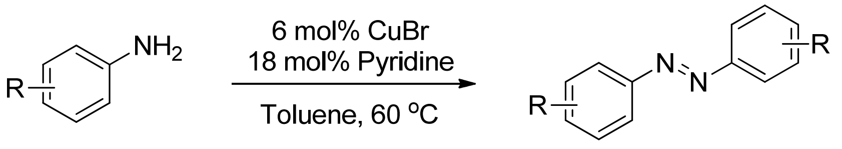

A 100 mL round flask was equipped with a stir bar was charged with CuBr (6 mol%), pyridine (18 mol%), aniline (5 mmol) in toluene (20 mL). The reaction mixture was vigorously stirred at 60 °C for 20 h under air or O_2 (1 atm). After cooling down to room temperature and concentrating in vacuum, the residue was purified by flash chromatography on silica gel.

Synthesis of unsymmetric azobenzenes[2-4]

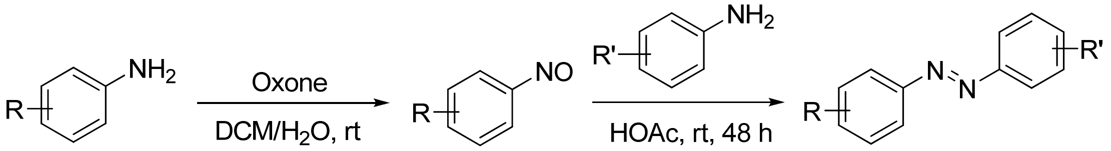

Substituted aniline (10 mmol) was dissolved in 30 mL of DCM. To this solution Oxone (15 mmol) dissolved in 30 ml of water was added. The solution was stirred under nitrogen at room temperature until TLC monitoring indicated complete consumption of the starting material. After separation of the layers, the aqueous layer was extracted with DCM twice. The combined organic layers were washed with 1N HCl twice, saturated sodium bicarbonate solution, water, brine and dried (sodium sulfate), filtered. The filtrate was concentrated under reduced pressure and the crude products were used without further purifications. To a round bottom flask equipped with a magnetic stir bar was combined the indicated aniline (6-10 mmol, 1.2-2 equiv) and the indicated nitrosobenzene (5 mmol) in glacial acetic acid (30 mL). The reaction solution was stirred at room temperature for 48 h. Upon completion, the reaction mixture was concentrated under reduced pressure. The residue was then purified by chromatography on silica gel.

1a-1l, **1n-1q**, **1t**, **1ab**, **1ad-1ae** are known compounds, and the analytical data are consistent with previously reported data.[1-2, 5-11]

Analytical data for new azobenzenes

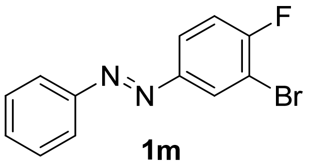

1m

(E)-1-(3-bromo-4-fluorophenyl)-2-phenyldiazene (1m) : ^{1}H NMR (400 MHz, CDCl$_3$) δ: 8.18 (d, *J* = 6.1 Hz, 1H), 7.93 (d, *J* = 6.7 Hz, 3H), 7.55-7.54 (m, 3H), 7.29 (t, *J* = 8.2 Hz, 1H). **^{13}C NMR (100 MHz, CDCl$_3$) δ:** 160.4 (d, *J* = 252.8 Hz), 152.2, 149.4 (d, *J* = 3.4 Hz), 131.5, 129.1, 126.9, 124.9 (d, *J* = 7.9 Hz), 123.0, 116.7 (d, *J* = 23.7 Hz), 110.0 (d, *J* = 22.6 Hz). **^{19}F NMR (376 MHz, CDCl$_3$) δ:** -103.8. **HRMS (ESI):** calcd for $C_{12}H_8N_2BrF$ $[M+H]^+$: 278.9928, found 278.9927.

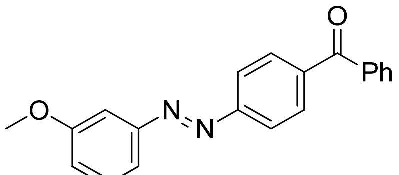

1s

(E)-(4-((3-methoxyphenyl)diazenyl)phenyl)(phenyl)methanone (1s) : ^{1}H NMR (400 MHz, $CDCl_3$) δ: 7.98 (q, *J* = 8.3 Hz, 4H), 7.84 (d, *J* = 7.6 Hz, 2H), 7.61 (d, *J* = 7.3 Hz, 2H), 7.48 (m, 4H), 7.09 (d, *J* = 8.2 Hz, 1H), 3.91 (s, 3H). **^{13}C NMR (100 MHz, $CDCl_3$) δ:** 195.96, 160.36, 154.56, 153.79, 139.16, 137.39, 132.64, 131.00, 130.01, 129.85, 128.37, 122.59, 118.53, 117.62, 105.81, 55.48. **HRMS (ESI):** calcd for $C_{20}H_{16}O_2N_2$ $[M+H]^+$: 317.1285, found 317.1282.

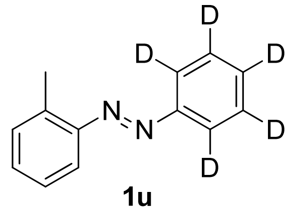

1u

(E)-1-(2,3,4,5,6-pentadeuterophenyl)-2-o-tolyldiazene (1u) : ^{1}H NMR (400 MHz, $CDCl_3$) δ: 7.67 (d, *J* = 7.9 Hz, 1H), 7.43-7.36 (m, 2H), 7.33-7.26 (m, 1H), 2.77 (s, 3H). **^{13}C NMR (100 MHz, $CDCl_3$) δ:** 152.9, 150.7, 138.0, 131.2, 130.9, 130.2 (t, *J* = 24.2 Hz), 129.9, 128.5 (t, *J* = 24.5 Hz), 126.4, 122.5 (t, *J* = 24.7 Hz) 115.4, 17.5. **HRMS (ESI):** calcd for $C_{13}H_7D_5N_2$ $[M+H]^+$: 202.1387, found 202.1386.

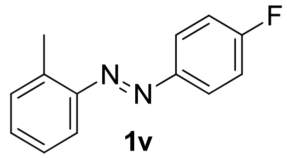

1v

(E)-1-(4-fluorophenyl)-2-o-tolyldiazene (1v) : ^{1}H NMR (400 MHz, $CDCl_3$) δ: 7.97 (dd, *J* = 8.4 Hz, 5.5 Hz, 2H), 7.65 (d, *J* = 8.0 Hz, 1H), 7.43-7.35 (m, 2H), 7.32-7.26 (m, 1H), 7.22 (t, *J* = 8.5 Hz, 2H), 2.75 (s, 3). **^{13}C NMR (100 MHz, $CDCl_3$) δ:** 164.2 (d, J = 251.6 Hz), 150.5, 149.5, 138.1, 131.1 (d, *J* = 32.4 Hz), 126.4, 124.9 (d, *J* = 8.9 Hz), 115.9 (d, *J* = 22.9 Hz), 115.4, 17.5. **^{19}F NMR (376 MHz, $CDCl_3$) δ:** -109.8. **HRMS (ESI):** calcd for $C_{13}H_{11}N_2F$ $[M+H]^+$: 215.0979, found 215.0978.

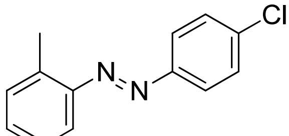

1w

(E)-1-(4-chlorophenyl)-2-o-tolyldiazene (1w) : ^{1}H NMR (400 MHz, $CDCl_3$) δ: 7.89 (d, *J* = 8.6 Hz, 2H), 7.66 (d, *J* = 8.0 Hz, 1H), 7.51 (d, *J* = 8.6 Hz, 2H), 7.44-7.34 (m, 2H), 7.33-7.25 (m, 1H), 2.75 (s, 3H). **^{13}C NMR (100 MHz, $CDCl_3$) δ:** 151.3, 150.5, 138.3, 136.6, 131.3, 131.2, 129.3, 126.4, 124.2, 115.4, 17.5. **HRMS (ESI):** calcd for $C_{13}H_{11}N_2Cl$ $[M+H]^+$: 231.0684, found 231.0682.

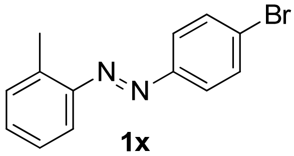

1x

(E)-1-(4-bromophenyl)-2-o-tolyldiazene (1x) : ^{1}H NMR (400 MHz, $CDCl_3$) δ: 7.82 (d, *J* = 8.2 Hz, 2H), 7.70-7.62 (m, 3H), 7.44-7.35 (m, 2H), 7.33-7.24 (m, 1H), 2.75 (s, 3H). **^{13}C NMR (100 MHz, $CDCl_3$) δ:** 151.7, 150.5, 138.4, 132.3, 131.3, 131.3, 126.45, 125.1, 124.4, 115.4, 17.5. **HRMS (ESI):** calcd for $C_{13}H_{11}N_2Br$ $[M+H]^+$: 275.0178, found 275.0177.

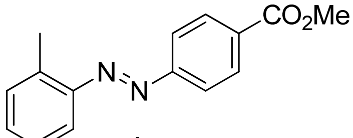

1y

(E)-methyl-4-(o-tolyldiazenyl)benzoate (1y) : ^{1}H NMR (400 MHz, $CDCl_3$) δ: 8.21 (d, *J* = 8.3 Hz, 2H), 7.97 (d, *J* = 8.3 Hz, 2H), 7.68 (d, *J* = 8.0 Hz, 1H), 7.44-7.35 (m, 2H), 7.33-7.27 (m, 1H), 3.98 (s, 3H), 2.77 (s, 3H). **^{13}C NMR (100 MHz, $CDCl_3$) δ:** 166.5, 155.4, 150.6, 138.8, 131.6, 131.5, 131.3, 130.5, 126.4, 122.7, 115.3, 52.2, 17.5. **HRMS (ESI):** calcd for $C_{15}H_{14}N_2O_2$ $[M+H]^+$: 255.1128, found 255.1128.

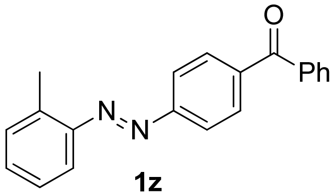

1z

(E)-phenyl(4-(o-tolyldiazenyl)phenyl)methanone (1z) : ^{1}H NMR (400 MHz, $CDCl_3$) δ: 7.98 (q, *J* = 8.4 Hz, 4H), 7.85 (d, *J* = 7.6 Hz, 2H), 7.68 (d, *J* = 8.0 Hz, 1H), 7.62 (t, *J* = 7.3 Hz, 1H), 7.52 (t, *J* = 7.5 Hz, 2H), 7.45-7.34 (m, 2H), 7.31-7.27 (m, 1H), 2.76 (s, 3H). **^{13}C NMR (100 MHz, $CDCl_3$) δ:** 196.0, 154.9, 150.7, 138.9, 138.9, 137.5, 132.6, 131.7, 131.4, 131.0, 130.0, 128.4, 126.5, 122.7, 115.4, 17.5. **HRMS (ESI):** calcd for $C_{20}H_{16}N_2O$ $[M+H]^+$: 301.1335, found 301.1334.

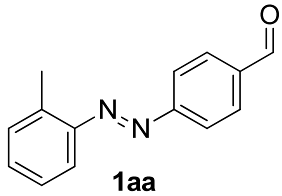

1aa

(E)-4-(o-tolyldiazenyl)benzaldehyde (1aa) : ^{1}H NMR (400 MHz, $CDCl_3$) δ: 10.11 (s, 1H), 8.03 (s, 4H), 7.68 (d, *J* = 8.0 Hz, 1H), 7.40 (dt, *J* = 14.7 Hz, 7.3 Hz, 2H), 7.31-7.26 (m, 1H), 2.76 (s, 3H). **^{13}C NMR (100 MHz, $CDCl_3$) δ:** 191.6, 156.2, 150.6, 139.2, 137.3, 132.0, 131.5, 130.7, 126.5, 123.4, 115.4, 17.5. **HRMS (ESI):** calcd for $C_{14}H_{12}N_2O$ $[M+H]^+$: 225.1022, found 225.1022.

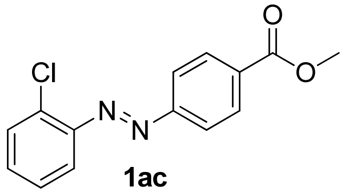

1ac

(E)-methyl-4-((2-chlorophenyl)diazenyl)benzoate (1ac) : ^{1}H NMR (400 MHz, $CDCl_3$) δ: 8.20

(d, J = 8.5 Hz, 2H), 8.00 (d, J = 8.5 Hz, 2H), 7.72 (dd, J = 8.0 Hz, 1.5, 1H), 7.61-7.57 (m, 1H), 7.43 (td, J = 7.6 Hz, 1.6 Hz, 1H), 7.36 (dd, J = 11.1 Hz, 4.1 Hz, 1H), 3.96 (s, 3H). **^{13}C NMR (100 MHz, CDCl$_3$) δ:** 166.3, 155.2, 148.4, 135.9, 132.4, 132.3, 130.8, 130.6, 127.3, 123.1, 117.5, 52.3. **HRMS (ESI):** calcd for $C_{14}H_{11}N_2O_2Cl$ $[M+H]^+$: 275.0582, found 275.0580.

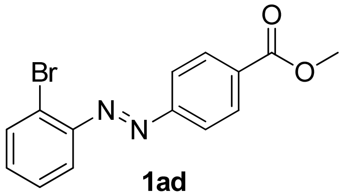

1ad

(E)-methyl-4-((2-chlorophenyl)diazenyl)benzoate (1ad) : ^{1}H NMR (400 MHz, CDCl$_3$) δ: 8.20 (d, J = 8.6 Hz, 2H), 8.01 (d, J = 8.6 Hz, 2H), 7.78 (dd, J = 7.8 Hz, 1.2 Hz, 1H), 7.70 (dd, J = 7.9 Hz, 1.7 Hz, 1H), 7.42-7.33 (m, 2H), 3.96 (s, 3H). **^{13}C NMR (100 MHz, CDCl$_3$) δ:** 166.3, 155.0, 149.5, 133.9, 132.6, 132.3, 130.7, 128.0, 123.2, 117.7, 52.3. **HRMS (ESI):** calcd for $C_{14}H_{11}N_2O_2Br$ $[M+H]^+$: 319.0077, found 319.0075.

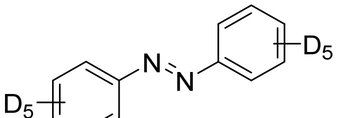

1a-D10

(E)-Dipentadeuteroazobenzene (1a-D$_{10}$): ^{13}C NMR (100 MHz, CDCl$_3$) δ: 152.5, 130.4 (t, J = 24.1 Hz), 128.5 (t, J = 24.5 Hz), 122.4 (t, J = 24.6 Hz). **HRMS (ESI):** calcd for $C_{12}D_{10}N_2$ $[M+H]^+$: 193.1544, found 193.1544.

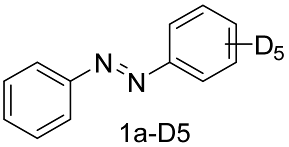

1a-D5

2,3,4,5,6-pentadeuteroazobenzene (1a-D$_5$): ^{1}H NMR (400 MHz, CDCl$_3$) δ: 7.97-7.92 (m, 2H), 7.57-7.46 (m, 3H). **^{13}C NMR (100 MHz, CDCl$_3$) δ:** 152.6, 152.5, 130.9, 130.4 (t, J = 25.0 Hz), 129.0, 128.5 (t, J = 24.4 Hz), 122.8, 122.4 (t, J = 24.6 Hz). **HRMS (ESI):** calcd for $C_{12}H_5D_5N_2$ $[M+H]^+$: 188.1231, found 188.1230.

General procedure for the reaction of azobenzene with [NO][BF$_4$] with Pd(OAc)$_2$ and TABI: In a glove box, a 25 mL of the Schlenk tube equipped with a stir bar was charged with Pd(OAc)$_2$ (3 mol%, 0.0014 g), TBAI (3 mol%, 0.0022 g), p-toluenesulfonic acid (6 mol%, 0.0021 g), Azobenzene (0.2 mmol), [NO][BF$_4$] (0.6 mmol, 0.0701 g) and DCB (1.5 mL). The tube was sealed and removed out of the glove box. The reaction mixture was stirred at 90 °C for 48 h. Upon completion, the reaction mixture was diluted with 10 mL of ethyl acetate, filtered through a pad of silica gel, followed by washing the pad of the silica gel with the ethyl acetate (20 mL). The filtrate was concentrated under reduced pressure. The residue was then purified by chromatography on silica gel to provide the corresponding product.

Analytical data for products

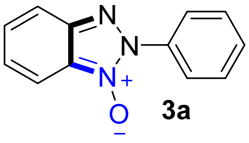

3a

2-Phenyl-2*H*-benzo[*d*][1,2,3]triazole 1-oxide (3a): The title compound was prepared according to the general procedure. The reaction was carried out with $Pd(OAc)_2$ (1 mol%, 0.0005 g), TBAI (1 mol%, 0.0007 g), **1a** (0.2 mmol, 0.0364 g), [NO][BF_4] (0.4 mmol, 0.0468 g) in DCB (1.5 mL) at 80 °C for 24 h. **3a** was obtained as white solid (38.3 mg, 90%; eluent: 10%-30% ether/hexane). **^{1}H NMR (400 MHz, $CDCl_3$) δ:** 8.17 (d, *J* = 7.8 Hz, 2H), 7.80 (d, *J* = 8.7 Hz, 1H), 7.76 (d, *J* = 8.7 Hz, 1H), 7.60-7.57 (m, 2H), 7.54-7.50 (m, 1H), 7.44 (t, *J* = 7.8 Hz, 1H), 7.34 (t, *J* = 7.8 Hz, 1H). **^{13}C NMR (100 MHz, $CDCl_3$) δ:** 141.1, 135.3, 129.9, 129.2, 129.1, 126.5, 126.3, 123.5, 119.1, 114.0. **HRMS (ESI):** calcd for $C_{12}H_9N_3O$ $[M+H]^+$: 212.0818, found 212.0818.

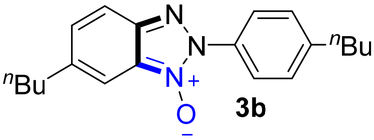

3b

6-Butyl-2-(4-butylphenyl)-2H-benzo[d][1,2,3]triazole 1-oxide (3b): The title compound was prepared according to the general procedure. The reaction was carried out with $Pd(OAc)_2$ (3 mol%, 0.0014 g), TBAI (3 mol%, 0.0022 g), TsOH (6 mol%, 0.0021 g), **1b** (0.2 mmol, 0.0589 g), [NO][BF_4] (3 equiv, 0.0701 g) in DCB (1.5 mL) at 90 °C for 48 h. **3b** was obtained as yellow oil (34.4 mg, 53%; eluent: 5-10% ether/hexane). **^{1}H NMR (400 MHz, $CDCl_3$) δ:** 8.02 (d, *J* = 8.4 Hz, 2H), 7.65 (d, *J* = 8.9 Hz, 2H), 7.54 (s, 1H), 7.37 (d, *J* = 8.4 Hz, 2H), 7.28 (dd, *J* = 8.9, 1.0 Hz, 2H), 2.77-2.64 (m, 4H), 1.71-1.57 (m, 4H), 1.43-1.30 (m, 4H), 0.97-0.89 (m, 6H). **^{13}C NMR (100 MHz, $CDCl_3$) δ:** 145.0, 141.9, 140.1, 133.0, 131.4, 129.1, 126.6, 123.4, 118.5, 111.3, 35.7, 35.3, 33.3, 32.8, 22.2, 22.1, 13.8. **HRMS (ESI):** calcd for $C_{20}H_{26}N_3O$ $[M+H]^+$: 324.2070, found 324.2072.

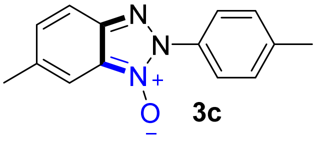

3c

6-Methyl-2-p-tolyl-2*H*-benzo[*d*][1,2,3]triazole 1-oxide (3c): The title compound was prepared according to the general procedure. The reaction was carried out with $Pd(OAc)_2$ (3 mol%, 0.0014 g), TBAI (3 mol%, 0.0022 g), TsOH (6 mol%, 0.0021 g), **1c** (0.2 mmol, 0.0421 g), [NO][BF_4] (3 equiv, 0.0701 g) in DCB (1.5 mL) at 90 °C for 48 h. **3c** was obtained as pale yellow solid (35.5 mg, 74%; eluent: 10%-30% ether/hexane). **^{1}H NMR (400 MHz, $CDCl_3$) δ:** 8.03 (d, *J* = 7.9 Hz, 2H), 7.65 (d, *J* = 8.8 Hz, 1H), 7.55 (s, 1H), 7.38 (d, *J* = 7.8 Hz, 2H), 7.27 (d, *J* = 8.5 Hz, 1H), 2.50 (s, 3H), 2.46 (s, 3H). **^{13}C NMR (100 MHz, $CDCl_3$) δ:** 140.1, 139.9, 137.1, 132.88, 132.0, 129.7, 126.7, 123.4, 118.5, 111.9, 21.9, 21.30. **HRMS (ESI):** calcd for $C_{14}H_{13}N_3O$ $[M+H]^+$: 240.1131, found 240.1131.

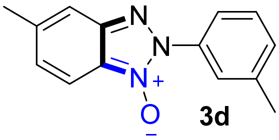

3d

5-Methyl-2-*m*-tolyl-2*H*-benzo[*d*][1,2,3]triazole 1-oxide (3d): The title compound was prepared

according to the general procedure. The reaction was carried out with $Pd(OAc)_2$ (3 mol%, 0.0014 g), TBAI (3 mol%, 0.0022 g), TsOH (6 mol%, 0.0021 g), **1d** (0.2 mmol, 0.0421 g), $[NO][BF_4]$ (3 equiv, 0.0701 g) in DCB (1.5 mL) at 90 °C for 48 h. **3d** was obtained as pale yellow solid (36.2 mg, 76%; eluent: 10%-30% ether/hexane). **^{1}H NMR (400 MHz, $CDCl_3$) δ:** 7.94 (d, *J* = 7.1 Hz, 2H), 7.67 (d, *J* = 8.9 Hz, 1H), 7.47 (s, 1H), 7.43 (d, *J* = 7.9 Hz, 1H), 7.31 (d, *J* = 7.6 Hz, 1H), 7.16 (d, *J* = 8.9 Hz, 1H), 2.47 (s, 3H), 2.46 (s, 3H). **^{13}C NMR (100 MHz, $CDCl_3$) δ:** 141.5, 139.5, 139.3, 135.2, 130.5, 129.3, 128.92, 125.3, 124.0, 120.7, 117.1, 113.4, 22.1, 21.4. **HRMS (ESI):** calcd for $C_{14}H_{13}N_3O$ $[M+H]^+$: 240.1131, found 240.1130.

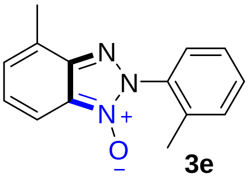

3e

4-Methyl-2-(o-tolyl)-2*H*-benzo[*d*][1,2,3]triazole 1-oxide (3e): The title compound was prepared according to the general procedure. The reaction was carried out with $Pd(OAc)_2$ (3 mol%, 0.0014 g), TBAI (3 mol%, 0.0022 g), TsOH (6 mol%, 0.0021 g), **1e** (0.2 mmol, 0.0421 g), $[NO][BF_4]$ (3 equiv, 0.0701 g) in DCB (1.5 mL) at 90 °C for 48 h. **3e** was obtained as pale yellow oil (10.2 mg, 21%; eluent: 10%-30% ether/hexane). **^{1}H NMR (400 MHz, $CDCl_3$) δ:** 7.66 (d, *J* = 8.5 Hz, 1H), 7.54-7.39 (m, 5H), 7.30-7.22 (m, 3H), 2.65 (s, 3H), 2.22 (s, 3H). **^{13}C NMR (100 MHz, $CDCl_3$) δ:** 142.0, 137.0, 133.8, 131.5, 131.3, 129.8, 127.9, 126.9, 126.5, 125.3, 111.4, 17.8, 15.9. **HRMS (ESI):** calcd for $C_{14}H_{13}N_3O$ $[M+H]^+$: 240.1131, found 240.1130.

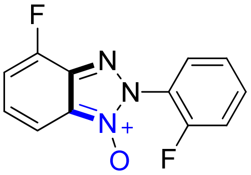

3f

4-Fluoro-2-(2-fluorophenyl)-2*H*-benzo[*d*][1,2,3]triazole 1-oxide (3f): The title compound was prepared according to the general procedure. The reaction was carried out with $Pd(OAc)_2$ (3 mol%, 0.0014 g), TBAI (3 mol%, 0.0022 g), TsOH (6 mol%, 0.0021 g), **1f** (0.2 mmol, 0.0436 g), $[NO][BF_4]$ (3 equiv, 0.0701 g) in DCB (1.5 mL) at 90 °C for 48 h. **3f** was obtained as pale yellow solid (8.5 mg, 17%; eluent: 1:1:9 ether/CH_2Cl_2/hexane). **^{1}H NMR (400 MHz, $CDCl_3$) δ:** 7.71-7.58 (m, 3H), 7.41-7.35 (m, 2H), 7.32-7.25 (m, 1H), 7.16-7.10 (m, 1H). **^{13}C NMR (100 MHz, $CDCl_3$) δ:** 156.2 (d, *J* = 228.5 Hz), 153.6 (d, *J* = 229.9 Hz), 133.9 (d, *J* = 21.4 Hz), 133.6 (d, *J* = 8.0 Hz), 128.9, 127.3 (d, *J* = 5.8 Hz), 126.2 (d, *J* = 6.1 Hz), 124.9 (d, *J* = 3.9 Hz), 122.0 (d, *J* = 12.4 Hz), 117.2 (d, *J* = 18.8 Hz), 112.6 (d, *J* = 16.1 Hz), 110.3 (d, *J* = 5.6 Hz). **^{19}F NMR (376 MHz, $CDCl_3$) δ:** -116.67 (s, 1F), -127.10 (s, 1F). **HRMS (ESI):** calcd for $C_{12}H_8N_3OF_2$ $[M+H]^+$: 248.0630, found 248.0629.

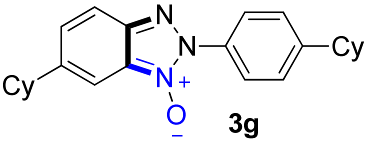

3g

6-Cyclohexyl-2-(4-cyclohexylphenyl)-2*H*-benzo[*d*][1,2,3]triazole 1-oxide (3g): The title compound was prepared according to the general procedure. The reaction was carried out with

Pd(OAc)$_2$ (3 mol%, 0.0014 g), TBAI (3 mol%, 0.0022 g), TsOH (6 mol%, 0.0021 g), **1g** (0.2 mmol, 0.0693 g), [NO][BF$_4$] (3 equiv, 0.0701 g) in DCB (1.5 mL) at 90 °C for 48 h. **3g** was obtained as pale yellow solid (54.4 mg, 72%; eluent: 1:1:9 ether/CH$_2$Cl$_2$/hexane). **^{1}H NMR (400 MHz, CDCl$_3$) δ:** 8.01 (d, *J* = 8.2 Hz, 2H), 7.65 (d, *J* = 9.0 Hz, 1H), 7.56 (s, 1H), 7.40 (d, *J* = 8.2 Hz, 2H), 7.33 (d, *J* = 9.0 Hz, 1H), 2.62-2.59 (m, 2H), 1.96-1.75 (m, 10H), 1.50-1.25 (m, 10H). **^{13}C NMR (100 MHz, CDCl$_3$) δ:** 150.0, 147.1, 140.2, 133.0, 130.5, 127.5, 126.7, 123.5, 118.5, 109.6, 44.6, 44.4, 34.2, 33.9, 26.7, 26.6, 26.0, 26.0. **HRMS (ESI):** calcd for C$_{24}$H$_{29}$N$_3$O [M+H]$^+$: 376.2383, found 376.2382.

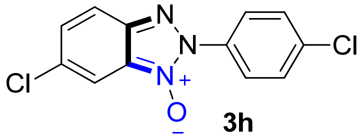

3h

6-Chloro-2-(4-chlorophenyl)-2*H*-benzo[*d*][1,2,3]triazole 1-oxide (3h): The title compound was prepared according to the general procedure. The reaction was carried out with Pd(OAc)$_2$ (3 mol%, 0.0014 g), TBAI (3 mol%, 0.0022 g), TsOH (6 mol%, 0.0021 g), **1h** (0.2 mmol, 0.0502 g), [NO][BF$_4$] (3 equiv, 0.0701 g) in DCB (1.5 mL) at 90 °C for 48 h. **3h** was obtained as white solid (38.6 mg, 69%; eluent: 1:1:9 ether/CH$_2$Cl$_2$/hexane). **^{1}H NMR (400 MHz, CDCl$_3$) δ:** 8.17 (d, *J* = 8.6 Hz, 2H), 7.79 (s, 1H), 7.70 (d, *J* = 9.2 Hz, 1H), 7.56 (d, *J* = 8.6 Hz, 2H), 7.39 (d, *J* = 9.1 Hz, 1H). **^{13}C NMR (100 MHz, CDCl$_3$) δ:** 139.6, 136.1, 133.6, 132.7, 131.2, 129.5, 126.6, 124.4, 120.4, 113.1. **HRMS (ESI):** calcd for C$_{12}$H$_7$N$_3$OCl$_2$ [M+H]$^+$: 280.0039, found 280.0038.

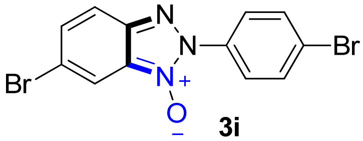

3i

5-Bromo-2-(3-bromophenyl)-2*H*-benzo[*d*][1,2,3]triazole 1-oxide (3i): The title compound was prepared according to the general procedure. The reaction was carried out with Pd(OAc)$_2$ (3 mol%, 0.0014 g), TBAI (3 mol%, 0.0022 g), TsOH (6 mol%, 0.0021 g), **1i** (0.2 mmol, 0.0680 g), [NO][BF$_4$] (3 equiv, 0.0701 g) in DCB (1.5 mL) at 100 °C for 24 h. **3i** was obtained as brown solid (57.0 mg, 77%; eluent: 1:1:9 ether/CH$_2$Cl$_2$/hexane). **^{1}H NMR (400 MHz, CDCl$_3$) δ:** 8.38 (s, 1H), 8.19 (d, *J* = 7.9 Hz, 1H), 7.95 (s, 1H), 7.67 (t, *J* = 7.4 Hz, 2H), 7.48-7.40 (m, 2H). **^{13}C NMR (100 MHz, CDCl$_3$) δ:** 141.7, 135.9, 133.1, 130.5, 130.5, 126.1, 125.3, 123.60, 122.7, 121.6, 121.4, 115.4. **HRMS (ESI):** calcd for C$_{12}$H$_7$N$_3$OBr$_2$ [M+H]$^+$: 367.9029, found 367.9027.

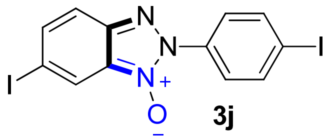

3j

6-Iodo-2-(4-iodophenyl)-2*H*-benzo[*d*][1,2,3]triazole 1-oxide (3j): The title compound was prepared according to the general procedure. The reaction was carried out with Pd(OAc)$_2$ (3 mol%, 0.0014 g), TBAI (3 mol%, 0.0022 g), TsOH (6 mol%, 0.0021 g), **1j** (0.2 mmol, 0.0868 g), [NO][BF$_4$] (3 equiv, 0.0701 g) in DCB (1.5 mL) at 90 °C for 48 h. **3j** was obtained as brown solid (82.4 mg, 89%; eluent: 1:1:9 ether/CH$_2$Cl$_2$/hexane). **^{1}H NMR (400 MHz, CDCl$_3$) δ:** 8.22 (s, 1H), 7.98-7.91 (m, 4H), 7.67 (d, *J* = 9.1 Hz, 1H), 7.51 (d, *J* = 9.1 Hz, 1H). **^{13}C NMR (100 MHz, CDCl$_3$) δ:** 140.0, 138.5, 138.4, 134.7, 127.7, 124.5, 123.0, 120.4, 96.0, 90.9. **HRMS (ESI):** calcd for C$_{12}$H$_7$N$_3$OI$_2$ [M+H]$^+$: 463.8751, found 463.8749.

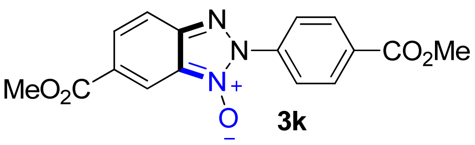

3k

6-(Methoxycarbonyl)-2-(4-(methoxycarbonyl)phenyl)-2*H*-benzo[*d*][1,2,3]triazole 1-oxide (3k): The title compound was prepared according to the general procedure. The reaction was carried out with $Pd(OAc)_2$ (3 mol%, 0.0014 g), TBAI (3 mol%, 0.0022 g), TsOH (6 mol%, 0.0021 g), **1k** (0.2 mmol, 0.0596 g), [NO][BF_4] (3 equiv, 0.0701 g) in DCB (1.5 mL) at 90 °C for 48 h. **3k** was obtained as pale yellow solid (57.9 mg, 88%; eluent: 1:2:5 ether/CH_2Cl_2/hexane). **^{1}H NMR (400 MHz, $CDCl_3$) δ:** 8.54 (s, 1H), 8.37 (d, J = 8.1 Hz, 2H), 8.24 (d, J = 7.5 Hz, 2H), 8.05 (d, J = 9.2 Hz, 1H), 7.77 (d, J = 9.1 Hz, 1H), 3.97 (s, 3H), 3.96 (s, 3H). **^{13}C NMR (100 MHz, $CDCl_3$) δ:** 165.7, 165.5, 142.4, 138.4, 131.2, 130.6, 129.4, 128.3, 126.4, 122.5, 119.2, 117.7, 52.7, 52.5. **HRMS (ESI):** calcd for $C_{16}H_{13}N_3O_5$ $[M+H]^+$: 328.0928, found 328.0926.

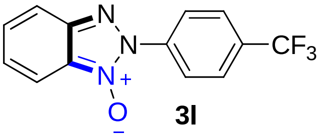

3l

2-(4-(Trifluoromethyl)phenyl)-2*H*-benzo[*d*][1,2,3]triazole 1-oxide (3l): The title compound was prepared according to the general procedure. The reaction was carried out with $Pd(OAc)_2$ (3 mol%, 0.0014 g), TBAI (3 mol%, 0.0022 g), TsOH (6 mol%, 0.0021 g), **1l** (0.2 mmol, 0.0501 g), [NO][BF_4] (3 equiv, 0.0701 g) in DCB (1.5 mL) at 90 °C for 48 h. **3l** was obtained as white solid (41.2 mg, 74%; eluent: 10%-30% ether/hexane). **^{1}H NMR (400 MHz, $CDCl_3$) δ:** 8.44 (d, J = 8.5 Hz, 2H), 7.85 (d, J = 8.6 Hz, 2H), 7.80-7.75 (m, 2H), 7.49-7.45 (m, 1H), 7.38-7.34 (m, 1H). **^{13}C NMR (100 MHz, $CDCl_3$) δ:** 141.5, 137.9, 131.4 (q, J = 32.9 Hz), 129.7, 126.9, 126.7, 126.4 (q, J = 3.7 Hz), 123.5 (q, J = 270.8 Hz), 123.2, 119.2, 114.1. **^{19}F NMR (376 MHz, $CDCl_3$) δ:** -62.8. **HRMS (ESI):** calcd for $C_{13}H_8N_3OF_3$ $[M+H]^+$: 280.0692, found 280.0691.

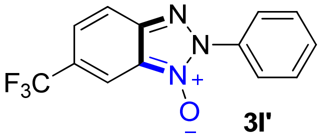

3l'

2-Phenyl-6-(trifluoromethyl)-2*H*-benzo[*d*][1,2,3]triazole 1-oxide (3l'): The title compound was prepared according to the general procedure. The reaction was carried out with $Pd(OAc)_2$ (3 mol%, 0.0014 g), TBAI (3 mol%, 0.0022 g), TsOH (6 mol%, 0.0021 g), **1l** (0.2 mmol, 0.0501 g), [NO][BF_4] (3 equiv, 0.0701 g) in DCB (1.5 mL) at 90 °C for 48 h. **3l'** was obtained as white solid (6.6 mg, 12%; eluent: 10% ether/hexane). **^{1}H NMR (400 MHz, $CDCl_3$) δ:** 8.18 (d, J = 7.7 Hz, 3H), 7.90 (d, J =9.1 Hz, 1H), 7.63-7.55 (m, 4H). **^{13}C NMR (100 MHz, $CDCl_3$) δ:** 141.4, 135.0, 130.5, 129.4, 128.3 (q, J = 33.3 Hz), 125.6, 125.3 (q, J = 2.7 Hz), 123.5, 123.5 (q, J = 270.8 Hz), 120.6, 113.3 (q, J = 5.1 Hz). **^{19}F NMR (376 MHz, $CDCl_3$) δ:** -62.8. **HRMS (ESI):** calcd for $C_{13}H_8N_3OF_3$ $[M+H]^+$: 280.0692, found 280.0691.

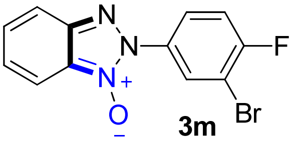

3m

2-(3-Bromo-4-fluorophenyl)-2*H*-benzo[*d*][1,2,3]triazole 1-oxide (3m): The title compound was

prepared according to the general procedure. The reaction was carried out with $Pd(OAc)_2$ (3 mol%, 0.0014 g), TBAI (3 mol%, 0.0022 g), TsOH (6 mol%, 0.0021 g), **1m** (0.2 mmol, 0.0558 g), $[NO][BF_4]$ (3 equiv, 0.0701 g) in chlorobenzene (1.5 mL) at 100 °C for 48 h. **3m** was obtained as white solid (45.0 mg, 73%; eluent: 10%-30% ether/hexane). **^{1}H NMR (400 MHz, $CDCl_3$) δ:** 8.49 (dd, J = 6.0 Hz, 2.6 Hz, 1H), 8.22 (ddd, J = 9.0 Hz, 4.1 Hz, 2.6 Hz, 1H), 7.75 (dd, J = 12.4 Hz, 8.8 Hz, 2H), 7.48-7.44 (m, 1H), 7.37-7.30 (m, 2H). **^{13}C NMR (100 MHz, $CDCl_3$) δ:** 159.4 (d, J = 252.4 Hz), 141.3, 131.9, 129.6, 128.6, 126.7, 126.5, 124.1 (d, J = 8.0 Hz), 119.1, 116.9 (d, J = 24.1 Hz), 114.0, 109.7 (d, J = 22.7 Hz). **^{19}F NMR (376 MHz, $CDCl_3$) δ:** -103.9. **HRMS (ESI):** calcd for $C_{12}H_7N_3OBrF$ $[M+H]^+$: 307.9829, found 307.9828.

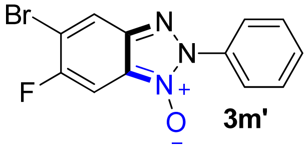

3m'

5-Bromo-6-fluoro-2-phenyl-2*H*-benzo[*d*][1,2,3]triazole 1-oxide (3m'): The title compound was prepared according to the general procedure. The reaction was carried out with $Pd(OAc)_2$ (3 mol%, 0.0014 g), TBAI (3 mol%, 0.0022 g), TsOH (6 mol%, 0.0021 g), **1m** (0.2 mmol, 0.0558 g), $[NO][BF_4]$ (3 equiv, 0.0701 g) in chlorobenzene (1.5 mL) at 100 °C for 48 h. **3m'** was obtained as white solid (7.5 mg, 12%; eluent: 10% ether/hexane). **^{1}H NMR (400 MHz, $CDCl_3$) δ:** 8.15-8.12 (m, 2H), 8.08 (d, J = 5.9 Hz, 1H), 7.63-7.55 (m, 3H), 7.53 (d, J = 6.7 Hz, 1H). **^{13}C NMR (100 MHz, $CDCl_3$) δ:** 156.7 (d, J = 252.6 Hz), 138.3, 135.0, 130.3, 129.4, 125.1 (d, J = 11.6 Hz), 124.0, 123.5, 114.8 (d, J = 27.1 Hz), 99.1 (d, J = 30.0 Hz). **^{19}F NMR (376 MHz, $CDCl_3$) δ:** -104.3. **HRMS (ESI):** calcd for $C_{12}H_7N_3OBrF$ $[M+H]^+$: 307.9829, found 307.9828.

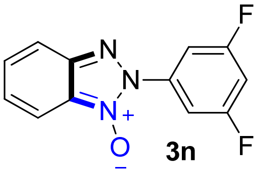

3n

2-(3,5-Difluorophenyl)-2*H*-benzo[*d*][1,2,3]triazole 1-oxide (3n): The title compound was prepared as a inseperable mixture of **3n** and **3n'** according to the general procedure. The reaction was carried out with $Pd(OAc)_2$ (3 mol%, 0.0014 g), TBAI (3 mol%, 0.0022 g), TsOH (6 mol%, 0.0021 g), **1n** (0.2 mmol, 0.0436 g), $[NO][BF_4]$ (3 equiv, 0.0701 g) in chlorobenzene (1.5 mL) at 100 °C for 48 h. The mixture of **3n** and **3n'** was obtained as white solid (32.0 mg, 65%; **3n** : **3n**' = 11.1, eluent: 10-20% ether/hexane). **^{1}H NMR (400 MHz, $CDCl_3$) δ:** 8.02-7.97 (m, 2H), 7.75 (dd, J = 12.1 Hz, 8.8 Hz, 2H), 7.49-7.45 (m, 1H), 7.36 (dd, J = 8.3 Hz, 7.2 Hz, 1H), 6.97 (tt, J = 8.6 Hz, 2.3 Hz, 1H). **^{13}C NMR (100 MHz, $CDCl_3$) δ:** 162.8 (dd, J = 249.7 Hz, 13.6 Hz), 141.4, 136.8 (t, J = 13.3 Hz), 129.9, 126.9, 126.8, 119.2, 114.1, 106.3 (dd, J = 15.7 Hz, 5.8 Hz), 105.1 (t, J = 25.3 Hz). **^{19}F NMR (376 MHz, $CDCl_3$) δ:** -106.5. **HRMS (ESI):** calcd for $C_{12}H_7N_3OF_2$ $[M+H]^+$: 248.0630, found 248.0630.

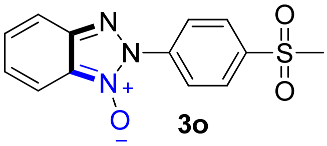

3o

2-(4-(Methylsulfonyl)phenyl)-2*H*-benzo[*d*][1,2,3]triazole 1-oxide (3o): The title compounds was prepared according to the general procedure. The reaction was carried out with $Pd(OAc)_2$ (3

mol%, 0.0014 g), TBAI (3 mol%, 0.0022 g), TsOH (6 mol%, 0.0021 g), **1o** (0.2 mmol, 0.0436 g), [NO][BF_4] (3 equiv, 0.0701 g) in nitrobenzene (1.5 mL) at 100 °C for 24 h. **3o** was obtained as white solid (38.5 mg, 67%; eluent: 10-20% ether/hexane then CH_2Cl_2). **^{1}H NMR (400 MHz, $CDCl_3$) δ:** 8.56-8.54 (m, 2H), 8.16-8.14 (m, 2H), 7.75 (t, J = 7.6 Hz, 2H), 7.49- 7.45 (m, 1H), 7.38-7.34 (m, 1H), 3.11 (s, 3H). **^{13}C NMR (100 MHz, $CDCl_3$) δ:** 141.7, 140.9, 139.20, 130.0, 128.6, 127.1, 126.7, 123.3, 119.2, 114.0, 44.5. **HRMS (ESI):** calcd for $C_{13}H_{11}N_3O_3S$ $[M+H]^+$: 290.0594, found 290.0593.

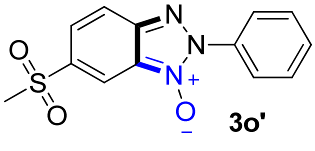

3o'

6-(Methylsulfonyl)-2-phenyl-2*H*-benzo[*d*][1,2,3]triazole 1-oxide (3o'): The title compound was prepared according to the general procedure. The reaction was carried out with Pd(OAc)$_2$ (3 mol%, 0.0014 g), TBAI (3 mol%, 0.0022 g), TsOH (6 mol%, 0.0021 g), **1o** (0.2 mmol, 0.0436 g), [NO][BF_4] (3 equiv, 0.0701 g) in nitrobenzene (1.5 mL) at 100 °C for 24 h. **3o'** was obtained as white solid (6.0 mg, 10%; eluent: 10-20% ether/hexane). **^{1}H NMR (400 MHz, $CDCl_3$) δ:** 8.57 (s, 1H), 8.19 (d, J = 7.3 Hz, 2H), 7.97-7.91 (m, 2H), 7.65- 7.58 (m, 3H), 3.14 (s, 3H). **^{13}C NMR (100 MHz, $CDCl_3$) δ:** 141.7, 138.1, 134.9, 130.7, 129.4, 126.0, 125.7, 123.4, 121.1, 117.2, 44.4. **HRMS (ESI):** calcd for $C_{13}H_{11}N_3O_3S$ $[M+H]^+$: 290.0594, found 290.0593.

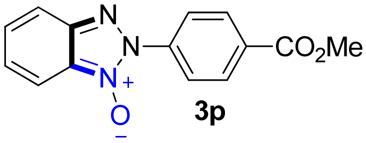

3p

2-(4-(Methoxycarbonyl)phenyl)-2*H*-benzo[*d*][1,2,3]triazole 1-oxide (3p): The title compounds were prepared according to the general procedure. The reaction was carried out with Pd(OAc)$_2$ (0.5 mol%, 0.0007 g), TBAI (0.5 mol%, 0.0011 g), **1p** (0.6 mmol, 0.1441 g), [NO][BF_4] (2 equiv, 0.1402 g) in DCB (1.5 mL) at 80 °C for 24 h. **3p** was obtained as pale yellow solid (122.9 mg, 76%; eluent: 10-30% ether/hexane then CH_2Cl_2). **^{1}H NMR (400 MHz, $CDCl_3$) δ:** 8.39 (d, J = 8.7 Hz, 2H), 8.24 (d, J = 8.7 Hz, 2H), 7.77 (dd, J = 11.6 Hz, 9.0 Hz, 2H), 7.46 (t, J = 8.2 Hz, 2H), 7.37-7.34 (m, 1H), 3.96 (s, 3H). **^{13}C NMR (100 MHz, $CDCl_3$) δ:** 165.8, 141.5, 138.7, 130.9, 130.6, 129.7, 126.74, 122.6, 119.2, 114.1, 52.4. **HRMS (ESI):** calcd for $C_{14}H_{11}N_3O_3$ $[M+H]^+$: 270.0873, found 270.0872.

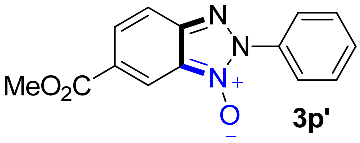

3p'

6-(Methoxycarbonyl)-2-phenyl-2*H*-benzo[*d*][1,2,3]triazole 1-oxide (3p'): The title compounds were prepared according to the general procedure. The reaction was carried out with Pd(OAc)$_2$ (0.5 mol%, 0.0007 g), TBAI (0.5 mol%, 0.0011 g), **1p** (0.6 mmol, 0.1441 g), [NO][BF_4] (2 equiv, 0.1402 g) in DCB (1.5 mL) at 80 °C for 24 h. **3p'** was obtained as white solid (17.5 mg, 11%; eluent: 10-20% ether/hexane). **^{1}H NMR (400 MHz, $CDCl_3$) δ:** 8.59 (s, 1H), 8.18 (d, J = 7.5 Hz, 2H), 8.07 (d, J = 9.1 Hz, 1H), 7.80 (d, J = 9.1 Hz, 1H), 7.62-7.55 (m, 3H), 3.98 (s, 3H). **^{13}C NMR (100 MHz, $CDCl_3$) δ:** 165.8, 142.2, 135.1, 130.3, 129.3, 129.0, 128.0, 126.3, 123.4, 119.1, 117.8, 52.6. **HRMS (ESI):** calcd for $C_{14}H_{11}N_3O_3$

[M+H]$^+$: 270.0873, found 270.0872.

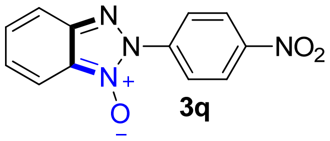

3q

2-(4-Nitrophenyl)-2*H*-benzo[*d*][1,2,3]triazole 1-oxide (3q): The title compounds were prepared according to the general procedure. The reaction was carried out with $Pd(OAc)_2$ (3 mol%, 0.0014 g), TBAI (3 mol%, 0.0022 g), TsOH (6 mol%, 0.0021 g), **1q** (0.2 mmol, 0.0454 g), [NO][BF_4] (3 equiv, 0.0701 g) in nitrobenzene (1.5 mL) at 100 °C for 24 h. **3q** was obtained as yellow solid (44.3 mg, 86%; eluent: 10-30% ether/hexane then CH_2Cl_2). **^{1}H NMR (400 MHz, $CDCl_3$) δ:** 8.61 (d, J = 9.2 Hz, 2H), 8.45 (d, J = 9.2 Hz, 2H), 7.78 (t, J = 8.7 Hz, 2H), 7.52-7.48 (m, 1H), 7.41-7.37 (m, 1H). **^{13}C NMR (100 MHz, $CDCl_3$) δ:** 147.4, 141.9, 139.9, 130.2, 127.2, 126.9, 124.7, 123.1, 119.3, 114.1. **HRMS (ESI):** calcd for $C_{12}H_8N_4O_3$ [M+H]$^+$: 257.0669, found 257.0668.

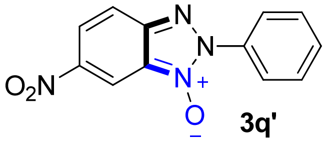

3q'

6-Nitro-2-phenyl-2*H*-benzo[*d*][1,2,3]triazole 1-oxide (3q'): The title compounds were prepared according to the general procedure. The reaction was carried out with $Pd(OAc)_2$ (3 mol%, 0.0014 g), TBAI (3 mol%, 0.0022 g), TsOH (6 mol%, 0.0021 g), **1q** (0.2 mmol, 0.0454 g), [NO][BF_4] (3 equiv, 0.0701 g) in nitrobenzene (1.5 mL) at 100 °C for 24 h. **3q'** was obtained as yellow solid (3.5 mg, 7%; eluent: 10-20% ether/hexane). **^{1}H NMR (400 MHz, $CDCl_3$) δ:** 8.85 (d, J = 2.0 Hz, 1H), 8.28 (dd, J = 9.5 Hz, 2.1 Hz, 1H), 8.21-8.19 (m, 2H), 7.91 (d, J = 9.5 Hz, 1H), 7.64-7.61 (m, 3H). **^{13}C NMR (100 MHz, $CDCl_3$) δ:** 145.4, 141.8, 134.9, 130.8, 129.5, 125.4, 123.4, 123.4, 120.5, 113.0. **HRMS (ESI):** calcd for $C_{12}H_8N_4O_3$ [M+H]$^+$: 257.0669, found 257.0668.

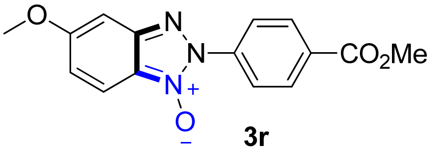

3r

5-Methoxy-2-(4-(methoxycarbonyl)phenyl)-2*H*-benzo[*d*][1,2,3]triazole 1-oxide (3r): The title compound was prepared according to the general procedure. The reaction was carried out with $Pd(OAc)_2$ (3 mol%, 0.0014 g), TBAI (3 mol%, 0.0022 g), TsOH (6 mol%, 0.0021 g), **1r** (0.2 mmol, 0.0541 g), [NO][BF_4] (3 equiv, 0.0701 g) in DCB (1.5 mL) at 90 °C for 48 h. **3r** was obtained pale yellow solid (40.6 mg, 68%; eluent: 1:2:5 ether/CH_2Cl_2/hexane). **^{1}H NMR (400 MHz, $CDCl_3$) δ:** 8.34 (d, J = 8.5 Hz, 2H), 8.20 (d, J = 8.5 Hz, 2H), 7.62 (d, J = 9.4 Hz, 1H), 6.99 (d, J = 9.4 Hz, 1H), 6.87 (s, 1H), 3.94 (s, 3H), 3.88 (s, 3H). **^{13}C NMR (100 MHz, $CDCl_3$) δ:** 165.9, 161.0, 142.6, 138.8, 130.5, 130.3, 123.0, 122.5, 122.1, 114.7, 95.0, 55.7, 52.4. **HRMS (ESI):** calcd for $C_{15}H_{13}N_3O_4$ [M+H]$^+$: 300.0979, found 300.0977.

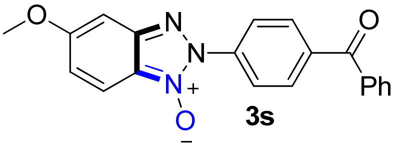

3s

2-(4-Benzoylphenyl)-5-methoxy-2*H*-benzo[*d*][1,2,3]triazole 1-oxide (3s): The title compound was prepared according to the general procedure. The reaction was carried out with $Pd(OAc)_2$ (3

mol%, 0.0014 g), TBAI (3 mol%, 0.0022 g), TsOH (6 mol%, 0.0021 g), **1s** (0.2 mmol, 0.0633 g), [NO][BF_4] (3 equiv, 0.0701 g) in DCB (1.5 mL) at 90 °C for 48 h. **3s** was obtained as white solid (50.0 mg, 72%; eluent: 1:2:5 ether/CH_2Cl_2/hexane). **^{1}H NMR (400 MHz, $CDCl_3$) δ:** 8.38 (d, J = 8.7 Hz, 2H), 7.98 (d, J = 8.7 Hz, 2H), 7.83-7.81 (m, 2H), 7.65-7.59 (m, 2H), 7.50 (t, J = 7.6 Hz, 2H), 7.01 (dd, J = 9.4 Hz, 2.1 Hz, 1H), 6.89 (d, J = 1.9 Hz, 1H), 3.89 (s, 3H). **^{13}C NMR (100 MHz, $CDCl_3$) δ:** 195.2, 161.0, 142.6, 138.2, 137.6, 137.0, 132.7, 130.9, 130.0, 128.4, 123.0, 122.5, 122.1, 114.7, 95.0, 55.7. **HRMS (ESI):** calcd for $C_{20}H_{15}N_3O_3$ [M+H]$^+$: 346.1186, found 346.1186.

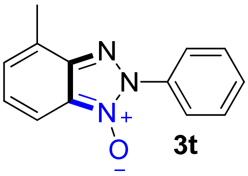

3t

4-Methyl-2-phenyl-2*H*-benzo[*d*][1,2,3]triazole 1-oxide (3t): The title compound was prepared according to the general procedure. The reaction was carried out with Pd(OAc)$_2$ (3 mol%, 0.0014 g), TBAI (3 mol%, 0.0022 g), TsOH (6 mol%, 0.0021 g), **1t** (0.2 mmol, 0.0392 g), [NO][BF_4] (3 equiv, 0.0701 g) in DCB (1.5 mL) at 90 °C for 48 h. **3t** was obtained orange oil (31.0 mg, 69%; eluent: 10%-30% ether/hexane). **^{1}H NMR (400 MHz, $CDCl_3$) δ:** 8.18 (d, J = 8.1 Hz, 2H), 7.62 (dd, J = 16.0 Hz, 8.5 Hz, 3H), 7.55 (d, J = 7.4 Hz, 1H), 7.31-7.20 (m, 2H), 2.66 (s, 3H). **^{13}C NMR (100 MHz, $CDCl_3$) δ:** 141.4, 135.3, 129.8, 129.7, 129.2, 128.0, 126.6, 126.4, 123.7, 111.2, 15.8. **HRMS (ESI):** calcd for $C_{13}H_{11}N_3O$ [M+H]$^+$: 226.0975, found 226.0974.

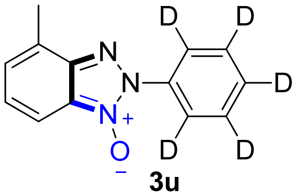

3u

4-Methyl-2-(2,3,4,5,6-peptadeuterophenyl)-2*H*-benzo[*d*][1,2,3]triazole 1-oxide (3u): The title compound was prepared according to the general procedure. The reaction was carried out with Pd(OAc)$_2$ (3 mol%, 0.0014 g), TBAI (3 mol%, 0.0022 g), TsOH (6 mol%, 0.0021 g), **1u** (0.2 mmol, 0.0402 g), [NO][BF_4] (3 equiv, 0.0701 g) in DCB (1.5 mL) at 90 °C for 48 h. **3u** was obtained as orange oil (30.0 mg, 65%; eluent: 10%-30% ether/hexane). **^{1}H NMR (400 MHz, $CDCl_3$) δ:** 7.65 (d, J = 8.5 Hz, 1H), 7.29-7.26 (m, 1H), 7.23-7.21 (m, 1H), 2.67 (s, 3H). **^{13}C NMR (100 MHz, $CDCl_3$) δ:** 141.5, 135.2, 129.7, 129.3 (t, J = 25.2 Hz), 128.7 (t, J = 24.8 Hz), 128.0, 126.6, 126.4, 123.3 (t, J = 25.3 Hz), 111.2, 15.8. **HRMS (ESI):** calcd for $C_{13}H_6D_5N_3O$ [M+H]$^+$: 231.1289, found 231.1288.

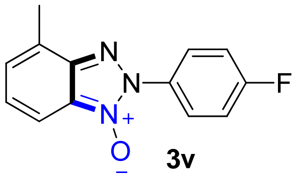

3v

2-(4-Fluorophenyl)-4-methyl-2*H*-benzo[*d*][1,2,3]triazole 1-oxide (3v): The title compound was prepared according to the general procedure. The reaction was carried out with Pd(OAc)$_2$ (3 mol%, 0.0014 g), TBAI (3 mol%, 0.0022 g), TsOH (6 mol%, 0.0021 g), **1v** (0.2 mmol, 0.0428 g), [NO][BF_4] (3 equiv, 0.0701 g) in DCB (1.0 mL) and nitrobenzene (0.5 mL). **3v** was obtained as

orange solid (29.9 mg, 62%; eluent: 1:1:8 ether/CH_2Cl_2/hexane). **^{1}H NMR (400 MHz, $CDCl_3$) δ:** 8.21 (dd, J = 8.5 Hz, 4.8 Hz, 2H), 7.63 (d, J = 8.5 Hz, 1H), 7.32-7.22 (m, 4H), 2.66 (s, 3H). **^{13}C NMR (100 MHz, $CDCl_3$) δ:** 162.9 (d, J = 251.4 Hz), 141.5, 131.4 (d, J = 3.1), 129.7, 128.2, 126.8, 126.3, 125.9 (d, J = 8.9 Hz, 2H), 116.3 (d, J = 23.3 Hz), 111.2, 15.8. **^{19}F NMR (376 MHz, $CDCl_3$) δ:** 109.6. **HRMS (ESI):** calcd for $C_{13}H_{10}N_3OF$ $[M+H]^+$: 244.0881, found 244.0880.

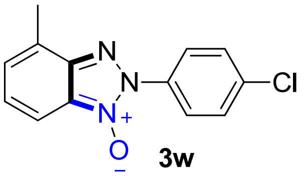

3w

2-(4-Chrolophenyl)-4-methyl-2*H*-benzo[*d*][1,2,3]triazole 1-oxide (3w): The title compound was prepared according to the general procedure. The reaction was carried out with $Pd(OAc)_2$ (3 mol%, 0.0014 g), TBAI (3 mol%, 0.0022 g), TsOH (6 mol%, 0.0021 g), **1w** (0.2 mmol, 0.0461 g), [NO][BF_4] (3 equiv, 0.0701 g) in DCB (1.0 mL) and nitrobenzene (0.5 mL). **3w** was obtained as orange solid (30.8 mg, 59%; eluent: 1:1:8 ether/CH_2Cl_2/hexane). **^{1}H NMR (400 MHz, $CDCl_3$) δ:** 8.22 (d, J = 8.8 Hz, 2H), 7.62 (d, J = 8.5 Hz, 1H), 7.57 (d, J = 8.8 Hz, 2H), 7.27-7.23 (m, 2H), 2.66 (s, 3H). **^{13}C NMR (100 MHz, $CDCl_3$) δ:** 141.6, 135.7, 133.9, 129.7, 129.4, 128.3, 126.84, 124.6, 111.2, 15.8. **HRMS (ESI):** calcd for $C_{13}H_{10}N_3OCl$ $[M+H]^+$: 260.0585, found 260.0584.

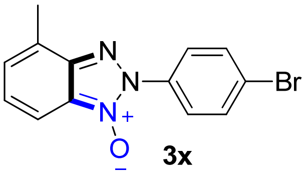

3x

2-(4-Bromophenyl)-4-methyl-2*H*-benzo[*d*][1,2,3]triazole 1-oxide (3x): The title compound was prepared according to the general procedure. The reaction was carried out with $Pd(OAc)_2$ (3 mol%, 0.0014 g), TBAI (3 mol%, 0.0022 g), TsOH (6 mol%, 0.0021 g), **1x** (0.2 mmol, 0.0550 g), [NO][BF_4] (3 equiv, 0.0701 g) in DCB (1.0 mL) and nitrobenzene (0.5 mL). **3x** was obtained as pale yellow solid (33.2 mg, 55%; eluent: 1:1:8 ether/CH_2Cl_2/hexane). **^{1}H NMR (400 MHz, $CDCl_3$) δ:** 8.17-8.15 (m, 1H), 7.74-7.72 (m, 1H), 7.61 (d, J = 8.5 Hz, 1H), 7.29-7.21 (m, 2H), 2.65 (s, 3H). **^{13}C NMR (100 MHz, $CDCl_3$) δ:** 141.6, 134.4, 132.3, 129.7, 128.3, 126.8, 124.78, 123.8, 111.2, 15.8. **HRMS (ESI):** calcd for $C_{13}H_{10}N_3OBr$ $[M+H]^+$: 304.0080, found 304.0079.

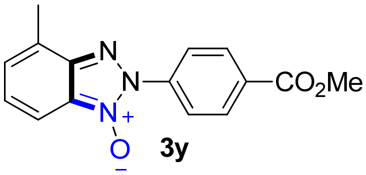

3y

2-(4-(Methoxycarbonyl)phenyl)-4-methyl-2*H*-benzo[*d*][1,2,3]triazole 1-oxide (3y): The title compound was prepared according to the general procedure. The reaction was carried out with $Pd(OAc)_2$ (3 mol%, 0.0014 g), TBAI (3 mol%, 0.0022 g), TsOH (6 mol%, 0.0021 g), **1y** (0.2 mmol, 0.0508 g), [NO][BF_4] (3 equiv, 0.0701 g) in DCB (1.5 mL). **3y** was obtained as pale yellow solid (46.4 mg, 82%; eluent: 10-30% ether/hexane then CH_2Cl_2). **^{1}H NMR (400 MHz, $CDCl_3$) δ:** 8.41 (d, J = 8.5 Hz, 2H), 8.26 (d, J = 8.5 Hz, 2H), 7.61 (d, J = 8.5 Hz, 1H), 7.28-7.25 (m, 1H), 7.22 (d, J = 6.7 Hz, 1H), 3.98 (s, 3H), 2.66 (s, 3H). **^{13}C NMR (100 MHz, $CDCl_3$) δ:** 165.9, 141.8, 138.8, 130.7, 130.5, 129.8, 128.5, 127.0, 126.6, 122.7, 111.2, 52.4, 15.8. **HRMS (ESI):** calcd for

$C_{15}H_{13}N_3O_3$ [M+H]$^+$: 284.1030, found 284.1028.

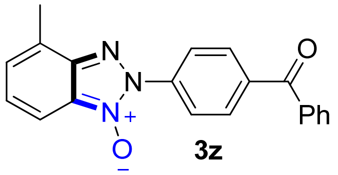

3z

2-(4-Benzoylphenyl)-4-methyl-2*H*-benzo[*d*][1,2,3]triazole 1-oxide 1-oxide (3z): The title compound was prepared according to the general procedure. The reaction was carried out with $Pd(OAc)_2$ (3 mol%, 0.0014 g), TBAI (3 mol%, 0.0022 g), TsOH (6 mol%, 0.0021 g), **1z** (0.2 mmol, 0.0601 g), [NO][BF_4] (3 equiv, 0.0701 g) in DCB (1.5 mL). **3z** is obtained as pale yellow solid (54.7 mg, 83%; eluent: 1:1:3 ether/CH_2Cl_2/hexane). **^{1}H NMR (400 MHz, $CDCl_3$) δ:** 8.45 (d, J = 8.6 Hz, 2H), 8.03 (d, J = 8.6 Hz, 2H), 7.87-7.84 (m, 2H), 7.63 (t, J = 8.1 Hz, 2H), 7.52 (t, J = 7.7 Hz, 2H), 7.30-7.22 (m, 2H), 2.67 (s, 3H). **^{13}C NMR (100 MHz, $CDCl_3$) δ:** 195.2, 141.9, 138.2, 138.0, 137.0, 132.8, 130.9, 130.0, 129.8, 128.5, 128.4, 127.1, 126.6, 122.7, 111.2, 15.8. **HRMS (ESI):** calcd for $C_{20}H_{15}N_3O_2$ [M+H]$^+$: 330.1237, found 330.1234.

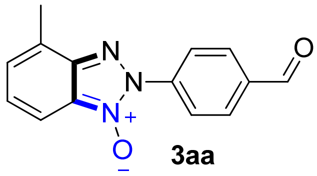

3aa

2-(4-Formylphenyl)-4-methyl-2*H*-benzo[*d*][1,2,3]triazole 1-oxide (3aa): The title compound was prepared according to the general procedure. The reaction was carried out with $Pd(OAc)_2$ (3 mol%, 0.0014 g), TBAI (3 mol%, 0.0022 g), TsOH (6 mol%, 0.0021 g), **1aa** (0.2 mmol, 0.0449 g), [NO][BF_4] (3 equiv, 0.0701 g) in DCB (1.5 mL). **3aa** was obtained as pale yellow solid (27.4 mg, 54%; eluent: 1:1:3 ether/CH_2Cl_2/hexane). **^{1}H NMR (400 MHz, $CDCl_3$) δ:** 10.12 (s, 1H), 8.55 (d, J = 8.5 Hz, 2H), 8.11 (d, J = 8.4 Hz, 2H), 7.62 (d, J = 8.5 Hz, 1H), 7.30-7.22 (m, 2H), 2.66 (s, 3H). **^{13}C NMR (100 MHz, $CDCl_3$) δ:** 190.8, 142.0, 139.7, 136.2, 130.5, 129.8, 128.6, 127.2, 123.2, 111.2, 15.8. **HRMS (ESI):** calcd for $C_{14}H_{11}N_3O_2$ [M+H]$^+$: 254.0924, found 254.0923.

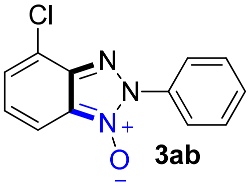

3ab

4-Chloro-2-phenyl-2*H*-benzo[*d*][1,2,3]triazole 1-oxide (3ab): The title compound was prepared according to the general procedure. The reaction was carried out with $Pd(OAc)_2$ (3 mol%, 0.0014 g), TBAI (3 mol%, 0.0022 g), TsOH (6 mol%, 0.0021 g), **1ab** (0.2 mmol, 0.0433 g), [NO][BF_4] (3 equiv, 0.0701 g) in DCB (1.5 mL). **3ab** was obtained as pale yellow solid (20.0 mg, 41%; eluent: 10%-30% ether/hexane). **^{1}H NMR (400 MHz, $CDCl_3$) δ:** 8.17 (d, J = 8.0, 2H), 7.74 (d, J = 8.7, 1H), 7.62-7.53 (m, 3H), 7.48 (d, J = 7.3, 1H), 7.29-7.26 (m, 1H). **^{13}C NMR (100 MHz, $CDCl_3$) δ:** 139.0, 135.0, 130.3, 129.3, 128.5, 127.5, 126.2, 124.6, 123.8, 112.9. **HRMS (ESI):** calcd for $C_{12}H_8N_3OCl$ [M+H]$^+$: 246.0429, found 246.0428.

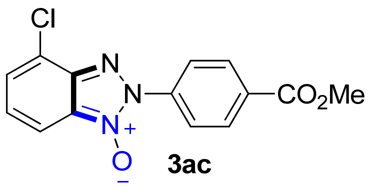

3ac

4-Chloro-2-(4-(methoxycarbonyl)phenyl)-2*H*-benzo[*d*][1,2,3]triazole 1-oxide (3ac): The title compound was prepared according to the general procedure. The reaction was carried out with $Pd(OAc)_2$ (3 mol%, 0.0014 g), TBAI (3 mol%, 0.0022 g), TsOH (6 mol%, 0.0021 g), **1ac** (0.2 mmol, 0.0548 g), [NO][BF_4] (3 equiv, 0.0701 g) in DCB (1.0 mL) and nitrobenzene (0.5 mL). **3ac** was obtained as pale yellow solid (37.0 mg, 61%; eluent: 1:3:96-1:3:60 ether/CH_2Cl_2/hexane). **^{1}H NMR (400 MHz, $CDCl_3$) δ:** 8.41 (d, *J* = 8.8 Hz, 2H), 8.27 (d, *J* = 8.8 Hz, 2H), 7.73 (d, *J* = 8.7 Hz, 1H), 7.51 (d, *J* = 7.2 Hz, 1H), 7.31-7.27 (m, 1H), 3.99 (s, 3H). **^{13}C NMR (100 MHz, $CDCl_3$) δ:** 165.7, 139.3, 138.3, 131.2, 130.6, 128.9, 127.7, 126.5, 124.6, 122.9, 112.8, 52.5. **HRMS (ESI):** calcd for $C_{14}H_{10}N_3O_3Cl$ $[M+H]^+$: 304.0483, found 304.0483.

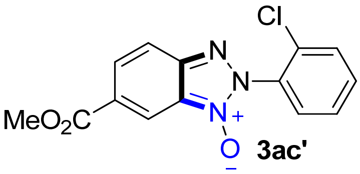

3ac'

2-(2-Chlorophenyl)-6-(methoxycarbonyl)-2*H*-benzo[*d*][1,2,3]triazole 1-oxide (3ac'): The title compound was prepared according to the general procedure. The reaction was carried out with $Pd(OAc)_2$ (3 mol%, 0.0014 g), TBAI (3 mol%, 0.0022 g), TsOH (6 mol%, 0.0021 g), **1ac** (0.2 mmol, 0.0548 g), [NO][BF_4] (3 equiv, 0.0701 g) in DCB (1.0 mL) and nitrobenzene (0.5 mL). **3ac'** was obtained as pale yellow solid (4.9 mg, 8%; eluent: 1:3:100-1:3:10 ether/CH_2Cl_2/hexane). **^{1}H NMR (400 MHz, $CDCl_3$) δ:** 8.62 (s, 1H), 8.09 (dd, *J* = 9.2 Hz, 1.4 Hz, 1H), 7.82 (d, *J* = 9.2 Hz, 1H), 7.69-7.66 (m, 1H), 7.64-7.61 (m, 2H), 7.55-7.53 (m, 1H), 3.99 (s, 3H). **^{13}C NMR (100 MHz, $CDCl_3$) δ:** 165.7, 143.0, 133.0, 133.0, 132.1, 130.9, 129.7, 129.1, 128.2, 127.9, 124.8, 119.5, 118.0, 52.7. **HRMS (ESI):** calcd for $C_{14}H_{10}N_3O_3Cl$ $[M+H]^+$: 304.0483, found 304.0482.

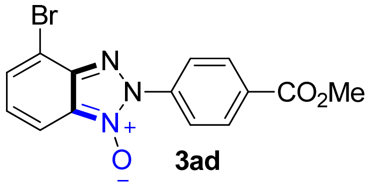

3ad

4-Bromo-2-(4-(methoxycarbonyl)phenyl)-2*H*-benzo[*d*][1,2,3]triazole 1-oxide (3ad): The title compound was prepared according to the general procedure. The reaction was carried out with $Pd(OAc)_2$ (3 mol%, 0.0014 g), TBAI (3 mol%, 0.0022 g), TsOH (6 mol%, 0.0021 g), **1ad** (0.2 mmol, 0.0638 g), [NO][BF_4] (3 equiv, 0.0701 g) in DCB (1.0 mL) and nitrobenzene (0.5 mL). **3ad** was obtained as pale yellow solid (51.9 mg, 74%; eluent: 1:3:96-1:3:60 ether/CH_2Cl_2/hexane). **^{1}H NMR (400 MHz, $CDCl_3$) δ:** 8.38 (d, *J* = 8.7 Hz, 2H), 8.25 (d, *J* = 8.7 Hz, 2H), 7.76 (d, *J* = 8.7 Hz, 1H), 7.68 (d, *J* = 7.2 Hz, 1H), 7.24-7.20 (m, 1H), 3.97 (s, 3H). **^{13}C NMR (100 MHz, $CDCl_3$) δ:** 165.7, 140.4, 138.3, 132.3, 131.2, 130.6, 127.4, 126.9, 122.9, 113.4, 112.7, 52.5. **HRMS (ESI):** calcd for $C_{14}H_{10}N_3O_3Br$ $[M+H]^+$: 347.9978, found 347.9977.

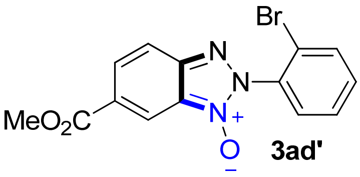

3ad'

2-(2-Bromophenyl)-6-(methoxycarbonyl)-2*H*-benzo[*d*][1,2,3]triazole 1-oxide (3ad'): The title compound was prepared according to the general procedure. The reaction was carried out with $Pd(OAc)_2$ (3 mol%, 0.0014 g), TBAI (3 mol%, 0.0022 g), TsOH (6 mol%, 0.0021 g), **1ad** (0.2 mmol, 0.0638 g), [NO][BF_4] (3 equiv, 0.0701 g) in DCB (1.0 mL) and nitrobenzene (0.5 mL). **3ad'** was obtained as pale yellow solid (6.7 mg, 10%; eluent: 1:3:100-1:3:10 ether/CH_2Cl_2/hexane). **^{1}H NMR (400 MHz, $CDCl_3$) δ:** 8.62 (s, 1H), 8.09 (dd, *J* = 9.2 Hz, 1.4 Hz, 1H), 7.84 (t, *J* = 7.8 Hz, 2H), 7.60-7.56 (m, 3H), 3.99 (s, 3H). **^{13}C NMR (100 MHz, $CDCl_3$) δ:** 165.7, 142.9, 134.0, 133.9, 133.2, 130.0, 129.1, 128.5, 128.2, 124.8, 122.2, 119.5, 118.0, 52.7. **HRMS (ESI):** calcd for $C_{14}H_{10}N_3O_3Br$ $[M+H]^+$: 347.9978, found 347.9977.

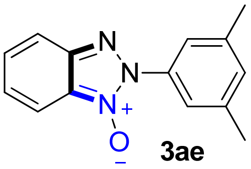

3ae

2-(3,5-Dimethylphenyl)-2*H*-benzo[*d*][1,2,3]triazole 1-oxide (3ae): The title compound was prepared as an inseperable mixture of **3ae** and **3ae'** according to the general procedure. The reaction was carried out with $Pd(OAc)_2$ (3 mol%, 0.0014 g), TBAI (3 mol%, 0.0022 g), TsOH (6 mol%, 0.0021 g), **1ae** (0.2 mmol, 0.0421 g), [NO][BF_4] (3 equiv, 0.0701 g) in DCB (1.5 mL). The mixture of **3ae** and **3ae'** was obtained as light yellow oil (29.4 mg, mixture of **3ae** and **3ae', 3ae** : **3ae**' = 18.5, eluent: 10-30% ether/hexane). **^{1}H NMR (400 MHz, $CDCl_3$) δ:** 7.81-7.76 (m, 2H), 7.74 (s, 2H), 7.46-7.42 (m, 1H), 7.34 (t, *J* = 7.7 Hz, 1H), 7.15 (s, 1H), 2.42 (s, 6H). **^{13}C NMR (100 MHz, $CDCl_3$) δ:** 140.9, 139.1, 135.0, 131.7, 129.0, 126.5, 126.2, 121.3, 119.0, 114.0, 21.3. **HRMS (ESI):** calcd for $C_{14}H_{13}N_3O$ $[M+H]^+$: 240.1131, found 240.1131.

Computational Section

Computational methods. All geometry optimizations were carried out with the hybrid density functional theory (DFT) at the level of M06[12] using DZP basis sets. Here, the DZP stands for a basis set that employs 6-31G(d) all-electron basis set[13] for H, C, N, and O atoms, and the corresponding basis sets with the Stuttgart/Dresden effective-core potential (SDD)[14-16] for the palladium and iodine atoms. Analytical frequencies were calculated to confirm the correctness of the structure of either a local minimum or a transition state (TS). The solvation effects were considered using the IEF-PCM model[17] with 1,2-dichlorobenzene as the model solvent. A step size of 0.1 amu$^{1/2}$ bohr was used in the IRC (intrinsic reaction coordinate) procedure[18] to check the connectivity between a transition state and the reactant as well as the product.

The optimized structures are then adopted to calculate the free energies at the level of M06 functional[12] with TZP basis sets. Here TZP stands for a basis set that employs a 6-311++G(2d,2p) all-electron basis set[19] for the main group elements (6-311G(d) for iodine atom)[20] and the corresponding basis sets with the Stuttgart/Dresden effective-core potential (SDD)[14-16] for the

palladium atom. The free energy at M06/TZP level in solution phase was calculated according to Eq. 1:[21-23]

$$G_{\text{soln}}^{\text{M06/TZP}} = E_{\text{gas}}^{\text{M06/TZP}} + \Delta G_{\text{thermo(soln)}}^{\text{M06/DZP}} + \Delta G_{\text{solv}} + RT \ln\left(\frac{RT}{P}\right) \qquad (1)$$

The first term in the right-hand side is the electronic energy computed at M06/TZP level in gas phase. The second term is the thermal correction to the free energy of the solute in the solution phase at M06/DZP level. The third term is the solvation free energy. The last term denotes the free energy correction from the gas-phase standard state (1 atm) to the solution phase standard state of 1 M. It should be noted that the solvation free energy ΔG_{solv} was obtained by using SMD model[24] at the level of B3LYP/6-31G(d) to make it consistent with the specific methods used in the development of such solvation model.[21-23] All calculations were carried out using the Gaussian 09 program.[25]

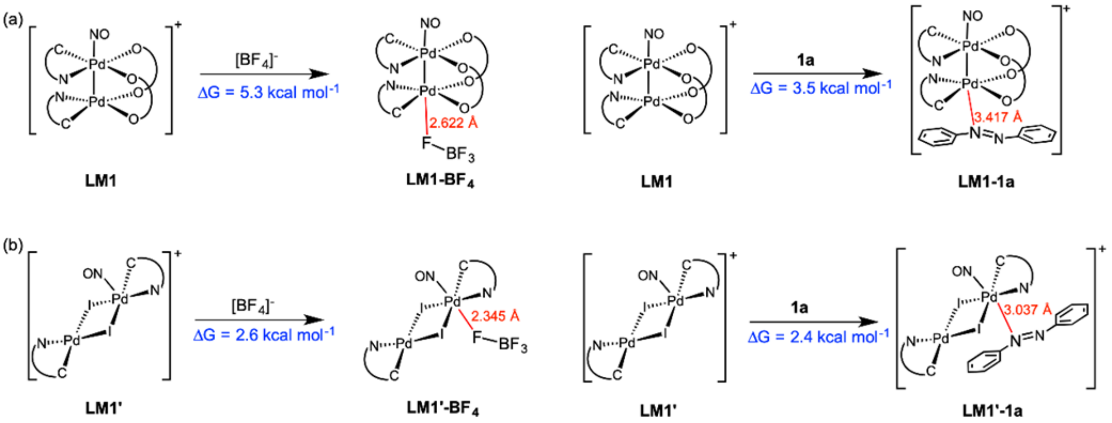

Supplementary Figure 15. Computed free energy changes for $[BF_4]^-$ and azobenezene 1a coordinate to the unoccupied axial site of (a) Pd(III)-Pd(III) intermediate LM1, and (b) Pd(II)-Pd(IV) intermediate LM1'. As shown, all of the considered coordination steps are endothermic. It is worthy to note the distances between Pd center and azobenezene **1a** are too long for an effective chemical interaction. We suggest the steric repulsion between the axial azobenezene **1a** and equatorial ligands hinders the coordination.

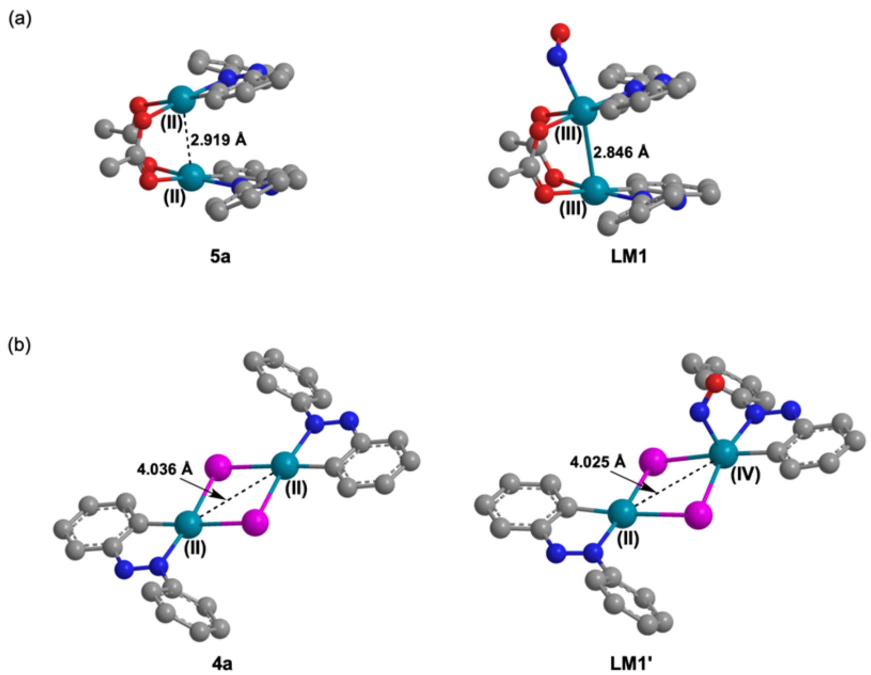

Supplementary Figure 16. Optimized structures for (a) acetate-bridged bipalladium species 5a and LM1, and (b) iodide-bridged bipalladium species 4a and LM1'. LM1 could be considered as a Pd(III)-Pd(III) species with a direct chemical interaction between the two palladium atoms. However, **LM1'** should be a Pd(II)-Pd(IV) species without the direct Pd-Pd interaction.

X-ray Crystallographic Analysis

Supplementary Table 12. Crystal data and structure refinement for **3a (CCDC 1428672)**

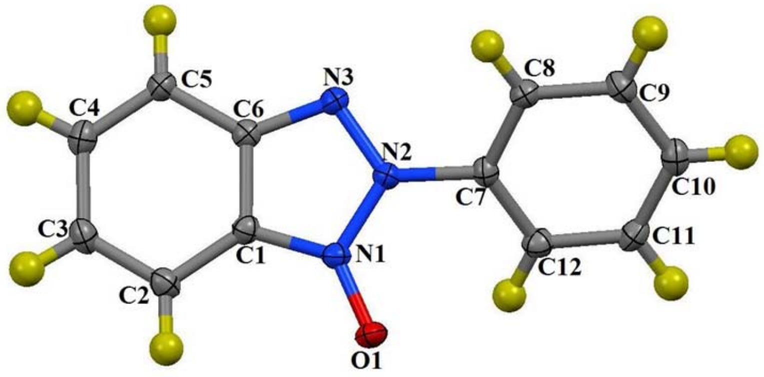

Identification code	Compound-1
Empirical formula	C12 H9 N3 O
Formula weight	211.22
Temperature	100(2) K
Wavelength	1.54184 Å
Crystal system	Monoclinic

Space group	Cc	
Unit cell dimensions	a = 6.4226(2) Å	α= 90°.
	b = 23.1159(8) Å	β= 107.696(4)°.
	c = 6.9516(3) Å	γ = 90°.
Volume	983.23(7) Å^3	
Z	4	
Density (calculated)	1.427 Mg/m^3	
Absorption coefficient	0.776 mm^{-1}	
F(000)	440	
Crystal size	0.320 x 0.220 x 0.080 mm^3	
Theta range for data collection	3.824 to 72.787°.	
Index ranges	-6<=h<=7, -27<=k<=27, -7<=l<=8	
Reflections collected	1658	
Independent reflections	1119 [R(int) = 0.0124]	
Completeness to theta = 67.684°	99.7 %	
Absorption correction	Semi-empirical from equivalents	
Max. and min. transmission	1.00000 and 0.76102	
Refinement method	Full-matrix least-squares on F^2	
Data / restraints / parameters	1119 / 2 / 146	
Goodness-of-fit on F^2	1.092	
Final R indices [I>2sigma(I)]	R1 = 0.0243, wR2 = 0.0633	
R indices (all data)	R1 = 0.0248, wR2 = 0.0638	
Absolute structure parameter	0.0(3)	
Extinction coefficient	0.0049(6)	
Largest diff. peak and hole	0.226 and -0.126 e.Å^{-3}	

Supplementary Table 13. Crystal data and structure refinement for complex **4a (CCDC 1858247)**

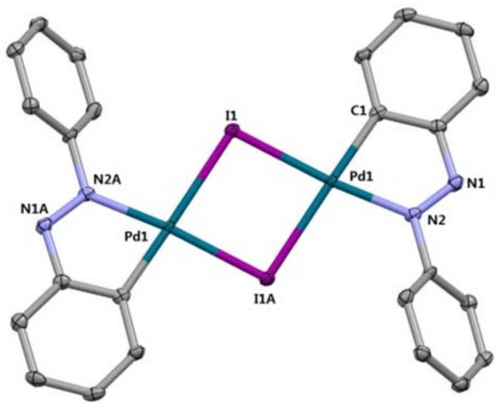

Identification code	complex **4a**

Empirical formula	C24 H18 I2 N4 Pd2	
Formula weight	829.02	
Temperature	100(2) K	
Wavelength	1.54178 Å	
Crystal system	Triclinic	
Space group	P-1	
Unit cell dimensions	a = 4.18530(10) Å	α= 110.318(2)°.
	b = 10.9291(3) Å	β= 90.072(2)°.
	c = 13.2771(3) Å	γ = 96.088(2)°.
Volume	565.84(2) $Å^3$	
Z	1	
Density (calculated)	2.433 Mg/m^3	
Absorption coefficient	34.402 mm^{-1}	
F(000)	388	
Crystal size	0.18 x 0.05 x 0.02 mm^3	
Theta range for data collection	3.55 to 69.95°.	
Index ranges	-5<=h<=4, -13<=k<=13, -16<=l<=16	
Reflections collected	19939	
Independent reflections	2125 [R(int) = 0.0430]	
Completeness to theta = 69.95°	99.6 %	
Absorption correction	Semi-empirical from equivalents	
Max. and min. transmission	0.5462 and 0.0629	
Refinement method	Full-matrix least-squares on F^2	
Data / restraints / parameters	2125 / 0 / 145	
Goodness-of-fit on F^2	1.079	
Final R indices [I>2sigma(I)]	R1 = 0.0268, wR2 = 0.0678	
R indices (all data)	R1 = 0.0289, wR2 = 0.0690	
Largest diff. peak and hole	1.220 and -1.169 e.$Å^{-3}$	

Supplementary Table 14. Crystal data and structure refinement for comlex **4b (CCDC 1858251)**

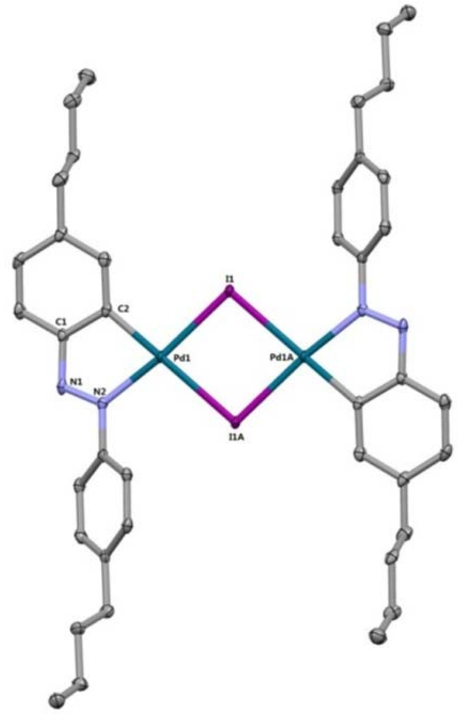

Identification code	complex **4b**	
Empirical formula	C40 H50 I2 N4 Pd2	
Formula weight	1053.44	
Temperature	100(2) K	
Wavelength	1.54178 Å	
Crystal system	Triclinic	
Space group	P-1	
Unit cell dimensions	a = 4.63200(10) Å	α= 65.897(2)°.
	b = 13.8323(5) Å	β= 85.801(2)°.
	c = 16.6380(6) Å	γ = 81.030(2)°.
Volume	961.12(5) Å^3	
Z	1	
Density (calculated)	1.820 Mg/m^3	
Absorption coefficient	20.405 mm^{-1}	
F(000)	516	
Crystal size	0.22 x 0.20 x 0.05 mm^3	
Theta range for data collection	3.53 to 69.99°.	
Index ranges	-5<=h<=3, -16<=k<=16, -19<=l<=20	
Reflections collected	6474	
Independent reflections	3630 [R(int) = 0.0302]	

Completeness to theta = 69.99°	99.2 %
Absorption correction	Semi-empirical from equivalents
Max. and min. transmission	0.4285 and 0.0939
Refinement method	Full-matrix least-squares on F^2
Data / restraints / parameters	3630 / 0 / 217
Goodness-of-fit on F^2	1.044
Final R indices [I>2sigma(I)]	R1 = 0.0387, wR2 = 0.1041
R indices (all data)	R1 = 0.0394, wR2 = 0.1047
Largest diff. peak and hole	1.738 and -1.924 e.Å^{-3}

Supplementary Table 15. Crystal data and structure refinement for complex **5b** **(CCDC 1858266)**

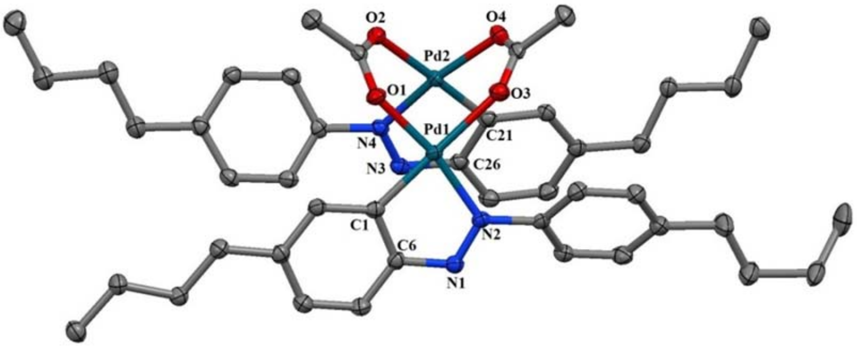

Identification code	complex **5b**	
Empirical formula	C44 H56 N4 O4 Pd2	
Formula weight	917.73	
Temperature	100(2) K	
Wavelength	1.54184 Å	
Crystal system	Triclinic	
Space group	P-1	
Unit cell dimensions	a = 10.9439(3) Å	α= 85.362(2)°.
	b = 11.2900(3) Å	β= 75.755(2)°.
	c = 17.5270(5) Å	γ = 81.146(2)°.
Volume	2071.89(9) Å^3	
Z	2	
Density (calculated)	1.471 Mg/m^3	
Absorption coefficient	7.365 mm^{-1}	
F(000)	944	
Crystal size	0.40 x 0.20 x 0.12 mm^3	
Theta range for data collection	3.97 to 70.00°.	
Index ranges	-13<=h<=13, -13<=k<=13, -21<=l<=21	
Reflections collected	37130	

Independent reflections	7858 [R(int) = 0.0382]
Completeness to theta = 70.00°	99.9 %
Absorption correction	Semi-empirical from equivalents
Max. and min. transmission	0.4719 and 0.1566
Refinement method	Full-matrix least-squares on F^2
Data / restraints / parameters	7858 / 0 / 506
Goodness-of-fit on F^2	1.051
Final R indices [I>2sigma(I)]	R1 = 0.0277, wR2 = 0.0756
R indices (all data)	R1 = 0.0301, wR2 = 0.0772
Largest diff. peak and hole	0.634 and -0.942 e.Å^{-3}

Supplementary References

1. Zhang, C. & Jiao, N. Copper-catalyzed aerobic oxidative dehydrogenative coupling of anilines leading to aromatic azo compounds using dioxygen as an oxidant. *Angew. Chem., Int. Ed.* **49**, 6174- 6177 (2010).
2. Lian, Y., Bergman, R. G., Lavis, L. D. & Ellman, J. A. *J. Am. Chem. Soc.* **135**, 7122-7125 (2013).
3. Beate, P. & Karola, R. Efficient preparation of nitrosoarenes for the synthesis of azobenzenes. *J. Org. Chem.* **70**, 2350-2352 (2005).
4. Samanta, A. & Ravoo, B. J. *Chem. Eur. J.* **20**, 4966-4893 (2014).
5. Rahim, M. A., Rao, P. N. P. & Knaus, E. E. Synthesis of 4-alkyl-1,2-diphenyl-3,5-dioxopyrazolidines possessing aryl methylsulfonyl and sulfonamide pharmacophores for evaluation as selective cyclooxygenase-2 (COX-2) inhibitors. *J. Heterocyclic Chem.* **39**, 1309-1314 (2002).
6. Dai, J.-J., Fang, C., Xiao, B., Yi, J., Xu, J., Liu, Z.-J.; Lu, X., Liu, L. & Fu, Y. Copper-promoted Sandmeyer trifluoromethylation reaction. *J. Am. Chem. Soc.* **135**, 8436-8439 (2013).
7. Ishikawa, N., Namkung, M. J. & Fletcher, T. L. Fluorinated azo dyes.[1a] I. synthesis and spectral properties of 3,5-difluoro-4-*N*-methylaminoazobenzene, 2,6-difluoroacetanilide, and related compounds. *J. Org. Chem.* **30**, 3878-3882 (1965).
8. Khalaf, A. I., Anthony, N., Breen, D., Donoghue, G., MacKay, S. P., Scott, F. J. & Suckling, C. J. Amide isosteres in structure-activity studies of antibacterial minor groove binders. *Eur. J. Med. Chem.* **46**, 5343-5355 (2011).
9. Zhao, R., Tan, C., Xie, Y.; Gao, C., Liu, H. & Jiang, Y. One step synthesis of azo compounds from nitroaromatics and anilines. *Tetrahedron Lett*, **52**, 3805-3809 (2011).
10. Liu, X., Li, H.-Q., Ye, S., Liu, Y.-M., He, H.-Y. & Cao, Y. Gold-catalyzed direct hydrogenative coupling of nitroarenes to synthesize aromatic azo compounds. *Angew. Chem., Int. Ed.* **53**, 7624-7628 (2014).
11. Lux, J. & Rebek, J. Reversible switching between self-assembled homomeric and hybrid capsules. *Chem. Comm.* **49**, 2127-2129 (2013).
12. Zhao, Y. & Truhlar, D. G. The M06 suite of density functionals for main group

thermochemistry, thermochemical kinetics, noncovalent interactions, excited states, and transition elements: two new functionals and systematic testing of four M06-class functionals and 12 other functionals. *Theor. Chem. Acc.* **120**, 215-241 (2008).

13. Petersson, G. A., Bennett, A., Tensfeldt, T. G., Allaham, M. A., Shirley, W. A. & Mantzaris, J. A complete basis set model chemostry .1. The total energies of closed-shell atoms and hydrides of the 1st-row elements. *J. Chem. Phys.* **89**, 2193-2218 (1988).
14. Häussermann, U., Dolg, M., Stoll, H., Preuss, H., Schwerdtfeger, P. & Pitzer, R. M. Accuracy of energy-adjusted quasirelativistic ab initio pseudopotentials. *Mol. Phys.* **78**, 1211-1224 (1993).
15. Kuchle, W., Dolg, M., Stoll, H. & Preuss, H. Energy-adjusted pseudopotentials for the actinides - parameter sets and test calculations for thorium and thorium monoxide. *J. Chem. Phys.* **100**, 7535-7542 (1994).
16. Leininger, T., Nicklass, A., Stoll, H., Dolg, M. & Schwerdtfeger, P. The accuracy of the pseudopotential approximation .2. A comparison of various core sizes for indium pseudopotentials in calculations for spectroscopic constants of InH, InF, and InCl. *J. Chem. Phys.* **105**, 1052-1059 (1996).
17. Tomasi, J., Mennucci, B. & Cammi, R. Quantum mechanical continuum solvation models. *Chem. Rev.* **105**, 2999-3093 (2005).
18. Gonzalez, C. & Schlegel, H. B. An Improved Algorithm for Reaction-Path Following. *J. Chem. Phys.* **90**, 2154-2161 (1989).
19. Krishnan, R., Binkley, J. S., Seeger, R. & Pople, J. A. Self-consistent molecular-orbital methods .20. Basis set for correlated wave-functions. *J. Chem. Phys.* **72**, 650-654 (1980).
20. Glukhovtsev, M. N., Pross, A., McGrath, M. P. & Radom, L. Extension of Gaussian - 2 (G2) theory to bromine - and iodine - containing molecules: Use of effective core potentials. *J. Chem. Phys.* **103**, 1878-1885 (1995).
21. Ho, J. M., Klamt, A. & Coote, M. L. Comment on the Correct Use of Continuum Solvent Models. *J. Phys. Chem. A* **114**, 13442-13444 (2010).
22. Liu, G., Wu, J. M., Zhang, I. Y., Chen, Z. N., Li, Y. W. & Xu, X. Theoretical Studies on Thermochemistry for Conversion of 5-Chloromethylfurfural into Valuable Chemicals. *J. Phys. Chem. A* **115**, 13628-13641 (2011).
23. Chen, Z. N., Chan, K. Y., Pulleri, J. K., Kong, J. & Hu, H. Theoretical Study on the Mechanism of Aqueous Synthesis of Formic Acid Catalyzed by Ru3+ -EDTA Complex. *Inorg. Chem.* **54**, 1314-1324 (2015).
24. Marenich, A. V., Cramer, C. J. & Truhlar, D. G. Universal Solvation Model Based on Solute Electron Density and on a Continuum Model of the Solvent Defined by the Bulk Dielectric Constant and Atomic Surface Tensions. *J. Phys. Chem. B* **113**, 6378-6396 (2009).
25. Frisch, M. J., Trucks, G. W., Schlegel, H. B., Scuseria, G. E., Robb, M. A., Cheeseman, J. R., Scalmani, G., Barone, V., Mennucci, B., Petersson, G. A., Nakatsuji, H., Caricato, M., Li, X., Hratchian, H. P., Izmaylov, A. F., Bloino, J., Zheng, G., Sonnenberg, J. L., Hada, M., Ehara, M., Toyota, K., Fukuda, R., Hasegawa, J., Ishida, M., Nakajima, T., Honda, Y., Kitao, O., Nakai, H., Vreven, T., Montgomery, J. A., Peralta, J. J. E., Ogliaro, F., Bearpark, M., Heyd, J. J., Brothers, E., Kudin, K. N., Staroverov, V. N., Kobayashi, R., Normand, J., Raghavachari, K., Rendell, A., Burant, J. C., Iyengar, S. S., Tomasi, J., Cossi, M., Rega, N., Millam, J. M., Klene, M., Knox, J. E., Cross, J. B., Bakken, V., Adamo, C., Jaramillo, J., Gomperts, R.,

Stratmann, R. E., Yazyev, O., Austin, A. J., Cammi, R., Pomelli, C., Ochterski, J. W., Martin, R. L., Morokuma, K., Zakrzewski, V. G., Voth, G. A., Salvador, P., Dannenberg, J. J., Dapprich, S., Daniels, A. D., Farkas, Ö., Foresman, J. B., Ortiz, J. V., Cioslowski, J. & Fox, D. J. Gaussian 09, Revision D. 01. Wallingford, CT: Gaussian, Inc.; 2009.

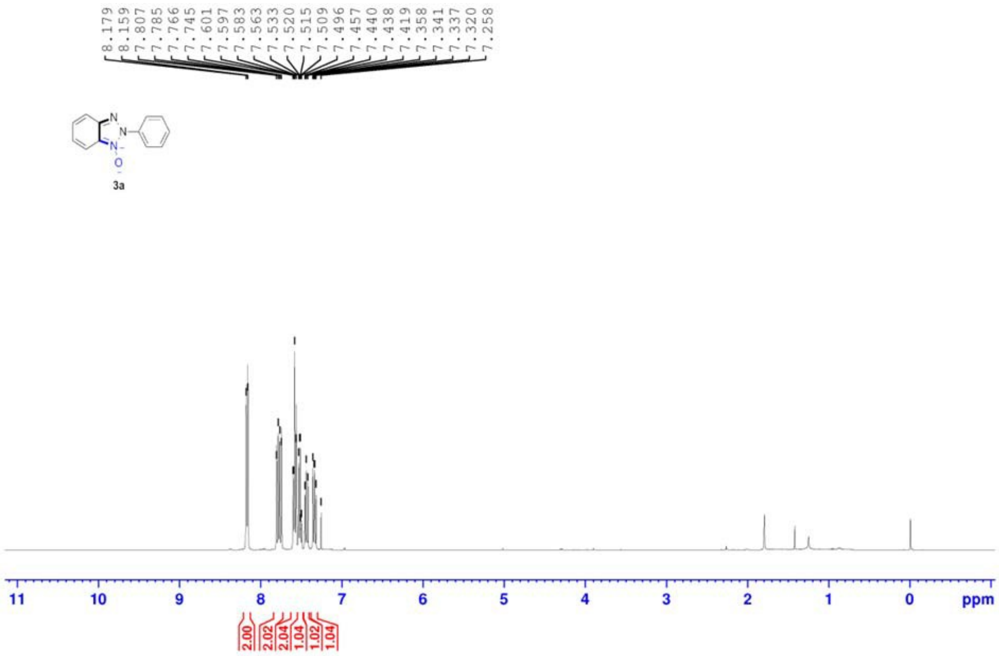

Supplementary Figure 17. ^{1}H NMR spectra of **3a**.

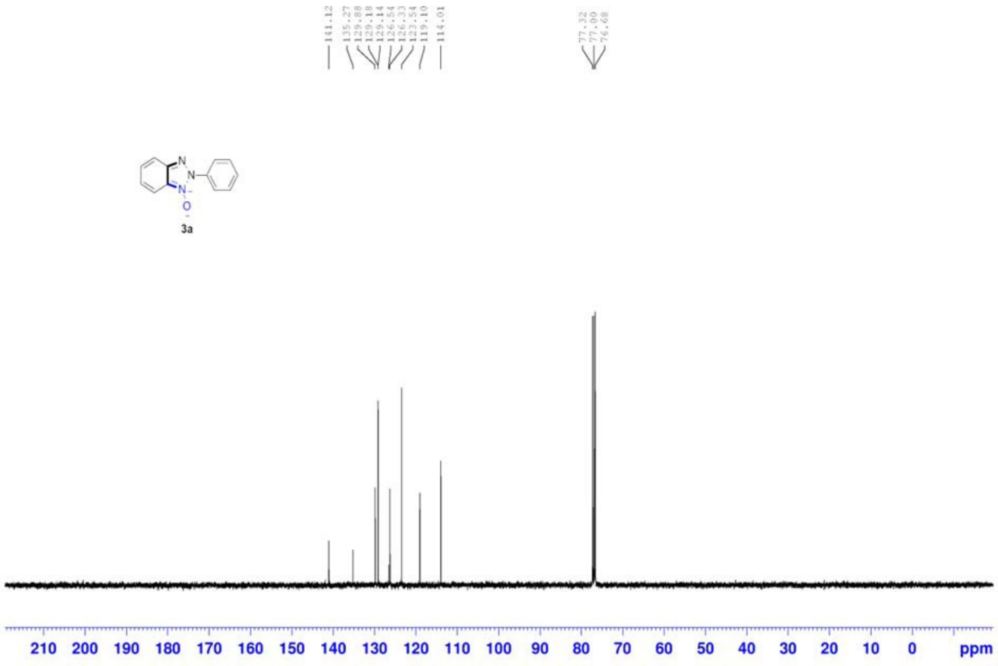

Supplementary Figure 18. ^{13}C NMR spectra of **3a**.

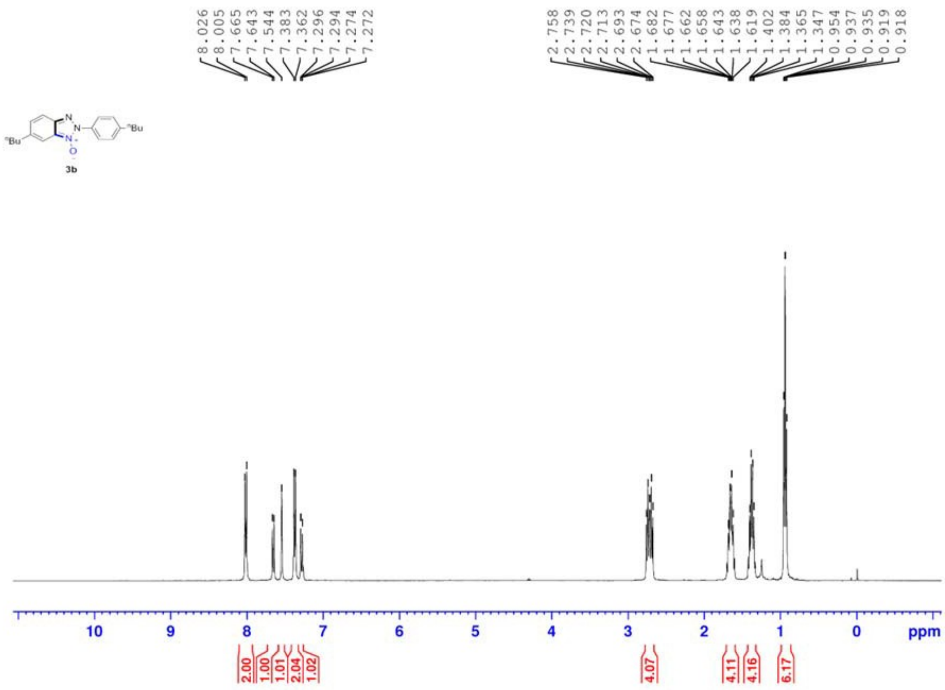

Supplementary Figure 19. ^{1}H NMR spectra of **3b**.

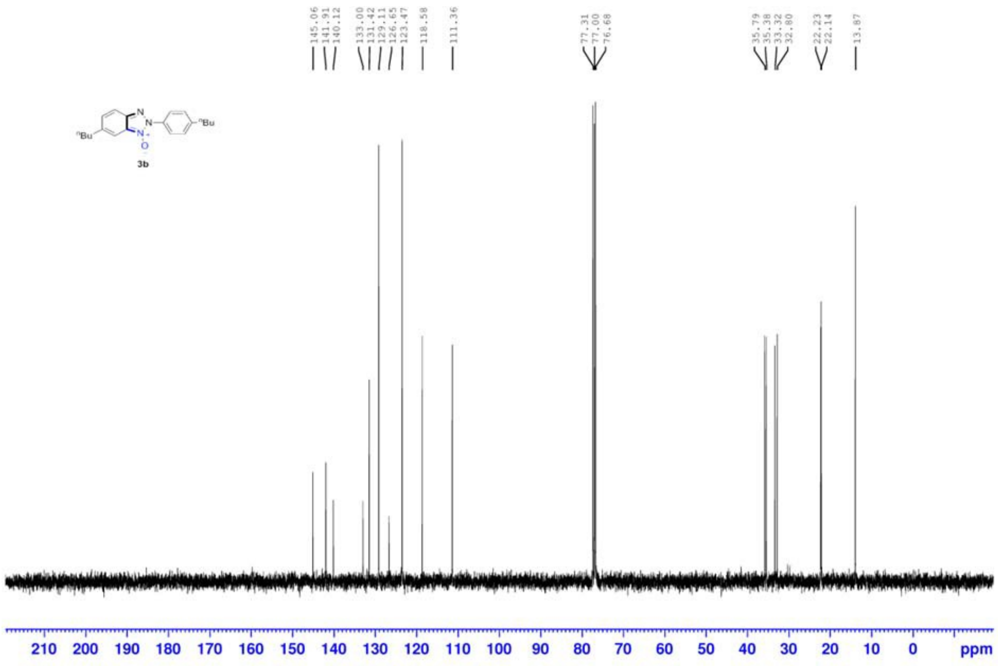

Supplementary Figure 20. ^{13}C NMR spectra of **3b**.

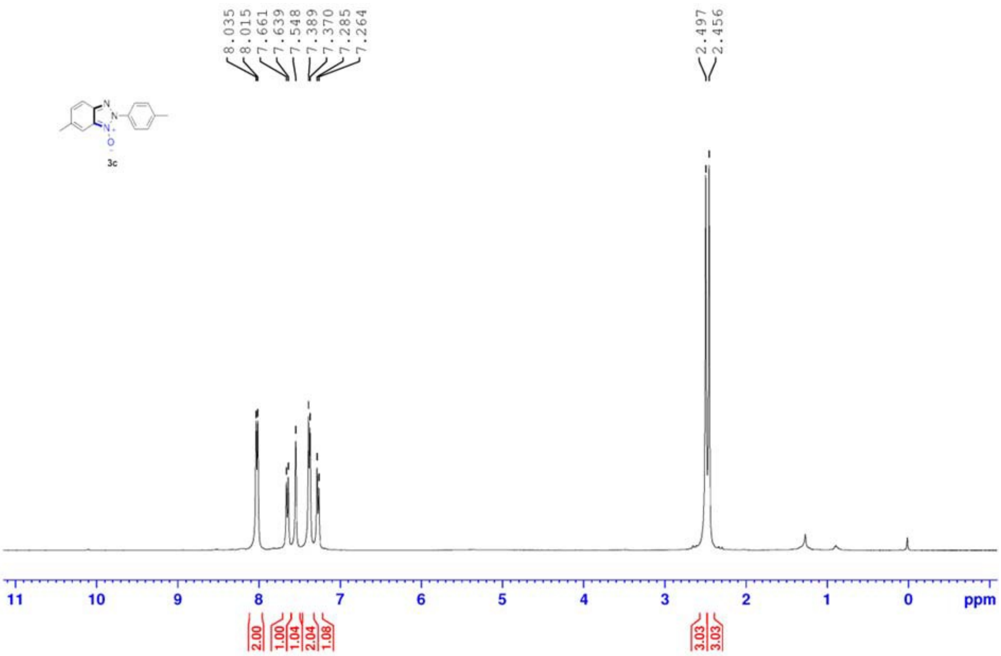

Supplementary Figure 21. ^{1}H NMR spectra of **3c**.

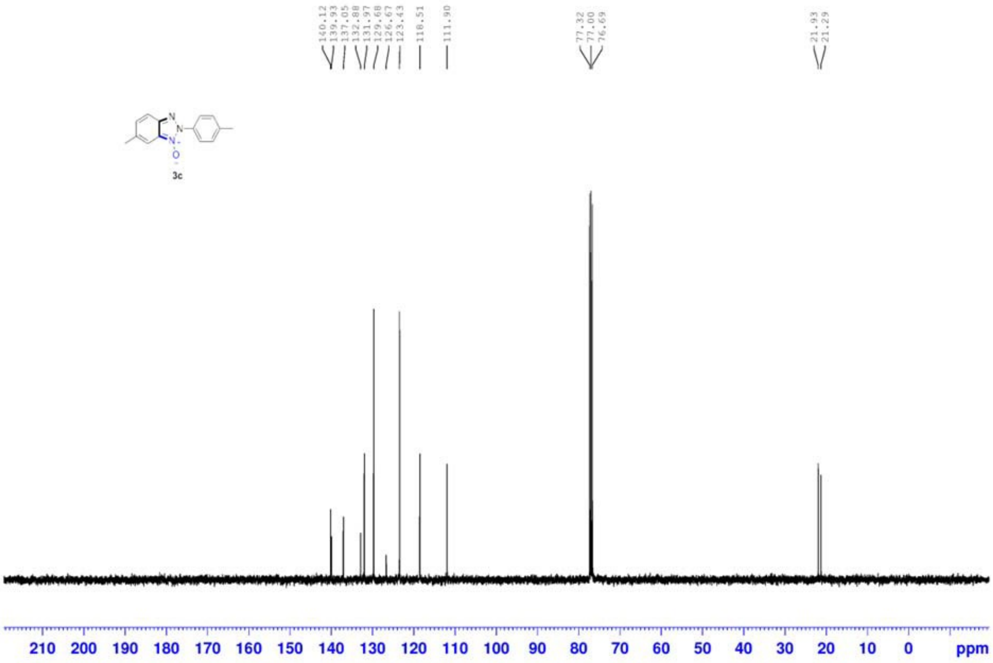

Supplementary Figure 22. ^{13}C NMR spectra of **3c**.

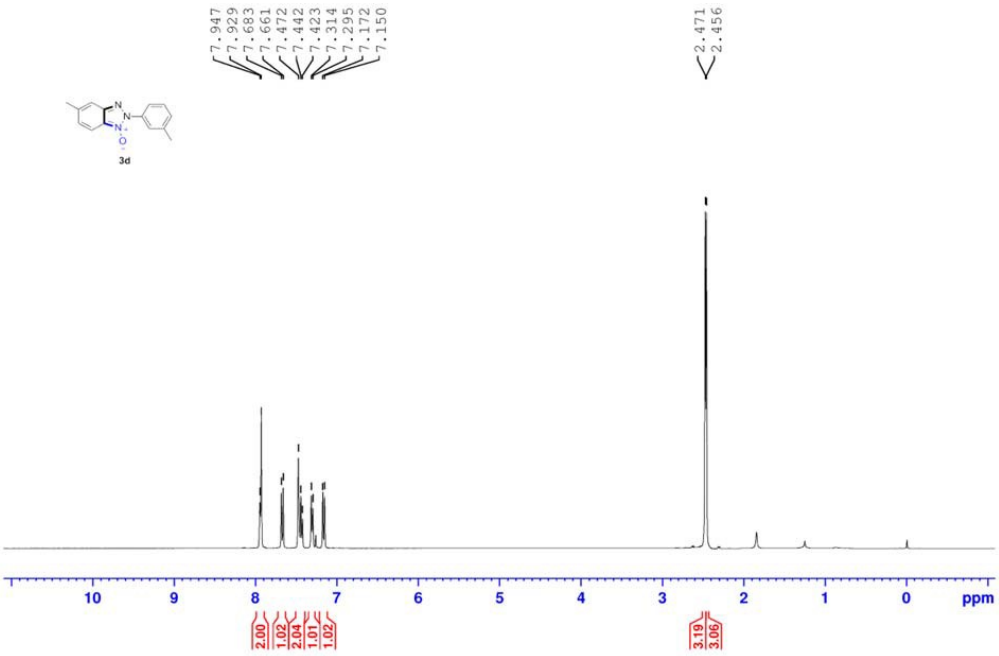

Supplementary Figure 23. ^{1}H NMR spectra of **3d**.

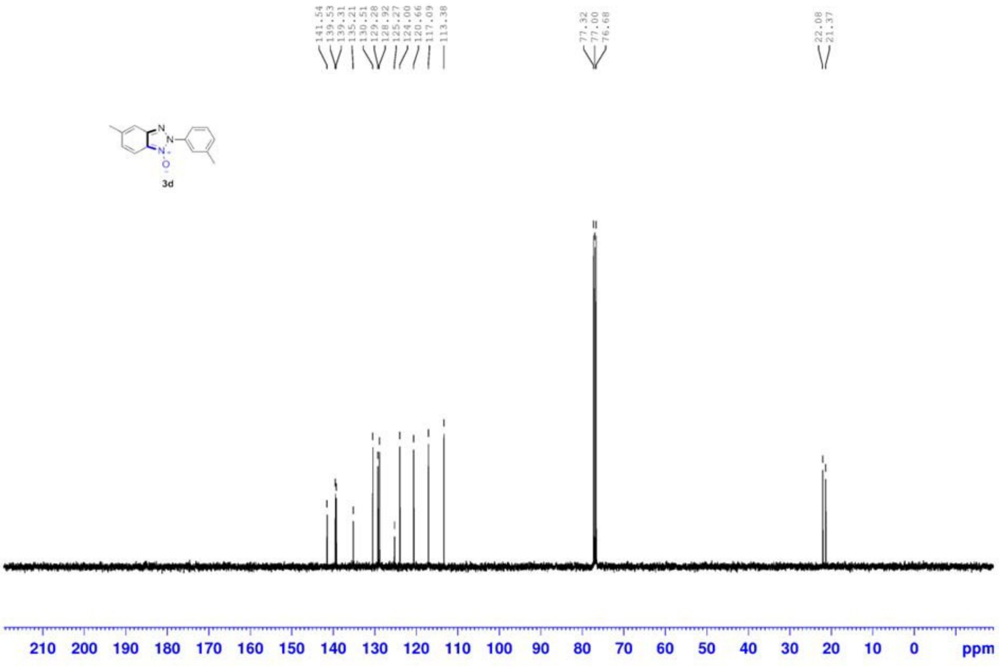

Supplementary Figure 24. ^{13}C NMR spectra of **3d**.

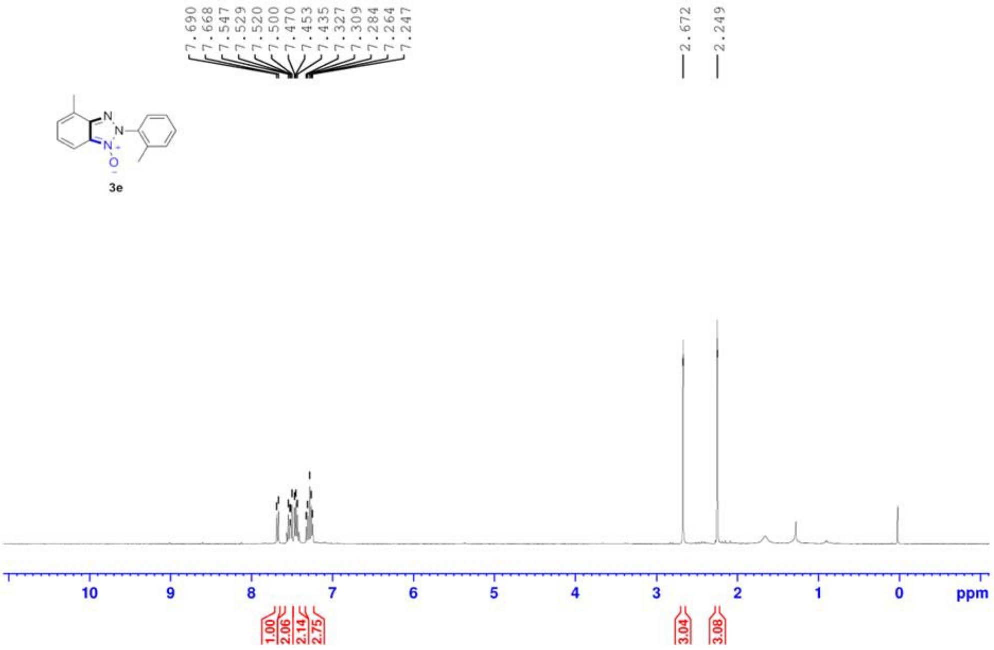

Supplementary Figure 25. ^{1}H NMR spectra of **3e**.

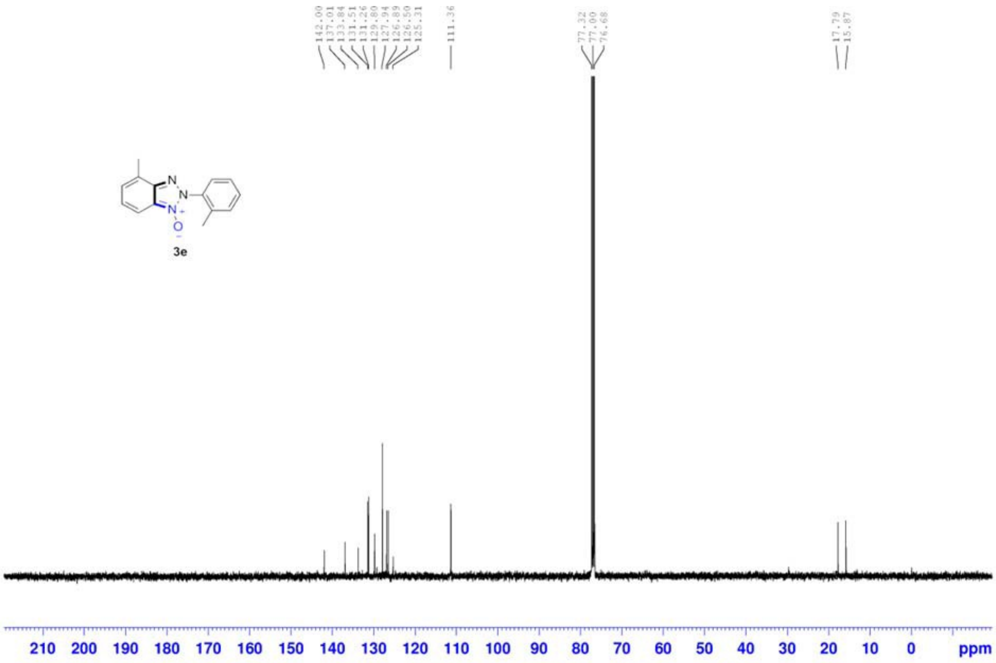

Supplementary Figure 26. ^{13}C NMR spectra of **3e**.

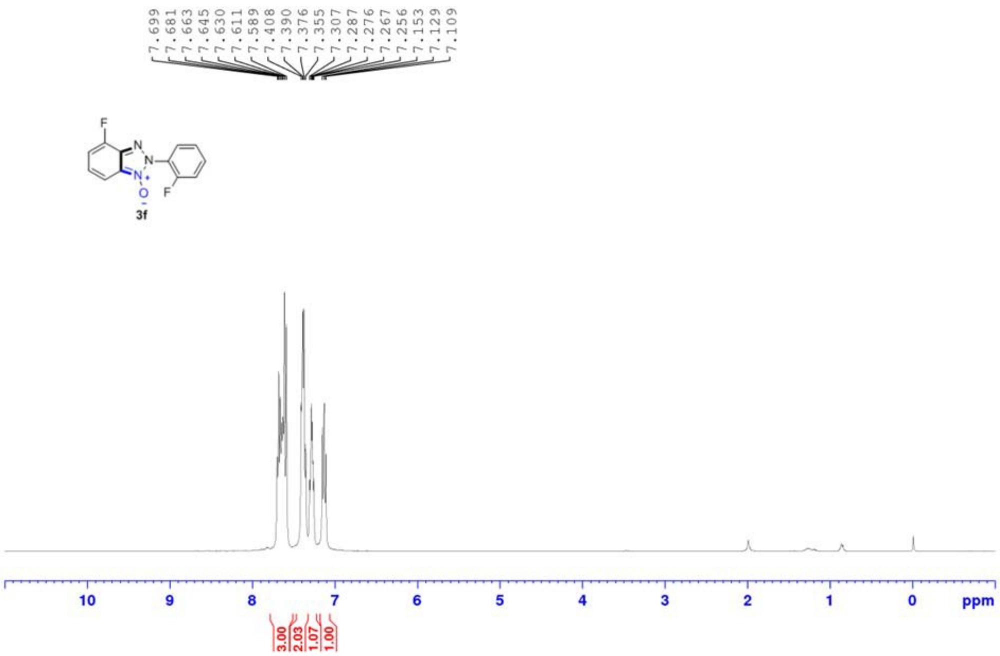

Supplementary Figure 27. ^{1}H NMR spectra of **3f**.

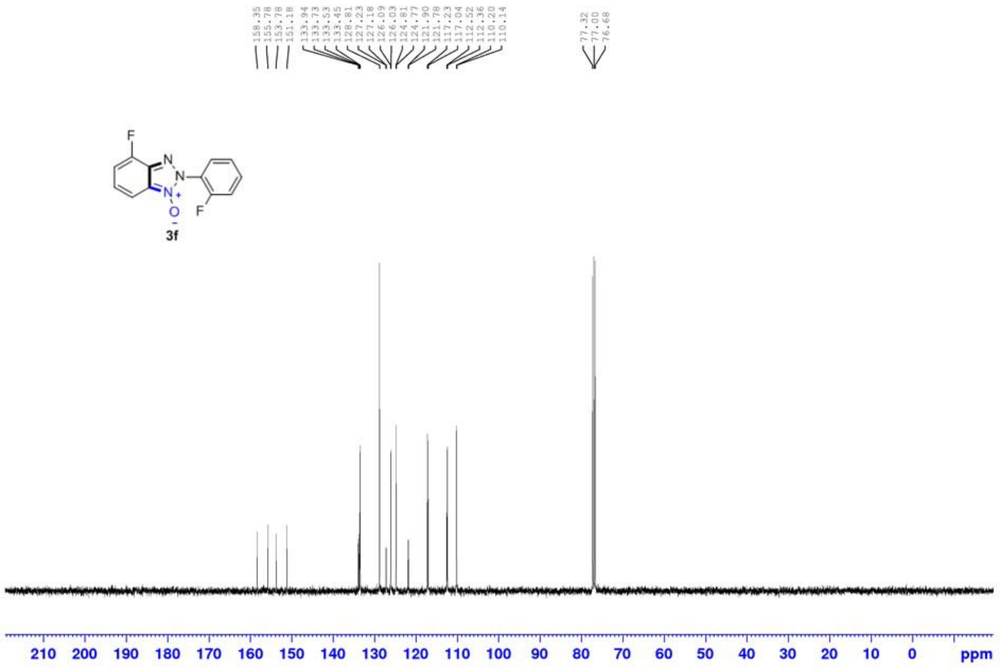

Supplementary Figure 28. ^{13}C NMR spectra of **3f**.

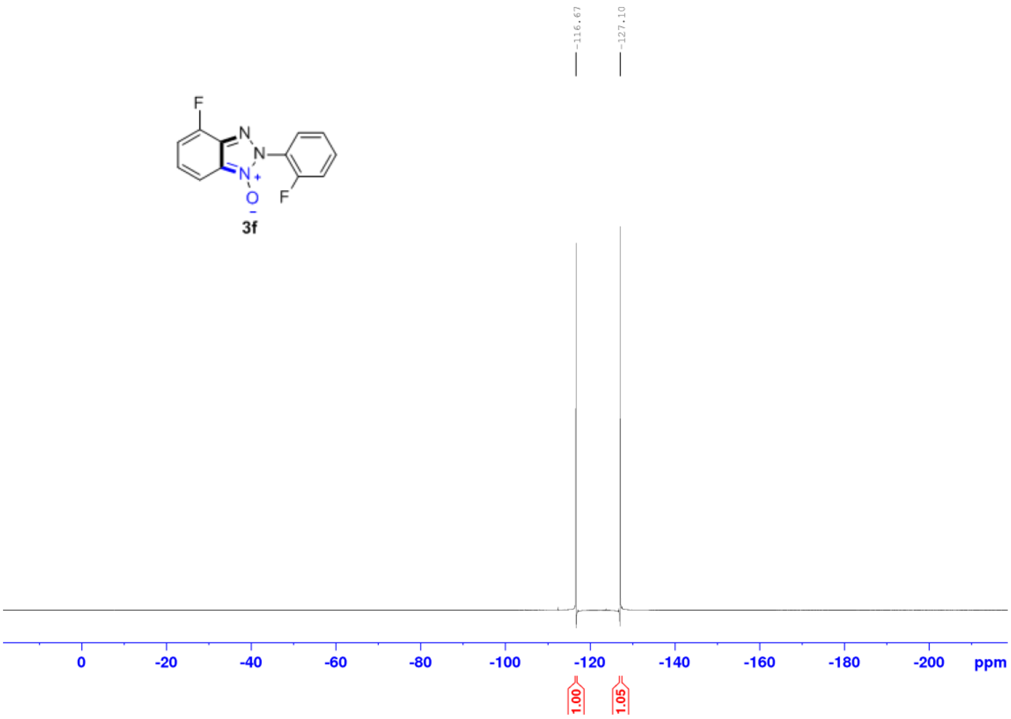

Supplementary Figure 29. ^{19}F NMR spectra of **3f**.

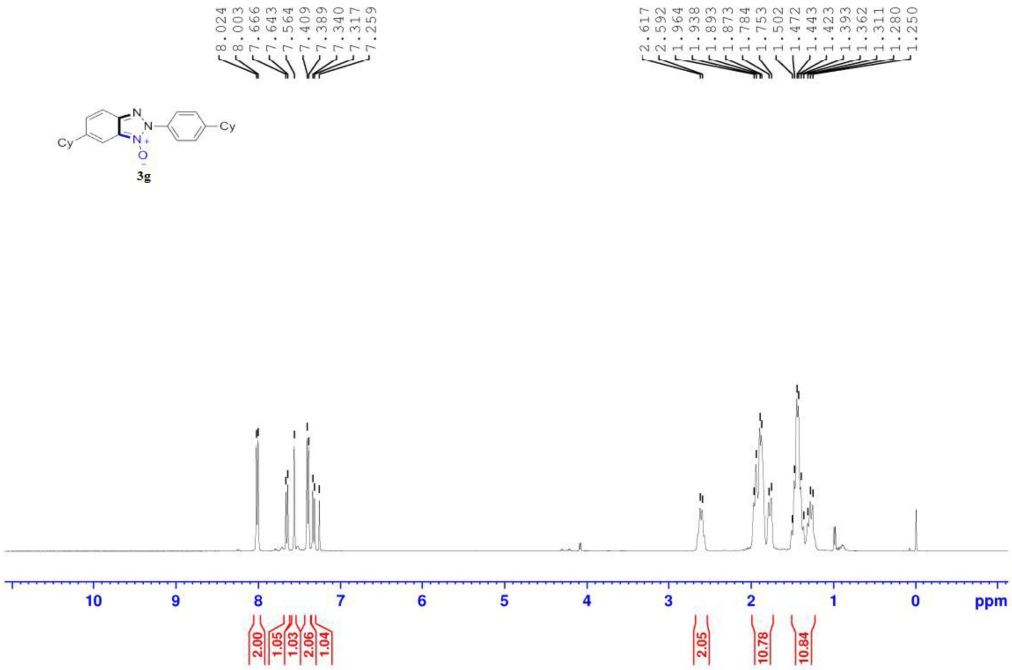

Supplementary Figure 30. ^{1}H NMR spectra of **3g**.

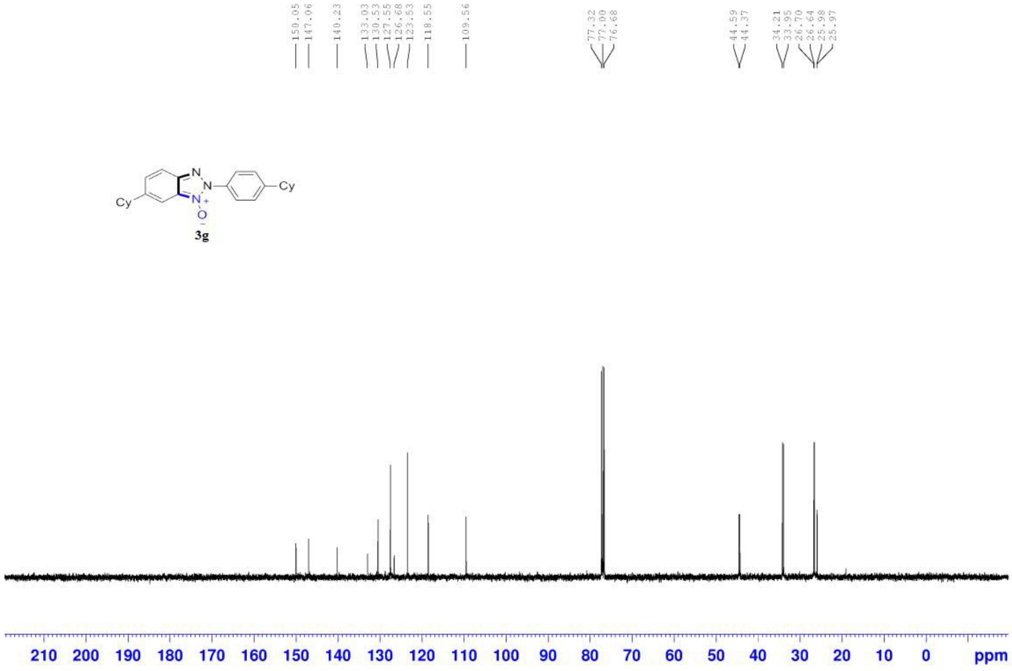

Supplementary Figure 31. ^{13}C NMR spectra of **3g**.

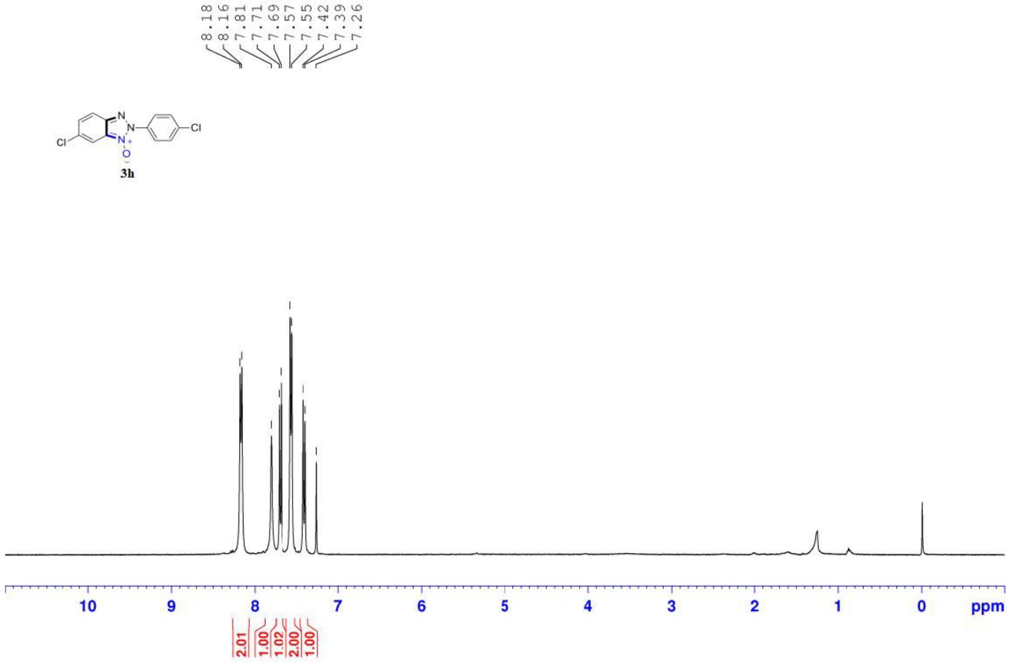

Supplementary Figure 32. ^{1}H NMR spectra of **3h**.

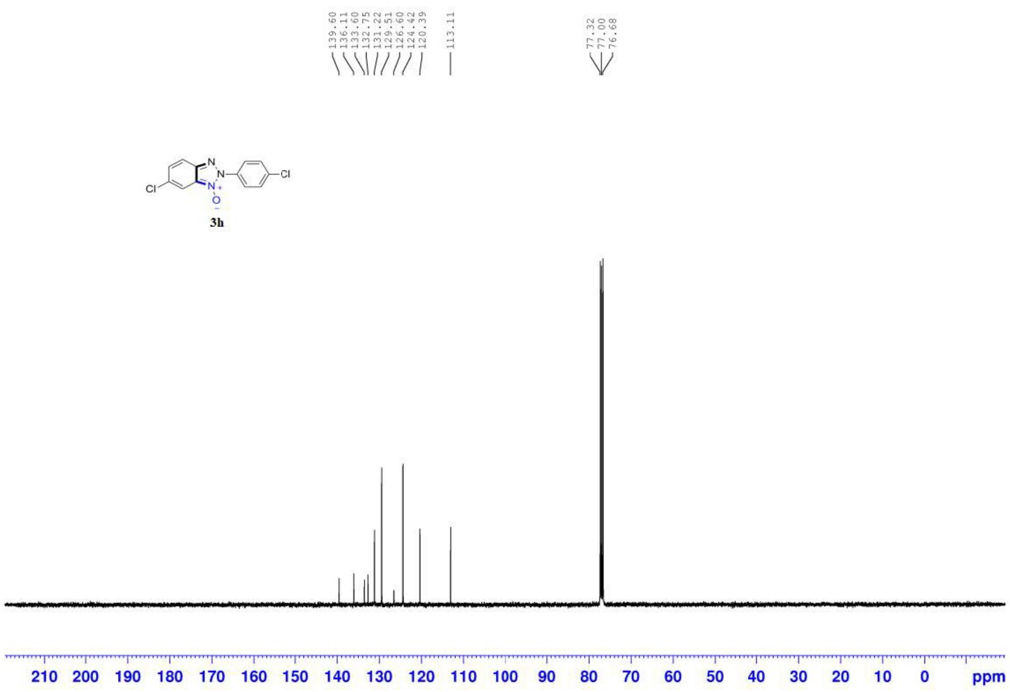

Supplementary Figure 33. ^{13}C NMR spectra of **3h**.

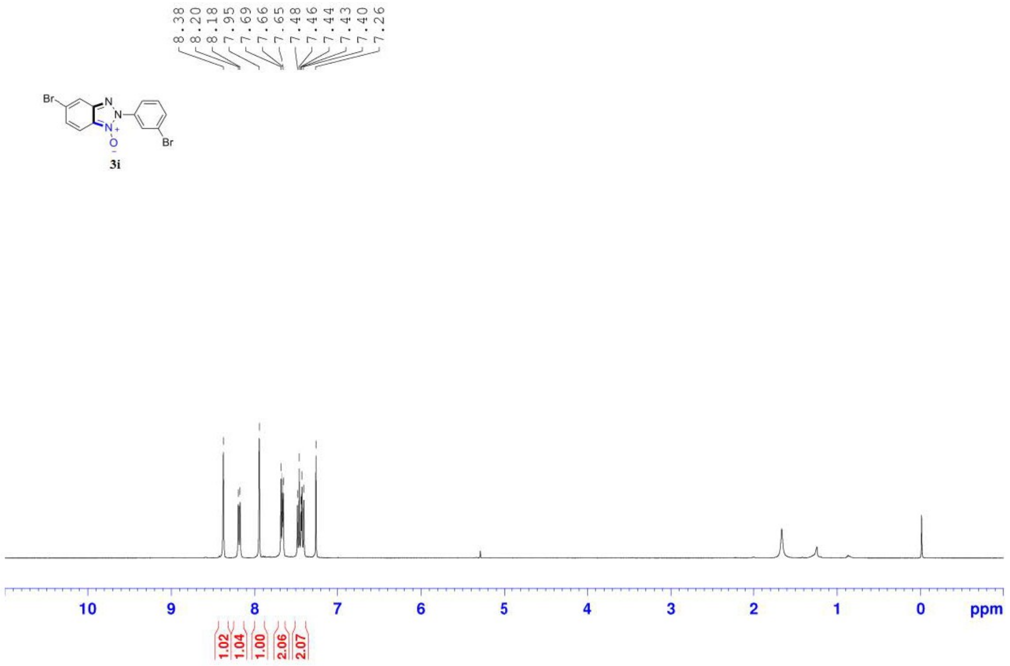

Supplementary Figure 34. ^{1}H NMR spectra of **3i**.

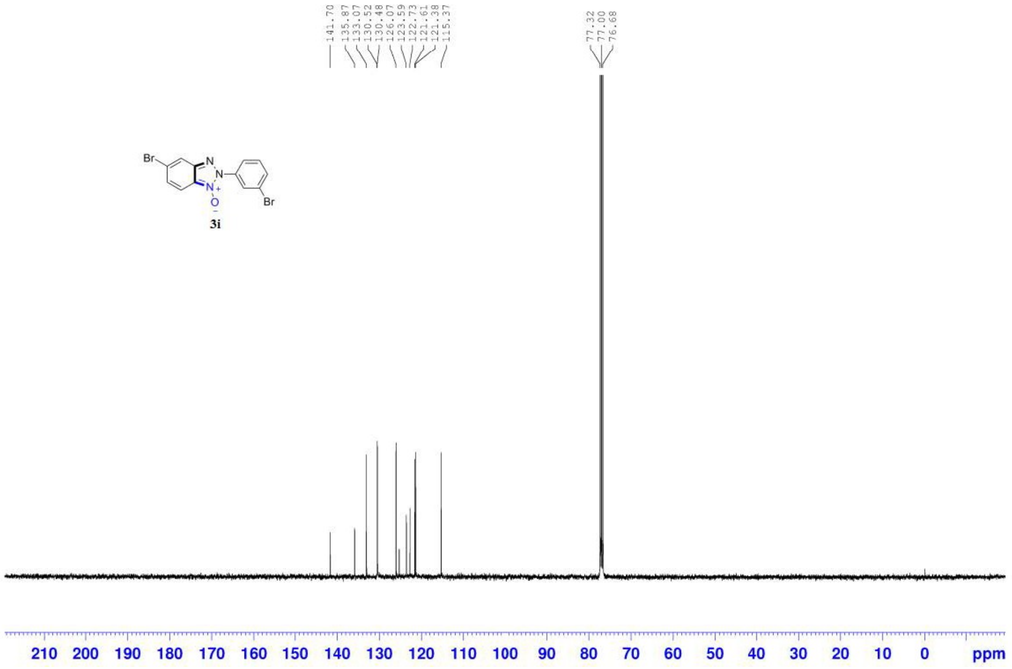

Supplementary Figure 35. ^{13}C NMR spectra of **3i**.

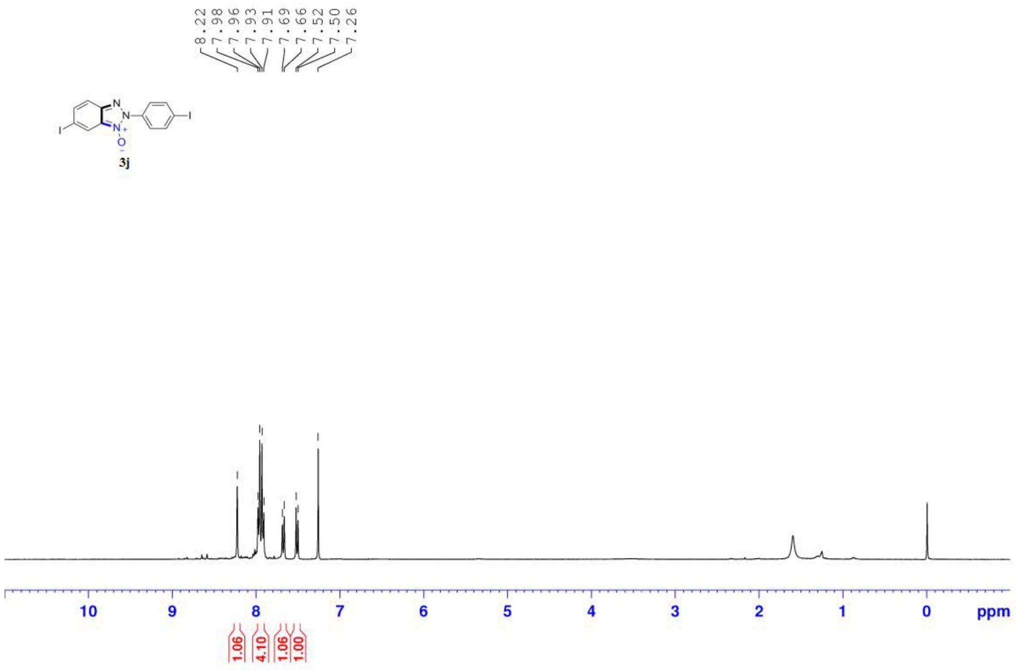

Supplementary Figure 36. ^{1}H NMR spectra of **3j**.

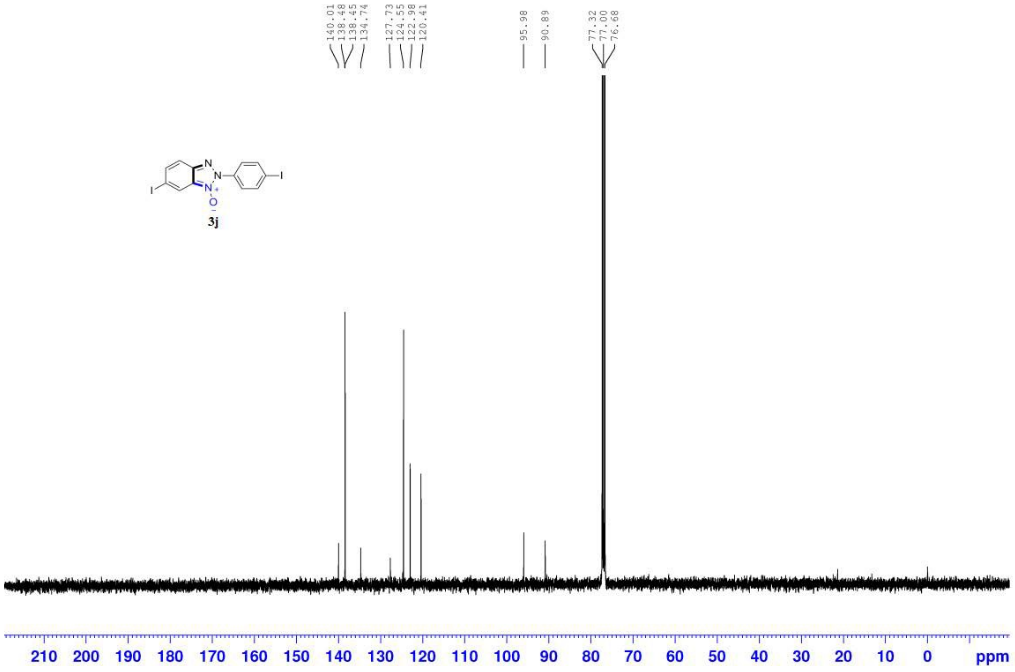

Supplementary Figure 37. ^{13}C NMR spectra of **3j**.

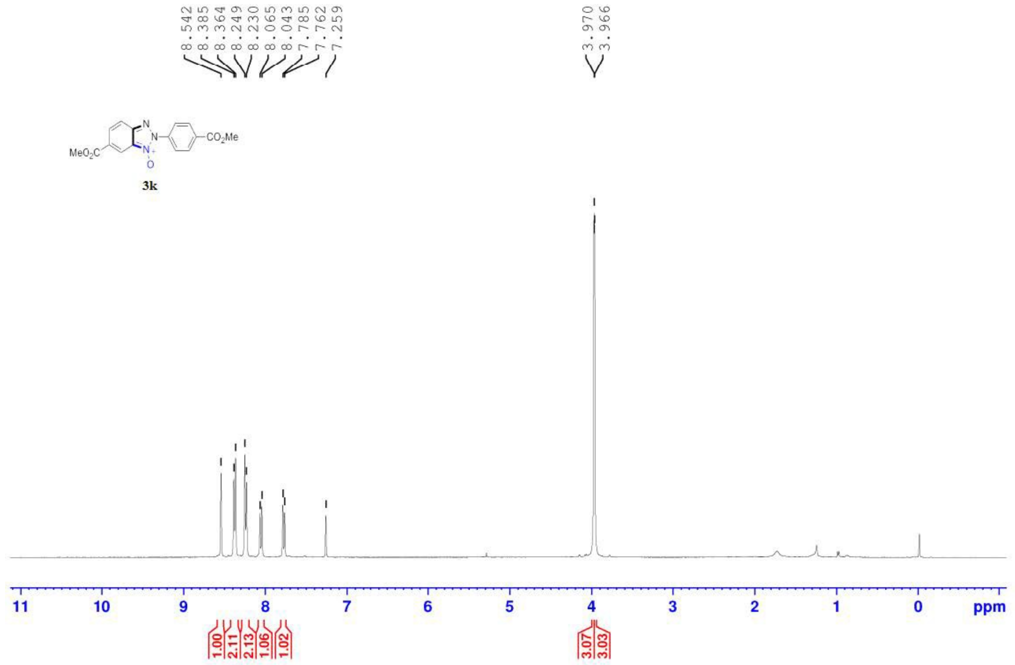

Supplementary Figure 38. ^{1}H NMR spectra of **3k**.

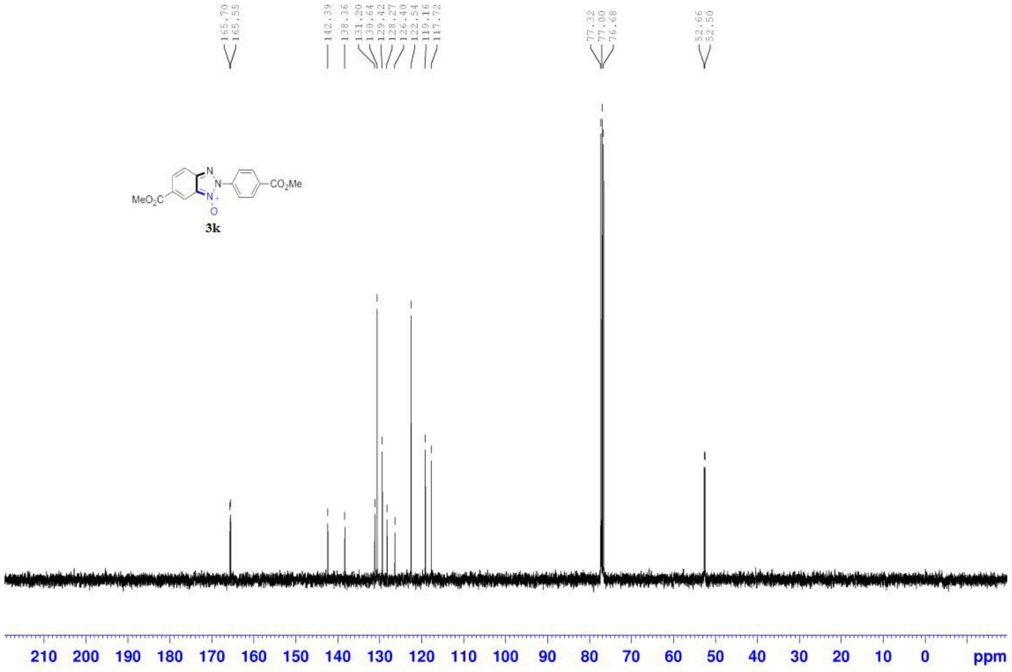

Supplementary Figure 39. ^{13}C NMR spectra of **3k**.

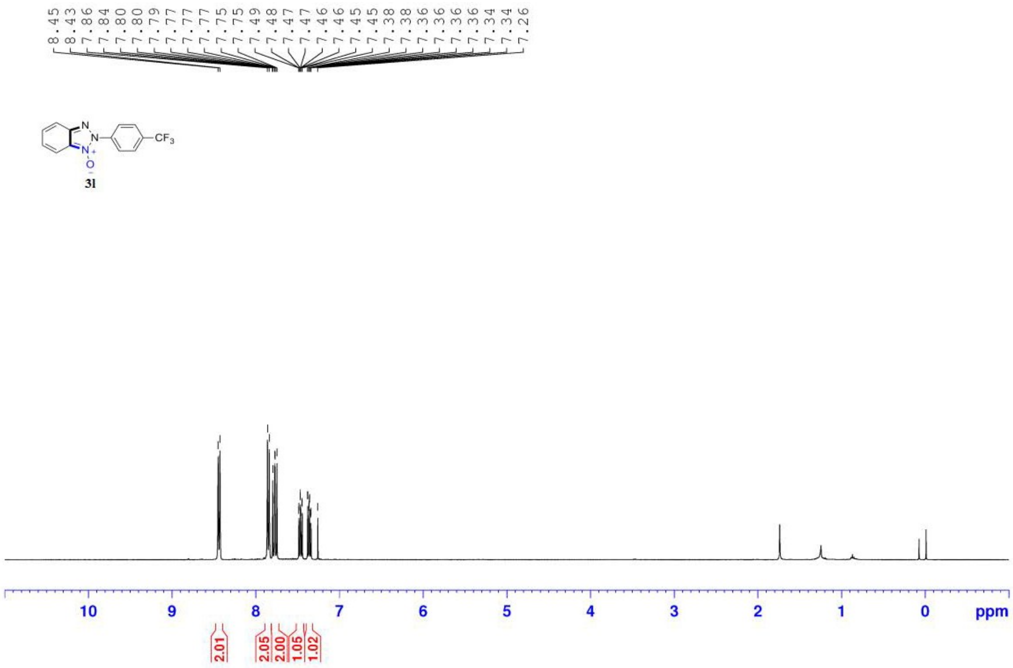

Supplementary Figure 40. ^{1}H NMR spectra of **3l**.

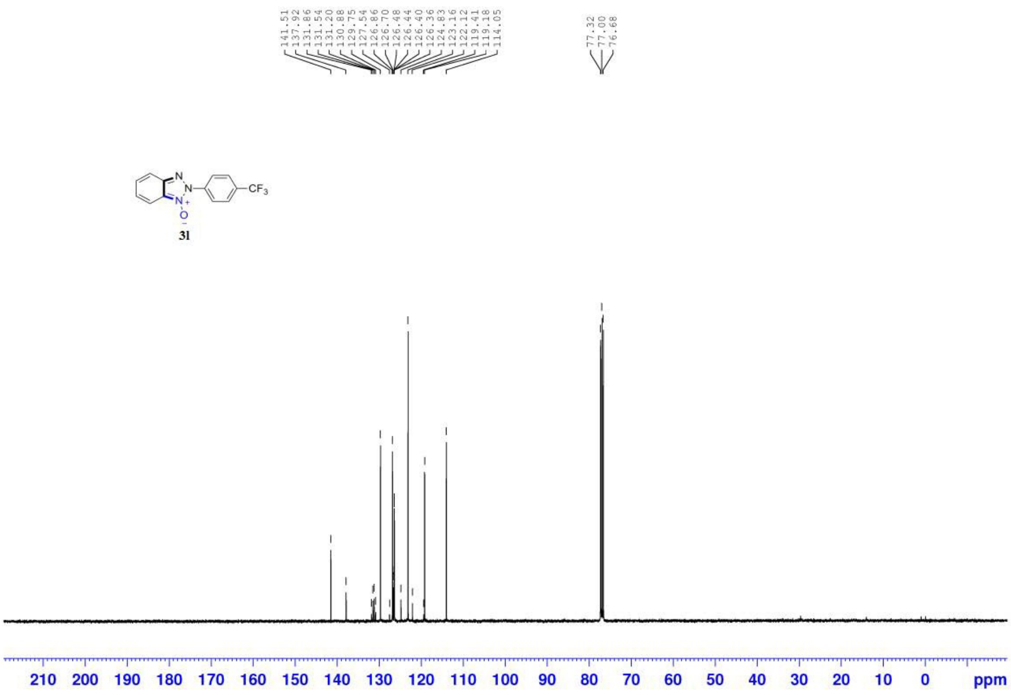

Supplementary Figure 41. ^{13}C NMR spectra of **3l**.

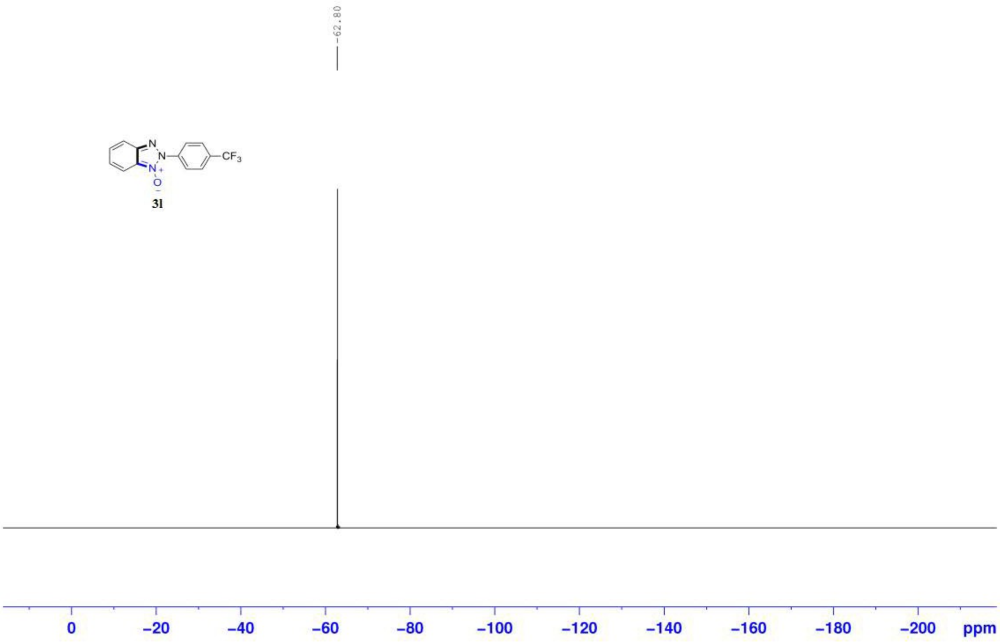

Supplementary Figure 42. ^{19}F NMR spectra of **3l**.

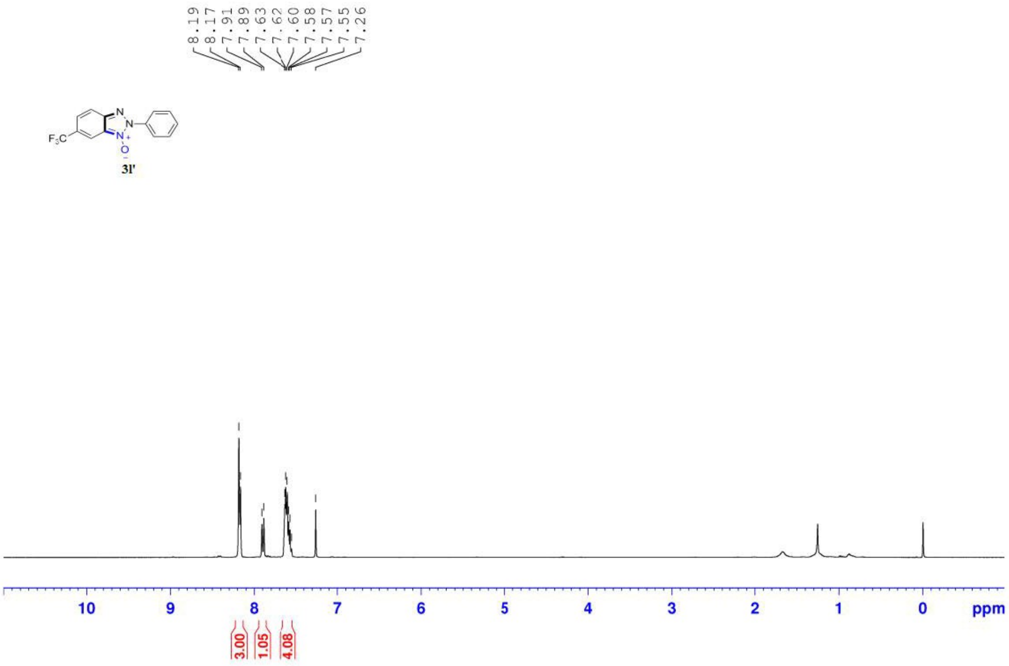

Supplementary Figure 43. ^{1}H NMR spectra of **3l'**.

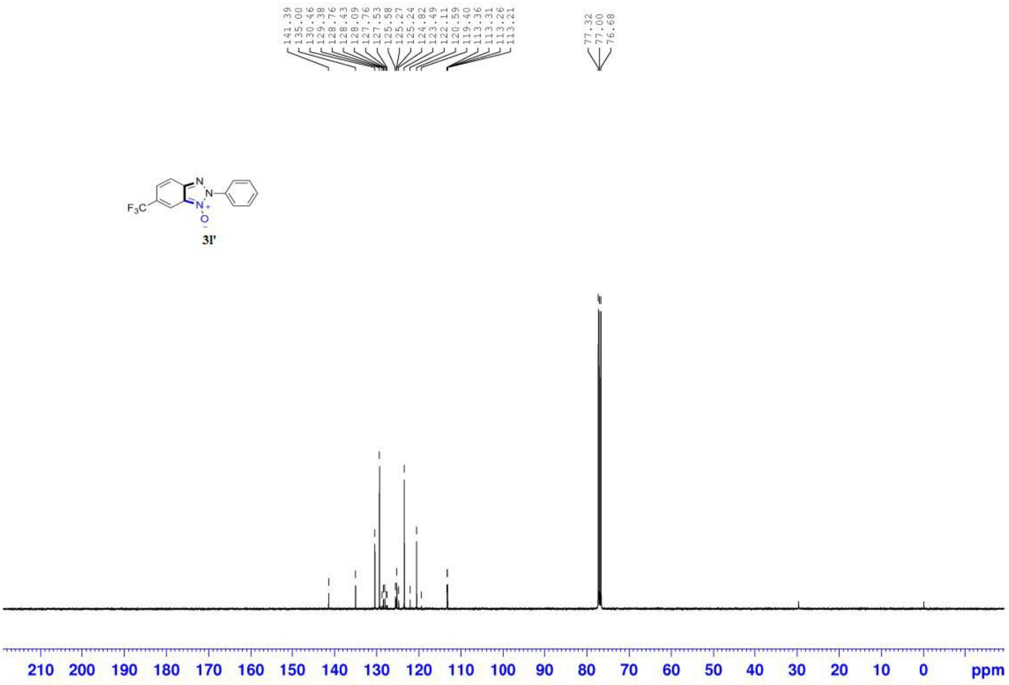

Supplementary Figure 44. ^{13}C NMR spectra of **3l'**.

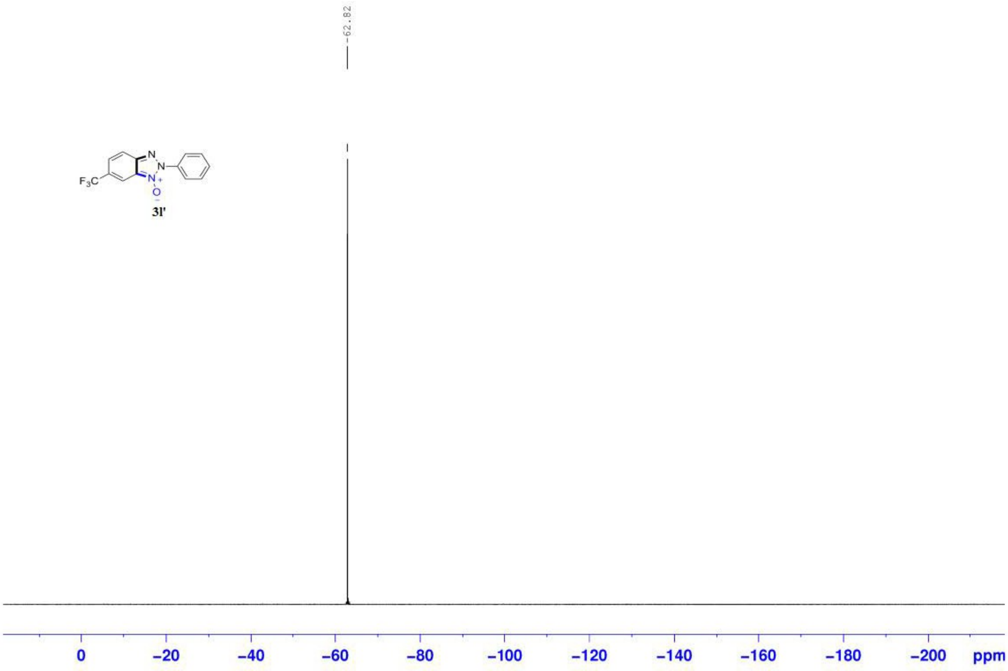

Supplementary Figure 45. ^{19}F NMR spectra of **3l'**.

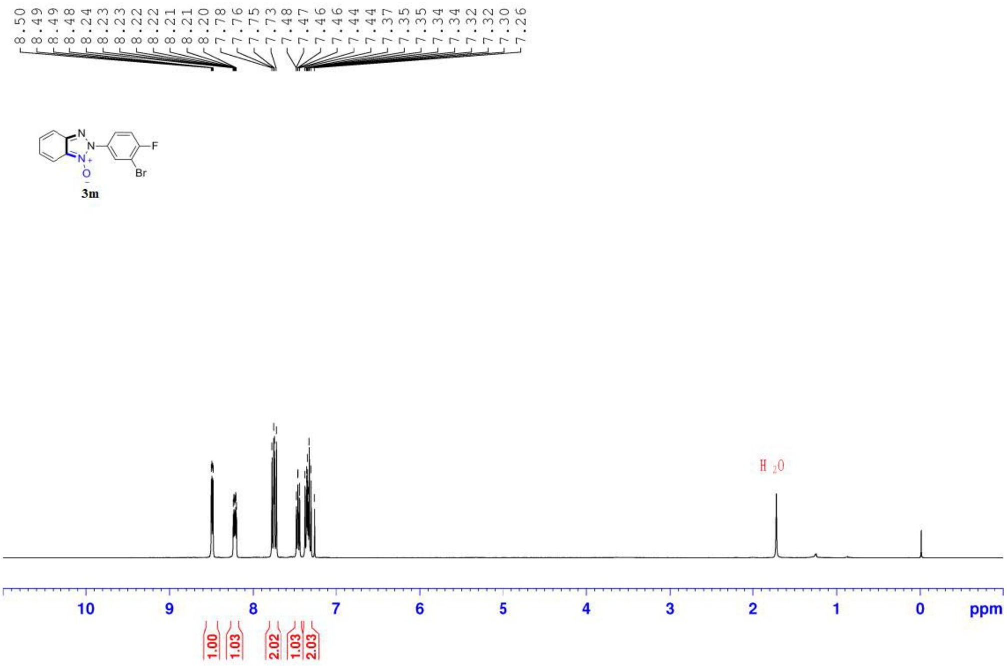

Supplementary Figure 46. ^{1}H NMR spectra of **3m**.

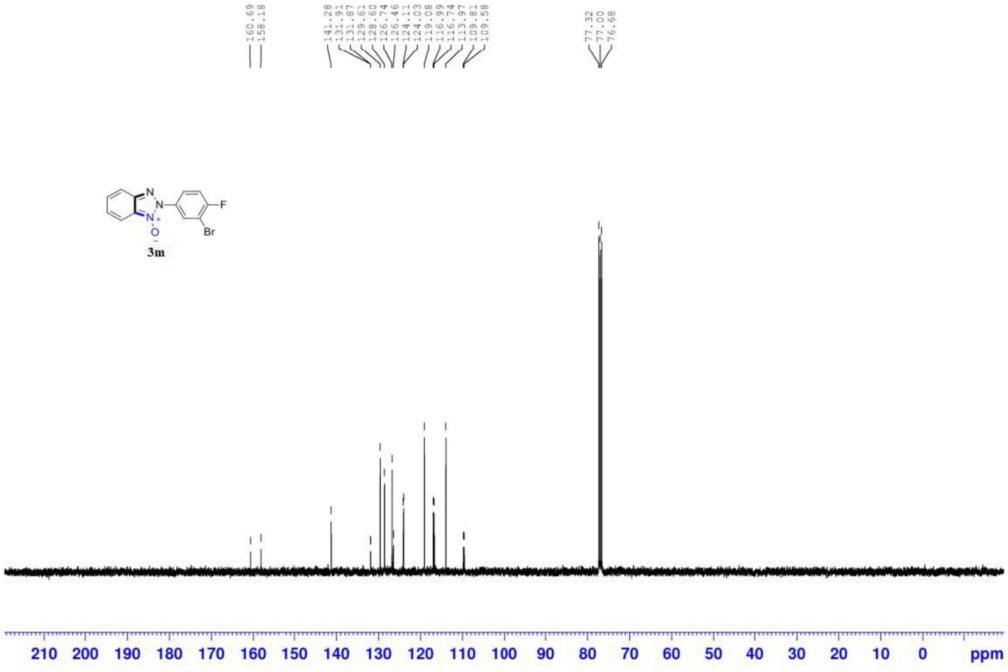

Supplementary Figure 47. ^{13}C NMR spectra of **3m**.

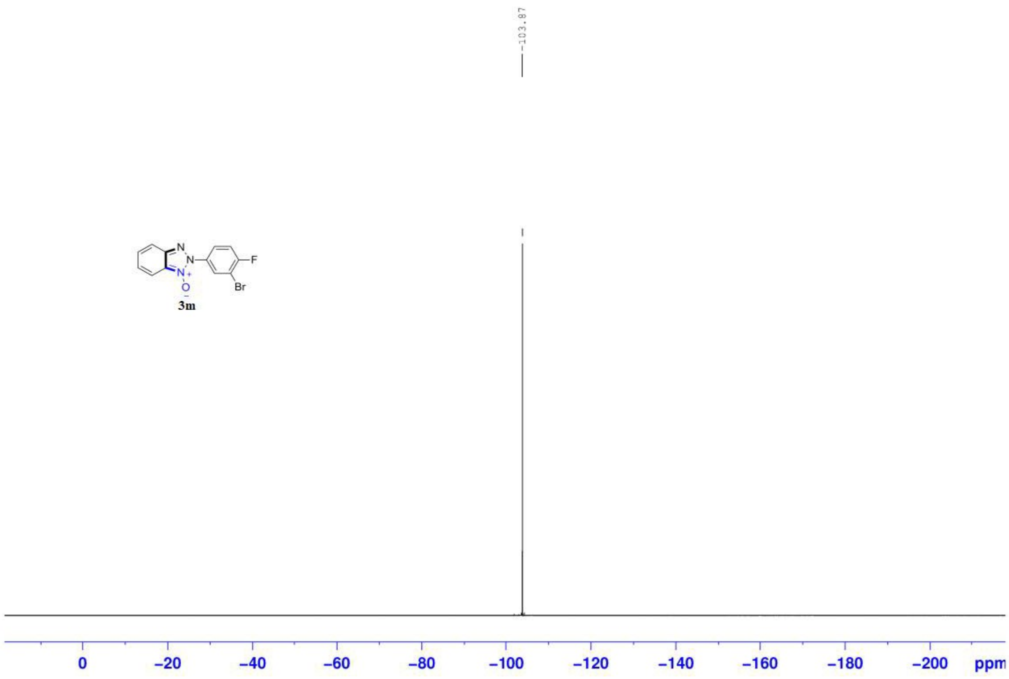

Supplementary Figure 48. ^{19}F NMR spectra of **3m**.

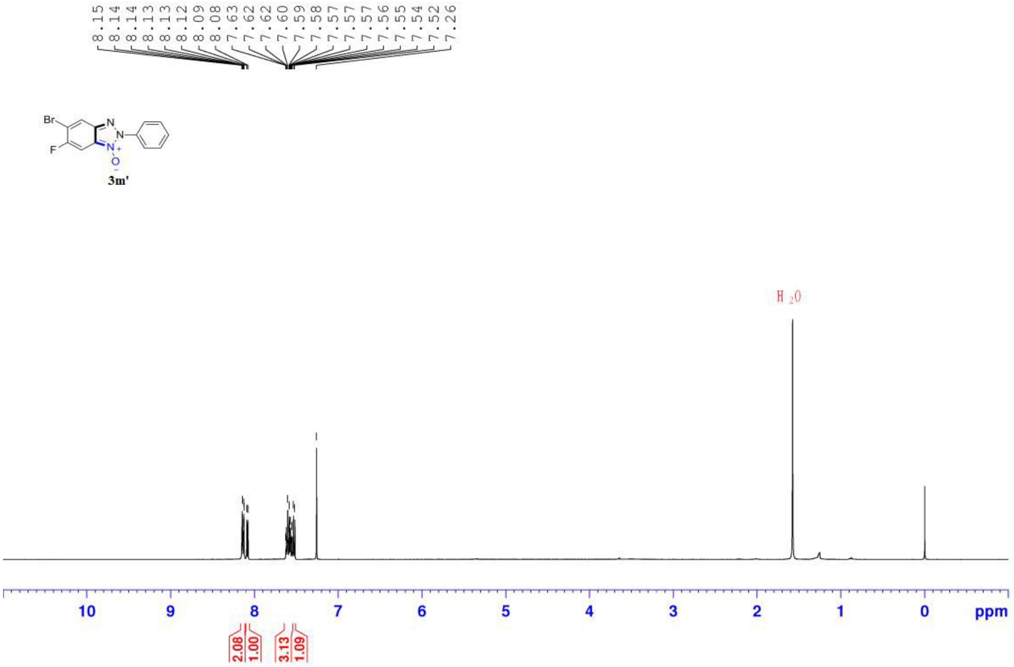

Supplementary Figure 49. ^{1}H NMR spectra of **3m'**.

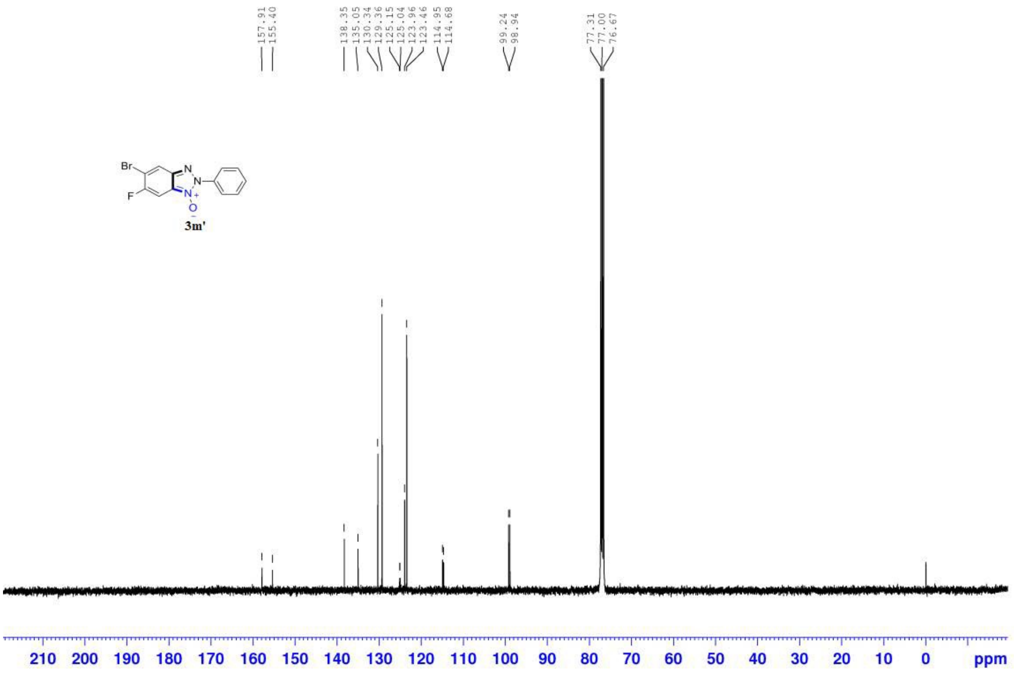

Supplementary Figure 50. ^{13}C NMR spectra of **3m'**.

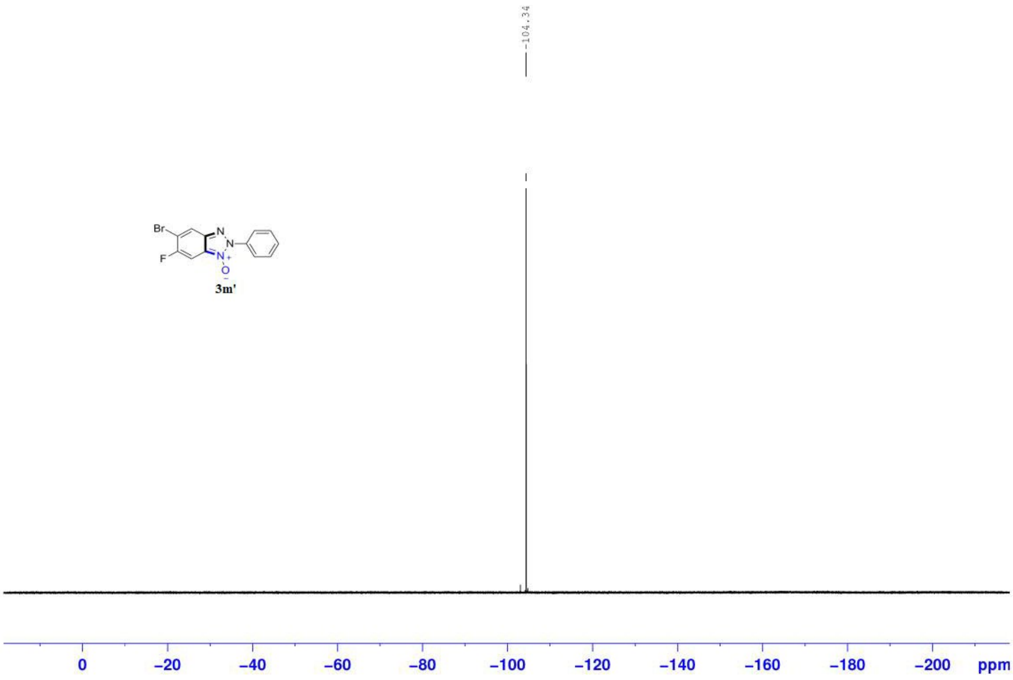

Supplementary Figure 51. ^{19}F NMR spectra of **3m’**.

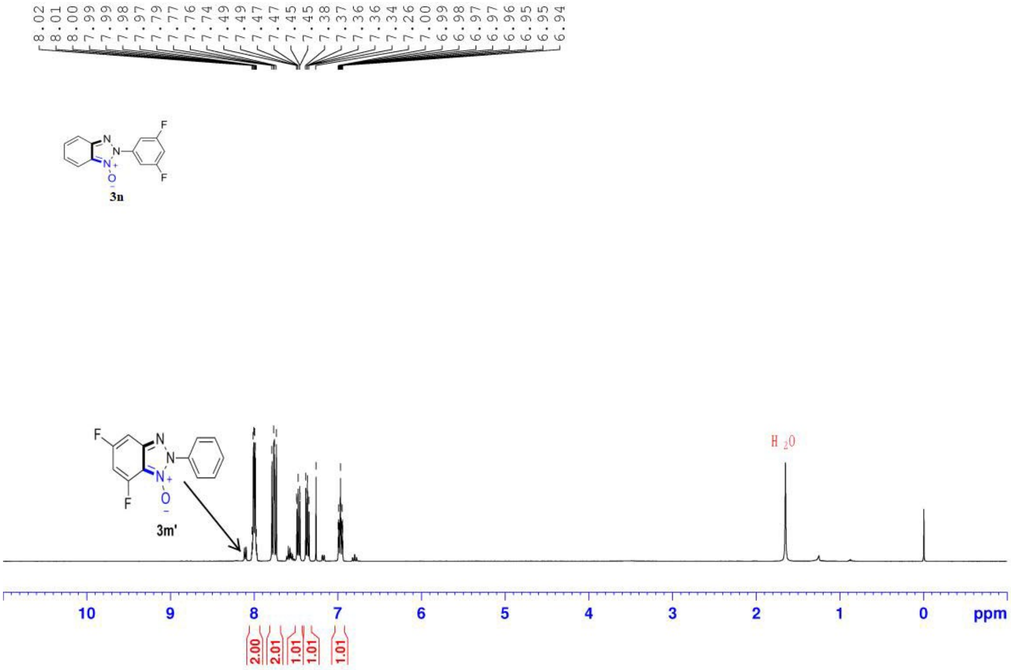

Supplementary Figure 52. ^{1}H NMR spectra of **3n**.

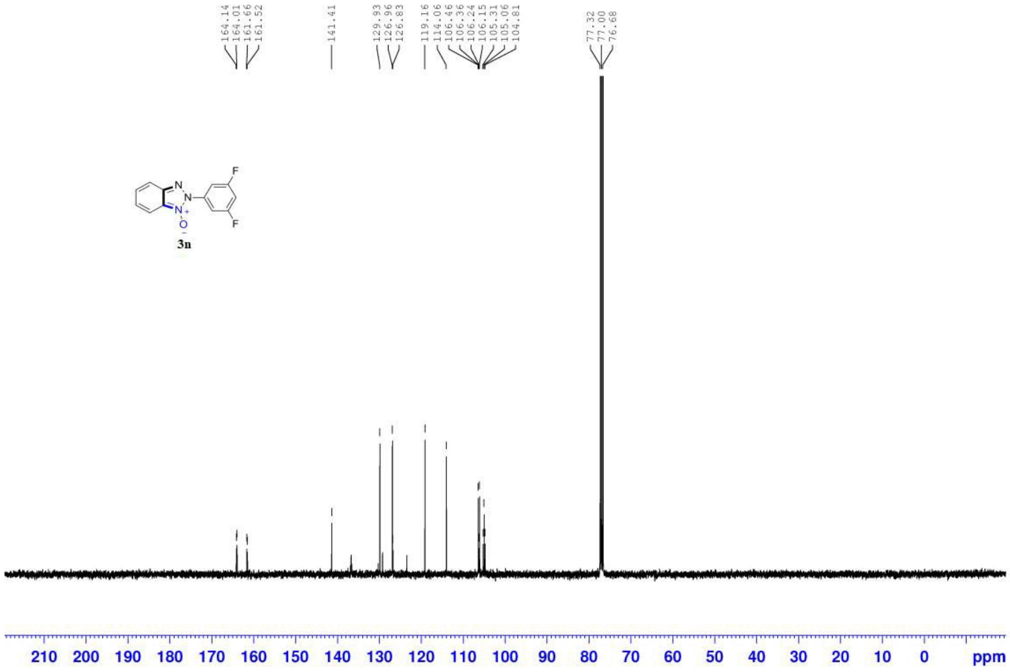

Supplementary Figure 53. ^{13}C NMR spectra of **3n**.

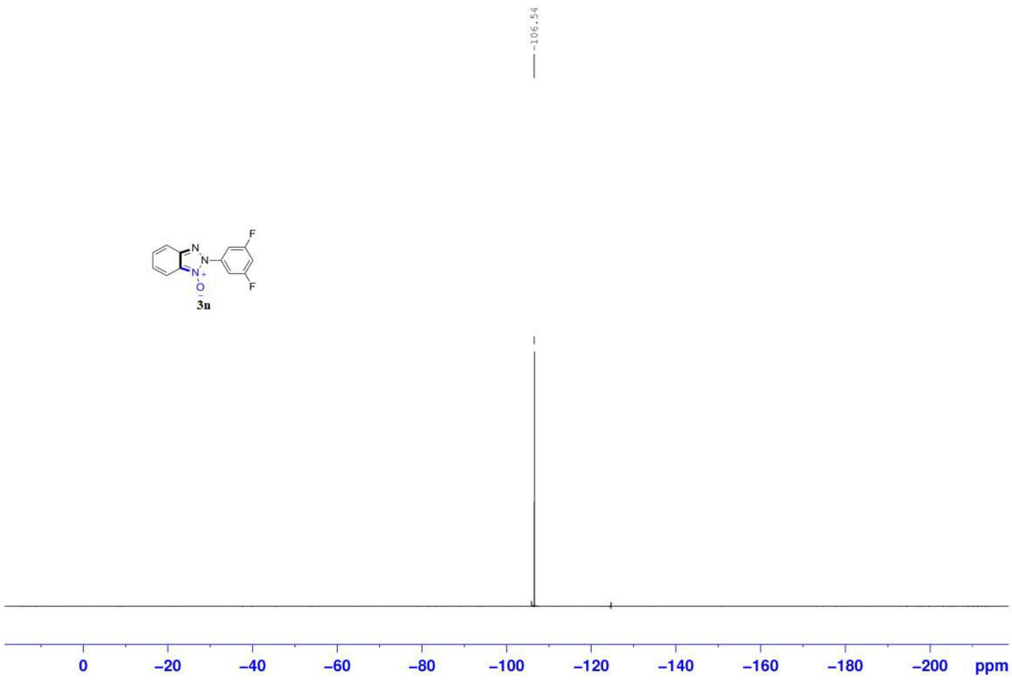

Supplementary Figure 54. ^{19}F NMR spectra of **3n**.

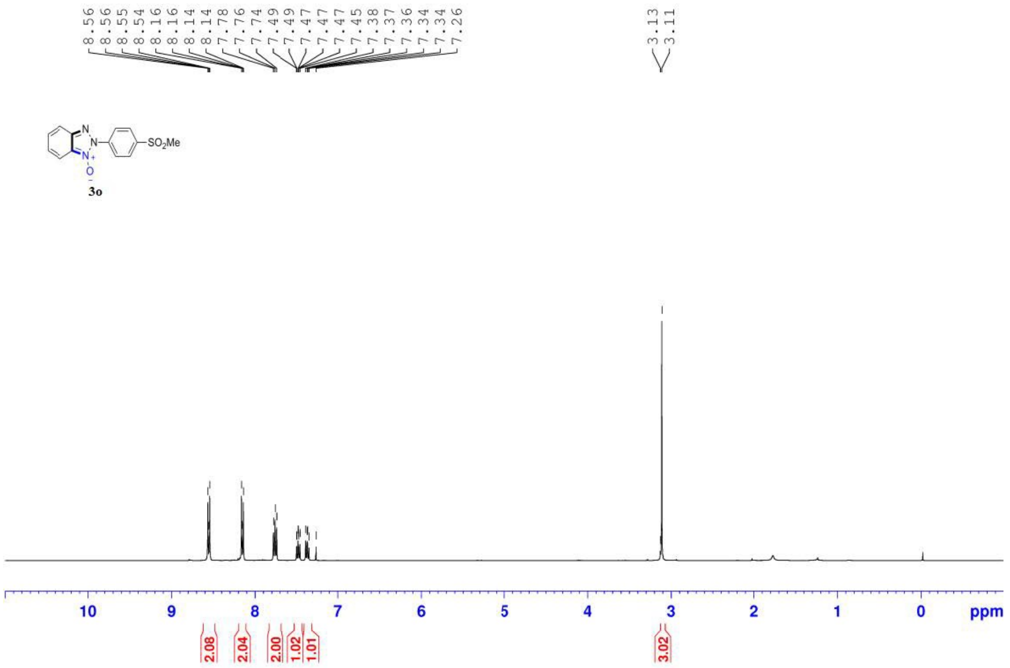

Supplementary Figure 55. ^{1}H NMR spectra of **3o**.

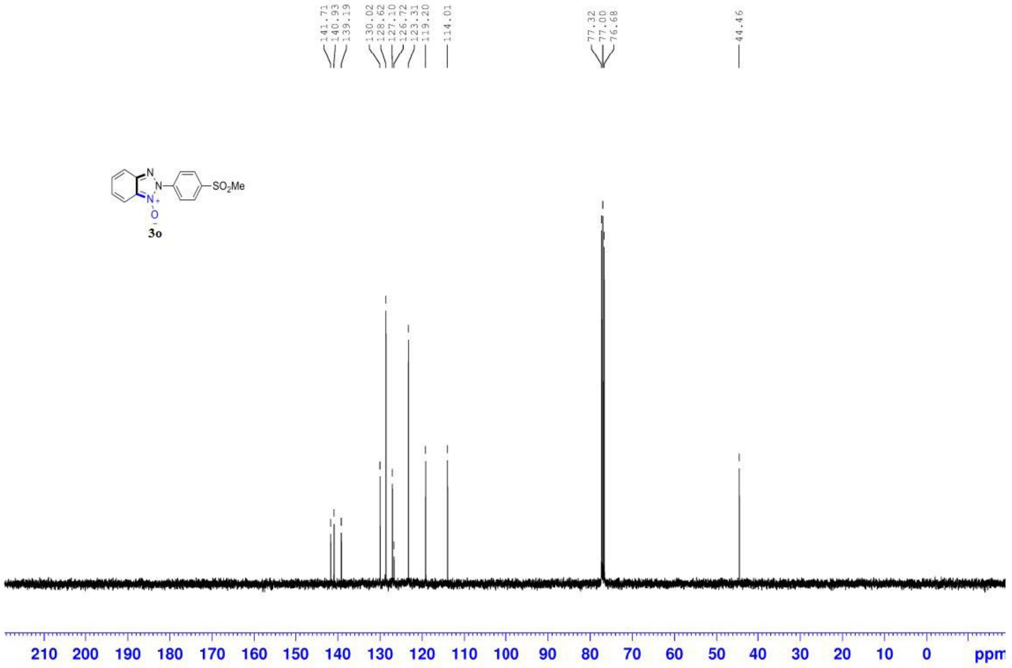

Supplementary Figure 56. ^{13}C NMR spectra of **3o**.

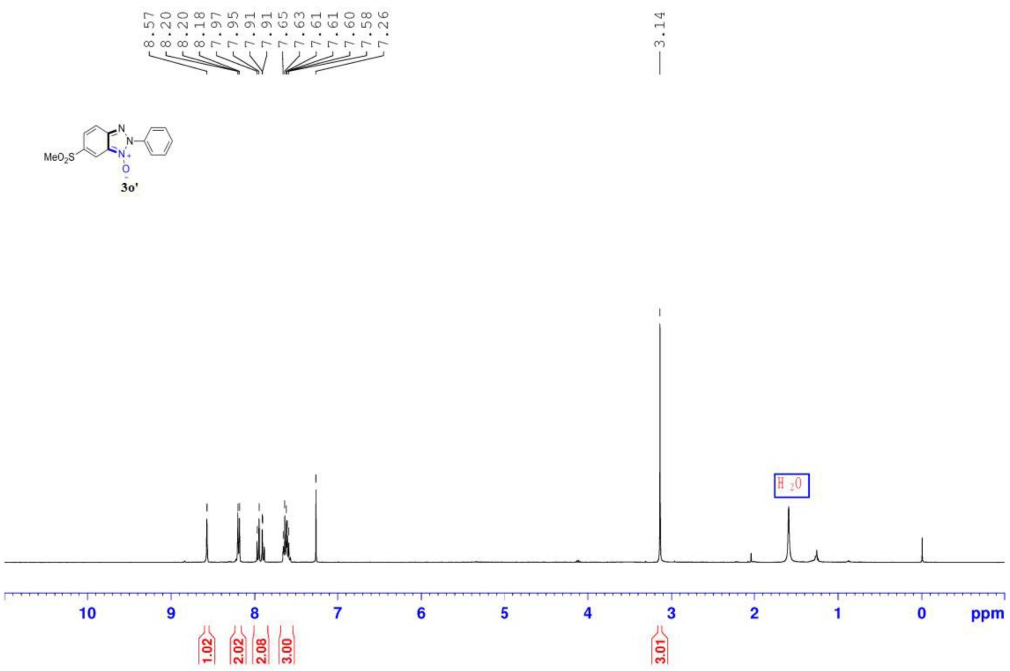

Supplementary Figure 57. ^{1}H NMR spectra of **3o'**.

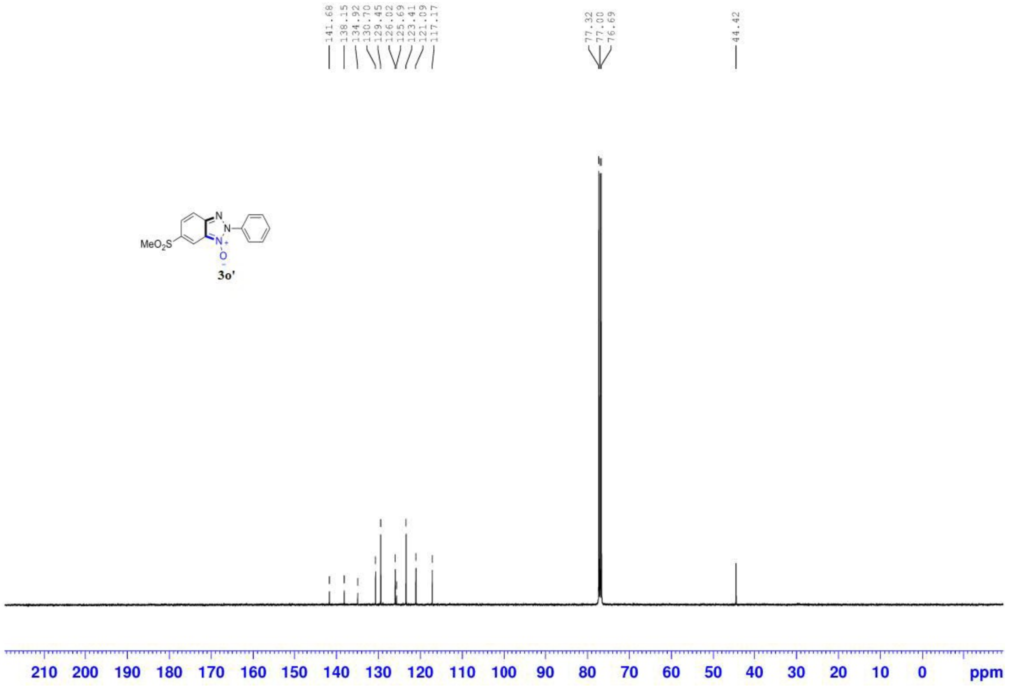

Supplementary Figure 58. ^{13}C NMR spectra of **3o'**.

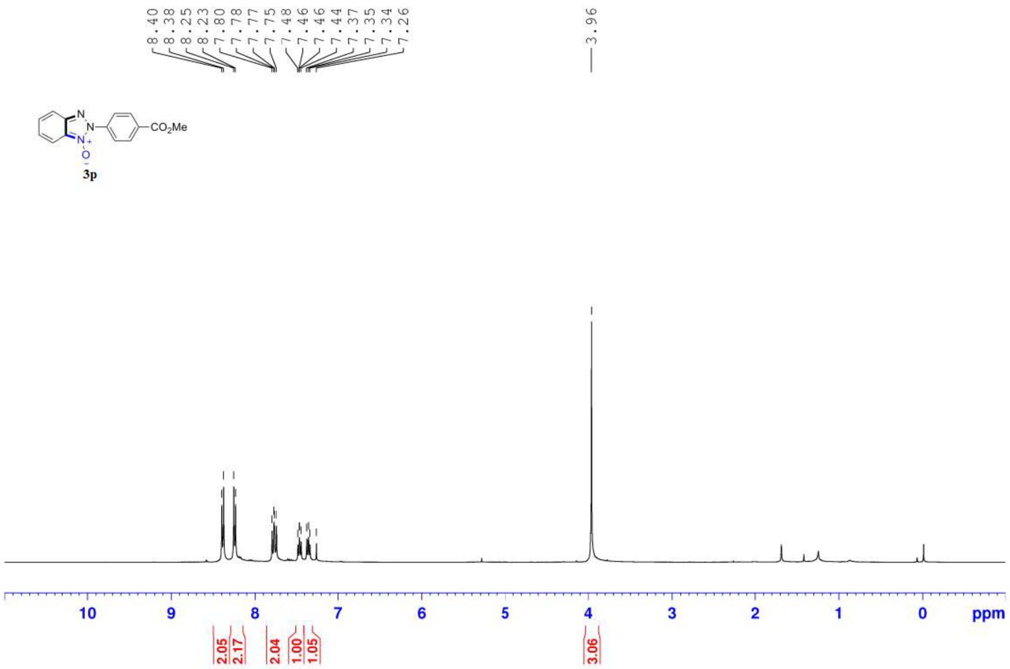

Supplementary Figure 59. ^{1}H NMR spectra of **3p**.

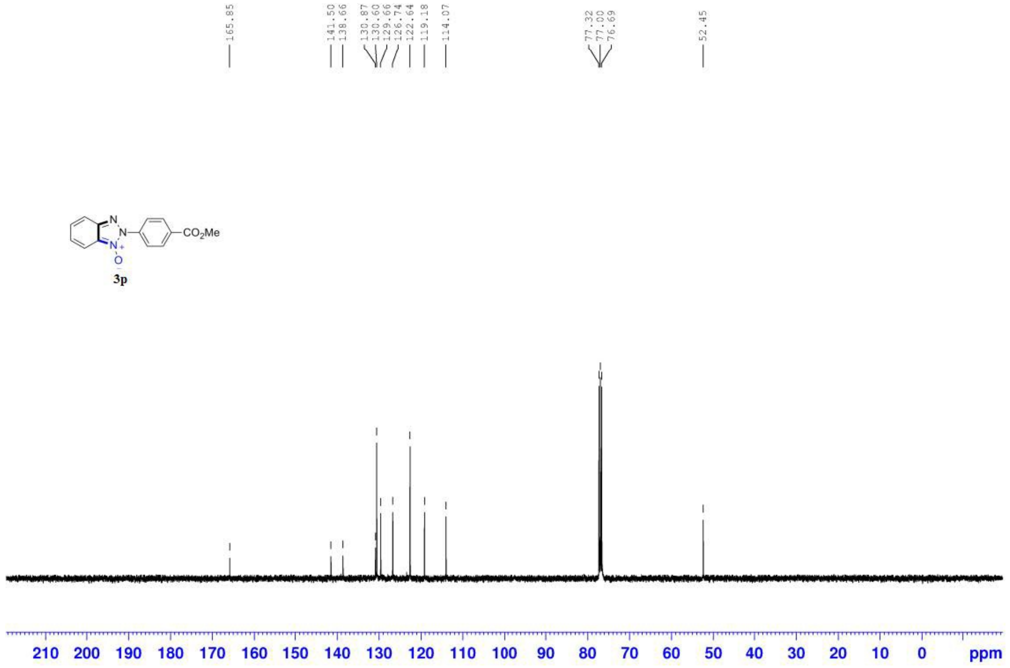

Supplementary Figure 60. ^{13}C NMR spectra of **3p**.

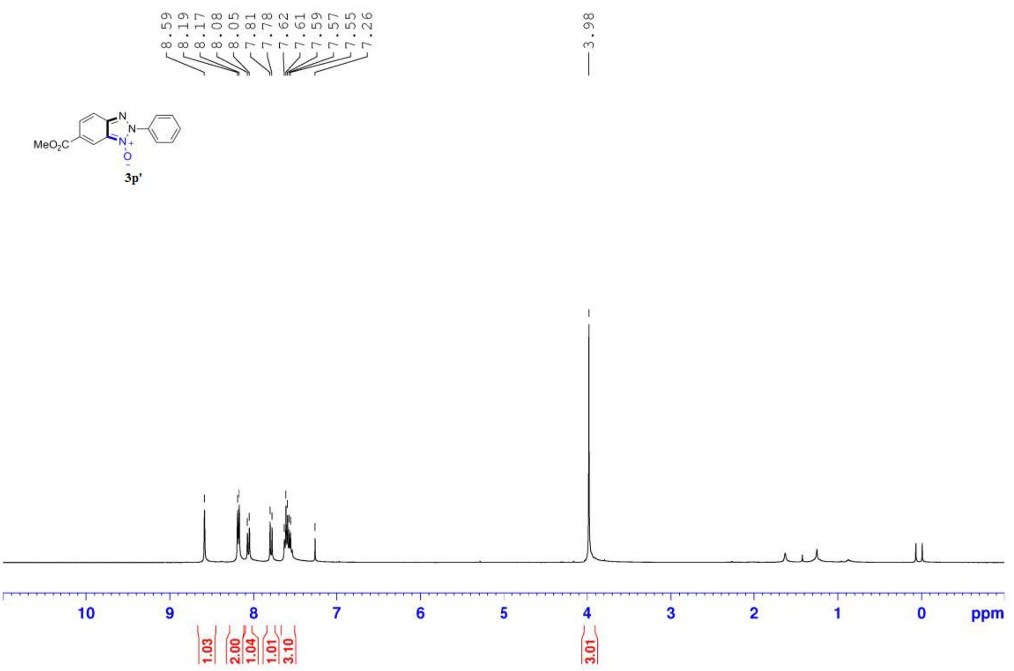

Supplementary Figure 61. ^{1}H NMR spectra of **3p’**.

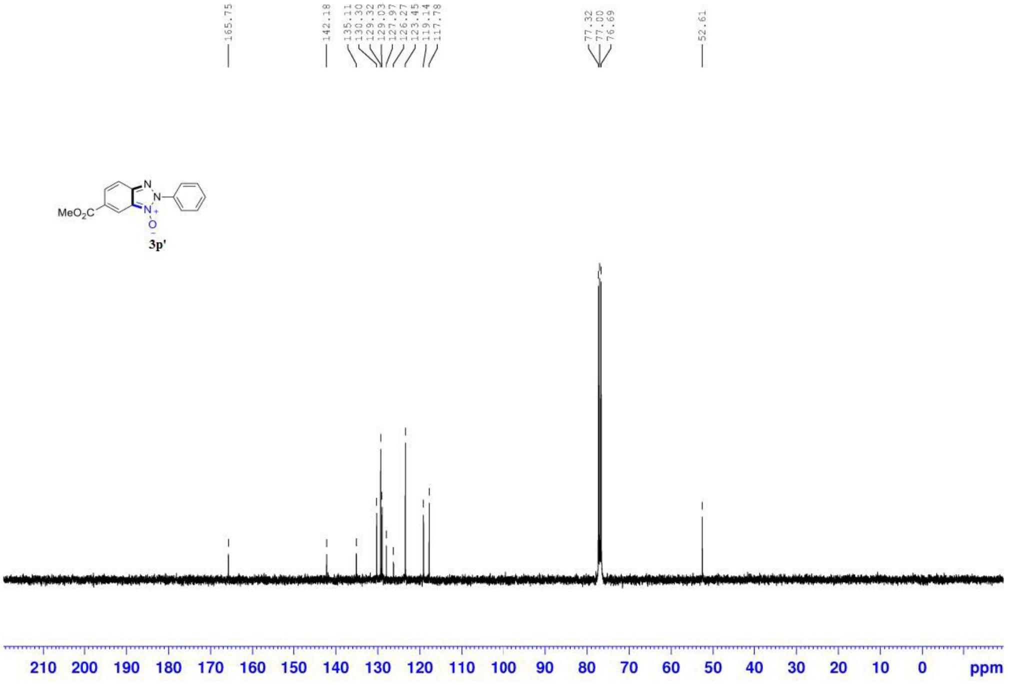

Supplementary Figure 62. ^{13}C NMR spectra of **3p’**.

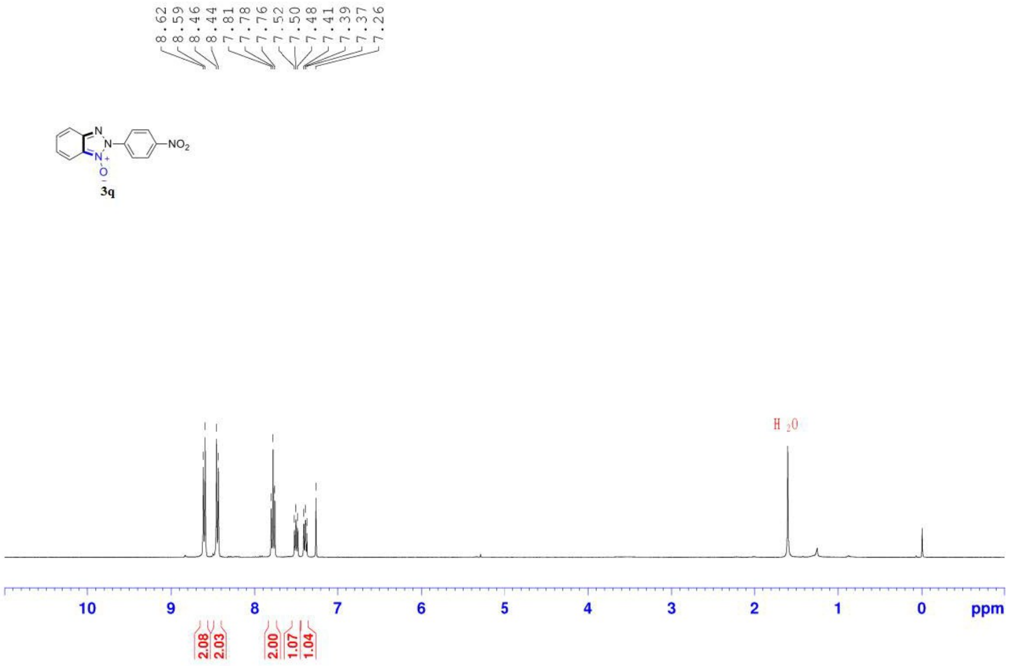

Supplementary Figure 63. ^{1}H NMR spectra of **3q**.

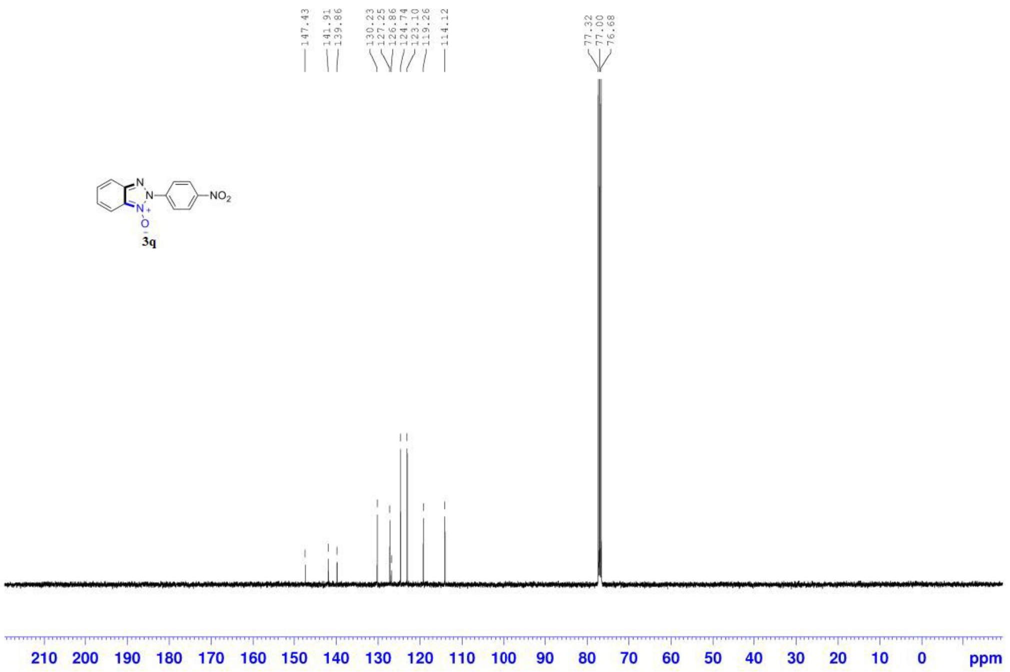

Supplementary Figure 64. ^{13}C NMR spectra of **3q**.

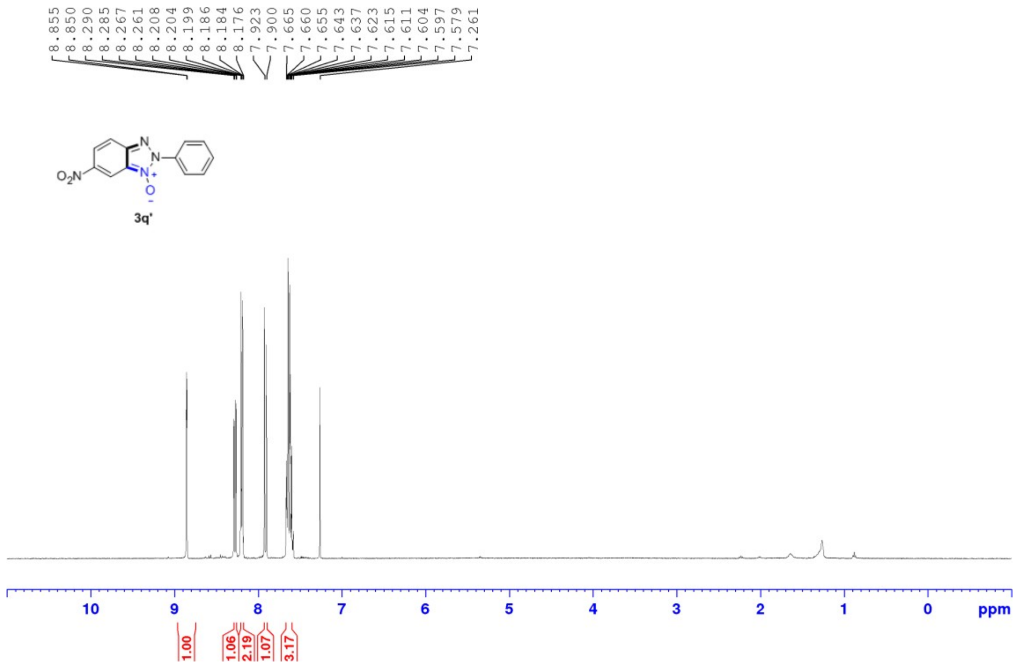

Supplementary Figure 65. ^{1}H NMR spectra of **3q'**.

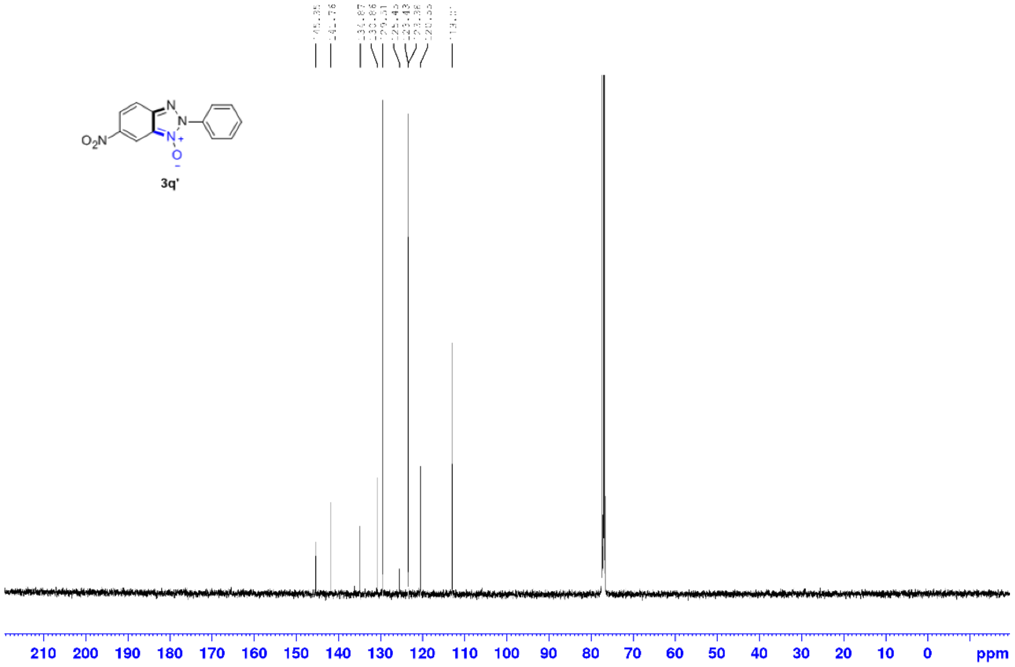

Supplementary Figure 66. ^{13}C NMR spectra of **3q'**.

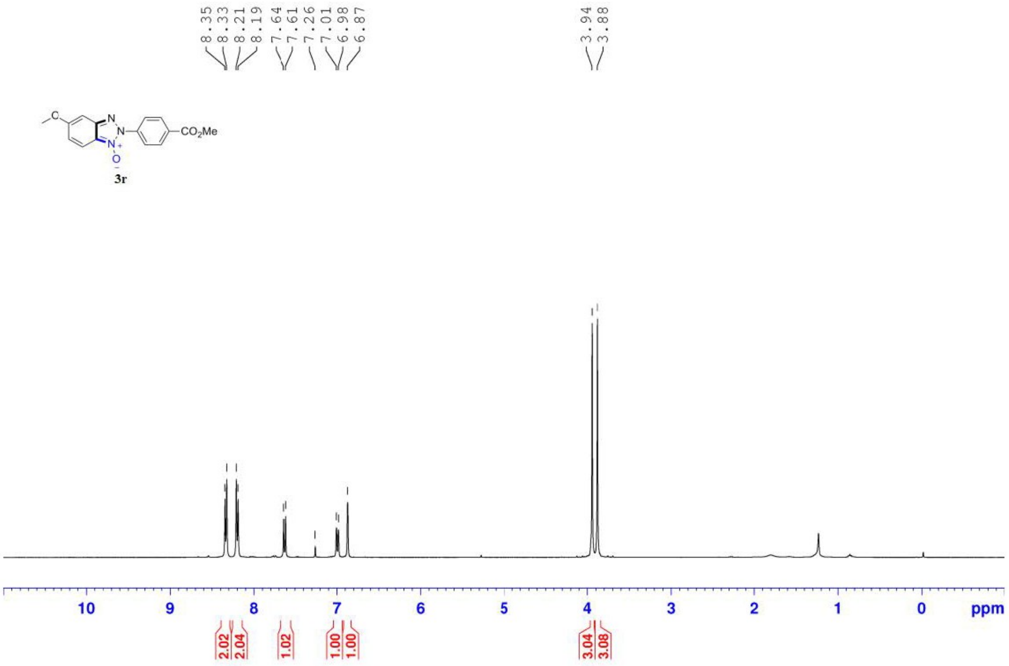

Supplementary Figure 67. ^{1}H NMR spectra of **3r**.

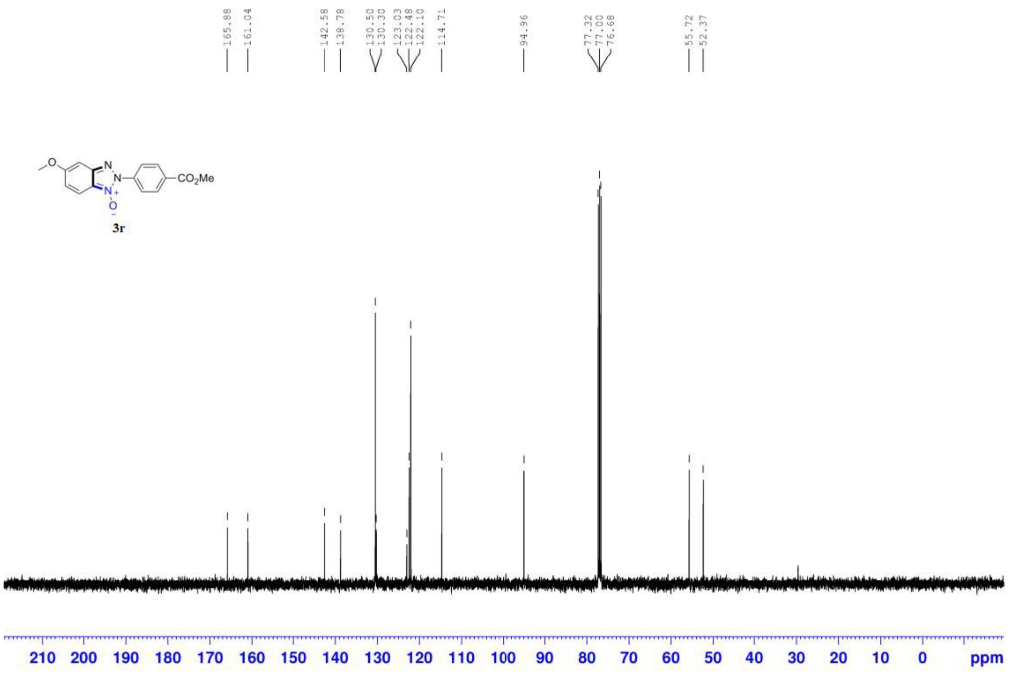

Supplementary Figure 68. ^{13}C NMR spectra of **3r**.

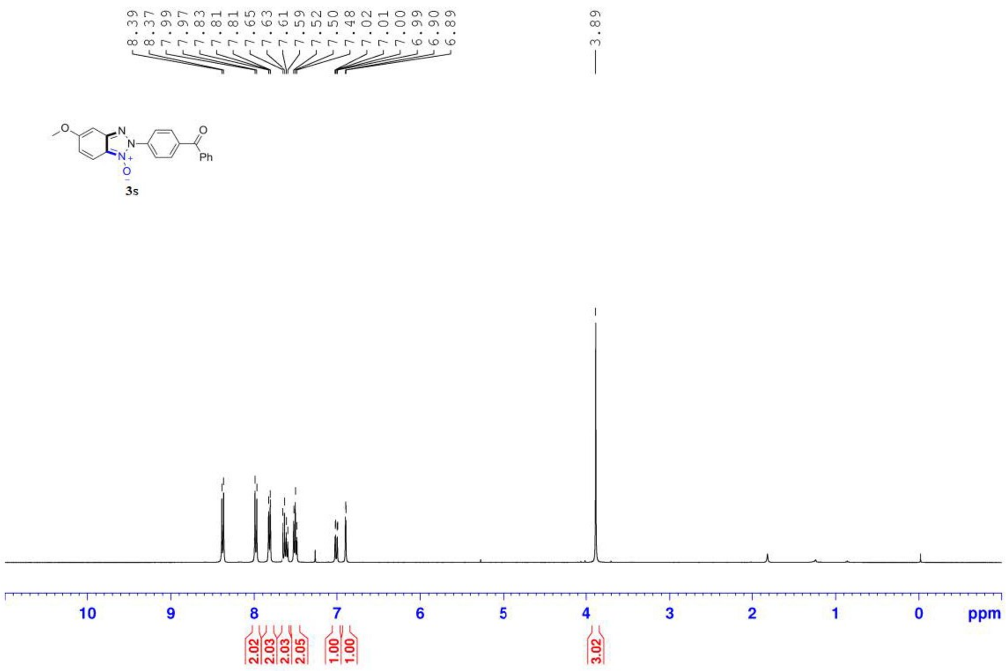

Supplementary Figure 69. ^{1}H NMR spectra of **3s**.

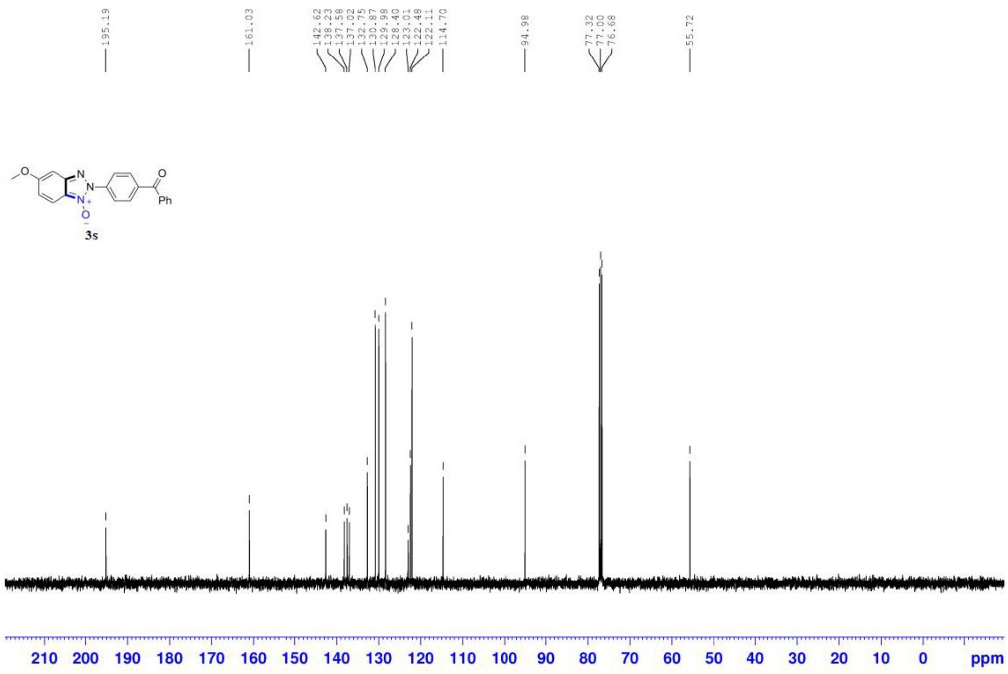

Supplementary Figure 70. ^{13}C NMR spectra of **3s**.

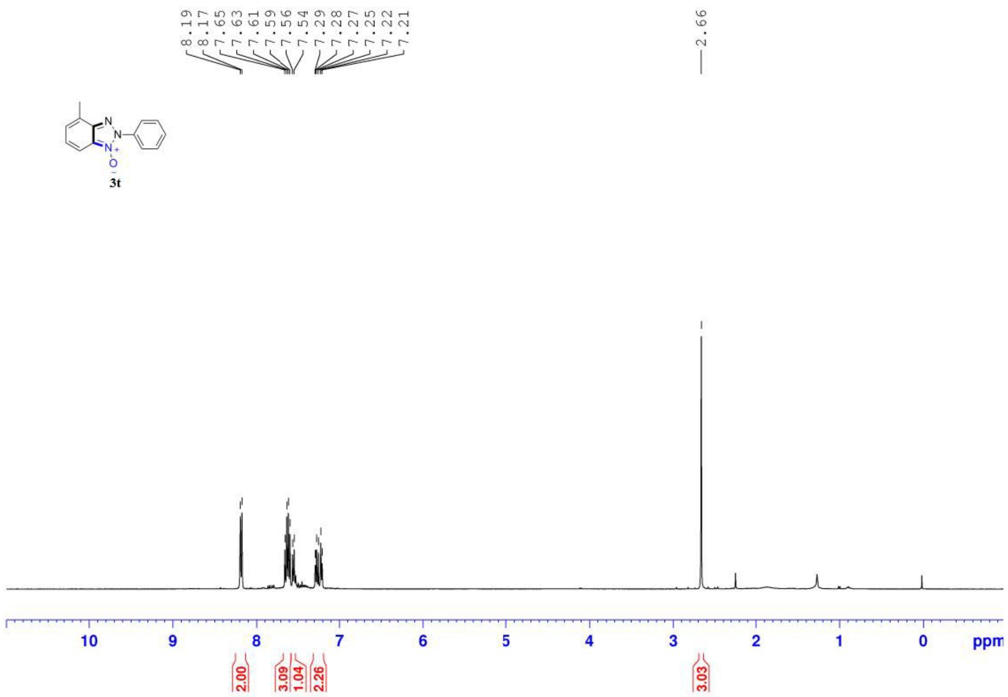

Supplementary Figure 71. ^{1}H NMR spectra of **3t**.

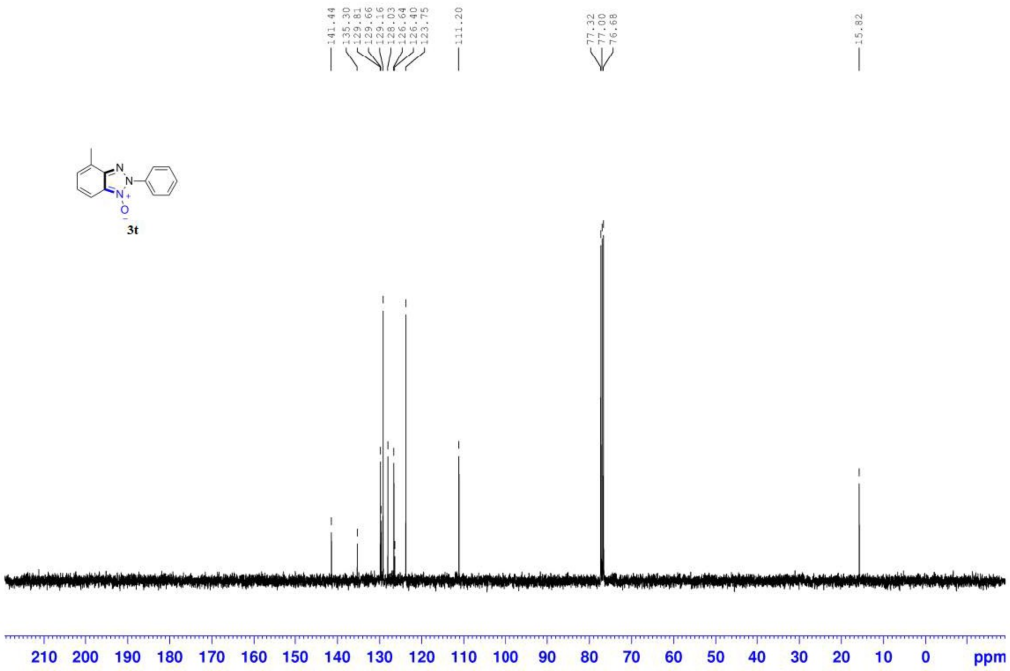

Supplementary Figure 72. ^{13}C NMR spectra of **3t**.

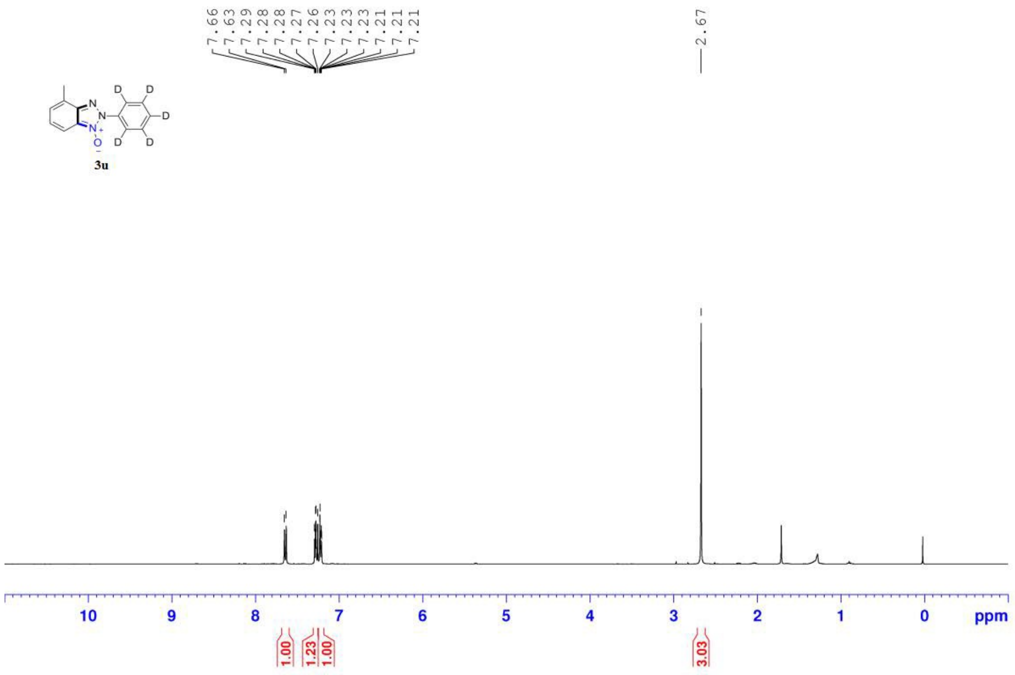

Supplementary Figure 73. ^{1}H NMR spectra of **3u**.

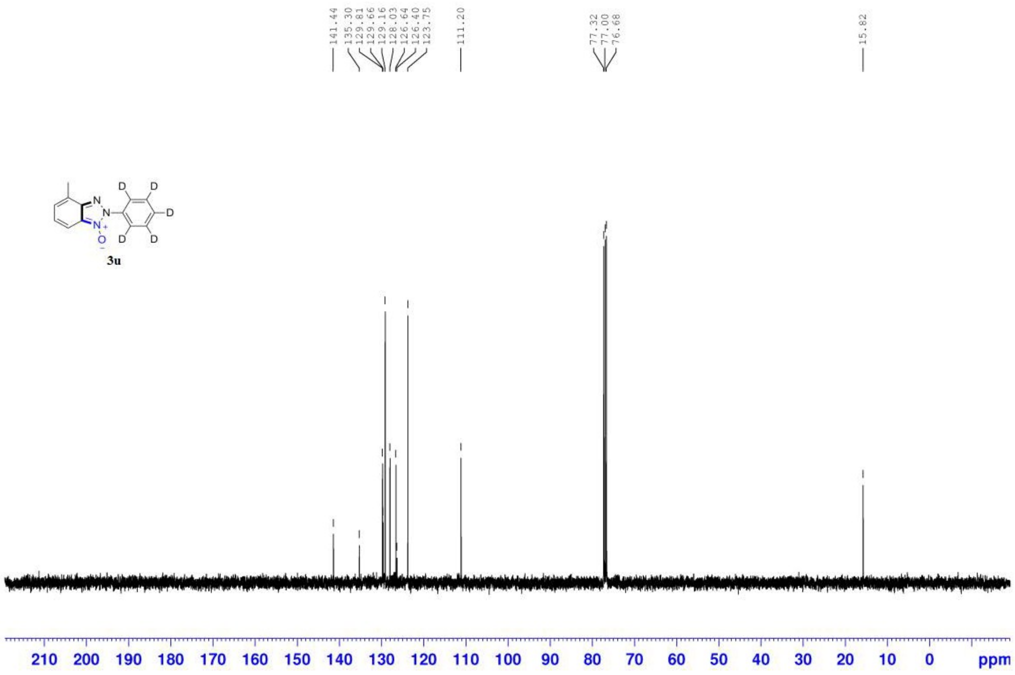

Supplementary Figure 74. ^{13}C NMR spectra of **3u**.

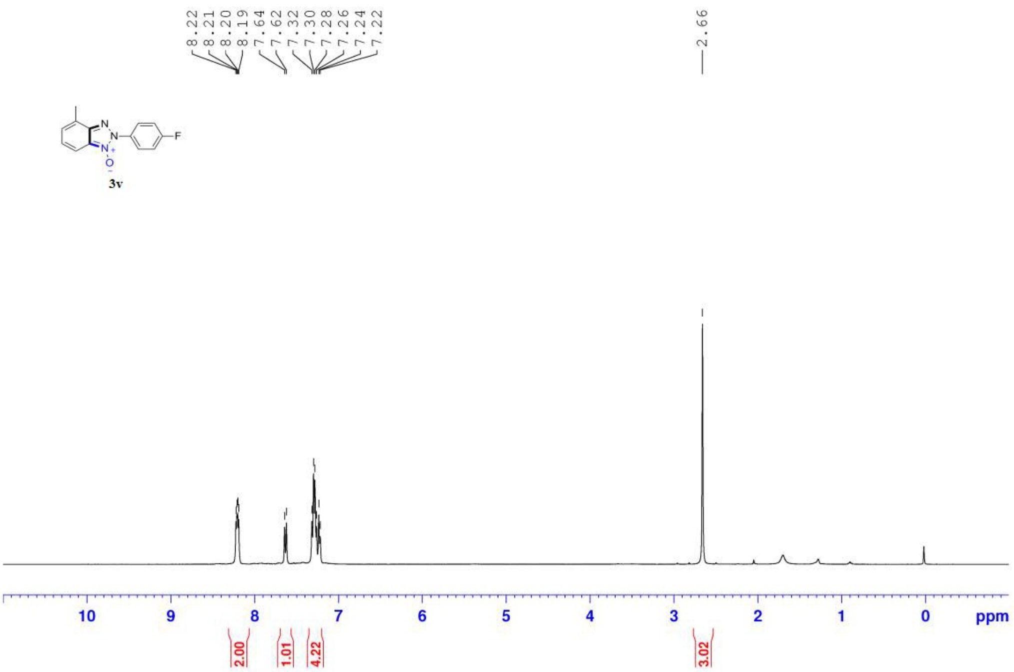

Supplementary Figure 75. ^{1}H NMR spectra of **3v**.

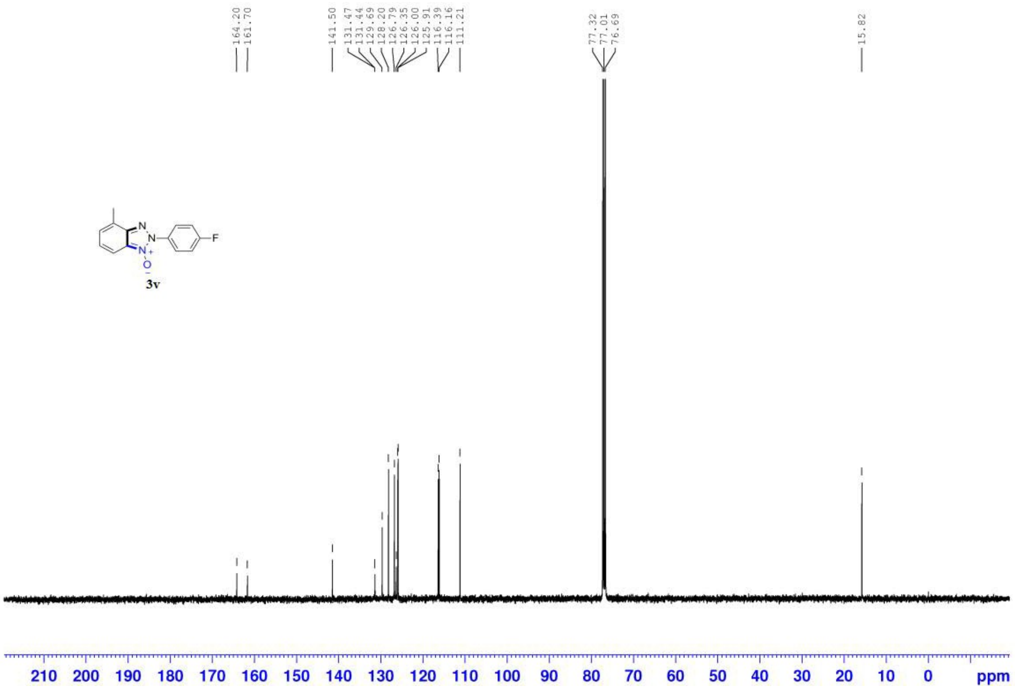

Supplementary Figure 76. ^{13}C NMR spectra of **3v**.

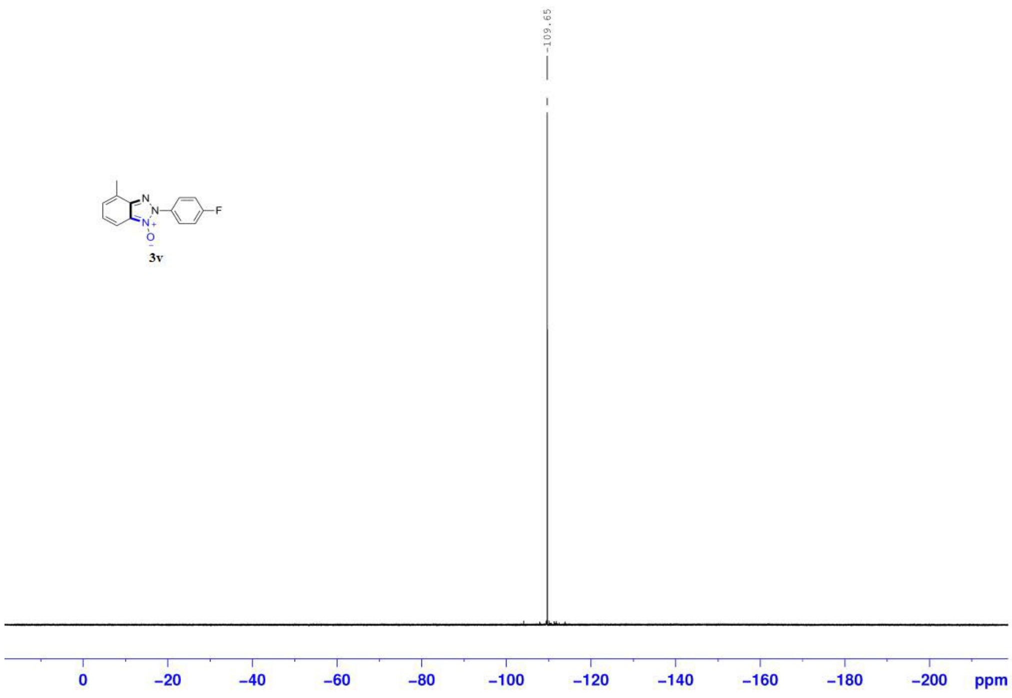

Supplementary Figure 77. ^{19}F NMR spectra of **3v**.

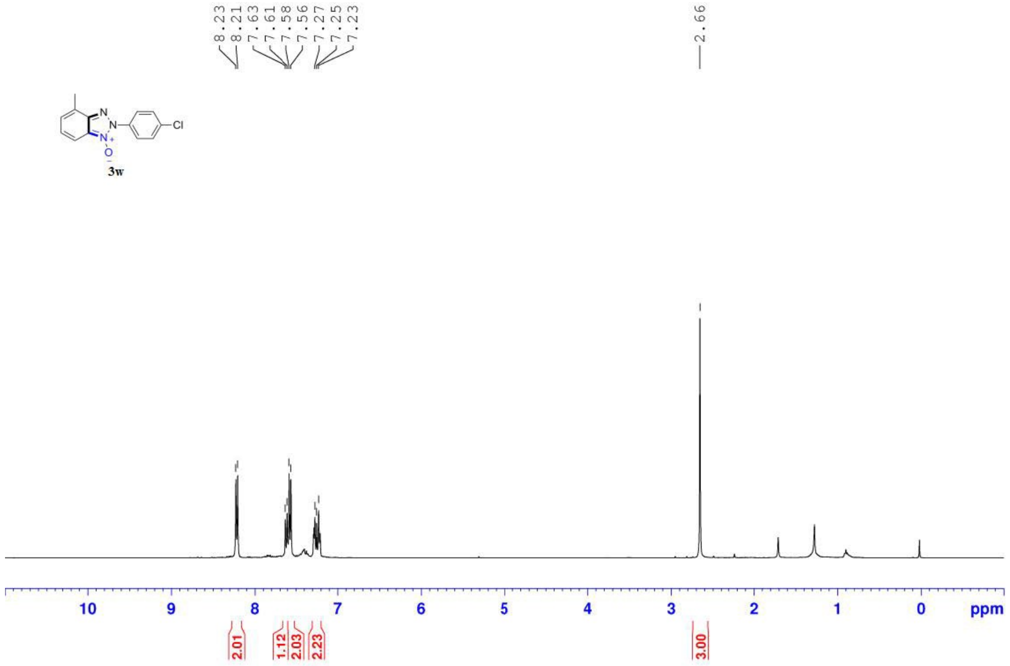

Supplementary Figure 78. ^{1}H NMR spectra of **3w**.

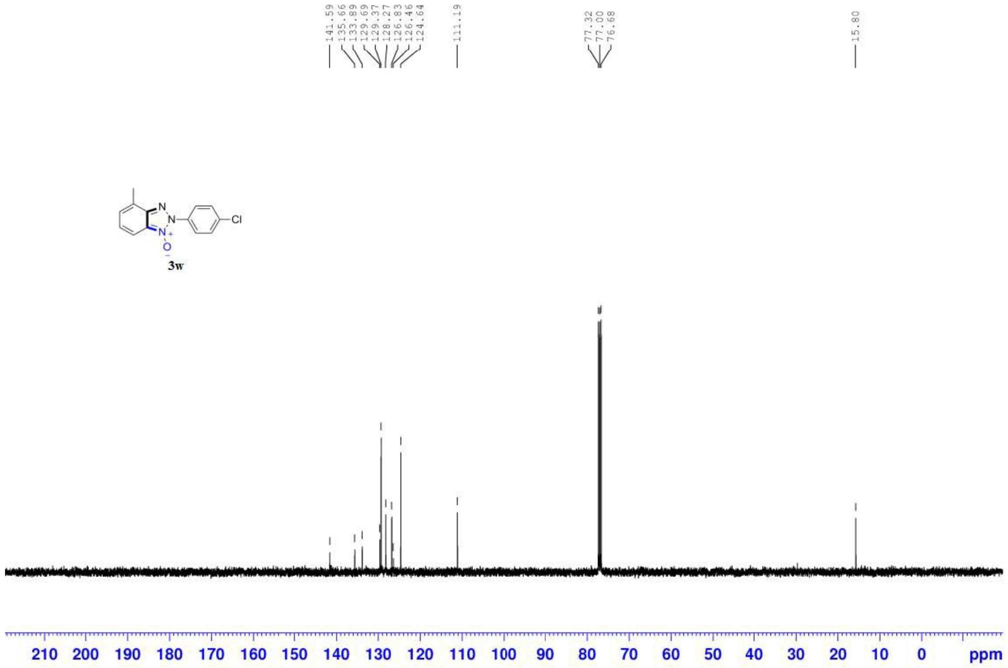

Supplementary Figure 79. ^{13}C NMR spectra of **3w**.

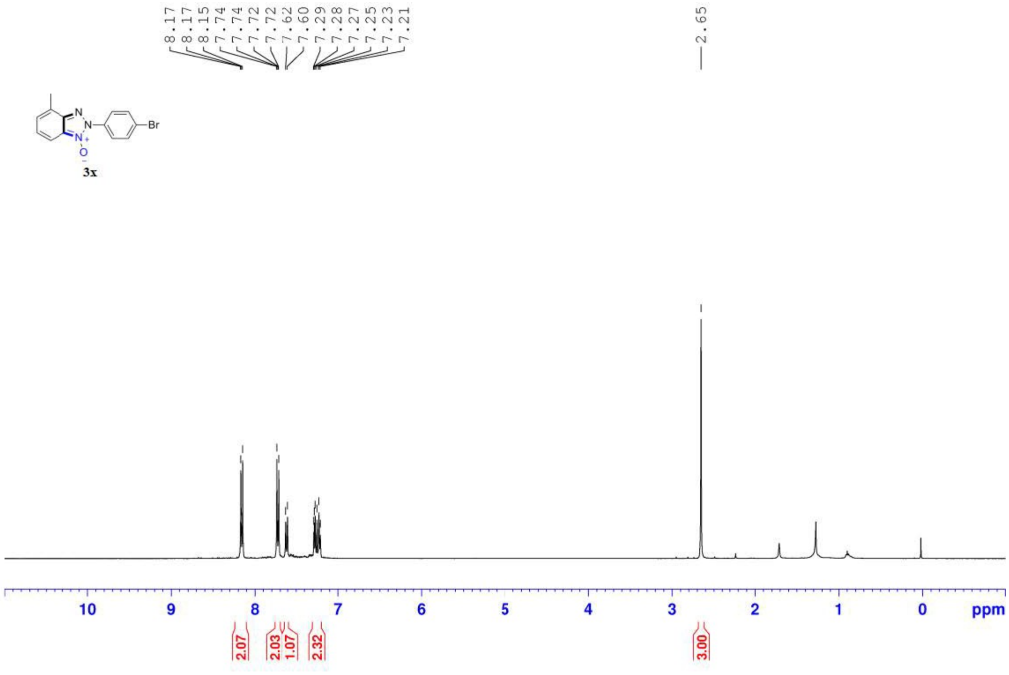

Supplementary Figure 80. ^{1}H NMR spectra of **3x**.

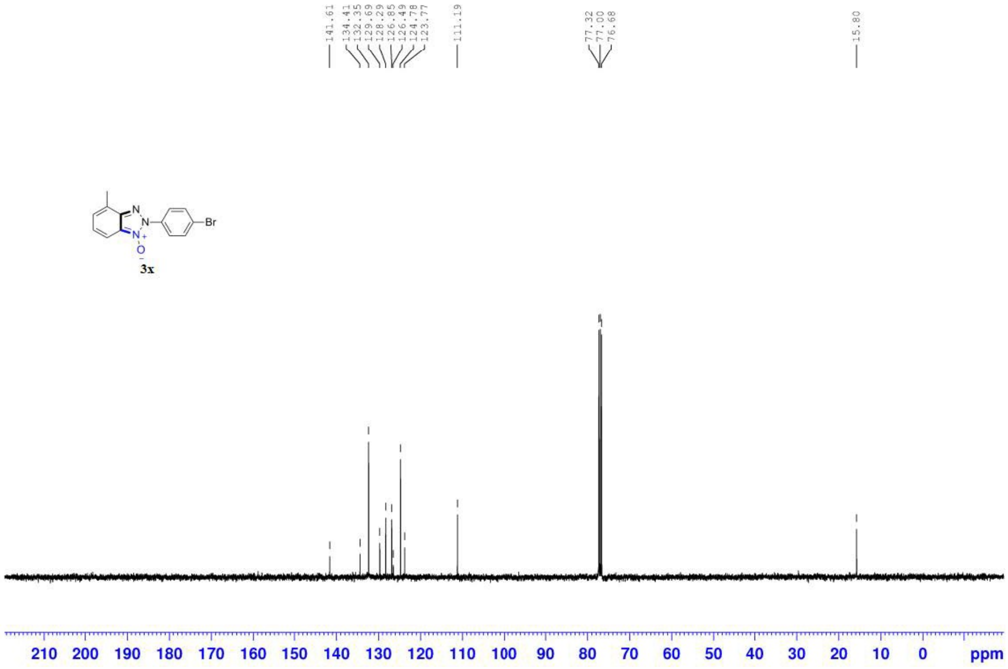

Supplementary Figure 81. ^{13}C NMR spectra of **3x**.

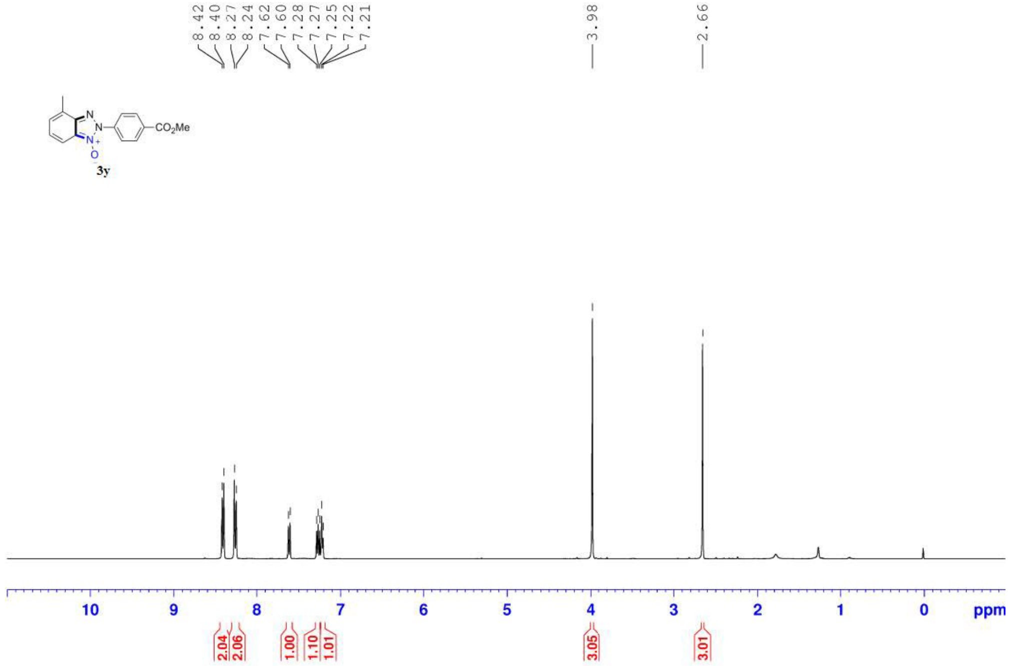

Supplementary Figure 82. ^{1}H NMR spectra of **3y**.

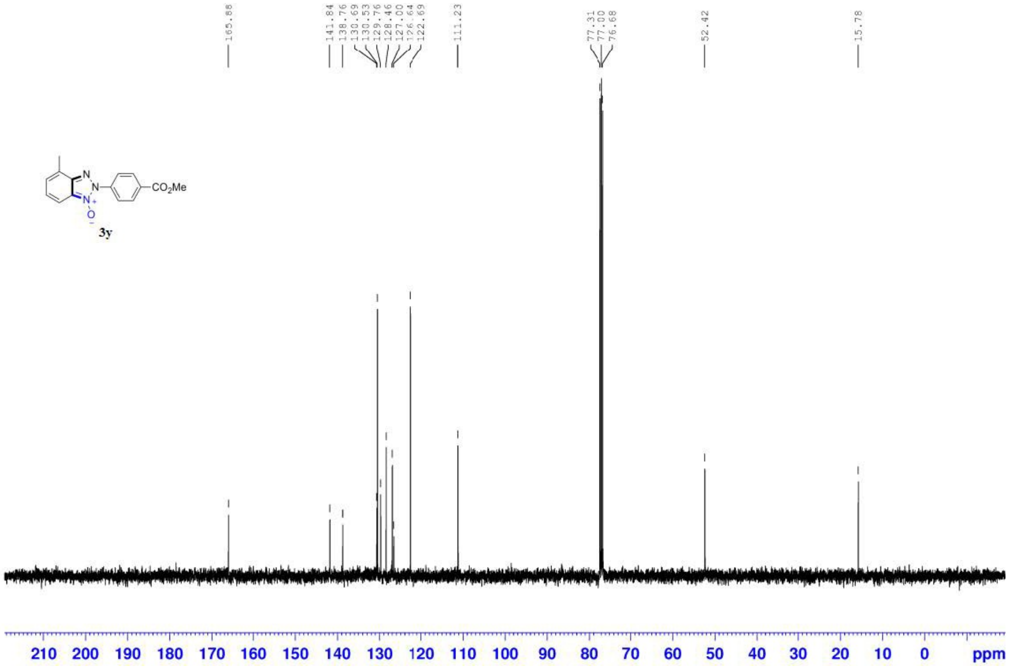

Supplementary Figure 83. ^{13}C NMR spectra of **3y**.

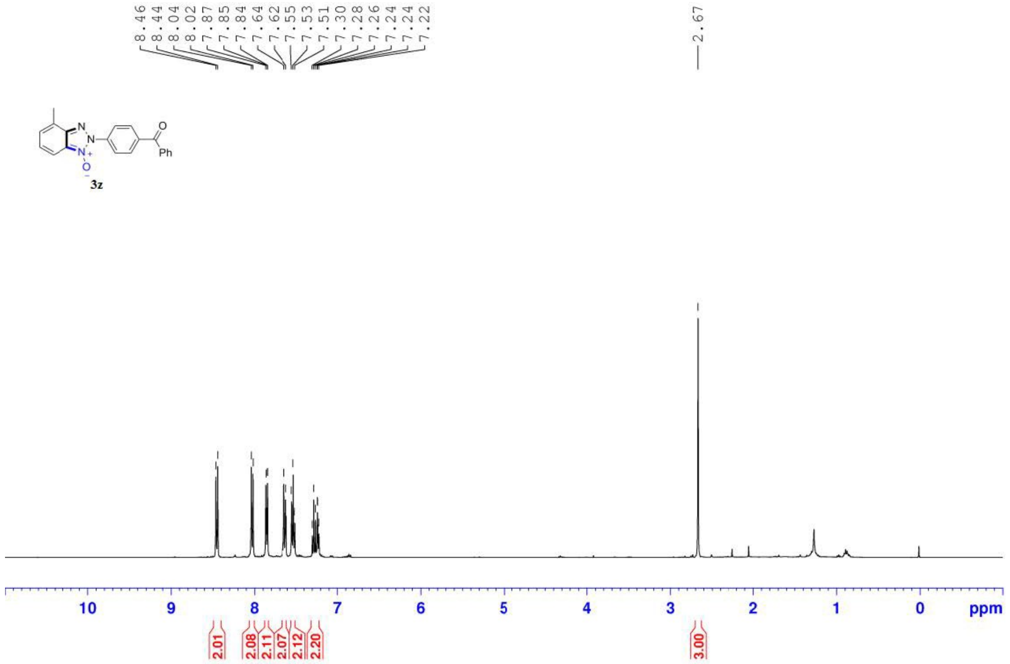

Supplementary Figure 84. ^{1}H NMR spectra of **3z**.

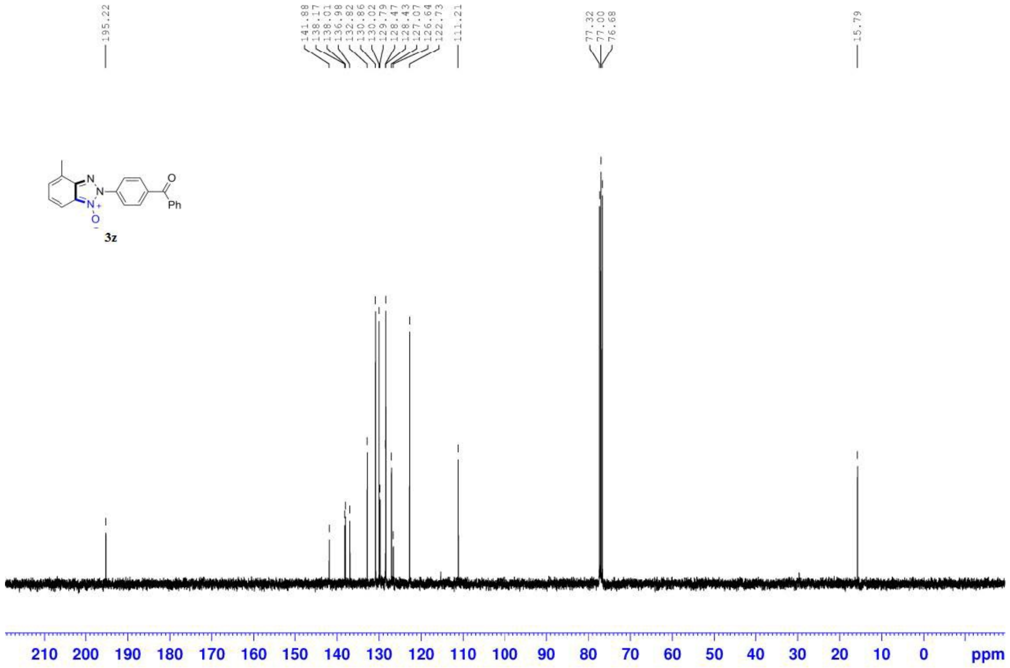

Supplementary Figure 85. ^{13}C NMR spectra of **3z**.

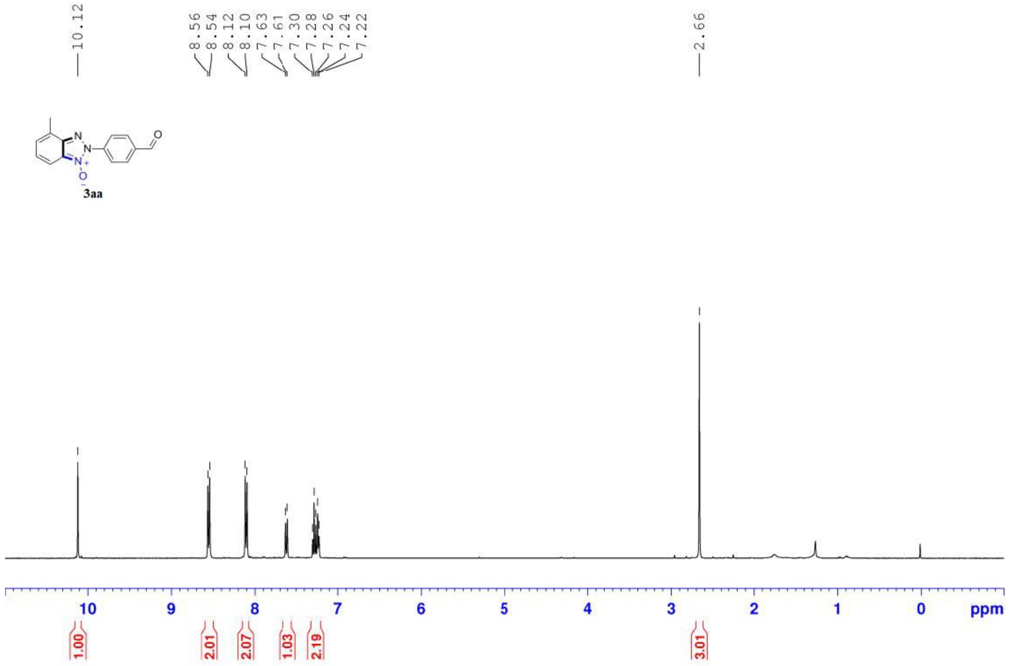

Supplementary Figure 86. ^{1}H NMR spectra of **3aa**.

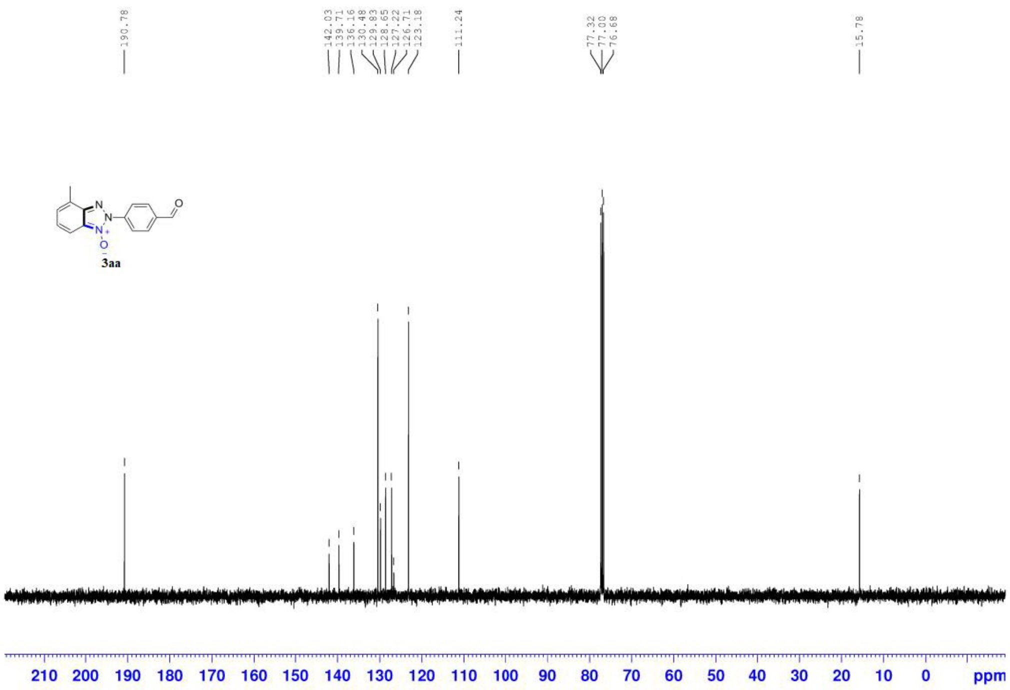

Supplementary Figure 87. ^{13}C NMR spectra of **3aa**.

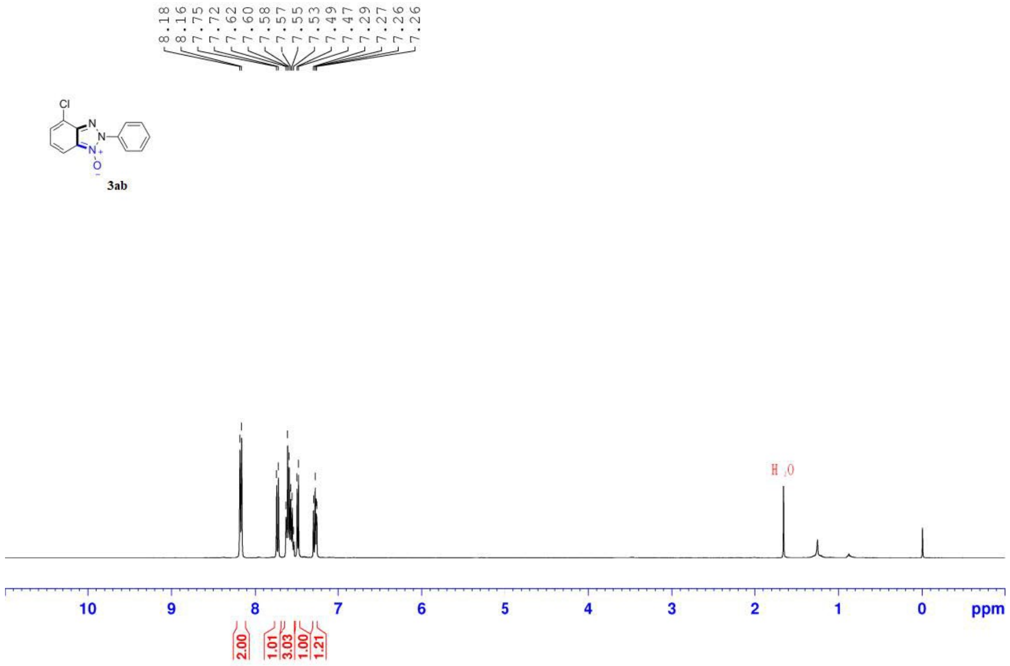

Supplementary Figure 88. ^{1}H NMR spectra of **3ab**.

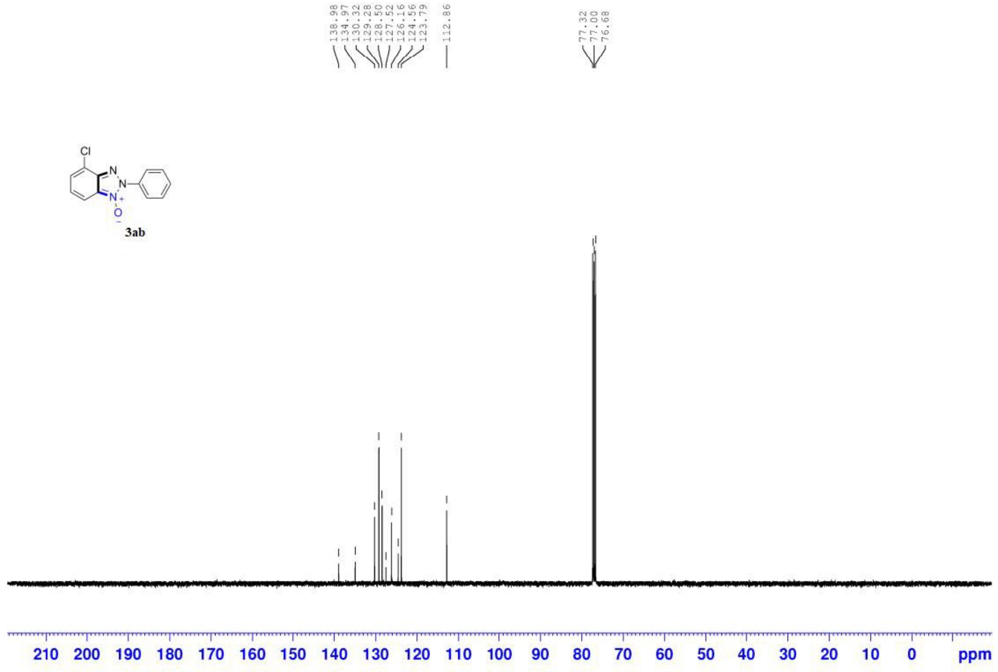

Supplementary Figure 89. ^{13}C NMR spectra of **3ab**.

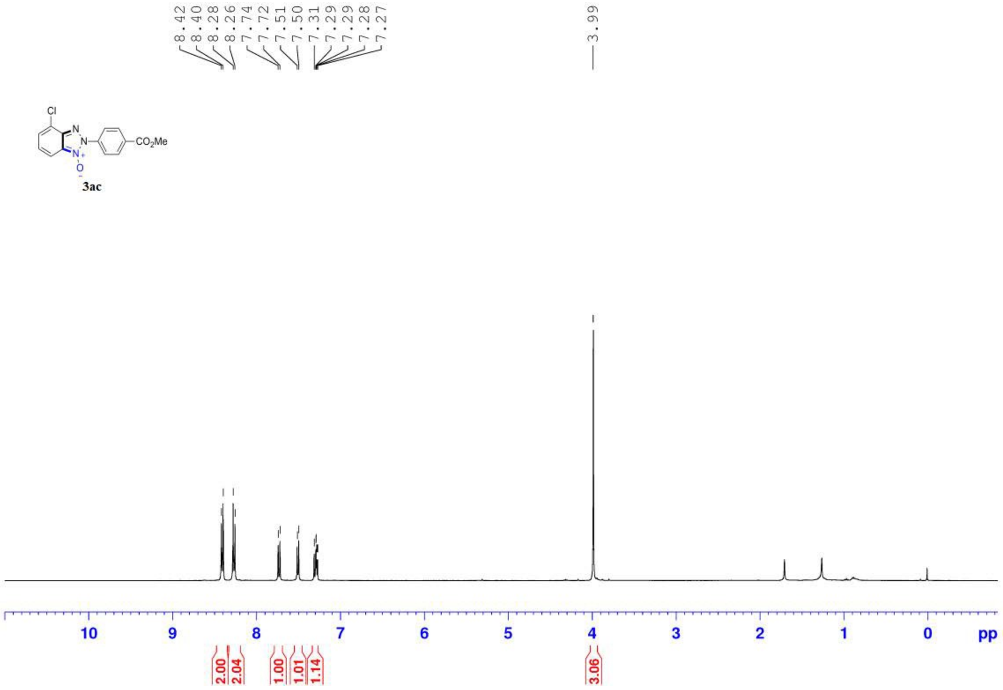

Supplementary Figure 90. ^{1}H NMR spectra of **3ac**.

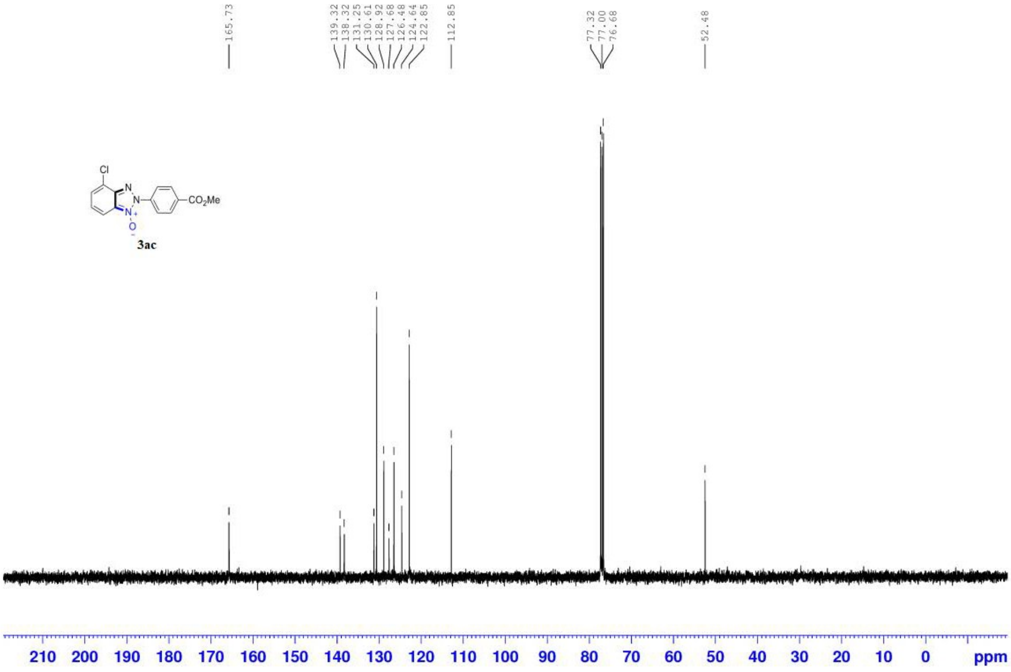

Supplementary Figure 91. ^{13}C NMR spectra of **3ac**.

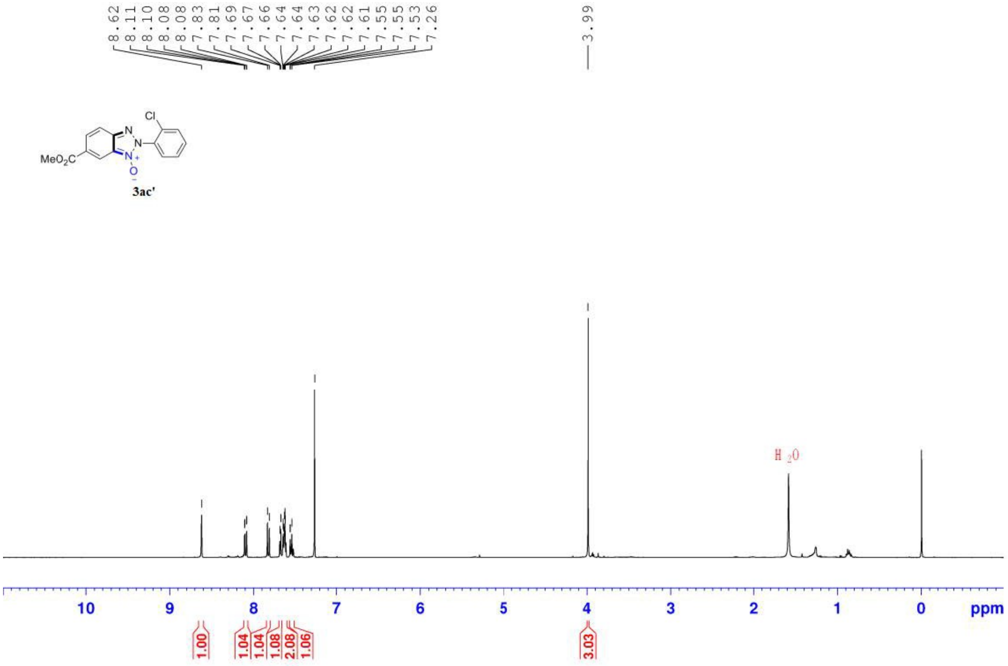

Supplementary Figure 92. ^{1}H NMR spectra of **3ac’**.

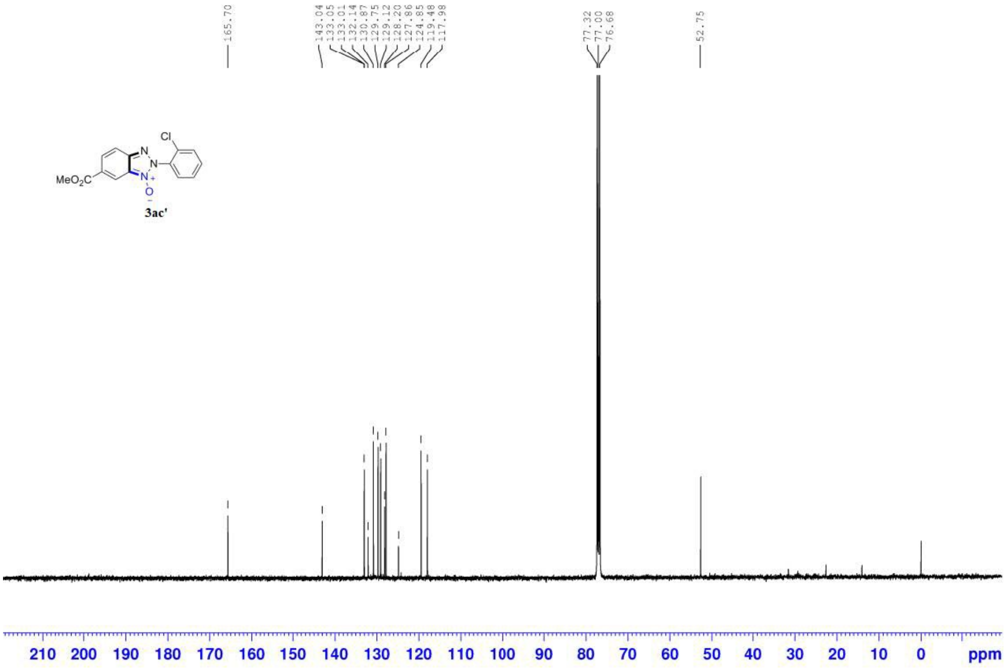

Supplementary Figure 93. ^{13}C NMR spectra of **3ac'**.

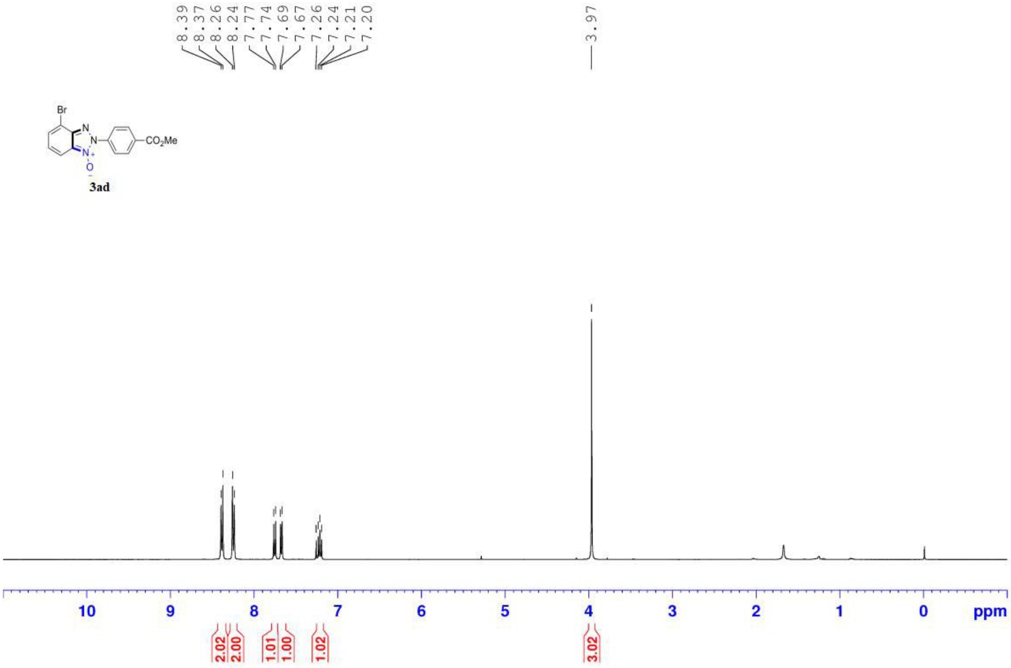

Supplementary Figure 94. ^{1}H NMR spectra of **3ad**.

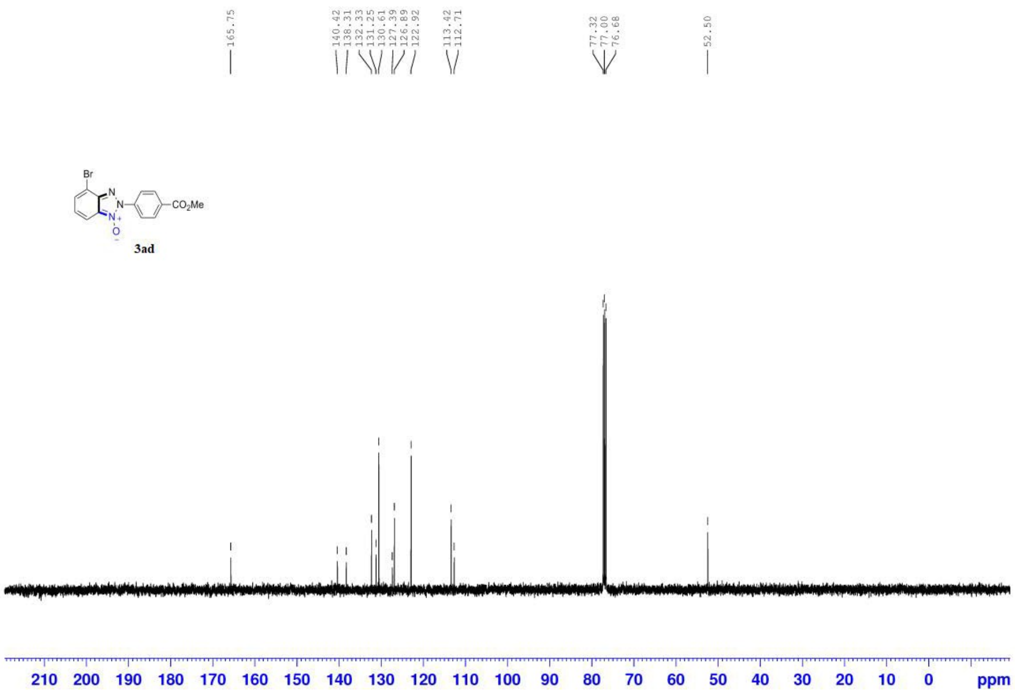

Supplementary Figure 95. ^{13}C NMR spectra of **3ad**.

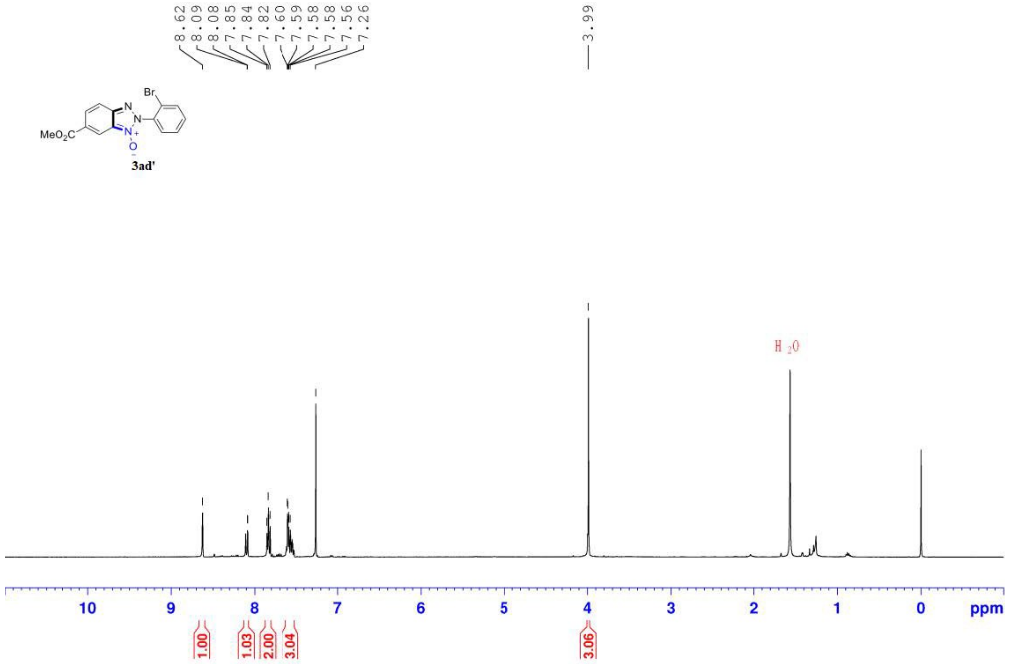

Supplementary Figure 96. ^{1}H NMR spectra of **3ad'**.

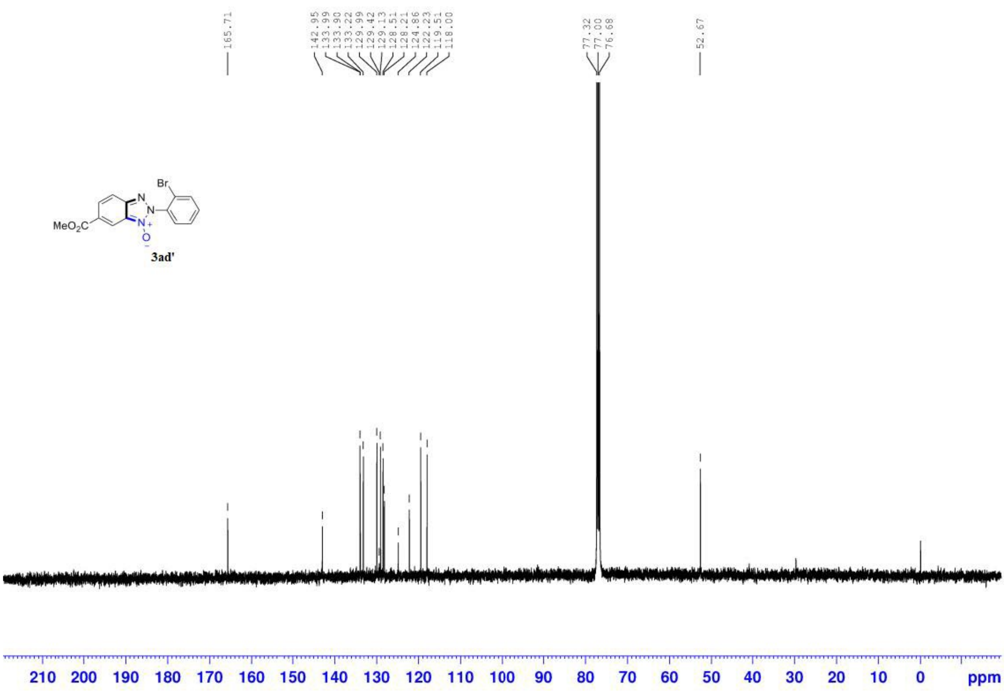

Supplementary Figure 97. ^{13}C NMR spectra of **3ad’**.

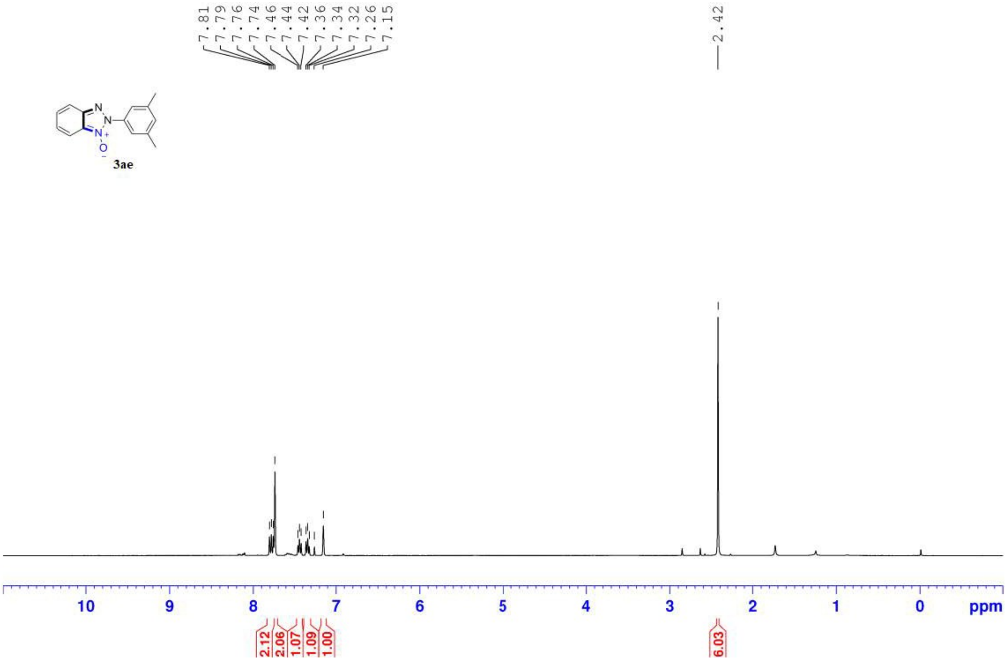

Supplementary Figure 98. ^{1}H NMR spectra of **3ae**.

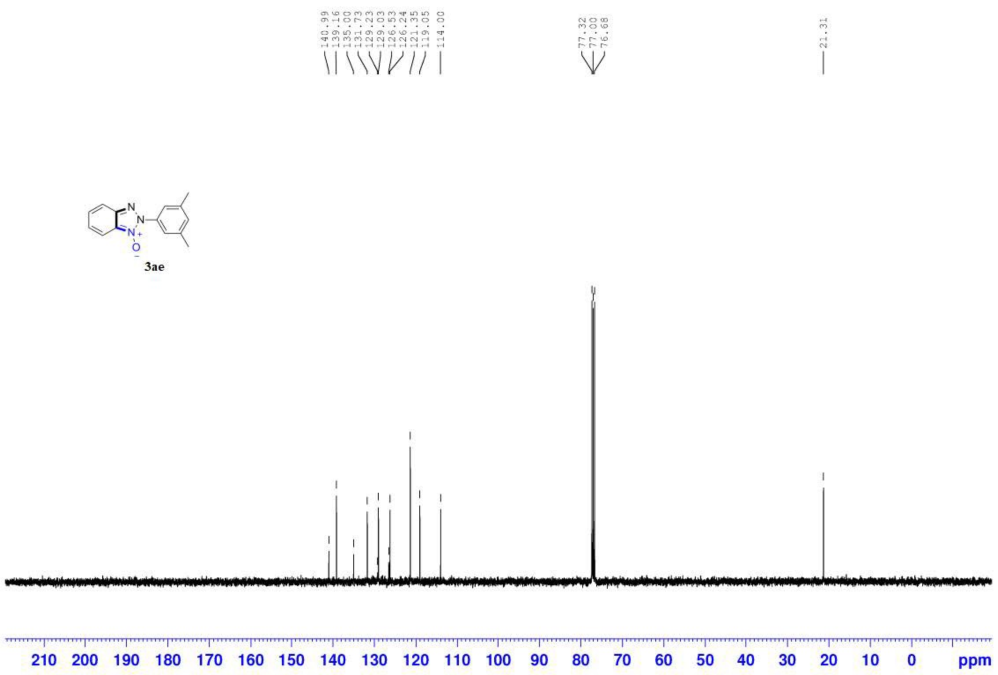

Supplementary Figure 99. ^{13}C NMR spectra of **3ae**.

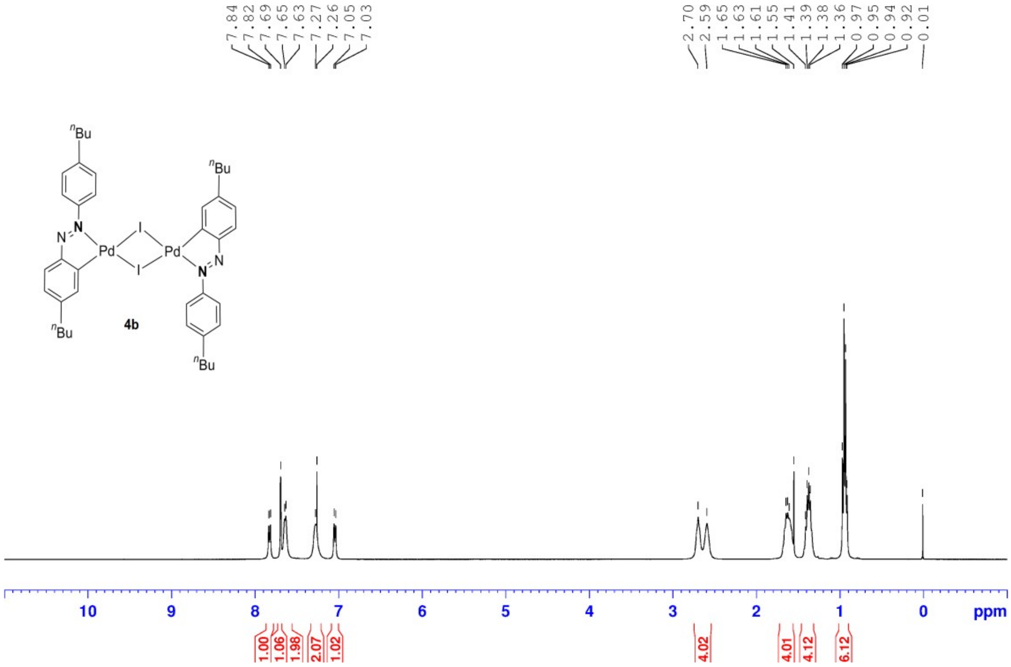

Supplementary Figure 100. ^{1}H NMR spectra of **4b**.

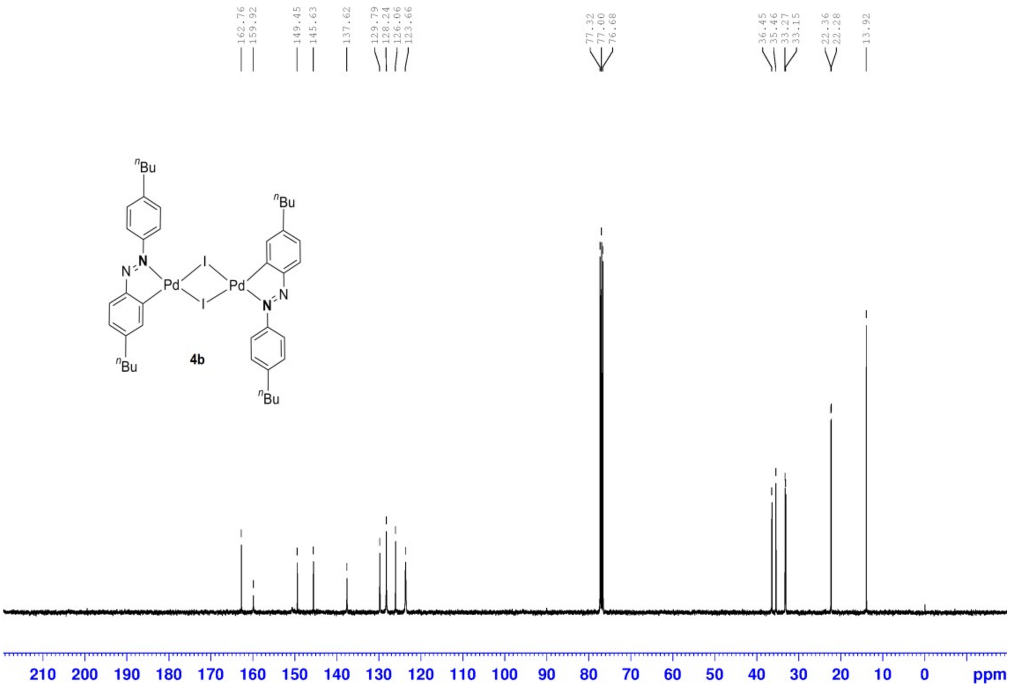

Supplementary Figure 101. ^{13}C NMR spectra of **4b**.

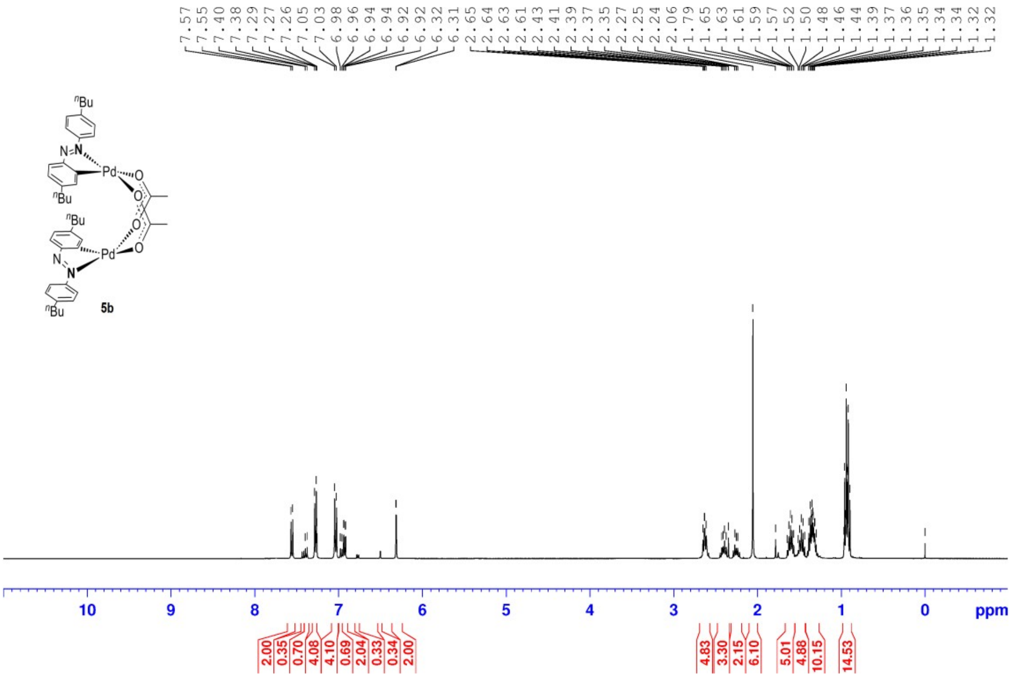

Supplementary Figure 102. ^{1}H NMR spectra of **5b**.

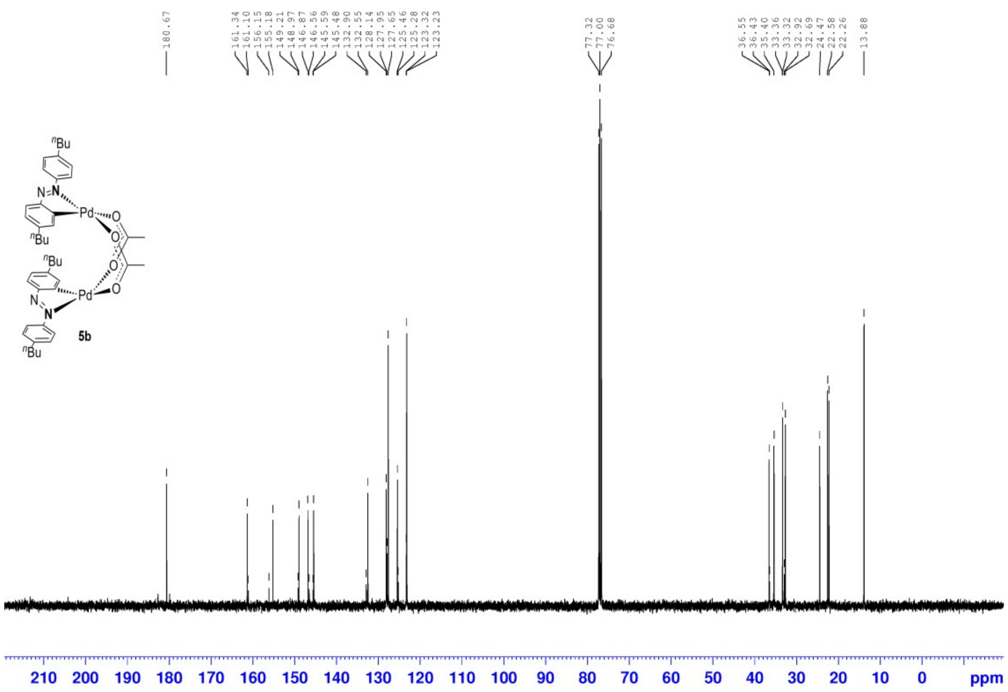

Supplementary Figure 103. ^{13}C NMR spectra of **5b**.
