# Supplementary material for: Iodide-enhanced palladium catalysis via formation of iodide-bridged binuclear palladium complex
Source: Commun Chem. 2020 Mar 31;3:41. doi: 10.1038/s42004-020-0287-0 (PMC9814094; doi:10.1038/s42004-020-0287-0)
Supplement: Supplementary file 2 — Supplementary Data 1 [file 42004_2020_287_MOESM2_ESM.pdf]

5a:

Pd	8.79345400	4.37628000	1.23197100
Pd	6.69577400	2.96249700	2.68851600
O	8.70962600	5.79743000	2.74580700
O	7.06909400	4.75183100	3.89454300
O	6.94120000	5.24626700	0.42959300
O	5.36434300	4.09641500	1.57007800
N	9.18437600	2.81886400	-0.09328200
N	10.20297600	2.10086000	0.13505600
N	7.94465400	1.55031100	3.57004800
N	7.97269000	0.39419000	3.05210800
C	7.91251300	5.66615200	3.72780800
C	8.05318600	6.68744500	4.82536800
H	7.09145500	6.86831300	5.31395300
H	8.47488700	7.62437200	4.45155000
H	8.74141100	6.27666200	5.57777200
C	5.75481300	4.93688800	0.69939700
C	4.67021500	5.57750800	-0.12603900
H	4.92791900	6.61261500	-0.36886000
H	3.70068200	5.53344700	0.37694700
H	4.59576900	5.02158000	-1.07133200
C	8.34199800	2.38037700	-1.14907400
C	7.57516000	3.33182500	-1.82380400
H	7.62880600	4.37451300	-1.52075000
C	6.75797700	2.93097000	-2.87396900
H	6.16915800	3.67339600	-3.40920600
C	6.69833600	1.59000600	-3.24303300
H	6.05351800	1.27892500	-4.06258500
C	7.46143400	0.64427900	-2.55889300
H	7.40310100	-0.40729500	-2.83369200
C	8.28327800	1.03104700	-1.51162500
H	8.85725800	0.29814300	-0.94942800
C	10.48012500	3.66655000	1.94045000
C	11.21946200	4.10266800	3.03025400
H	10.87586700	4.95327300	3.61881600
C	12.40554100	3.44524400	3.36894400
H	12.98116600	3.79555800	4.22502000
C	12.86214600	2.34439400	2.63995300
H	13.78706500	1.84789200	2.92494400
C	12.12891800	1.88347500	1.55756300
H	12.44925200	1.02405600	0.97115900
C	10.94354600	2.54427000	1.21302400
C	8.89979400	1.79321200	4.59272800
C	10.10380300	1.08309500	4.63241700

H	10.32095300	0.35607700	3.85356200
C	11.01297300	1.34216200	5.64572500
H	11.95776000	0.80204000	5.66902100
C	10.73207600	2.30407300	6.61575800
H	11.45324900	2.50590200	7.40531600
C	9.53191000	3.00816000	6.57019600
H	9.30618500	3.75441400	7.32952600
C	8.61276400	2.76028700	5.55774200
H	7.67384700	3.30583600	5.50586700
C	6.28404100	1.35802200	1.63553100
C	5.37750900	1.20325200	0.59669200
H	4.76157900	2.04417400	0.27916100
C	5.25018800	-0.03857900	-0.03203000
H	4.53053500	-0.15226100	-0.84208800
C	6.03125200	-1.13178800	0.34982500
H	5.91785500	-2.08698800	-0.15812200
C	6.95077100	-0.99475100	1.37816300
H	7.57765900	-1.82434800	1.70036500
C	7.07085800	0.24513000	2.01800600

LM1:

Pd	8.65168400	4.43007200	1.45391400
Pd	6.63095700	2.90674600	2.75616700
O	8.53401100	5.76689300	3.02574200
O	6.84241900	4.67902400	4.04731200
O	6.86153000	5.36359800	0.63821700
O	5.32208200	4.04135800	1.62393600
N	9.11881700	2.92477200	0.03821500
N	10.15884900	2.25102100	0.27312900
N	7.91362800	1.51837900	3.62646800
N	7.99958200	0.38472500	3.07252400
C	7.67738100	5.60346100	3.96621300
C	7.76216300	6.60436300	5.08145500
H	6.78574900	6.73046500	5.55679000
H	8.14777000	7.56481800	4.73025800
H	8.46044100	6.20798400	5.83183400
C	5.67773300	4.97736700	0.84858100
C	4.58845700	5.64454900	0.06055500
H	4.83554000	6.69100300	-0.13756700
H	3.62508400	5.56561300	0.57046300
H	4.50896700	5.12679000	-0.90576200
C	8.33534900	2.51969800	-1.07624800
C	7.46083000	3.43728700	-1.66330000
H	7.37526000	4.44609800	-1.26915600

C	6.70369300	3.05011200	-2.76165800
H	6.02828300	3.76655900	-3.22415800
C	6.81202900	1.75872700	-3.26845700
H	6.21323100	1.45905200	-4.12576500
C	7.68503300	0.84674600	-2.67478600
H	7.76103700	-0.16765900	-3.06040200
C	8.44730500	1.21755500	-1.57985200
H	9.11537400	0.50868800	-1.09889000
C	10.35802200	3.69526800	2.19121300
C	11.05182300	4.12747600	3.29925300
H	10.68181500	4.95011300	3.90850400
C	12.25374500	3.48307000	3.62555100
H	12.80706500	3.81392800	4.50253100
C	12.74823100	2.43181300	2.85444000
H	13.68258600	1.94976500	3.13041700
C	12.05188600	2.00110200	1.73423800
H	12.41242600	1.18623700	1.11027500
C	10.85275300	2.63898500	1.39628700
C	8.79680300	1.75106200	4.71370900
C	10.01227000	1.06381300	4.79842300
H	10.28463400	0.36092300	4.01471900
C	10.86165300	1.31688500	5.86330800
H	11.81639300	0.79785600	5.92297400
C	10.50659800	2.24485100	6.84265500
H	11.18057900	2.44243300	7.67345600
C	9.29076000	2.91770400	6.75684800
H	9.00408600	3.63034800	7.52735000
C	8.43179800	2.67893800	5.69081200
H	7.47789500	3.19489800	5.61536900
C	6.35456100	1.33972500	1.60300500
C	5.48684500	1.20152500	0.53461800
H	4.85419900	2.03286600	0.22575600
C	5.42675200	-0.02333600	-0.14154300
H	4.73899400	-0.13135700	-0.97884800
C	6.22991200	-1.10147800	0.23206200
H	6.16753300	-2.04038900	-0.31277000
C	7.10509300	-0.97499300	1.30142700
H	7.73963900	-1.79818500	1.62357800
C	7.16102600	0.24368100	1.98682000
N	9.74187400	6.00196800	0.58199300
O	9.72543600	6.04626900	-0.55144500

LM1-1a:

Pd	8.94247100	4.44174600	1.22949100

Pd	7.01847100	3.15476900	2.84318400
O	8.89852500	5.99229700	2.59839500
O	7.27708700	5.07552000	3.87127800
O	7.13736000	5.26927300	0.34918000
O	5.66320300	4.15165400	1.63796400
N	9.32139400	2.74113100	0.02553200
N	10.30356500	2.03146100	0.37510400
N	8.36204300	1.85925600	3.80070800
N	8.42964500	0.68017200	3.34987200
C	8.10602400	5.97165400	3.60617300
C	8.26826300	7.10890500	4.57125000
H	7.30927600	7.35382900	5.03597200
H	8.70253900	7.99015400	4.09306100
H	8.95171800	6.77016300	5.36307300
C	5.96806500	4.93938500	0.69646600
C	4.81671100	5.48283100	-0.09886000
H	3.97506500	5.72132300	0.56124600
H	4.47645500	4.69715600	-0.78821900
H	5.11300800	6.35887400	-0.68115500
C	8.49955000	2.22775400	-1.01267700
C	7.67623500	3.10669700	-1.71979300
H	7.66374000	4.16395600	-1.47221400
C	6.87296900	2.61565300	-2.74122200
H	6.23691200	3.29986100	-3.29819200
C	6.88486600	1.25905700	-3.05114700
H	6.24836600	0.87699100	-3.84620400
C	7.70851400	0.38676000	-2.33827400
H	7.70794700	-0.67629600	-2.56971500
C	8.51782900	0.86115900	-1.32039300
H	9.14419000	0.18512200	-0.74493500
C	10.63966400	3.74989300	2.02980400
C	11.38388400	4.30054200	3.04943800
H	11.09103400	5.23880500	3.51714300
C	12.53478600	3.62248900	3.47682100
H	13.12931300	4.05163100	4.28108100
C	12.92524400	2.41556700	2.89814200
H	13.81869000	1.90848200	3.25329700
C	12.17936800	1.86387400	1.86634300
H	12.46020800	0.92756300	1.38903500
C	11.03586800	2.53849100	1.42315100
C	9.23998200	2.16424800	4.87509900
C	10.35172100	1.35758700	5.14695800
H	10.56709600	0.50820900	4.50392400
C	11.17286400	1.66906100	6.21869300

H	12.04236000	1.04866200	6.42635300
C	10.89607900	2.77566200	7.02279900
H	11.54538000	3.01409800	7.86260300
C	9.78987200	3.57352500	6.74777400
H	9.56186500	4.43241300	7.37606800
C	8.96034700	3.27434000	5.67387200
H	8.08541700	3.88382000	5.46488000
C	6.68561600	1.46714600	1.89424800
C	5.71444500	1.19883400	0.94558600
H	5.03902100	1.98394000	0.60569600
C	5.59391800	-0.10162300	0.44310800
H	4.82670500	-0.31231600	-0.30058800
C	6.43983600	-1.12769400	0.86892500
H	6.32800700	-2.12863400	0.45876900
C	7.41717100	-0.87023700	1.81917200
H	8.08137100	-1.65033600	2.18621200
C	7.53189100	0.42572400	2.33633300
N	10.03231500	5.85432900	0.11829600
O	9.93858800	5.77356900	-1.01066800
N	3.59070100	3.33863500	3.91626200
N	3.93748400	2.13436200	3.91130700
C	2.73944700	3.71900900	2.85268200
C	2.29915100	5.04325500	2.88292100
H	2.61746600	5.67665200	3.70936500
C	1.46814700	5.52658200	1.87786600
H	1.12123900	6.55747600	1.90758100
C	1.08391600	4.68757500	0.83578800
H	0.43473300	5.06183900	0.04644200
C	1.52978200	3.36402000	0.80174200
H	1.22729000	2.71218100	-0.01608600
C	2.35297000	2.87501100	1.80345300
H	2.70837900	1.84704800	1.79196800
C	5.20728500	2.60705700	6.00722800
C	6.09381500	2.14161900	6.96536600
H	6.39899100	2.79407500	7.78218200
C	6.60880600	0.84568800	6.88227100
H	7.32229800	0.49693600	7.62738900
C	6.21315800	0.00292200	5.84732900
H	6.61013000	-1.00855800	5.78072700
C	5.31269700	0.45766900	4.89076000
H	4.99331800	-0.17847100	4.06494800
C	4.82025700	1.76340800	4.95574700
H	4.80201700	3.61522500	6.05542200

LM1-BF4:

Pd	8.66083500	4.40466900	1.39642500
Pd	6.60655500	3.09201300	2.76415800
O	8.67393300	5.82889900	2.89056600
O	6.95358400	4.88661500	3.99668300
O	6.94156600	5.44325600	0.55487800
O	5.33777500	4.27787600	1.62880100
N	9.05285500	2.83846400	0.01527300
N	10.03328500	2.09540000	0.29452700
N	7.84491600	1.67418800	3.63891400
N	7.92029900	0.53952700	3.09181000
C	7.83603600	5.75283100	3.86196500
C	8.01626300	6.78614600	4.93587100
H	7.05808600	7.00647700	5.41391400
H	8.47259900	7.69975400	4.54625000
H	8.68612900	6.36103800	5.69633200
C	5.73699600	5.14744500	0.80339800
C	4.68157700	5.84674800	-0.00338700
H	4.97659800	6.87622600	-0.22489400
H	3.71477200	5.82361900	0.50529500
H	4.58283100	5.31114900	-0.95834000
C	8.28101000	2.47248900	-1.12117600
C	7.42796900	3.41929500	-1.69311000
H	7.36101000	4.42056300	-1.27593500
C	6.66645300	3.07086300	-2.80153500
H	6.00314200	3.80872600	-3.24790400
C	6.75328700	1.79010200	-3.33771600
H	6.14988800	1.51882200	-4.20133900
C	7.61426500	0.85215000	-2.76697700
H	7.67968400	-0.15213600	-3.18022000
C	8.38022300	1.18421800	-1.66244900
H	9.04263400	0.45628900	-1.20335200
C	10.33227400	3.61029600	2.14331500
C	11.07557600	4.06234100	3.21217000
H	10.77195700	4.94483000	3.77332800
C	12.23820000	3.36170300	3.56087500
H	12.82912700	3.70957700	4.40624600
C	12.64403400	2.22868300	2.85605400
H	13.54683000	1.70007300	3.15146400
C	11.90086700	1.77822200	1.77451400
H	12.19671500	0.90553300	1.19611500
C	10.74590200	2.47926400	1.40750800
C	8.65731100	1.88918000	4.78189400
C	9.87122200	1.21339300	4.93363500

H	10.19927000	0.51857000	4.16402400
C	10.65672100	1.47511100	6.04567900
H	11.61383700	0.96884800	6.15828000
C	10.23396300	2.39509700	7.00506800
H	10.85766100	2.60115800	7.87254600
C	9.01209900	3.04677800	6.85600400
H	8.66857200	3.74748900	7.61437300
C	8.21723700	2.80059800	5.74293000
H	7.24952500	3.27983200	5.61770400
C	6.31921800	1.53130700	1.60115800
C	5.43971600	1.40652600	0.54087800
H	4.81607200	2.24751700	0.23973200
C	5.34867200	0.18164600	-0.12799900
H	4.65046200	0.08264000	-0.95805300
C	6.13199300	-0.91265000	0.24487700
H	6.04276100	-1.85372800	-0.29290600
C	7.01496100	-0.80065700	1.30848200
H	7.62662500	-1.63852800	1.63749900
C	7.10196200	0.42023500	1.98732200
N	9.85726600	5.86881300	0.45283100
O	9.77334400	5.91109100	-0.68203100
B	4.16965800	1.79715900	4.57203300
F	3.22668200	1.68810600	5.58220000
F	4.92498900	2.98236800	4.77288900
F	5.04518300	0.70854400	4.59809600
F	3.54589100	1.87479600	3.32531200

LM2:

Pd	8.59761500	4.62080600	0.79804900
Pd	7.50175900	2.65764000	2.77353600
O	8.45108300	5.93847000	2.36274800
O	7.14286100	4.63462900	3.66511700
O	6.58354600	4.90075600	0.48468100
O	5.85836200	3.02493500	1.51683200
N	8.71388700	3.25963200	-0.72905600
N	9.46456800	2.25906800	-0.73800800
N	9.10860700	1.98838200	3.89038100
N	9.59839300	0.85519300	3.60823200
C	7.71474300	5.71170100	3.38391000
C	7.55791200	6.86030800	4.34082900
H	7.93221900	7.79627200	3.92011500
H	8.11648400	6.62927100	5.25677600
H	6.50403000	6.96471700	4.61817200
C	5.69822300	4.03591600	0.78506900

C	4.35515800	4.23580000	0.14718700
H	4.11047300	5.30035500	0.09181200
H	3.57766100	3.69021100	0.68695600
H	4.40557800	3.85368100	-0.88185300
C	7.72791900	3.27740100	-1.75458500
C	7.30926500	4.51604200	-2.24580100
H	7.73434900	5.43065700	-1.83945600
C	6.37237300	4.55359300	-3.26779400
H	6.05253800	5.51119900	-3.67067000
C	5.83737600	3.36768800	-3.76722400
H	5.08958000	3.40284800	-4.55620500
C	6.24956400	2.13532100	-3.25770400
H	5.82155800	1.21352100	-3.64414700
C	7.20474300	2.07975700	-2.25578400
H	7.53867600	1.13130200	-1.84060400
C	11.11172100	3.09058900	1.00690100
C	12.30005100	2.80746200	1.68777600
H	12.74095500	3.58692800	2.30394100
C	12.87153800	1.54759600	1.60068900
H	13.78051100	1.32783700	2.15429900
C	12.27434200	0.56845700	0.80887100
H	12.71455400	-0.42303700	0.74115800
C	11.12807000	0.86127500	0.07792200
H	10.67660400	0.12527600	-0.58349100
C	10.51774700	2.11339300	0.16200700
C	9.82321600	2.75894600	4.84522900
C	11.21460300	2.65013900	4.94817500
H	11.74936500	1.96554900	4.29328100
C	11.89083100	3.42259500	5.88059700
H	12.97365100	3.35097400	5.95486100
C	11.18711900	4.28704500	6.71800400
H	11.72182300	4.89021800	7.44840500
C	9.80134500	4.37858900	6.62077100
H	9.24931200	5.04030100	7.28493300
C	9.11266900	3.62480900	5.67852200
H	8.03134600	3.69181700	5.58918900
C	7.82171000	0.88588800	1.99068600
C	7.17164400	0.27581100	0.92897200
H	6.33616900	0.77239600	0.43495200
C	7.59275400	-0.98639000	0.49408200
H	7.07239900	-1.46223700	-0.33614700
C	8.65821100	-1.64962600	1.10629900
H	8.96452200	-2.63103600	0.75272600
C	9.32698100	-1.04968500	2.16343900

H	10.17177400	-1.53007400	2.65429200
C	8.91036900	0.21418300	2.59688700
N	10.52689700	4.36136600	1.18990000
O	11.17443100	5.23100800	1.72059300

LM3:

Pd	9.35264100	3.76335600	1.38012000
Pd	6.39523500	3.23663000	2.49405200
O	9.31357400	5.08945100	2.94110100
O	7.08513200	5.17531900	3.24709600
O	8.10268500	4.97780900	0.25829000
O	6.05416500	4.10324000	0.62881300
N	6.48773800	2.08778500	4.21562500
N	5.87171600	0.98094600	4.22554100
C	8.24819800	5.58888900	3.43371000
C	8.46785100	6.78784000	4.31601500
H	9.25028400	6.57264900	5.05320600
H	7.54288000	7.07010400	4.82611100
H	8.81885100	7.62726400	3.70344800
C	6.85904300	4.86327200	0.03313100
C	6.31116200	5.75609000	-1.04421900
H	6.13532300	6.75065800	-0.61618500
H	5.36129700	5.36981200	-1.42208800
H	7.03395000	5.86448300	-1.85860800
C	7.35090500	2.32599000	5.31855500
C	8.11942000	1.28169800	5.84031600
H	8.03646700	0.28621500	5.40938200
C	8.99503000	1.53828300	6.88524800
H	9.60665400	0.73256800	7.28545600
C	9.09750800	2.82372100	7.41384700
H	9.78871700	3.02123900	8.23037400
C	8.31309900	3.85487600	6.90111200
H	8.37755600	4.85376000	7.32791500
C	7.44127300	3.61384000	5.84691100
H	6.81483700	4.40430500	5.44024700
C	5.36542900	1.64410500	1.97499700
C	4.77338000	1.35283900	0.75686700
H	4.88114100	2.03750600	-0.08298600
C	4.02358400	0.17897900	0.61933700
H	3.55565100	-0.04020500	-0.33937000
C	3.85952500	-0.71232300	1.68185300
H	3.27313700	-1.61793400	1.54707200
C	4.45323900	-0.44113400	2.90528500
H	4.36319300	-1.12098400	3.75053400

C	5.20003200	0.73396300	3.04646700
N	9.28579200	1.53833100	-0.76093800
N	9.67819800	2.65622900	-0.35947400
C	8.58353200	0.59312600	-0.02165100
C	8.17503000	-0.49774000	-0.81134900
H	8.36524700	-0.45182200	-1.88124900
C	7.57133200	-1.60031100	-0.23226400
H	7.25242300	-2.43530500	-0.85082700
C	7.41715600	-1.64834500	1.15123700
H	6.97667700	-2.52537700	1.62117100
C	7.82440700	-0.57360700	1.94158300
H	7.69231500	-0.61851100	3.02082700
C	8.37549900	0.55990000	1.36816100
H	8.62513900	1.40663800	2.00283700
C	11.52579800	2.49143000	-1.94330600
C	12.44444900	3.07972600	-2.79932600
H	13.18726000	2.46051400	-3.29750200
C	12.42107400	4.45674900	-3.01119600
H	13.13997000	4.91510600	-3.68675900
C	11.47976000	5.24468600	-2.35511700
H	11.45424500	6.32015400	-2.52098400
C	10.57021800	4.67362700	-1.47233500
H	9.83525900	5.29133700	-0.96323300
C	10.58913600	3.29073700	-1.28143600
H	11.54486700	1.42044800	-1.75085200
N	12.34853200	4.62212500	1.92499600
N	12.86940800	3.98168100	0.97869700
C	12.32768700	6.02012400	1.80394500
C	11.91839700	6.71685700	2.94593900
H	11.64805000	6.14643100	3.83144000
C	11.85913500	8.10274500	2.92177100
H	11.54970800	8.65061600	3.80939800
C	12.19661400	8.78805600	1.75691000
H	12.14433600	9.87462300	1.73395700
C	12.60392800	8.09104700	0.61563500
H	12.86814400	8.63691000	-0.28777000
C	12.67689500	6.70910100	0.63276300
H	12.99661100	6.14649700	-0.24112900
C	11.79330600	1.96516400	1.92020700
C	11.82819400	0.58199900	2.12294700
H	11.06537200	0.11904300	2.74547900
C	12.78540000	-0.17807000	1.47097100
H	12.79365600	-1.25680700	1.60270300
C	13.72493800	0.43563100	0.63883000

H	14.47610600	-0.16605800	0.13369000
C	13.71271200	1.81082800	0.46004400
H	14.45118900	2.31254300	-0.16111700
C	12.76192600	2.59810200	1.10854300
N	10.69406400	2.67499600	2.48042600
O	10.36758100	2.38678900	3.60945800

LM4:

Pd	8.49169400	4.23299100	0.83456100
Pd	7.49057100	2.65204500	3.06644300
O	8.86618700	5.60718800	2.28147400
O	7.58721000	4.69122200	3.90000100
O	6.50146500	4.86260200	0.82862300
O	5.69005300	3.25276500	2.18970700
N	9.13200300	1.67049600	3.91140800
N	9.41723700	0.52732400	3.44794600
C	8.29685900	5.60575800	3.43024200
C	8.57039400	6.81511300	4.27813500
H	8.86440900	7.67553500	3.67219800
H	9.39198000	6.57720500	4.96644200
H	7.68944800	7.05497500	4.88065600
C	5.56325100	4.21719800	1.39187300
C	4.17220700	4.63964200	1.01402800
H	4.10153800	5.73073700	0.97882000
H	3.43477700	4.23180300	1.70899400
H	3.96312200	4.26222200	0.00425600
C	9.99635400	2.17197700	4.92095600
C	11.25607800	1.60274100	5.15378400
H	11.58502800	0.76136800	4.55006200
C	12.06195000	2.11730800	6.15559700
H	13.04265700	1.68027500	6.33111900
C	11.62292300	3.18846300	6.93588200
H	12.26322600	3.58779100	7.71942300
C	10.36635400	3.73962800	6.71216400
H	10.01235800	4.56508900	7.32614400
C	9.54999100	3.23776600	5.70456500
H	8.56385900	3.66114100	5.53250800
C	7.41998600	0.91598500	2.16651300
C	6.48901300	0.46420300	1.24642100
H	5.62505800	1.07713500	0.99053700
C	6.66687700	-0.78965300	0.64951600
H	5.93081000	-1.14189500	-0.07252000
C	7.76765600	-1.59157000	0.95650000
H	7.88690000	-2.55977900	0.47645300

C	8.70031800	-1.15750400	1.88779400
H	9.56469200	-1.76141400	2.15927700
C	8.52252100	0.09176000	2.49186100
N	8.37090900	2.90554400	-0.73090200
N	9.29439400	2.05067100	-0.82653000
C	7.24938400	2.73391200	-1.57424500
C	6.50388500	3.86177400	-1.93041600
H	6.79328800	4.84243900	-1.56259200
C	5.41457700	3.70793000	-2.77670300
H	4.83723500	4.58019100	-3.07358000
C	5.06341800	2.44269900	-3.24136700
H	4.19993500	2.32667800	-3.89239600
C	5.81325600	1.32196500	-2.87871100
H	5.53277100	0.33625000	-3.24239600
C	6.91406800	1.46020600	-2.05183800
H	7.51227000	0.60341500	-1.75223400
C	10.58586800	3.56300400	0.61052500
C	11.51486600	3.70375100	1.65328100
H	11.71921000	4.69462500	2.05252000
C	12.18608600	2.58921600	2.13692300
H	12.90957300	2.69723300	2.94128500
C	11.96942000	1.33475500	1.55977500
H	12.52400800	0.47249200	1.92409500
C	11.04864500	1.16243100	0.52778600
H	10.84489700	0.18255200	0.10294900
C	10.32712500	2.26215400	0.08610500
H	10.42426900	4.43935500	-0.05209900

LM5:

Pd	9.03134300	3.71527200	1.07079600
Pd	6.64486500	3.09293500	3.35626800
O	9.41601100	5.01416100	2.60408500
O	8.41961600	3.93057600	4.30717800
O	7.43173900	4.88895500	0.50903100
O	6.23344800	4.92360100	2.43361600
N	6.62665100	1.23071400	4.27833800
N	5.69614200	0.43261700	3.96444300
C	9.16674700	4.81633900	3.84032600
C	9.88175500	5.75211100	4.77511300
H	10.96469700	5.60413300	4.67925100
H	9.57853500	5.57909600	5.81023300
H	9.67165500	6.78981800	4.49424000
C	6.54598100	5.35668400	1.29494300
C	5.76772900	6.52695600	0.76163800

H	5.08133200	6.16771400	-0.01589000
H	6.44012200	7.25337000	0.29551200
H	5.18499400	7.00042100	1.55532900
C	7.59943800	0.72047500	5.18205400
C	7.94125800	-0.63605300	5.17167400
H	7.48497500	-1.30123200	4.44247500
C	8.86774600	-1.10853300	6.08810300
H	9.14607900	-2.16003600	6.07761400
C	9.44250600	-0.24103000	7.01685900
H	10.16722400	-0.61868600	7.73519900
C	9.09307900	1.10589900	7.02338100
H	9.53354900	1.78239600	7.75256900
C	8.17774000	1.59682000	6.10015400
H	7.89989900	2.64639000	6.08882400
C	4.96478900	2.32703400	2.67292900
C	3.99434000	2.90761700	1.87170500
H	4.09327700	3.94048400	1.54073600
C	2.86727500	2.16534300	1.49706700
H	2.10872900	2.63456200	0.87268200
C	2.69721600	0.84242100	1.90820100
H	1.81478200	0.28530100	1.60348800
C	3.65323300	0.24623200	2.71780100
H	3.54721700	-0.77707400	3.07255500
C	4.77414500	0.99035800	3.10125800
N	8.87710500	2.64667700	-0.73975900
N	9.70637100	2.76339900	-1.66372800
C	7.87230300	1.64792900	-0.96095200
C	6.63287100	1.80625500	-0.34505100
H	6.45681100	2.65563500	0.31232600
C	5.62594400	0.88251400	-0.59709300
H	4.64981200	1.01501300	-0.13233300
C	5.86668700	-0.20142200	-1.43544700
H	5.08068700	-0.93051800	-1.62026600
C	7.11819900	-0.36246800	-2.03005800
H	7.31324800	-1.22026200	-2.66994800
C	8.12348700	0.56469500	-1.80775200
H	9.10702000	0.44681700	-2.25585800
C	10.14532300	5.15244200	-1.48995800
C	11.03914900	6.20495800	-1.64790600
H	10.68264600	7.22811000	-1.55152300
C	12.37985600	5.95246400	-1.92947200
H	13.07578700	6.78053900	-2.04398900
C	12.82989300	4.64198100	-2.08909600
H	13.87487300	4.44746500	-2.31865500

C	11.93975200	3.58188000	-1.99061100
H	12.25972700	2.55461000	-2.15468300
C	10.60860800	3.83952700	-1.65306700
H	9.09029700	5.33888800	-1.29630100
N	11.13783600	1.53901300	1.50431100
N	10.82973800	2.75025900	1.55509700
C	10.20265600	0.52751000	1.29192000
C	10.69261700	-0.59793300	0.61163100
H	11.72069000	-0.58686600	0.25568700
C	9.86059300	-1.68249600	0.39299000
H	10.22651600	-2.54566800	-0.15766400
C	8.56211700	-1.67495300	0.90403300
H	7.91242900	-2.53362300	0.74841000
C	8.09637900	-0.57884500	1.62604800
H	7.08372600	-0.58382700	2.02868100
C	8.90672500	0.53001600	1.82154100
H	8.55586000	1.37903400	2.41129000
C	12.79802300	3.09462400	2.96246100
C	13.86014600	3.88743000	3.37225800
H	14.55117600	3.51286900	4.12395100
C	14.03896900	5.15656200	2.82472400
H	14.87724100	5.77207100	3.14409900
C	13.14207200	5.64161100	1.87612700
H	13.27795500	6.63104800	1.44402500
C	12.06349700	4.86597400	1.47492800
H	11.35470000	5.24163900	0.74131900
C	11.90943200	3.58587300	2.00218200
H	12.64264300	2.10399800	3.38235200

TS1:

Pd	8.51998000	4.63121000	0.83729300
Pd	7.29814800	2.85963400	2.77802000
O	8.36577300	6.10059700	2.24246400
O	7.49667900	4.78340200	3.86099700
O	6.47587900	4.93809600	0.40433800
O	5.53769200	3.65734300	2.01136700
N	8.99682500	3.14784500	-0.54343900
N	10.12088500	2.58069800	-0.38401600
N	8.94157700	1.70903300	3.39494900
N	9.09838700	0.59211900	2.81926100
C	7.93142600	5.87072000	3.42690200
C	8.02558600	7.03556500	4.37090200
H	8.98650800	6.96505700	4.89923200
H	7.22642500	6.98592800	5.11540100

H	7.99722000	7.98914700	3.83779400
C	5.49032200	4.43639000	1.02031600
C	4.13530600	4.76880300	0.46317700
H	3.33873300	4.46546700	1.14619100
H	4.02276500	4.22672300	-0.48609900
H	4.06327500	5.83778900	0.24174700
C	8.13272500	2.56934000	-1.49126100
C	7.08780600	3.34976200	-2.00325100
H	6.97439500	4.37985400	-1.67960700
C	6.21881200	2.79367100	-2.92916100
H	5.41088500	3.39568100	-3.33780000
C	6.38620200	1.47194100	-3.33774500
H	5.69475300	1.03711900	-4.05609600
C	7.43506000	0.70127800	-2.83008600
H	7.55249900	-0.33387500	-3.14253300
C	8.31262000	1.24042600	-1.90841200
H	9.11136200	0.64422400	-1.47564200
C	10.57996100	4.46548000	1.10176300
C	11.27473400	4.94270100	2.23849800
H	11.05746700	5.94971100	2.59349200
C	12.28050700	4.18553900	2.81120900
H	12.82884000	4.56648300	3.66859900
C	12.61905300	2.94372300	2.26203800
H	13.43624200	2.37327100	2.69743400
C	11.92937900	2.41800900	1.16493800
H	12.15680300	1.42907100	0.77363500
C	10.89084000	3.14949000	0.61269800
C	9.92586700	2.04456800	4.36467000
C	11.11299300	1.30663300	4.48079300
H	11.29290100	0.46668000	3.81508300
C	12.04832500	1.66521400	5.43882100
H	12.97092900	1.09467900	5.52523600
C	11.81590000	2.75217600	6.28272900
H	12.55583200	3.02798200	7.03091200
C	10.63563100	3.47846800	6.16582200
H	10.44201200	4.32005900	6.82745000
C	9.68754900	3.12950600	5.20885700
H	8.75977100	3.68878900	5.12295200
C	7.06023400	1.22630100	1.71336500
C	6.04842800	0.90983000	0.82023300
H	5.22145500	1.59657800	0.64713600
C	6.09041800	-0.30924800	0.13429800
H	5.29189200	-0.54731100	-0.56687400
C	7.13443600	-1.21650700	0.32167600

H	7.14616700	-2.15432600	-0.22863200
C	8.15437200	-0.91643600	1.21129100
H	8.98014500	-1.60206500	1.39063400
C	8.11230200	0.29959200	1.90523800
N	10.59480500	5.75789100	0.03811900
O	10.82463900	5.48930000	-1.07994400

TS2:

Pd	9.36169100	3.68518600	1.67206800
Pd	6.80163100	3.72653200	3.10638800
O	9.81529700	5.01426300	3.56094900
O	7.77411100	5.03567000	4.50995600
O	8.39079500	5.17731500	0.67944400
O	6.50707400	5.39182400	1.89972900
N	6.75353500	1.95464300	4.18242200
N	6.18845400	0.95931200	3.64547800
C	9.00027900	5.33638400	4.46665900
C	9.52095200	6.13954100	5.63425600
H	9.50483500	5.50300600	6.52856600
H	8.86015000	6.99065100	5.83018600
H	10.54189800	6.48930300	5.46222500
C	7.29814700	5.74701800	0.98807600
C	6.90838300	6.91137900	0.12766200
H	7.79382000	7.48062400	-0.17469100
H	6.19202800	7.55490700	0.64456800
H	6.42970700	6.52673000	-0.78388500
C	7.37467900	1.71574000	5.43871000
C	7.94249600	0.46834000	5.71779900
H	7.92320500	-0.30983200	4.95844200
C	8.55093800	0.25650400	6.94506500
H	9.00694200	-0.70777500	7.15930100
C	8.58622800	1.27568000	7.89645600
H	9.06525900	1.10506000	8.85834300
C	8.01396000	2.51233900	7.61355600
H	8.03390500	3.30696200	8.35648800
C	7.41440600	2.74329200	6.38073300
H	6.97701500	3.70919500	6.14500600
C	5.85441900	2.53229400	1.85480500
C	5.39962100	2.78119400	0.56867000
H	5.50618900	3.77053200	0.12388200
C	4.78192400	1.75862600	-0.16197600
H	4.42834600	1.96609600	-1.17056400
C	4.60503500	0.48479200	0.37950700
H	4.12020200	-0.29329000	-0.20469000

C	5.05062300	0.21496500	1.66530600
H	4.93829900	-0.76994800	2.11562100
C	5.67847400	1.23374200	2.38963600
N	9.86674200	1.18262400	-0.85611700
N	9.41479600	2.33163200	-0.66166900
C	9.67662700	0.21286800	0.14321000
C	10.56909800	-0.86379300	0.11755800
H	11.35148900	-0.87639600	-0.63937600
C	10.46029400	-1.87089700	1.06611200
H	11.16704500	-2.69780300	1.06427800
C	9.43459600	-1.82715600	2.00917800
H	9.33562600	-2.62433800	2.74294400
C	8.51140100	-0.78087400	1.99403700
H	7.68151500	-0.77263100	2.70016800
C	8.62494300	0.24283900	1.06468800
H	7.88789300	1.04451600	1.01612500
C	10.78870900	3.11017400	-2.55148900
C	10.96347400	3.98107000	-3.61695800
H	11.86607900	3.91942200	-4.22186700
C	9.99009300	4.93627700	-3.90955200
H	10.13216900	5.61787600	-4.74569200
C	8.84209000	5.02568900	-3.12727300
H	8.07758800	5.76496600	-3.35802100
C	8.66568100	4.17074500	-2.04605900
H	7.77446400	4.22251100	-1.42652600
C	9.63590000	3.20941400	-1.76559900
H	11.53735000	2.35866100	-2.30599300
N	11.42619500	4.44728200	0.85664900
N	12.32229300	3.64830700	0.49819400
C	11.44415900	5.70982400	0.23447600
C	10.79242700	6.75038900	0.90749000
H	10.33029700	6.54692900	1.87160600
C	10.78621700	8.02051400	0.35027100
H	10.29716600	8.83897600	0.87398700
C	11.39650200	8.24251900	-0.88255500
H	11.37049500	9.23477500	-1.32819600
C	12.04400000	7.20064100	-1.55017500
H	12.51436200	7.37991100	-2.51476300
C	12.08442900	5.93509000	-0.99272900
H	12.57790500	5.11057000	-1.50030300
C	11.63745000	1.83339200	2.17958600
C	12.06110500	0.65113200	2.80701400
H	11.44018900	0.24178300	3.59931600
C	13.20408500	-0.00474600	2.38934800

H	13.50308800	-0.92950000	2.87522000
C	13.94438700	0.51300000	1.32854400
H	14.83600500	-0.00130700	0.97971600
C	13.55583300	1.70216300	0.72296100
H	14.15137900	2.14851200	-0.06933300
C	12.42130100	2.40403500	1.13250700
N	10.39743300	2.35364400	2.61606000
O	9.90964200	1.93232300	3.63890100

TS3:

Pd	8.93556000	3.47329500	0.91626000
Pd	7.16895200	2.76358100	3.42854900
O	9.70054500	4.59268000	2.50066200
O	7.92672100	4.74614900	3.87023000
O	7.27657100	4.73846100	0.71682600
O	5.82970200	3.70333200	2.12068200
N	8.35586600	1.61050600	4.68074700
N	8.17125200	0.36143400	4.68279800
C	9.04545500	5.12595100	3.45177500
C	9.69633000	6.31707200	4.09776300
H	9.53114400	7.19971200	3.46696200
H	10.77813100	6.16190500	4.17408600
H	9.26854900	6.50225100	5.08752000
C	6.13336500	4.55450300	1.24673600
C	5.04833000	5.47662300	0.76280600
H	5.18395400	6.45632800	1.23690200
H	4.06327100	5.09021400	1.03517200
H	5.11902300	5.61824200	-0.31955200
C	9.46865400	2.07292000	5.43434700
C	10.61999300	1.28956200	5.55950000
H	10.67317600	0.33388900	5.04440000
C	11.68400500	1.76307100	6.31307800
H	12.58662600	1.16253300	6.40566400
C	11.60294300	3.00652700	6.94202900
H	12.44159200	3.37116900	7.53239500
C	10.45211300	3.77874000	6.81493100
H	10.38085000	4.74385100	7.31256900
C	9.38062700	3.31656500	6.05891000
H	8.47153700	3.90419400	5.95887000
C	6.36408500	0.98168200	3.22148900
C	5.21016200	0.61540200	2.54616000
H	4.62496400	1.36426700	2.01180200
C	4.79407500	-0.72169600	2.55654000
H	3.88016200	-0.99694700	2.03085800

C	5.52519200	-1.70540900	3.22329800
H	5.18645500	-2.73848000	3.21166500
C	6.67654300	-1.35678600	3.91482600
H	7.25967600	-2.09345100	4.46404300
C	7.08152800	-0.01836500	3.92169700
N	8.57761000	2.96642100	-1.09374800
N	9.17116600	3.52380600	-2.04109300
C	7.65859900	1.93689300	-1.46492800
C	6.85355000	1.38485100	-0.46983500
H	6.93088500	1.74949800	0.55694600
C	5.97748300	0.35673800	-0.79333800
H	5.35378600	-0.07898400	-0.01581800
C	5.91080900	-0.11992800	-2.09820000
H	5.23009900	-0.93142100	-2.34585300
C	6.72078400	0.43670600	-3.08917500
H	6.67237300	0.06049000	-4.10845900
C	7.59308800	1.46678900	-2.78198700
H	8.23817900	1.90018500	-3.54078700
C	9.81833300	5.65869000	-0.95373100
C	10.72714600	6.70786300	-0.90280900
H	10.53946600	7.54091900	-0.22837900
C	11.86129200	6.70288100	-1.71254900
H	12.56963900	7.52715100	-1.65824700
C	12.07719500	5.65864400	-2.61152700
H	12.95140200	5.66410600	-3.25809100
C	11.15906400	4.62315200	-2.70261400
H	11.28836500	3.81451300	-3.41927500
C	10.05209000	4.59675700	-1.84296900
H	8.90052400	5.70366200	-0.37330600
N	11.80126900	2.67275200	0.94245000
N	12.15112700	2.40961000	-0.24869000
C	12.38225700	3.67391100	1.74713300
C	12.46677300	3.47440600	3.12668000
H	12.06404500	2.57237800	3.57909900
C	13.08128400	4.45491800	3.89323800
H	13.16442800	4.31350600	4.96907000
C	13.58303100	5.60867800	3.29454500
H	14.05564900	6.37385300	3.90658700
C	13.48574200	5.78915700	1.91441200
H	13.87507900	6.69238100	1.44915500
C	12.89057900	4.81703600	1.12768100
H	12.80128700	4.93584900	0.04956900
C	10.42526900	1.00005700	0.25308000
C	9.61328400	-0.10974800	0.10734900

H	8.91429500	-0.40626800	0.88729200
C	9.74004300	-0.79983000	-1.09261000
H	9.12698200	-1.68186500	-1.26258000
C	10.63046100	-0.38464100	-2.10220100
H	10.67914800	-0.95382400	-3.02679800
C	11.46020400	0.70853300	-1.92926700
H	12.18095800	1.01770900	-2.68167800
C	11.36232200	1.38506300	-0.70850700
N	10.47323400	1.90392100	1.34426500
O	10.28774900	1.53385700	2.52931000

TS4:

Pd	9.11337600	4.51140200	2.00335400
Pd	7.88194800	2.49868000	3.69337000
O	9.95275400	5.26254800	3.72040900
O	9.29241500	3.55555000	5.04770800
O	7.33562800	5.53609900	2.58118700
O	6.58847300	4.07195500	4.12976800
N	8.81809500	0.68864100	3.16963300
N	8.21630600	-0.03518800	2.32176500
C	9.89017000	4.63312000	4.83240300
C	10.66295800	5.26829700	5.95655600
H	11.72721400	5.03183200	5.82790000
H	10.33161200	4.87980100	6.92268600
H	10.56218700	6.35733200	5.92953600
C	6.55440800	5.17070800	3.50174600
C	5.43206800	6.10612800	3.85936200
H	5.68890400	7.13983900	3.61377500
H	5.17612800	6.01623300	4.91869000
H	4.54590700	5.82240200	3.27563900
C	10.04196200	0.16469100	3.66883700
C	10.63720100	-0.97221700	3.10315400
H	10.16391100	-1.46051000	2.25593600
C	11.81902600	-1.46096300	3.63549000
H	12.27930100	-2.34366300	3.19599100
C	12.41930700	-0.82815300	4.72526500
H	13.34753200	-1.21849300	5.13749900
C	11.82544900	0.29822400	5.28354300
H	12.28443000	0.79337400	6.13672200
C	10.63977800	0.80048000	4.75731100
H	10.17662200	1.68393000	5.18862200
C	6.56733000	1.70796100	2.47296600
C	5.33338300	2.20955300	2.08634900
H	4.95994000	3.14441900	2.50155300

C	4.55652600	1.50260500	1.16300800
H	3.59021800	1.90671900	0.86440100
C	4.99903100	0.29870300	0.61148200
H	4.37900400	-0.23182000	-0.10735000
C	6.22965500	-0.21839700	0.98589700
H	6.60362000	-1.15820700	0.58390900
C	7.00752000	0.48491300	1.91437100
N	8.53720500	3.58677700	0.23482000
N	9.25661300	2.61993600	-0.15521300
C	7.31609200	3.78584000	-0.45011200
C	6.67652100	5.02104700	-0.30651800
H	7.12116600	5.79203300	0.31773300
C	5.47499800	5.24489300	-0.96446400
H	4.97773300	6.20674000	-0.86122000
C	4.91190600	4.24347800	-1.75069200
H	3.96359400	4.41800000	-2.25463400
C	5.55761900	3.01360200	-1.89264500
H	5.10976200	2.22869600	-2.49838400
C	6.76062700	2.77816700	-1.25064700
H	7.26076000	1.81753300	-1.33614400
C	10.83763200	3.53577000	1.44345900
C	11.97462600	3.32268900	2.24003000
H	12.30406000	4.11115200	2.91763800
C	12.69836100	2.13877300	2.13719900
H	13.59113600	1.99269500	2.74086900
C	12.28603900	1.13299300	1.25963300
H	12.85871700	0.21053600	1.19298600
C	11.14748700	1.29183200	0.47261700
H	10.80253800	0.50535100	-0.19590600
C	10.42236100	2.47308200	0.58310500
N	10.99978200	5.64327900	-0.31506700
N	11.02148700	5.27304500	-1.51326000
C	10.23933800	6.79469200	0.00766300
C	11.82429800	4.16354100	-1.79823600
C	10.44878400	7.33338800	1.27890400
C	9.30640700	7.35356600	-0.87039300
C	11.43289200	3.37986300	-2.89154100
C	12.99888000	3.87133800	-1.08973400
H	11.18290000	6.87859800	1.94314300
C	9.71667700	8.44429400	1.67759500
C	8.57716800	8.45786900	-0.45768300
H	9.15699600	6.90853900	-1.85144100
C	12.17678300	2.25943500	-3.22736300
H	10.53136900	3.65274500	-3.43665600

C	13.75203500	2.76890900	-1.45869400
H	13.32738100	4.53513800	-0.29192200
H	9.87541800	8.87035900	2.66550600
C	8.77805800	9.00129700	0.81302300
H	7.84096800	8.89648200	-1.12783300
H	11.86624100	1.63105000	-4.05856500
C	13.33533700	1.95546600	-2.51351900
H	14.67280600	2.54158600	-0.92524300
H	8.19812700	9.86655700	1.12690400
H	13.93165200	1.09006700	-2.79473500
H	11.00584000	4.66588200	0.70700800

TSA:

Pd	7.98451700	5.02536800	0.84495400
Pd	6.99530400	2.86212600	2.70206900
O	8.90473000	5.65369000	2.62491200
O	7.70386900	4.41648600	4.07742900
O	6.10229600	5.73528400	1.34135200
O	5.26475400	4.00898400	2.53171100
N	10.15532200	2.99472300	0.09726000
N	11.08123500	2.85857200	0.90908900
N	8.49218300	1.40899100	2.80523600
N	8.31550900	0.34988900	2.13287400
C	8.54459400	5.29650000	3.79606300
C	9.26254300	6.01189600	4.90955800
H	9.15066400	7.09569800	4.80040600
H	10.33389500	5.78488000	4.83841000
H	8.88546400	5.69114100	5.88421000
C	5.19438700	5.15414100	2.00874100
C	3.91976800	5.92390100	2.20421600
H	3.09530200	5.25436500	2.46115500
H	3.67860600	6.50265000	1.30792700
H	4.06318200	6.63182000	3.02979500
C	9.55920500	1.97536800	-0.64775800
C	8.35034800	2.22659000	-1.30593000
H	7.86659600	3.19650200	-1.22858700
C	7.78019200	1.21576600	-2.06438100
H	6.83689700	1.39455300	-2.57381700
C	8.41275100	-0.02031800	-2.17020500
H	7.96020900	-0.80768200	-2.76882800
C	9.62583800	-0.25584600	-1.51937700
H	10.11934700	-1.21968500	-1.61499600
C	10.21154000	0.73774600	-0.75625200
H	11.16700200	0.58623600	-0.25987300

C	10.89739300	5.17379700	0.69711800
C	11.25849000	6.48069000	0.92695500
H	10.72828400	7.30257200	0.45142100
C	12.31352100	6.69165200	1.82036700
H	12.63819400	7.71011200	2.01829500
C	12.94990600	5.63143700	2.47024600
H	13.75828700	5.83911700	3.16591100
C	12.57492100	4.31399700	2.22568000
H	13.06350900	3.46670000	2.70231700
C	11.54348300	4.10685600	1.31939800
C	9.72532600	1.49305300	3.51020100
C	10.84155000	0.75833200	3.09137300
H	10.76321500	0.12067200	2.21467300
C	12.03193400	0.86442900	3.79355800
H	12.90115300	0.29906700	3.46405700
C	12.11871200	1.69398300	4.91236200
H	13.05624100	1.77337600	5.45881700
C	11.00402700	2.41528200	5.32984500
H	11.06075400	3.05145800	6.21081000
C	9.80478900	2.31879400	4.63270000
H	8.92468800	2.86442100	4.96237500
C	6.27083000	1.46137600	1.53368300
C	5.07228200	1.44839200	0.83827800
H	4.39012300	2.29435300	0.90018500
C	4.73285700	0.32856000	0.06930900
H	3.78636200	0.32331300	-0.46904700
C	5.57668900	-0.77961100	-0.01504100
H	5.29002800	-1.63840000	-0.61734900
C	6.78179100	-0.78164600	0.67196200
H	7.46714200	-1.62627300	0.62660200
C	7.12459900	0.33656800	1.44019100
N	9.73924900	4.75852600	-0.05230500
O	9.50445300	5.13811600	-1.20692700

4a:

Pd	3.44720400	1.09373200	8.14165200
Pd	1.70680600	0.07172800	11.63693000
I	1.85029400	2.28617800	10.12873700
I	3.30408100	-1.12061300	9.65003700
N	1.12107100	-1.21983600	14.16801300
N	1.56644700	-1.49352600	13.02042500
N	3.58722100	2.65890700	6.75804500
N	4.03222400	2.38514100	5.61033100
C	0.72607400	0.94465900	13.15240600

C	0.18122200	2.21352900	13.29052400
C	-0.41363100	2.60225200	14.49676700
C	-0.46987400	1.74235100	15.59165600
C	0.06259000	0.46773500	15.48074800
C	0.64720500	0.07427200	14.27231700
C	2.07657400	-2.81109700	12.85586300
C	1.75574800	-3.50889400	11.69169200
C	2.23282700	-4.80281900	11.52238000
C	3.04374200	-5.38233300	12.49586500
C	3.36571000	-4.67357400	13.65172000
C	2.87773800	-3.38770900	13.84192800
C	3.07727900	3.97653000	6.92274700
C	3.39848700	4.67426100	8.08685300
C	2.92163100	5.96824900	8.25630900
C	2.11054100	6.54788500	7.28304200
C	1.78817200	5.83918400	6.12726300
C	2.27592900	4.55325900	5.93690200
C	4.42753300	0.22071500	6.62596700
C	4.50601700	1.09101000	5.50595600
C	5.09021800	0.69744500	4.29735900
C	5.62266100	-0.57717300	4.18638200
C	5.56682200	-1.43697300	5.28137100
C	4.97237200	-1.04815200	6.48778200
H	3.11999300	-2.81654800	14.73531000
H	4.00343000	-5.12423200	14.40914500
H	3.42859900	-6.38983600	12.35257700
H	1.96980400	-5.36027300	10.62589300
H	1.11501100	-3.04324600	10.94468500
H	0.03414000	-0.24009500	16.30690400
H	-0.93093800	2.06695200	16.52131400
H	-0.83936100	3.60151400	14.57559300
H	0.20427000	2.92506800	12.46685600
H	4.03931600	4.20849500	8.83371100
H	3.18494800	6.52565100	9.15274100
H	1.72585400	7.55543700	7.42644400
H	1.15030200	6.28993200	5.37001800
H	2.03337400	3.98214200	5.04357400
H	5.11837300	1.40519900	3.47112600
H	6.08339700	-0.90185800	3.25659000
H	5.99254900	-2.43623100	5.20249100
H	4.94961900	-1.75961200	7.31152600

LM1':

Pd	3.48661300	1.13559400	8.24321700

Pd	1.66410300	0.07587000	11.67233400
I	1.69040200	2.26253400	10.09923600
I	3.08799500	-1.18128200	9.55835500
N	1.22061500	-1.22483700	14.22371100
N	1.53879700	-1.49643400	13.03546600
N	3.62634900	2.71552100	6.80608400
N	4.17339300	2.41166500	5.71600300
C	0.91618800	0.98721100	13.28877600
C	0.51498900	2.29741100	13.49217700
C	0.08901000	2.71368500	14.76054800
C	0.06436700	1.83924400	15.84362300
C	0.45890200	0.52212500	15.66450000
C	0.87756600	0.10094000	14.39888600
C	1.89740500	-2.84551100	12.76892900
C	1.43999800	-3.42895900	11.58660100
C	1.77134600	-4.74942600	11.30769100
C	2.56690300	-5.47128700	12.19480400
C	3.01924700	-4.87841400	13.37230700
C	2.68355400	-3.56430700	13.66977000
C	2.93900100	3.95670100	6.85494000
C	3.02961000	4.73546800	8.00955200
C	2.36909300	5.95551400	8.05659800
C	1.60699700	6.37900800	6.96949000
C	1.51728800	5.59087000	5.82328100
C	2.18583900	4.37771700	5.75566300
C	4.62500500	0.27312100	6.78298100
C	4.74113700	1.15774900	5.67921300
C	5.45777300	0.78806800	4.53545600
C	6.07604200	-0.45225300	4.49308200
C	5.97639900	-1.30918300	5.58455500
C	5.24915500	-0.95557100	6.73150200
H	3.03143500	-3.08390700	14.58130300
H	3.64124200	-5.44359000	14.06284400
H	2.83355900	-6.50143900	11.96894600
H	1.40134400	-5.21599700	10.39741800
H	0.79443000	-2.85849900	10.91996000
H	0.44850300	-0.19621500	16.48165200
H	-0.26682700	2.18535200	16.81943300
H	-0.22909100	3.74625600	14.89406000
H	0.51897900	3.02266700	12.68069700
H	3.63467900	4.40460600	8.85161800
H	2.44879400	6.57657900	8.94548700
H	1.07855300	7.32842900	7.01632900
H	0.91845900	5.92106800	4.97777300

H	2.12514500	3.74448100	4.87381200
H	5.51273800	1.48765200	3.70442400
H	6.64069200	-0.75057200	3.61389000
H	6.47103500	-2.27802000	5.55845100
H	5.19837200	-1.65922000	7.55983400
N	5.12905100	1.69793300	9.37675700
O	5.97536200	2.27200000	8.89458800

LM1'-1a:

Pd	3.00173800	1.03640700	8.46100300
Pd	1.56582100	-0.24660600	11.95972800
I	1.03452100	1.86667500	10.38808200
I	2.61583700	-1.39922700	9.59918300
N	1.88147200	-1.48768100	14.55969400
N	1.93956300	-1.77091200	13.33339700
N	3.47957600	2.83652200	7.36901100
N	4.29418800	2.68826200	6.42250100
C	1.11392300	0.64033000	13.69657900
C	0.62239000	1.90569400	13.97499200
C	0.46239800	2.32207600	15.30334800
C	0.79602400	1.49369700	16.37144800
C	1.28622700	0.22131100	16.11886200
C	1.43977100	-0.20029500	14.79453000
C	2.37075000	-3.08440500	13.00565700
C	1.71755400	-3.75367800	11.97004500
C	2.11367400	-5.04317200	11.63697300
C	3.16640100	-5.64839100	12.32048400
C	3.81402900	-4.96982700	13.35111200
C	3.41706900	-3.68690900	13.70428600
C	2.88775700	4.12266900	7.49142200
C	2.59855100	4.62603500	8.76036400
C	2.03126900	5.88749800	8.87985800
C	1.74631300	6.63644900	7.74045300
C	2.05041200	6.13200300	6.47717800
C	2.62640500	4.87882400	6.34533400
C	4.43458500	0.37725500	7.16597700
C	4.82657100	1.42695500	6.29703600
C	5.80141400	1.21795300	5.31347300
C	6.40225700	-0.02612100	5.20432600
C	6.02751700	-1.04726500	6.07123700
C	5.04407300	-0.85390400	7.05358400
H	3.91278700	-3.14194000	14.50422300
H	4.63627400	-5.44343700	13.88294800
H	3.48171400	-6.65379900	12.05028600

H	1.59475300	-5.57730800	10.84402300
H	0.87959800	-3.27451700	11.46497800
H	1.55095300	-0.46181900	16.92331900
H	0.66814300	1.83969200	17.39400400
H	0.06651100	3.31779000	15.49632300
H	0.35199000	2.59378600	13.17638300
H	2.84185600	4.04867900	9.64870700
H	1.81834700	6.28852200	9.86812900
H	1.28880000	7.61848300	7.83805500
H	2.85734100	4.46395300	5.36781200
H	6.06859800	2.04679600	4.66160400
H	7.16327100	-0.20073200	4.44832400
H	6.49568100	-2.02662900	5.99066800
H	4.78244100	-1.68769800	7.70060800
N	4.41698100	1.41692400	9.98131600
O	5.37010800	1.96636700	9.71024200
N	1.82237100	0.18238200	4.88217700
N	1.36068100	0.69132900	5.92921400
C	2.39105600	-1.09917800	4.99474500
C	3.29038700	-1.46203600	3.98769300
H	3.53194700	-0.73302400	3.21579400
C	3.86772400	-2.72433000	3.99872200
H	4.58309200	-3.00100800	3.22706500
C	3.51002800	-3.64263200	4.98419900
H	3.94317400	-4.64088000	4.97913300
C	2.57465100	-3.29617700	5.95994400
H	2.27155900	-4.02739600	6.70793200
C	2.01330000	-2.02722700	5.97256000
H	1.26110300	-1.75341300	6.70973000
C	0.70319200	2.64425800	4.52800900
C	-0.03344200	3.81388800	4.42578200
H	-0.00494500	4.38980400	3.50256300
C	-0.80294300	4.26399500	5.50194700
H	-1.37056200	5.18825700	5.41268100
C	-0.84027300	3.53546900	6.68636700
H	-1.44111100	3.87668500	7.52765100
C	-0.10736500	2.35962400	6.79334400
H	-0.13695000	1.75271500	7.69659200
C	0.66704900	1.91470800	5.72297700
H	1.30991300	2.28445100	3.70058200
H	1.82336500	6.71349500	5.58601300

LM1’-BF4:

Pd	2.98235300	0.86238000	8.04840500

Pd	1.36572300	-0.10327100	11.49478900
I	0.96215800	1.88458300	9.73649000
I	2.52636500	-1.50327200	9.32259500
N	1.50510000	-1.12529500	14.20403700
N	1.62538800	-1.51387500	13.01147800
N	3.48741200	2.59364900	6.90428200
N	4.29531700	2.41129600	5.96288700
C	0.83322700	0.93631800	13.12144700
C	0.34906500	2.22756500	13.26944400
C	0.11805800	2.75281600	14.54723100
C	0.37239100	2.01011100	15.69736900
C	0.85352000	0.71541600	15.57726200
C	1.07715800	0.18519200	14.30276800
C	2.04785200	-2.85869800	12.82581200
C	1.41852300	-3.61508100	11.83671900
C	1.80997700	-4.93306100	11.63647000
C	2.83614100	-5.48027600	12.40401500
C	3.46015700	-4.71498900	13.38730900
C	3.06542700	-3.40183900	13.60945400
C	2.87194800	3.87277800	6.97672300
C	2.81655100	4.53562200	8.20165700
C	2.24373400	5.79971200	8.25793100
C	1.71210600	6.37691500	7.10668800
C	1.75852200	5.69524400	5.89173300
C	2.33934500	4.43820800	5.81730900
C	4.37834200	0.11703300	6.76323500
C	4.79767400	1.13109000	5.86772600
C	5.73152100	0.85876200	4.86308900
C	6.25138700	-0.42173200	4.74778700
C	5.83453800	-1.41489000	5.62769400
C	4.89353200	-1.15473000	6.63595900
H	3.54248700	-2.78745600	14.36966200
H	4.26234300	-5.14299700	13.98455100
H	3.15043900	-6.50806000	12.23574100
H	1.31040700	-5.53374300	10.87965000
H	0.60689200	-3.17496900	11.25883700
H	1.05785300	0.09566800	16.44809600
H	0.19000100	2.43942000	16.67937100
H	-0.26973200	3.76644100	14.63534300
H	0.13892200	2.85251600	12.40364200
H	3.24630600	4.08382700	9.09356500
H	2.21558900	6.33563200	9.20389300
H	1.25398800	7.36233800	7.15803600
H	1.33008300	6.14279700	4.99772500

H	2.35331000	3.86105100	4.89682500
H	6.02031900	1.66053300	4.18705800
H	6.97785500	-0.64849600	3.97195400
H	6.24304800	-2.42003500	5.54235400
H	4.59508400	-1.96326600	7.29943500
N	4.36671300	1.28767300	9.52059400
O	5.35112100	1.78425600	9.22108200
B	0.85440400	1.08529500	5.50570300
F	1.37672000	0.15686700	6.49193100
F	1.91259200	1.45106900	4.67679000
F	-0.13291800	0.43745600	4.80242200
F	0.36985900	2.18732900	6.18960600

LM2’:

Pd	4.02689800	1.20275500	8.74636800
Pd	1.60257300	-0.22473000	11.60331100
I	1.65066800	1.81882300	9.85487900
I	4.02831900	-0.95807600	10.32523300
N	0.65561000	-1.42754200	14.06196500
N	1.49011800	-1.63009200	13.14001200
N	3.98819700	2.80415700	7.39455700
N	4.23061200	2.68494900	6.17533000
C	-0.00162700	0.40027400	12.61998200
C	-0.88794600	1.43673200	12.37462800
C	-1.91222900	1.71706500	13.28898300
C	-2.06384200	0.98041600	14.46073500
C	-1.19217900	-0.06486500	14.72594500
C	-0.17566700	-0.35393200	13.81064600
C	2.39979800	-2.70409300	13.33515600
C	2.69635700	-3.52873400	12.24954000
C	3.58350700	-4.58425100	12.42129300
C	4.18270100	-4.79839700	13.66079500
C	3.88064400	-3.96787800	14.73829800
C	2.98309800	-2.91986300	14.58429300
C	3.48168100	4.07121800	7.79439700
C	3.80572600	4.53106300	9.07258800
C	3.34126600	5.77370000	9.47863500
C	2.53209600	6.52635200	8.63004900
C	2.20075800	6.04931400	7.36098700
C	2.68368000	4.82486300	6.92828500
C	5.61898200	0.58123300	6.35860900
C	4.82824200	1.53151100	5.65969400
C	4.77880400	1.44826300	4.26987300
C	5.43639000	0.42834100	3.58924700

C	6.22125900	-0.48963700	4.28377600
C	6.33083500	-0.39928100	5.66272100
H	2.73870400	-2.25736000	15.41142600
H	4.35047700	-4.13432200	15.70520100
H	4.88917900	-5.61571300	13.78791900
H	3.80606400	-5.24179800	11.58387400
H	2.20573300	-3.35847900	11.29216600
H	-1.27723200	-0.67018400	15.62605700
H	-2.86199300	1.21959000	15.15883400
H	-2.60072100	2.53199700	13.07206700
H	-0.81597500	2.04647400	11.47515600
H	4.43827600	3.93056100	9.72447600
H	3.60582300	6.15200900	10.46277800
H	2.15153100	7.48989500	8.96123100
H	1.55956900	6.63638500	6.70801600
H	2.43561100	4.43023100	5.94635000
H	4.22074400	2.21294500	3.73574800
H	5.35341700	0.36562900	2.50769700
H	6.74861000	-1.27638600	3.75169800
H	6.92967100	-1.10519800	6.23198100
N	5.69022700	0.57552900	7.76890000
O	6.64797900	0.06727100	8.29730400

LM3':

Pd	3.11766900	1.16264400	8.13535200
Pd	2.20892600	-0.00510200	11.35392900
I	0.70870900	0.54574500	9.18600000
I	4.10247300	-0.95871300	9.46202800
N	2.63967100	-0.02451500	14.22344700
N	2.99725100	-0.63891000	13.18286700
N	5.47329100	2.84490200	9.57929800
N	5.02160400	3.92570100	9.12462500
C	1.27102500	1.19496600	12.64335700
C	0.41491300	2.26023600	12.40894200
C	0.02648100	3.09013900	13.46958800
C	0.47995100	2.87316200	14.76848700
C	1.34320300	1.81760700	15.02324900
C	1.74294700	0.99274200	13.96733500
C	3.91437100	-1.70855800	13.36363700
C	3.81049900	-2.82174400	12.52923600
C	4.69155700	-3.88324800	12.69489500
C	5.68047700	-3.82440100	13.67392400
C	5.77500000	-2.71041900	14.50675300
C	4.88939700	-1.65220900	14.36262400

C	5.31039100	2.65379500	10.96239700
C	6.02474000	1.58621100	11.51496400
C	5.93626800	1.33018000	12.87702200
C	5.12532000	2.12928600	13.67899800
C	4.40320100	3.19049800	13.12469100
C	4.49259300	3.45909900	11.77020000
C	5.25845300	2.96114200	6.85271400
C	5.12027100	4.05286800	7.74034900
C	5.15137000	5.34761300	7.22551500
C	5.37272300	5.55907500	5.87195500
C	5.57544600	4.47731300	5.01162200
C	5.52607200	3.18042100	5.49795600
H	4.94378000	-0.77727200	15.00631500
H	6.54599500	-2.66434100	15.27272000
H	6.38014700	-4.64909600	13.79030100
H	4.60437600	-4.75924900	12.05621900
H	3.02209100	-2.86430500	11.77950700
H	1.73333200	1.62295600	16.02042100
H	0.16242900	3.52807500	15.57598500
H	-0.65170400	3.91744700	13.26664700
H	0.03443500	2.46773300	11.41042200
H	6.64530400	0.97675900	10.86075100
H	6.49973000	0.50708100	13.31205200
H	5.04993000	1.93057700	14.74765100
H	3.77127700	3.80911800	13.75971000
H	3.95232000	4.29001100	11.32298400
H	5.02213300	6.17451400	7.92074500
H	5.39779500	6.57333400	5.48177100
H	5.74659900	4.64566500	3.95131300
H	5.60724400	2.32343100	4.83185900
N	4.96765900	1.62575800	7.25302100
O	5.61727900	0.74436500	6.74119700
N	2.23386200	2.69312800	6.92201900
N	2.10745800	2.61427200	5.68247600
C	1.88732700	3.95912200	7.49706900
C	2.45008700	1.45074900	4.98323600
C	1.93077300	4.10205100	8.88482100
C	1.56315700	5.05190300	6.68348600
C	2.22436100	0.13886600	5.42595900
C	3.02218900	1.68251400	3.72438200
H	2.21388200	3.25933900	9.51536500
C	1.62525300	5.32874000	9.46117200
C	1.27527800	6.27297500	7.26813800
H	1.55960200	4.93386000	5.60392600

C	2.65075500	-0.92352200	4.64167900
H	1.67413100	-0.05324500	6.34340900
C	3.47597200	0.61320000	2.96723300
H	3.12934900	2.70941200	3.37952400
H	1.64466800	5.43225300	10.54418900
C	1.29878600	6.41472100	8.65679600
H	1.02915200	7.12442600	6.63791300
H	2.46486500	-1.94196800	4.97526900
C	3.29802200	-0.68985000	3.42925400
H	3.95111000	0.79300300	2.00593500
H	1.06708500	7.37642100	9.10910600
H	3.63610000	-1.52992700	2.82683800

LM4’:

Pd	3.41253100	1.11135600	8.26152100
Pd	1.56221000	-0.22265900	11.54407000
I	1.25396700	1.71459200	9.70959700
I	3.53268900	-1.11529100	9.71942300
N	1.22020400	-1.27243600	14.22031200
N	1.78517000	-1.55122500	13.12931400
C	0.30401200	0.53404600	12.89932500
C	-0.56686000	1.60886100	12.82821200
C	-1.31817500	1.97915300	13.95170400
C	-1.20822600	1.29400600	15.15850400
C	-0.34548600	0.21203900	15.25293000
C	0.39863000	-0.16560700	14.13152200
C	2.67403600	-2.65957200	13.13599300
C	2.65479500	-3.53478800	12.04865400
C	3.52219600	-4.62036400	12.03645800
C	4.41296700	-4.81477800	13.09016200
C	4.42507600	-3.93362500	14.16961800
C	3.55224400	-2.85425200	14.20233900
H	3.55162900	-2.15279700	15.03330700
H	5.12294300	-4.08561000	14.98993900
H	5.09925400	-5.65848800	13.07088200
H	3.49946000	-5.31741800	11.20175100
H	1.93576300	-3.38202200	11.24455600
H	-0.23471700	-0.35448600	16.17521300
H	-1.79825200	1.60069400	16.01838900
H	-2.00198000	2.82236700	13.87129700
H	-0.69408300	2.17957200	11.90938000
N	3.51310500	2.77622500	6.97050000
N	4.37183500	2.65161900	6.06980000
C	2.79369100	4.00731000	6.97820400

C	5.07497400	1.42604000	6.20796100
C	1.40199500	3.98139900	7.04103000
C	3.50338900	5.20612900	6.91009700
C	5.59141200	1.04035400	7.47201300
C	5.21557100	0.61699500	5.08819700
H	0.87101500	3.03159500	7.03801200
C	0.71012000	5.18548100	7.03129000
C	2.79770100	6.40138500	6.93033300
H	4.58987500	5.18741000	6.86623400
C	6.23612200	-0.20651900	7.57373200
H	5.75072400	1.79482900	8.24908800
C	5.82927900	-0.62281800	5.23301100
H	4.82015400	0.94997200	4.13193100
H	-0.37683900	5.18016100	7.05305000
C	1.40620500	6.39208600	6.99066100
H	3.33790300	7.34447400	6.89689300
H	6.67987100	-0.49669300	8.52354500
C	6.32605400	-1.04099600	6.47122200
H	5.92623300	-1.27239000	4.36656100
H	0.85882500	7.33180400	7.00187800
H	6.81515200	-2.00748000	6.55957100

LM5':

Pd	3.04474600	0.67078900	8.24855100
Pd	1.54095700	-0.07485900	11.18820000
I	0.60136100	1.43969900	9.15073800
I	2.50092100	-1.80199600	9.15338400
N	2.32453600	-0.47403100	13.94677700
N	2.18719500	-1.11838400	12.87335400
C	1.33460800	1.30233500	12.63037300
C	0.86545700	2.60628900	12.59126100
C	0.96496300	3.42327300	13.72499800
C	1.52914800	2.95962000	14.91063300
C	1.99254100	1.65466000	14.97980200
C	1.89511000	0.83484000	13.85047000
C	2.57215800	-2.48682800	12.90103800
C	1.78742600	-3.40769100	12.20620900
C	2.13373100	-4.75236200	12.23215100
C	3.26717900	-5.16813700	12.92787600
C	4.04698700	-4.23992700	13.61499100
C	3.70076000	-2.89522100	13.61241900
H	4.29841000	-2.15695800	14.14184700
H	4.93305300	-4.56467000	14.15597400
H	3.54422900	-6.22002600	12.93424500

H	1.51550400	-5.47761900	11.70799700
H	0.89762800	-3.06905500	11.67745000
H	2.42825300	1.24755300	15.89000500
H	1.59648600	3.61222100	15.77733800
H	0.58844200	4.44328100	13.67022100
H	0.42387900	3.01866100	11.68662100
N	3.46652200	2.68077900	7.70831600
N	4.05218200	3.60135500	8.31340800
C	2.87731800	3.09518300	6.46435500
C	4.58824600	3.47082600	9.59897800
C	3.14513500	2.36912500	5.30731400
C	2.03950200	4.20841200	6.44421300
C	4.09265000	2.64145200	10.61459300
C	5.64600200	4.35225000	9.86634400
H	3.83253400	1.52648800	5.33312000
C	2.56782800	2.76961400	4.10895700
C	1.45069500	4.58566900	5.24363100
H	1.84262200	4.74847300	7.36855900
C	4.66431300	2.68833700	11.87637300
H	3.23954800	1.99263800	10.43703000
C	6.24237400	4.35630100	11.11746200
H	5.98922900	5.01122600	9.07144100
H	2.79010200	2.22057000	3.19671200
C	1.71353000	3.86913100	4.07833800
H	0.78397600	5.44456600	5.21953600
H	4.25272500	2.06440300	12.67045200
C	5.74522400	3.53268700	12.12661800
H	7.07840300	5.02218100	11.31702700
H	1.25249800	4.17135300	3.14073400
H	6.19146800	3.56209900	13.11838600
N	4.85747200	0.01483300	7.43178200
N	5.10926500	-0.70125500	6.44050100
C	6.01394500	0.63766100	8.01371000
C	4.14617400	-1.35999400	5.67592900
C	6.21603800	0.53182400	9.38773100
C	6.89201300	1.34968500	7.19919900
C	2.77648100	-1.05665200	5.57629000
C	4.68427600	-2.41813900	4.92428900
H	5.51806800	-0.03993400	9.99914100
C	7.33648900	1.13199400	9.94791100
C	7.98622200	1.97820700	7.78016500
H	6.70172200	1.41860100	6.12966200
C	1.96633700	-1.83239000	4.76412000
H	2.34480400	-0.21185100	6.10938800

C	3.86031300	-3.20251300	4.13466900
H	5.75235700	-2.60989400	4.99767000
H	7.52101600	1.03309200	11.01541400
C	8.21183900	1.86494600	9.15006000
H	8.66705000	2.55511400	7.15842400
H	0.90910200	-1.59292100	4.67792700
C	2.50036200	-2.90930300	4.05487900
H	4.27738900	-4.03300800	3.57099500
H	9.07308600	2.35387400	9.60054400
H	1.85240800	-3.51301100	3.42320000

TS1':

Pd	3.78898800	1.13002300	8.34662400
Pd	1.58587700	-0.11996800	11.44199800
I	1.50420100	1.84813800	9.60370300
I	3.75162700	-1.06585900	9.86347500
N	0.92705700	-1.18793500	14.05478800
N	1.59670300	-1.47579900	13.02740300
N	3.97357700	2.77862800	7.04109100
N	4.52292100	2.56050300	5.92605400
C	0.21062200	0.65809200	12.66510000
C	-0.61361700	1.76188800	12.51812500
C	-1.46165700	2.14958900	13.56410400
C	-1.49622700	1.45301300	14.76919100
C	-0.68327800	0.34244400	14.93830400
C	0.15783500	-0.05227900	13.89376700
C	2.42734100	-2.62594200	13.11140800
C	2.47393000	-3.49091300	12.01760700
C	3.27885400	-4.62174100	12.07984000
C	4.04318800	-4.87320300	13.21751200
C	3.99035500	-4.00181400	14.30364300
C	3.17826800	-2.87632500	14.26032900
C	3.41483200	4.06238200	7.23228500
C	3.46503000	4.60913700	8.51816900
C	2.96300000	5.88510100	8.72886900
C	2.38280200	6.58801000	7.67461200
C	2.32220800	6.02719900	6.39831600
C	2.84803600	4.76672800	6.16439000
C	5.22369200	0.47339700	6.97914600
C	5.08025000	1.29831500	5.81886800
C	5.52063700	0.86197200	4.57619000
C	6.07110000	-0.41430300	4.45875800
C	6.20456200	-1.23781500	5.57568600
C	5.79942700	-0.79498500	6.83191400

H	3.12873700	-2.18202100	15.09590400
H	4.58807800	-4.19963900	15.19062600
H	4.68164900	-5.75301200	13.25765700
H	3.30506700	-5.30992000	11.23788800
H	1.85191900	-3.29222000	11.14600400
H	-0.68405800	-0.23526900	15.86034300
H	-2.15812300	1.77466900	15.56920500
H	-2.10542500	3.01614200	13.42387000
H	-0.62472700	2.34388500	11.59803000
H	3.92834500	4.04929000	9.32925200
H	3.02116500	6.33047700	9.71890900
H	1.97059300	7.57954100	7.84783800
H	1.85870000	6.57645800	5.58246800
H	2.80857400	4.31087900	5.17840300
H	5.40968800	1.51490000	3.71386500
H	6.41060200	-0.76424900	3.48733500
H	6.65041100	-2.22378600	5.47234600
H	5.97564200	-1.41525500	7.71026600
N	6.28891300	1.16187800	8.40016300
O	6.67407600	2.23001200	8.24639800

TS2':

Pd	3.88848100	2.29912300	8.83181100
Pd	3.33137800	0.46114200	12.13286900
I	1.78478500	1.90661500	10.47269000
I	4.80010500	-0.14225100	9.78640500
N	4.31326600	-0.19550600	14.77526000
N	4.43848000	-0.56161700	13.57553300
N	4.63112900	4.34666900	9.67654700
N	4.91623600	5.32131600	8.94818600
C	2.55957900	1.18214300	13.83426700
C	1.53457300	2.08753000	14.06352500
C	1.29862400	2.57189900	15.35587700
C	2.07810300	2.16976400	16.43799100
C	3.09477600	1.24798800	16.23925700
C	3.32807700	0.75636500	14.95048700
C	5.40619000	-1.57015600	13.32144300
C	5.11475100	-2.53371300	12.35538900
C	6.03217700	-3.54687600	12.10675600
C	7.23937100	-3.58106900	12.80127600
C	7.52426300	-2.60967400	13.75972600
C	6.60806900	-1.60302800	14.03053600
C	4.32455100	4.67064400	11.01906700
C	4.71645400	3.75947200	12.00353700

C	4.44456900	4.04225300	13.33483700
C	3.74173700	5.19704600	13.67209700
C	3.33469900	6.09073400	12.68066400
C	3.63902800	5.84340000	11.35029400
C	5.57768600	3.86291900	6.95132200
C	5.26944800	5.08801700	7.60913400
C	5.46747200	6.27387900	6.90234300
C	5.92161200	6.27674300	5.58917900
C	6.23106700	5.07507800	4.95703800
C	6.06926400	3.88082600	5.63868800
H	6.81106300	-0.83693300	14.77523200
H	8.46839900	-2.63588200	14.29936900
H	7.96219600	-4.36759200	12.59570300
H	5.80302700	-4.31213200	11.36852500
H	4.16075100	-2.49788900	11.83117800
H	3.71662300	0.89443600	17.05922400
H	1.88424700	2.56690800	17.43127900
H	0.48977400	3.28443800	15.50984300
H	0.90772300	2.44884600	13.25046200
H	5.25013300	2.85388100	11.71561100
H	4.76264800	3.34845700	14.11164400
H	3.50055800	5.39710000	14.71438800
H	2.77692900	6.98513200	12.94895000
H	3.33395400	6.52737800	10.56108400
H	5.25827700	7.20369900	7.42598300
H	6.04425800	7.22086500	5.06490000
H	6.59117300	5.06553000	3.93212800
H	6.28656500	2.93102900	5.15739100
N	5.37757700	2.57235600	7.52474200
O	5.97801800	1.64396900	7.04221400
N	2.48261700	3.07487300	6.89824900
N	2.53999300	2.45060300	5.81736600
C	2.21430500	4.46837000	6.77665300
C	2.76456100	1.06465500	5.89056900
C	1.83305100	5.15208800	7.93246700
C	2.35184300	5.15232900	5.56238100
C	2.14068600	0.25640300	6.84672100
C	3.59262200	0.50691000	4.91205600
H	1.70392400	4.60688100	8.86723100
C	1.59645300	6.52062000	7.87867200
C	2.13014100	6.51882400	5.52251700
H	2.64173700	4.60074000	4.67134300
C	2.38004900	-1.11061400	6.83805100
H	1.44329500	0.69814500	7.55506200

C	3.86326400	-0.85402400	4.94555900
H	4.02890900	1.15424800	4.15335700
H	1.28538000	7.05127800	8.77612100
C	1.75459400	7.20660800	6.67830400
H	2.25039500	7.05590800	4.58393800
H	1.88223600	-1.74782000	7.56643500
C	3.25447700	-1.66327100	5.90431500
H	4.53258900	-1.29026400	4.20761600
H	1.58134200	8.27974700	6.63862600
H	3.44582700	-2.73420100	5.90877600

TS3’:

Pd	3.49897100	1.69894200	8.49917900
Pd	1.74453100	-0.25614800	11.48547700
I	0.99645100	1.06315300	9.27264200
I	4.29763000	-0.11849100	10.31773600
N	1.24956700	-1.18327100	14.18596400
N	2.16095000	-1.16359300	13.31575000
N	6.21959300	3.18059100	8.67455000
N	6.01833200	4.31429000	8.15364800
C	-0.04418600	-0.21284300	12.38696200
C	-1.27985700	0.23741500	11.94695100
C	-2.38973200	0.20232900	12.80117200
C	-2.28806900	-0.27257700	14.10615800
C	-1.06372800	-0.72726300	14.57153300
C	0.04371500	-0.69857600	13.71823900
C	3.44151600	-1.61416700	13.73850400
C	4.17026500	-2.44906200	12.89155000
C	5.42298900	-2.89635000	13.29315800
C	5.94864200	-2.49515900	14.51976600
C	5.21296600	-1.65912700	15.35728700
C	3.95327800	-1.21807000	14.97436800
C	6.48804800	2.95745900	10.03709000
C	7.29647600	1.86917500	10.37083200
C	7.59188500	1.65217200	11.71035800
C	7.07740900	2.50050000	12.68716000
C	6.27094700	3.58458400	12.33482900
C	5.97273900	3.82545700	11.00356700
C	5.56116300	2.77408800	6.50999100
C	5.67833100	4.13001900	6.83422800
C	5.52191500	5.12235600	5.86204500
C	5.28849900	4.70019800	4.56601900
C	5.22706600	3.33068600	4.24404600
C	5.36660900	2.34045400	5.20827700

H	3.36515900	-0.56235300	15.61241500
H	5.62369400	-1.34538800	16.31450900
H	6.93476700	-2.83788800	14.82563000
H	5.98957400	-3.56243200	12.64619600
H	3.73702000	-2.76750000	11.94448500
H	-0.94021000	-1.10918000	15.58294700
H	-3.16161300	-0.28768100	14.75301700
H	-3.34986200	0.55637300	12.42926500
H	-1.41673300	0.62181200	10.93712600
H	7.68203300	1.21917500	9.59033800
H	8.22648100	0.81502900	11.99101000
H	7.30815500	2.31955400	13.73487300
H	5.87712500	4.24574800	13.10296500
H	5.35515700	4.66721000	10.69704600
H	5.60176800	6.17333100	6.12874000
H	5.17245400	5.43574600	3.77442300
H	5.06964400	3.03945000	3.20817300
H	5.33065600	1.28142700	4.96453200
N	5.66853400	1.96875400	7.67002500
O	6.24823400	0.86438800	7.65371600
N	2.59972900	3.17528000	7.28570000
N	2.01672700	3.06792000	6.18452200
C	2.64307900	4.50428200	7.82040000
C	1.88143500	1.86741200	5.49184400
C	2.81332600	4.67271800	9.19687100
C	2.54739700	5.61483000	6.97641900
C	2.57916100	0.66444500	5.70830800
C	0.93304300	1.94274800	4.45780900
H	2.85975400	3.80309100	9.85448300
C	2.89910300	5.95620900	9.72466000
C	2.64487600	6.88839400	7.51359900
H	2.42412100	5.46541600	5.90698900
C	2.29880700	-0.43809600	4.91953600
H	3.34889600	0.58307000	6.47575600
C	0.64876200	0.82931300	3.68388700
H	0.42424200	2.89080200	4.29817800
H	3.01934400	6.08733500	10.79801700
C	2.82675800	7.06331500	8.88555800
H	2.58794300	7.75321400	6.85645400
H	2.84275100	-1.36543300	5.08312200
C	1.33087700	-0.36246000	3.91599500
H	-0.09745500	0.89148500	2.89594800
H	2.90898800	8.06545400	9.30012300
H	1.11718400	-1.23753300	3.30628400

TS4':

Pd	3.30445700	-0.23222300	8.48650400
Pd	0.89718200	-0.53688400	10.85215800
I	0.59545600	-0.16550700	8.20357000
I	2.95729100	-2.33666900	10.10385600
N	0.27522000	0.07445300	13.61945900
N	0.78126000	-0.84161500	12.91687200
C	-0.10225200	1.08765100	11.45167700
C	-0.56327000	2.18382600	10.73697300
C	-1.07656400	3.29857000	11.41320200
C	-1.14295200	3.34128800	12.80388400
C	-0.69580000	2.25410700	13.53978000
C	-0.17684000	1.14150100	12.86930800
C	1.24265600	-1.99056000	13.61453500
C	1.08739400	-3.23697000	13.00721100
C	1.51151800	-4.37887600	13.67458500
C	2.10773500	-4.27378300	14.92917700
C	2.26233600	-3.02468900	15.52774900
C	1.82390500	-1.87835000	14.87930900
H	1.92921300	-0.89800700	15.33898200
H	2.72777700	-2.94171900	16.50748300
H	2.45164300	-5.16839600	15.44373900
H	1.37454900	-5.35417200	13.21273200
H	0.60952900	-3.30514200	12.03093100
H	-0.72920000	2.24430800	14.62743700
H	-1.54567700	4.21691200	13.30683400
H	-1.43354100	4.14642600	10.83044800
H	-0.53362100	2.19830800	9.64932800
N	3.78757500	1.27178100	7.11104800
N	4.81315000	1.04638100	6.41011500
C	3.05264100	2.44316100	6.81191000
C	5.57975100	-0.01142400	6.89079200
C	2.30119300	3.00785400	7.84579500
C	3.14649500	3.06923000	5.56448600
C	5.38272600	-0.45926300	8.23570500
C	6.59421100	-0.51631100	6.08417200
H	2.26029700	2.51275100	8.81581600
C	1.65507300	4.21809600	7.63741600
C	2.47996500	4.26740800	5.36325500
H	3.73874100	2.61050100	4.77695900
C	6.23452200	-1.47178700	8.70145300
C	7.42533900	-1.50887000	6.59339300
H	6.72251400	-0.12475000	5.07727500

H	1.08521500	4.67169900	8.44574400
C	1.74062000	4.84550900	6.39638200
H	2.53754800	4.75769300	4.39427000
H	6.12085600	-1.84999500	9.71694600
C	7.25285400	-1.97718100	7.89659500
H	8.22107900	-1.91483600	5.97356900
H	1.22697100	5.78985500	6.23016500
H	7.91946800	-2.74246000	8.28754000
N	5.50377100	2.91139900	9.38801700
N	5.36707700	1.76935300	9.88862000
C	6.33925700	3.03780500	8.27653500
C	4.58392100	1.67963200	11.06955900
C	7.45195100	2.21456400	8.04724500
C	6.05129700	4.11016300	7.42043000
C	3.67093700	2.67568300	11.43090600
C	4.79773200	0.56549200	11.87977700
H	7.71135300	1.43655500	8.76436500
C	8.23903500	2.44476500	6.93047200
C	6.82173000	4.30139100	6.28497800
H	5.20259800	4.75297800	7.65085100
C	2.98176800	2.55085300	12.62587900
H	3.52539200	3.53276500	10.77670200
C	4.11380100	0.45954300	13.08493300
H	5.51898600	-0.19130300	11.57495900
H	9.11310200	1.82267600	6.74964400
C	7.91428000	3.46964100	6.04092100
H	6.58597500	5.11073200	5.59811800
H	2.25942600	3.31163800	12.91737400
C	3.20971500	1.45089000	13.45607800
H	4.28283200	-0.40133600	13.72940900
H	8.53416200	3.63698800	5.16289900
H	2.66969700	1.37197800	14.39878000
H	5.38958900	0.60477400	9.13200700

TSA’:

Pd	3.65401800	2.68991600	9.88519200
Pd	1.39943900	0.49111900	11.88350500
I	1.68911100	3.16425700	11.63795600
I	2.47720200	0.40682600	9.24959900
N	1.04891200	-1.90371300	13.47225200
N	1.21261000	-1.54764300	12.27508000
N	4.76673100	2.61939900	7.04765800
N	5.04587800	1.49850900	6.58644900
C	0.90988400	0.48777800	13.82270000

C	0.67158700	1.52806900	14.70694400
C	0.40667600	1.25632400	16.05602500
C	0.38394700	-0.04627100	16.54674500
C	0.61498800	-1.10385600	15.68035500
C	0.87013300	-0.83810500	14.33157700
C	1.42065000	-2.58978500	11.32980600
C	0.76555400	-2.51543400	10.10034400
C	0.96091300	-3.52512000	9.16596900
C	1.81996200	-4.58495400	9.45133900
C	2.47122800	-4.64724100	10.68132000
C	2.27024900	-3.65364200	11.63096800
C	3.73808700	3.44979200	6.59373600
C	3.79612000	4.79953300	6.96078900
C	2.78034200	5.64452500	6.54062700
C	1.73338300	5.14429900	5.76810500
C	1.69084800	3.79559900	5.40721200
C	2.69072700	2.93267900	5.82045300
C	6.17419900	1.49214700	8.62932300
C	5.92060200	0.82692500	7.42560700
C	6.55629400	-0.37469000	7.12334200
C	7.46191500	-0.86842700	8.05101400
C	7.72762000	-0.17796500	9.24121100
C	7.09178300	1.02278200	9.54949300
H	2.77223600	-3.68168700	12.59542700
H	3.14249400	-5.47406100	10.90276000
H	1.98100700	-5.36650500	8.71202400
H	0.43758500	-3.48511900	8.21323200
H	0.08390800	-1.68939200	9.90107300
H	0.59921900	-2.13780200	16.01889600
H	0.18162400	-0.23250600	17.59839900
H	0.21527800	2.08960000	16.73005700
H	0.68113600	2.56484900	14.37775800
H	4.63093200	5.16576300	7.55398200
H	2.80770200	6.69717300	6.80939600
H	0.93894200	5.81168600	5.44224300
H	0.86682700	3.41734800	4.80754400
H	2.67544700	1.87539900	5.56607100
H	6.35435500	-0.87859200	6.18221400
H	7.98361900	-1.79953400	7.84796300
H	8.44285100	-0.59116300	9.94790100
H	7.27407600	1.55762600	10.47835400
N	5.36741900	2.64512000	8.80680800
O	5.81003100	3.69321500	9.30097100
