# Supplementary material for: Iodide-enhanced palladium catalysis via formation of iodide-bridged binuclear palladium complex
Source: Commun Chem. 2020 Mar 31;3:41. doi: 10.1038/s42004-020-0287-0 (PMC9814094; doi:10.1038/s42004-020-0287-0)

checkCIF/PLATON report

You have not supplied any structure factors. As a result the full set of tests cannot be run.

THIS REPORT IS FOR GUIDANCE ONLY. IF USED AS PART OF A REVIEW PROCEDURE FOR PUBLICATION, IT SHOULD NOT REPLACE THE EXPERTISE OF AN EXPERIENCED CRYSTALLOGRAPHIC REFEREE.

No syntax errors found.   CIF dictionary   Interpreting this report

Datablock: 3a

```
Bond precision:  C-C = 0.0030 A          Wavelength=1.54184

Cell:            a=6.4226(2)     b=23.1159(8)        c=6.9516(3)
                 alpha=90        beta=107.696(4)     gamma=90
Temperature:     100 K

                Calculated                 Reported
Volume          983.23(7)                  983.23(7)
Space group     C c                        C c
Hall group      C -2yc                     C -2yc
Moiety formula  C12 H9 N3 O                C12 H9 N3 O
Sum formula     C12 H9 N3 O                C12 H9 N3 O
Mr              211.22                     211.22
Dx,g cm-3       1.427                      1.427
Z               4                          4
Mu (mm-1)       0.776                      0.776
F000            440.0                      440.0
F000'           441.33
h,k,lmax        7,28,8                     7,27,8
Nref            1954[ 984]                 1119
Tmin,Tmax       0.815,0.940                0.761,1.000
Tmin'           0.780

Correction method= # Reported T Limits: Tmin=0.761 Tmax=1.000
AbsCorr = MULTI-SCAN

Data completeness= 1.14/0.57      Theta(max)= 72.787

R(reflections)= 0.0243( 1100)     wR2(reflections)= 0.0638( 1119)

S = 1.092                  Npar= 146
```

The following ALERTS were generated. Each ALERT has the format
test-name_ALERT_alert-type_alert-level.
Click on the hyperlinks for more details of the test.

Alert level C

```
PLAT089_ALERT_3_C Poor Data / Parameter Ratio (Zmax < 18) ........       6.74 Note
```

Alert level G

```
PLAT005_ALERT_5_G No Embedded Refinement Details Found  in the CIF     Please Do !
PLAT032_ALERT_4_G Std. Uncertainty on Flack Parameter Value High .      0.300 Report
PLAT850_ALERT_4_G Check Flack Parameter Exact Value 0.00 and s.u.       0.30 Check
PLAT883_ALERT_1_G No Info/Value for _atom_sites_solution_primary .     Please Do !
```

```
   0 ALERT level A = Most likely a serious problem - resolve or explain
   0 ALERT level B = A potentially serious problem, consider carefully
   1 ALERT level C = Check. Ensure it is not caused by an omission or oversight
   4 ALERT level G = General information/check it is not something unexpected

   1 ALERT type 1 CIF construction/syntax error, inconsistent or missing data
   0 ALERT type 2 Indicator that the structure model may be wrong or deficient
   1 ALERT type 3 Indicator that the structure quality may be low
   2 ALERT type 4 Improvement, methodology, query or suggestion
   1 ALERT type 5 Informative message, check
```

It is advisable to attempt to resolve as many as possible of the alerts in all categories. Often the minor alerts point to easily fixed oversights, errors and omissions in your CIF or refinement strategy, so attention to these fine details can be worthwhile. In order to resolve some of the more serious problems it may be necessary to carry out additional measurements or structure refinements. However, the purpose of your study may justify the reported deviations and the more serious of these should normally be commented upon in the discussion or experimental section of a paper or in the "special_details" fields of the CIF. checkCIF was carefully designed to identify outliers and unusual parameters, but every test has its limitations and alerts that are not important in a particular case may appear. Conversely, the absence of alerts does not guarantee there are no aspects of the results needing attention. It is up to the individual to critically assess their own results and, if necessary, seek expert advice.

Publication of your CIF in IUCr journals

A basic structural check has been run on your CIF. These basic checks will be run on all CIFs submitted for publication in IUCr journals (*Acta Crystallographica*, *Journal of Applied Crystallography*, *Journal of Synchrotron Radiation*); however, if you intend to submit to *Acta Crystallographica Section C* or *E* or *IUCrData*, you should make sure that full publication checks are run on the final version of your CIF prior to submission.

Publication of your CIF in other journals

Please refer to the *Notes for Authors* of the relevant journal for any special instructions relating to CIF submission.

PLATON version of 03/05/2019; check.def file version of 29/04/2019

Datablock 3a - ellipsoid plot

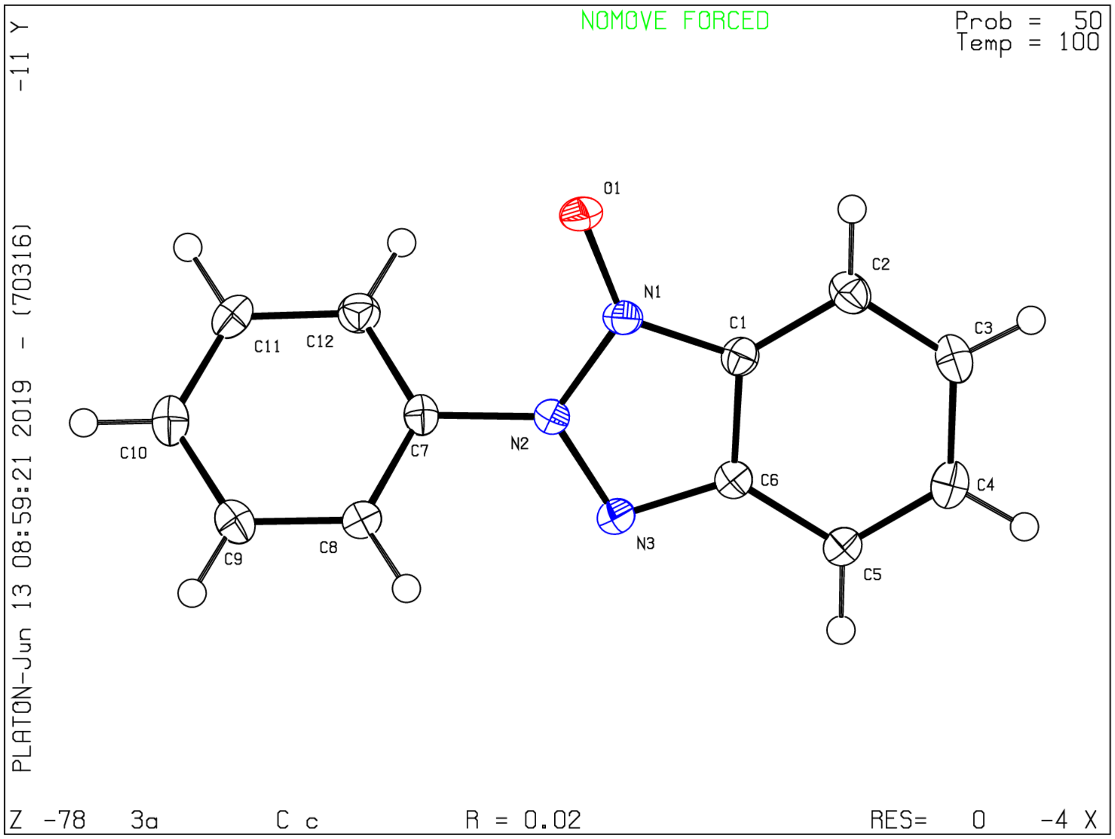

checkCIF/PLATON report

You have not supplied any structure factors. As a result the full set of tests cannot be run.

THIS REPORT IS FOR GUIDANCE ONLY. IF USED AS PART OF A REVIEW PROCEDURE FOR PUBLICATION, IT SHOULD NOT REPLACE THE EXPERTISE OF AN EXPERIENCED CRYSTALLOGRAPHIC REFEREE.

No syntax errors found. CIF dictionary Interpreting this report

Datablock: 4a

```
Bond precision:  C-C = 0.0071 A          Wavelength=1.54178

Cell:           a=4.1853(1)        b=10.9291(3)     c=13.2771(3)
                alpha=110.318(2)   beta=90.072(2)   gamma=96.088(2)
Temperature:    100 K

                Calculated                    Reported
Volume          565.84(3)                     565.84(2)
Space group     P -1                          P-1
Hall group      -P 1                          -P 1
Moiety formula  C24 H18 I2 N4 Pd2             C24 H18 I2 N4 Pd2
Sum formula     C24 H18 I2 N4 Pd2             C24 H18 I2 N4 Pd2
Mr              829.02                        829.02
Dx,g cm-3       2.433                         2.433
Z               1                             1
Mu (mm-1)       34.402                        34.402
F000            388.0                         388.0
F000'           388.73
h,k,lmax        5,13,16                       5,13,16
Nref            2132                          2125
Tmin,Tmax       0.159,0.503                   0.063,0.546
Tmin'           0.002

Correction method= # Reported T Limits: Tmin=0.063 Tmax=0.546
AbsCorr = MULTI-SCAN

Data completeness= 0.997          Theta(max)= 69.950

R(reflections)= 0.0268( 2014)     wR2(reflections)= 0.0690( 2125)

S = 1.079                  Npar= 145
```

The following ALERTS were generated. Each ALERT has the format
test-name_ALERT_alert-type_alert-level.
Click on the hyperlinks for more details of the test.

Alert level G

```
PLAT005_ALERT_5_G No Embedded Refinement Details Found  in the CIF     Please Do !
PLAT154_ALERT_1_G The s.u.'s on the Cell Angles are Equal ..(Note)      0.002 Degree
PLAT232_ALERT_2_G Hirshfeld Test Diff (M-X)  I1       --Pd1      .       14.0 s.u.
PLAT232_ALERT_2_G Hirshfeld Test Diff (M-X)  I1       --Pd1_a    .       15.0 s.u.
PLAT794_ALERT_5_G Tentative Bond Valency for Pd1       (II)      .       1.93 Info
PLAT899_ALERT_4_G SHELXL97   is Deprecated and Succeeded by SHELXL       2018 Note
```

```
   0 ALERT level A = Most likely a serious problem - resolve or explain
   0 ALERT level B = A potentially serious problem, consider carefully
   0 ALERT level C = Check. Ensure it is not caused by an omission or oversight
   6 ALERT level G = General information/check it is not something unexpected

   1 ALERT type 1 CIF construction/syntax error, inconsistent or missing data
   2 ALERT type 2 Indicator that the structure model may be wrong or deficient
   0 ALERT type 3 Indicator that the structure quality may be low
   1 ALERT type 4 Improvement, methodology, query or suggestion
   2 ALERT type 5 Informative message, check
```

It is advisable to attempt to resolve as many as possible of the alerts in all categories. Often the minor alerts point to easily fixed oversights, errors and omissions in your CIF or refinement strategy, so attention to these fine details can be worthwhile. In order to resolve some of the more serious problems it may be necessary to carry out additional measurements or structure refinements. However, the purpose of your study may justify the reported deviations and the more serious of these should normally be commented upon in the discussion or experimental section of a paper or in the "special_details" fields of the CIF. checkCIF was carefully designed to identify outliers and unusual parameters, but every test has its limitations and alerts that are not important in a particular case may appear. Conversely, the absence of alerts does not guarantee there are no aspects of the results needing attention. It is up to the individual to critically assess their own results and, if necessary, seek expert advice.

Publication of your CIF in IUCr journals

A basic structural check has been run on your CIF. These basic checks will be run on all CIFs submitted for publication in IUCr journals (*Acta Crystallographica*, *Journal of Applied Crystallography*, *Journal of Synchrotron Radiation*); however, if you intend to submit to *Acta Crystallographica Section C* or *E* or *IUCrData*, you should make sure that full publication checks are run on the final version of your CIF prior to submission.

Publication of your CIF in other journals

Please refer to the *Notes for Authors* of the relevant journal for any special instructions relating to CIF submission.

PLATON version of 03/05/2019; check.def file version of 29/04/2019

Datablock 4a - ellipsoid plot

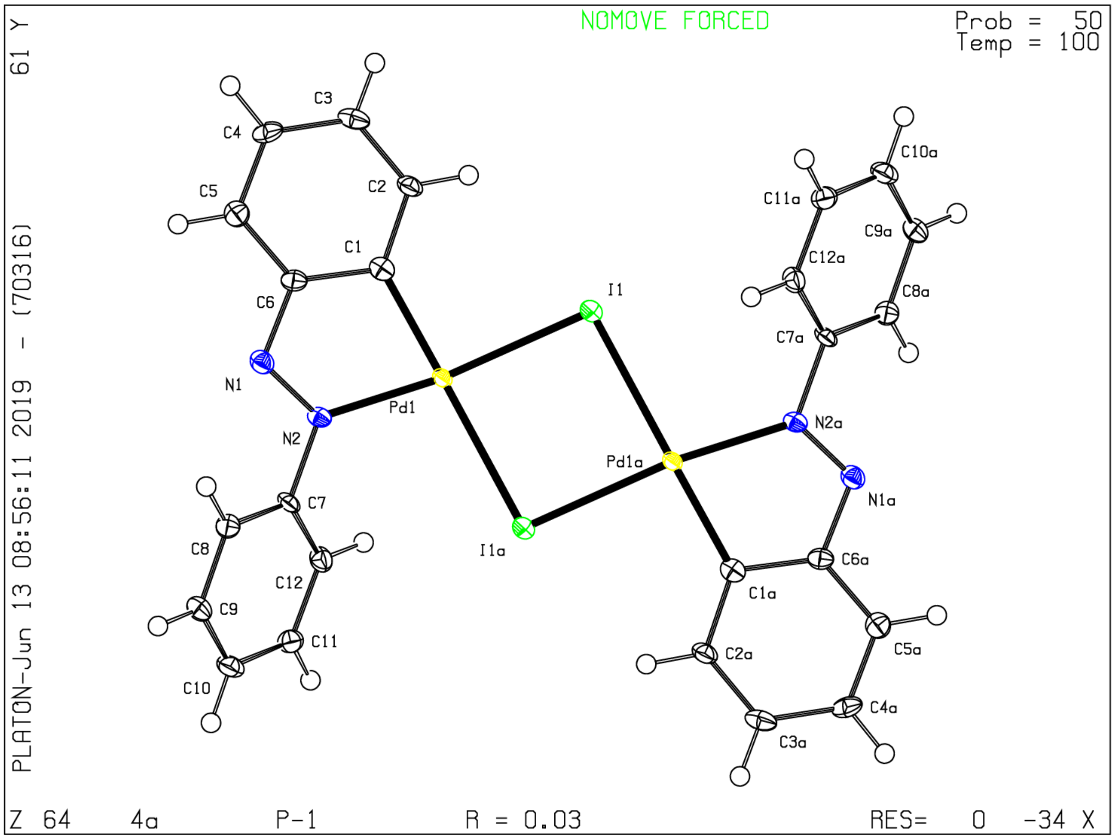

checkCIF/PLATON report

You have not supplied any structure factors. As a result the full set of tests cannot be run.

THIS REPORT IS FOR GUIDANCE ONLY. IF USED AS PART OF A REVIEW PROCEDURE FOR PUBLICATION, IT SHOULD NOT REPLACE THE EXPERTISE OF AN EXPERIENCED CRYSTALLOGRAPHIC REFEREE.

No syntax errors found.   CIF dictionary   Interpreting this report

Datablock: 4b

```
Bond precision:  C-C = 0.0057 A          Wavelength=1.54178

Cell:          a=4.6320(1)       b=13.8323(5)     c=16.6380(6)
               alpha=65.897(2)   beta=85.801(2)   gamma=81.030(2)
Temperature:   100 K

                Calculated                Reported
Volume          961.12(6)                 961.12(5)
Space group     P -1                      P -1
Hall group      -P 1                      -P 1
Moiety formula  C40 H50 I2 N4 Pd2         C40 H50 I2 N4 Pd2
Sum formula     C40 H50 I2 N4 Pd2         C40 H50 I2 N4 Pd2
Mr              1053.44                   1053.44
Dx,g cm-3       1.820                     1.820
Z               1                         1
Mu (mm-1)       20.405                    20.405
F000            516.0                     516.0
F000'           516.85
h,k,lmax        5,16,20                   5,16,20
Nref            3657                      3630
Tmin,Tmax       0.028,0.361               0.094,0.428
Tmin'           0.004

Correction method= # Reported T Limits: Tmin=0.094 Tmax=0.428
AbsCorr = MULTI-SCAN

Data completeness= 0.993          Theta(max)= 69.990

R(reflections)= 0.0387( 3594)     wR2(reflections)= 0.1047( 3630)

S = 1.044                  Npar= 217
```

The following ALERTS were generated. Each ALERT has the format
test-name_ALERT_alert-type_alert-level.
Click on the hyperlinks for more details of the test.

Alert level G

```
PLAT005_ALERT_5_G No Embedded Refinement Details Found  in the CIF     Please Do !
PLAT154_ALERT_1_G The s.u.'s on the Cell Angles are Equal ..(Note)      0.002 Degree
PLAT232_ALERT_2_G Hirshfeld Test Diff (M-X)  I1       --Pd1      .        6.0 s.u.
PLAT794_ALERT_5_G Tentative Bond Valency for Pd1       (II)      .       1.89 Info
PLAT899_ALERT_4_G SHELXL97   is Deprecated and Succeeded by SHELXL       2018 Note
```

```
   0 ALERT level A = Most likely a serious problem - resolve or explain
   0 ALERT level B = A potentially serious problem, consider carefully
   0 ALERT level C = Check. Ensure it is not caused by an omission or oversight
   5 ALERT level G = General information/check it is not something unexpected

   1 ALERT type 1 CIF construction/syntax error, inconsistent or missing data
   1 ALERT type 2 Indicator that the structure model may be wrong or deficient
   0 ALERT type 3 Indicator that the structure quality may be low
   1 ALERT type 4 Improvement, methodology, query or suggestion
   2 ALERT type 5 Informative message, check
```

It is advisable to attempt to resolve as many as possible of the alerts in all categories. Often the minor alerts point to easily fixed oversights, errors and omissions in your CIF or refinement strategy, so attention to these fine details can be worthwhile. In order to resolve some of the more serious problems it may be necessary to carry out additional measurements or structure refinements. However, the purpose of your study may justify the reported deviations and the more serious of these should normally be commented upon in the discussion or experimental section of a paper or in the "special_details" fields of the CIF. checkCIF was carefully designed to identify outliers and unusual parameters, but every test has its limitations and alerts that are not important in a particular case may appear. Conversely, the absence of alerts does not guarantee there are no aspects of the results needing attention. It is up to the individual to critically assess their own results and, if necessary, seek expert advice.

Publication of your CIF in IUCr journals

A basic structural check has been run on your CIF. These basic checks will be run on all CIFs submitted for publication in IUCr journals (*Acta Crystallographica*, *Journal of Applied Crystallography*, *Journal of Synchrotron Radiation*); however, if you intend to submit to *Acta Crystallographica Section C* or *E* or *IUCrData*, you should make sure that full publication checks are run on the final version of your CIF prior to submission.

Publication of your CIF in other journals

Please refer to the *Notes for Authors* of the relevant journal for any special instructions relating to CIF submission.

PLATON version of 03/05/2019; check.def file version of 29/04/2019

Datablock 4b - ellipsoid plot

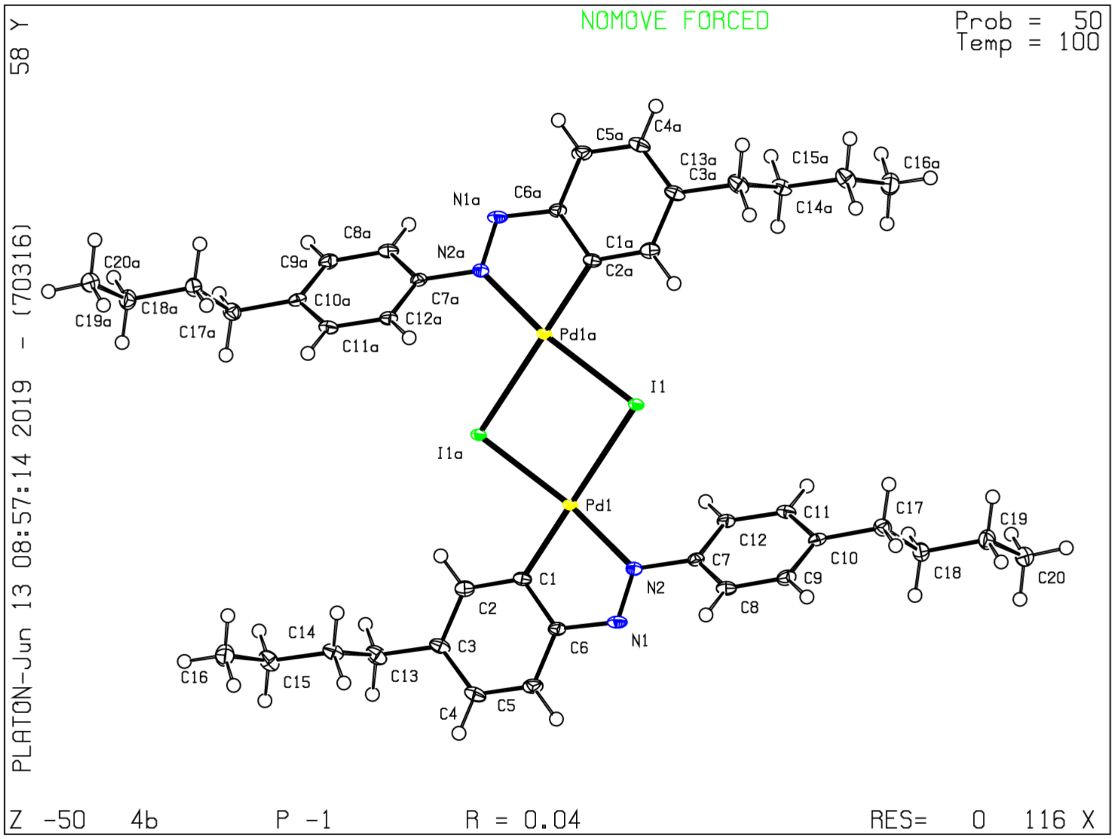

checkCIF/PLATON report

You have not supplied any structure factors. As a result the full set of tests cannot be run.

THIS REPORT IS FOR GUIDANCE ONLY. IF USED AS PART OF A REVIEW PROCEDURE FOR PUBLICATION, IT SHOULD NOT REPLACE THE EXPERTISE OF AN EXPERIENCED CRYSTALLOGRAPHIC REFEREE.

No syntax errors found. CIF dictionary Interpreting this report

Datablock: 5b

```
Bond precision:  C-C = 0.0036 A          Wavelength=1.54184

Cell:           a=10.9439(3)      b=11.2900(3)     c=17.5270(5)
                alpha=85.362(2)   beta=75.755(2)   gamma=81.146(2)
Temperature:    100 K

                Calculated                  Reported
Volume          2071.89(10)                 2071.89(9)
Space group     P -1                        P -1
Hall group      -P 1                        -P 1
Moiety formula  C44 H56 N4 O4 Pd2           C44 H56 N4 O4 Pd2
Sum formula     C44 H56 N4 O4 Pd2           C44 H56 N4 O4 Pd2
Mr              917.73                      917.73
Dx,g cm-3       1.471                       1.471
Z               2                           2
Mu (mm-1)       7.365                       7.365
F000            944.0                       944.0
F000'           946.75
h,k,lmax        13,13,21                    13,13,21
Nref            7869                        7858
Tmin,Tmax       0.213,0.413                 0.157,0.472
Tmin'           0.042

Correction method= # Reported T Limits: Tmin=0.157 Tmax=0.472
AbsCorr = MULTI-SCAN

Data completeness= 0.999          Theta(max)= 70.000

R(reflections)= 0.0277( 7309)     wR2(reflections)= 0.0772( 7858)

S = 1.051                   Npar= 506
```

The following ALERTS were generated. Each ALERT has the format
test-name_ALERT_alert-type_alert-level.
Click on the hyperlinks for more details of the test.

Alert level C

```
PLAT601_ALERT_2_C Structure Contains Solvent Accessible VOIDS of .         32 Ang**3
```

Alert level G

```
PLAT005_ALERT_5_G No Embedded Refinement Details Found  in the CIF     Please Do !
PLAT154_ALERT_1_G The s.u.'s on the Cell Angles are Equal ..(Note)      0.002 Degree
PLAT232_ALERT_2_G Hirshfeld Test Diff (M-X)  Pd2      --C21      .        6.0 s.u.
PLAT301_ALERT_3_G Main Residue  Disorder .............(Resd  1  )         7% Note
PLAT380_ALERT_4_G Incorrectly? Oriented X(sp2)-Methyl Moiety .....        C42 Check
PLAT380_ALERT_4_G Incorrectly? Oriented X(sp2)-Methyl Moiety .....        C44 Check
PLAT899_ALERT_4_G SHELXL97   is Deprecated and Succeeded by SHELXL       2018 Note
```

```
   0 ALERT level A = Most likely a serious problem - resolve or explain
   0 ALERT level B = A potentially serious problem, consider carefully
   1 ALERT level C = Check. Ensure it is not caused by an omission or oversight
   7 ALERT level G = General information/check it is not something unexpected

   1 ALERT type 1 CIF construction/syntax error, inconsistent or missing data
   2 ALERT type 2 Indicator that the structure model may be wrong or deficient
   1 ALERT type 3 Indicator that the structure quality may be low
   3 ALERT type 4 Improvement, methodology, query or suggestion
   1 ALERT type 5 Informative message, check
```

It is advisable to attempt to resolve as many as possible of the alerts in all categories. Often the minor alerts point to easily fixed oversights, errors and omissions in your CIF or refinement strategy, so attention to these fine details can be worthwhile. In order to resolve some of the more serious problems it may be necessary to carry out additional measurements or structure refinements. However, the purpose of your study may justify the reported deviations and the more serious of these should normally be commented upon in the discussion or experimental section of a paper or in the "special_details" fields of the CIF. checkCIF was carefully designed to identify outliers and unusual parameters, but every test has its limitations and alerts that are not important in a particular case may appear. Conversely, the absence of alerts does not guarantee there are no aspects of the results needing attention. It is up to the individual to critically assess their own results and, if necessary, seek expert advice.

Publication of your CIF in IUCr journals

A basic structural check has been run on your CIF. These basic checks will be run on all CIFs submitted for publication in IUCr journals (*Acta Crystallographica*, *Journal of Applied Crystallography*, *Journal of Synchrotron Radiation*); however, if you intend to submit to *Acta Crystallographica Section C* or *E* or *IUCrData*, you should make sure that full publication checks are run on the final version of your CIF prior to submission.

Publication of your CIF in other journals

Please refer to the *Notes for Authors* of the relevant journal for any special instructions relating to CIF submission.

PLATON version of 03/05/2019; check.def file version of 29/04/2019

Datablock 5b - ellipsoid plot

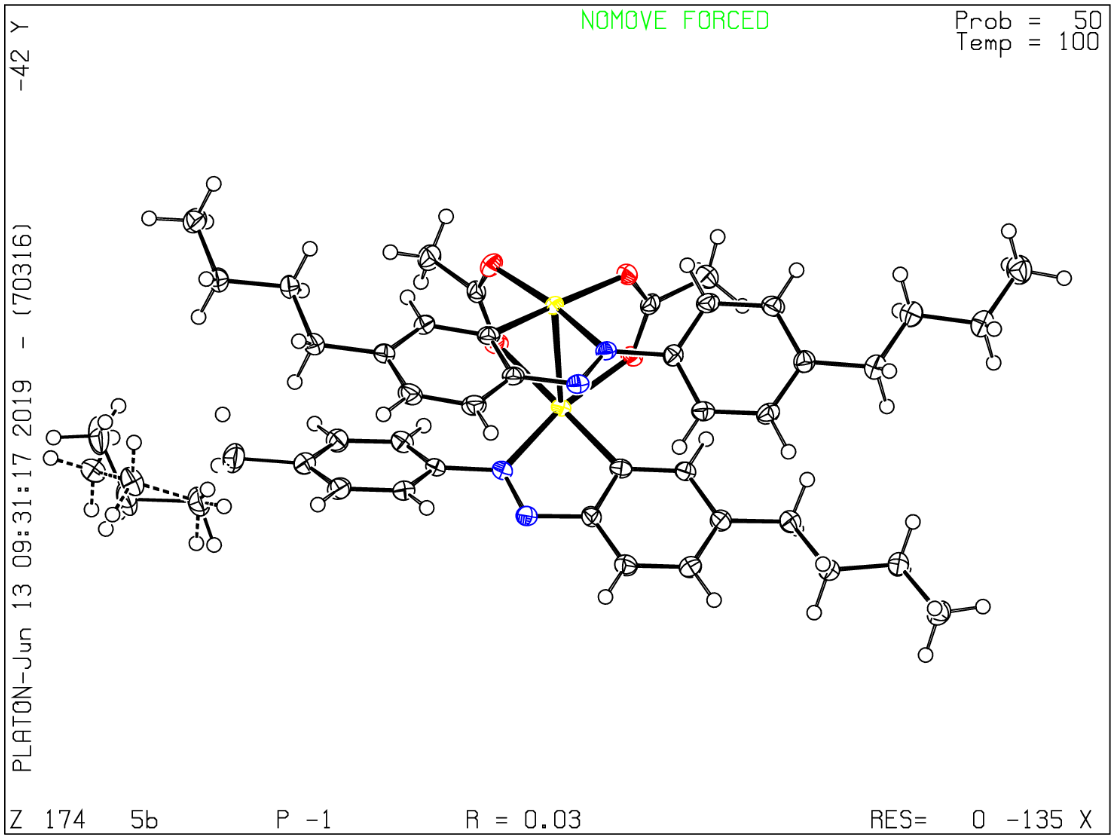

Supplement: Supplementary file 7 — Supplementary Data 6 [file 42004_2020_287_MOESM7_ESM.pdf]
