# Supplementary material for: Iodide-enhanced palladium catalysis via formation of iodide-bridged binuclear palladium complex
Source: Commun Chem. 2020 Mar 31;3:41. doi: 10.1038/s42004-020-0287-0 (PMC9814094; doi:10.1038/s42004-020-0287-0)
Supplement: Supplementary file 8 — Description of Additional Supplementary Files [file 42004_2020_287_MOESM8_ESM.pdf]

Description of Additional Supplementary Files

File Name: Supplementary Data 1
Description: DFT computed Cartesian coordinates of important structures.

File Name: Supplementary Data 2
Description: Crystallographic information file for compound **3a**.

File Name: Supplementary Data 3
Description: Crystallographic information file for compound **4a**.

File Name: Supplementary Data 4
Description: Crystallographic information file for compound **4b.**

File Name: Supplementary Data 5
Description: Crystallographic information file for compound **5b**.

File Name: Supplementary Data 6
Description: Check CIF/PLATON report file of all the four compounds.
